# Pd-catalyzed asymmetric hydrogenation of fluorinated aromatic pyrazol-5-ols *via* capture of active tautomers†
†Electronic supplementary information (ESI) available. CCDC 1040656–1040658. For ESI and crystallographic data in CIF or other electronic format see DOI: 10.1039/c5sc00835b



**DOI:** 10.1039/c5sc00835b

**Published:** 2015-03-31

**Authors:** Zhang-Pei Chen, Mu-Wang Chen, Lei Shi, Chang-Bin Yu, Yong-Gui Zhou

**Affiliations:** a State Key Laboratory of Catalysis , Dalian Institute of Chemical Physics , Chinese Academy of Sciences , Dalian 116023 , P. R. China . Email: ygzhou@dicp.ac.cn; b Collaborative Innovation Centre of Chemical Science and Engineering (Tianjin) , Tianjin 300071 , P. R. China

## Abstract

An efficient palladium-catalyzed asymmetric hydrogenation of fluorinated aromatic pyrazol-5-ols has been developed *via* capture of the active tautomers. A wide variety of 2,5-disubstituted and 2,4,5-trisubstituted pyrazolidinones have been synthesized with up to 96% and 95% ee, respectively. The hydrogenation pathway includes Brønsted acid promoted tautomerization of pyrazol-5-ols and Pd-catalyzed asymmetric hydrogenation of the active tautomer.

## Abstract 2

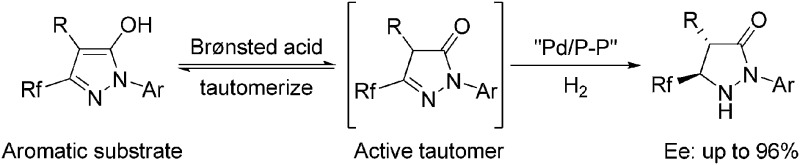
Here we explore a novel strategy for asymmetric hydrogenation of aromatic pyrazol-5-ols *via* capture of the active tautomers.

## 

Pyrazolidinones and related 1,2-diaza-3-one heterocycles are highly desirable building blocks owing to the fact that they frequently set up the core framework of numerous pharmaceutically and agrochemically active compounds.^1^ Particularly, aside from drug development, optically pure pyrazolidinones have also shown great advantages in synthetic methodology.^2^ For example, the chiral pyrazolidinones could act as efficient catalysts to promote Diels–Alder reactions, and catalyze kinetic resolution of racemic secondary alcohols.^3^ Over the last decades, the introduction of fluorine into molecules has received increasing attention in the fields of medicinal, agricultural, and material chemistry, primarily because the isosteric replacement of hydrogen by fluorine enhanced the lipophilicity, metabolic stability, and bioavailability of the parent compounds.^4^ Consequently, reliable methods toward the facile generation of optically active fluorinated pyrazolidinones would be very desirable in organic synthesis and drug research. However, the methods to chiral pyrazolidinones were still limited to transformation from chiral materials,^5^ classical chemical resolution^3*a*^ or kinetic resolution involving pyrazolidinone imides,^6^ and the synthesis of fluorinated pyrazolidinones was rarely explored. Considering the ready availability and easy preparation of fluorinated pyrazol-5-ols, asymmetric hydrogenation of these compounds would provide atom-economical and straightforward access to optically pure pyrazolidinones (Scheme 1).

**Scheme 1:**
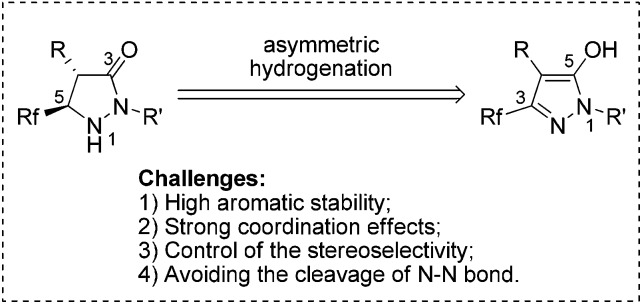
Challenges in the asymmetric hydrogenation of fluorinated pyrazol-5-ols.

Despite much progress having been achieved in the asymmetric hydrogenation of heteroaromatics^7^ including quinolines,^8^ iso-quinolines,^9^ quinoxalines,^10^ pyridines,^11^ indoles,^12^ pyrroles,^13^ (benzo)furans,^14^ (benzo)thiophenes,^15^ imidazoles,^16^ indolizines,^17^ pyrimidines,^18^ and naphthyridines,^19^ a great number of problems still remain unsettled in this field, such as the catalytic asymmetric hydrogenation of aromatic rings containing free hydroxyl, amido or other electron-enriched functional groups and heteroarenes containing two or more adjacent heteroatoms. The intrinsic problems are apparent: (i) the inherent stability resulting from aromaticity; (ii) the strong coordination effects endowed by the heteroatoms; (iii) the difficulty of controlling the stereoselectivity; (iv) the facile cleavage of the bond between the heteroatoms.^20^ Therefore, the hydrogenation of this kind of electron-enriched aromatic pyrazol-5-ol with a free hydroxyl and two adjacent nitrogen-atoms is a great and significant challenge. Herein, we wish to report our initial findings on the development of a Pd-catalyzed asymmetric hydrogenation of fluorinated pyrazol-5-ols with excellent enantioselectivities, yields, and diastereoselectivities.

At the outset, the readily available 1-phenyl-3-(trifluoromethyl)-1*H*-pyrazol-5-ol **1a**, which can be synthesized from the easily accessible starting materials ethyl 4,4,4-trifluoro-3-oxobutanoate and phenylhydrazine,^21^ was selected as the model substrate for investigation. To our disappointment, the hydrogenation failed to proceed in the presence of common Rh, Ru, and Ir catalysts (Scheme 2). This may be ascribed to the strong coordination effects and highly electron-enriched nature of fluorinated pyrazol-5-ols that impeded the hydrogenation.

**Scheme 2:**
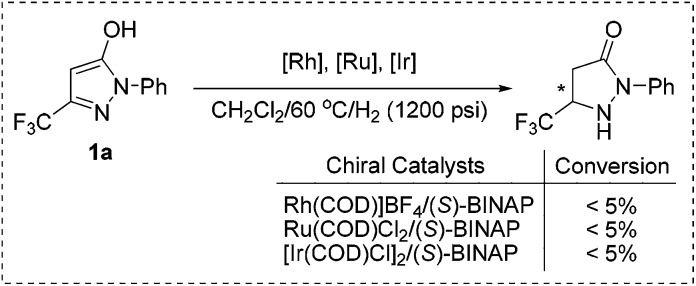
Asymmetric hydrogenation of fluorinated pyrazol-5-ol **1a**.

In principle, these kinds of substrates exist in three tautomeric forms, *i.e.* the OH– (form **A**), the NH– (form **B**) and the CH–isomer (form **C**) (Scheme 3).^1e,22^ A literature search^23^ and experimental data including X-ray crystal structure^24^ analysis demonstrated that form **A** is the most stable and dominant form. In view of the fact that the alkylation of pyrazol-5-ols would selectively lead to *O*-alkyl derivatives or *N*-alkyl derivatives as the alkylation products under specific requirements, we supposed that appropriate conditions would promote the tautomerization of the highly aromatic tautomer **A** to the more active tautomer **C**. Previous results have demonstrated that trifluoroacetic acid (TFA) could promote 1,4-dihydroxynaphthalene to form its stable tautomer tetralin-1,4-dione,^25^ and accelerate iminium–enamine isomerization to facilitate hydrogenation.^26^ On the basis of these analyses, we envisioned that the combination of a Pd catalyst which is excellently tolerant to acid^27^ and a Brønsted acid could be suitable for the asymmetric hydrogenation of fluorinated pyrazol-5-ols.

**Scheme 3:**
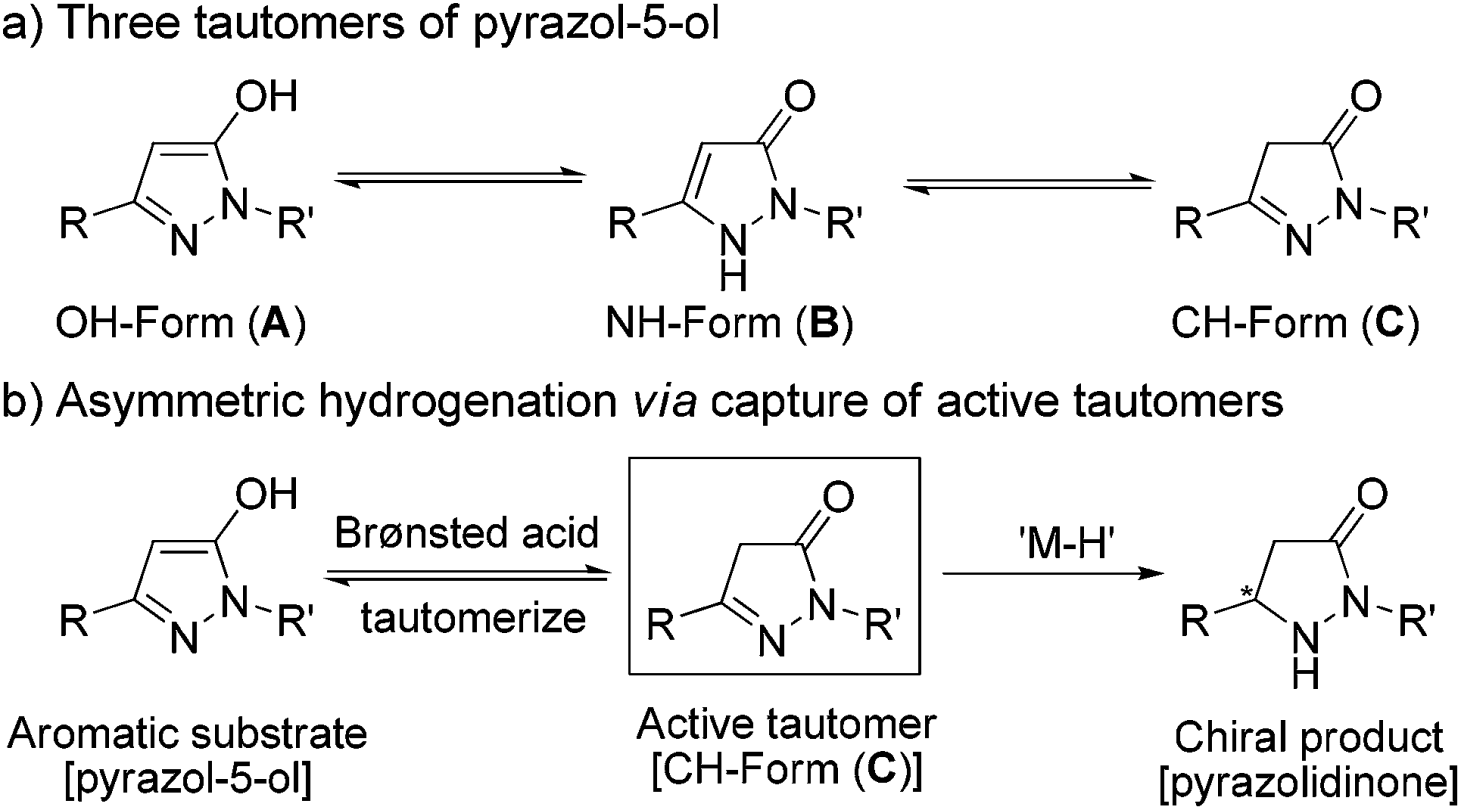
The tautomeric forms of pyrazol-5-ols and the hydrogenation strategy.

To our delight, the exposure of **1a** to TFA in dichloromethane furnished the desired pyrazolidinone **2a** with 91% ee and 54% conversion using Pd(OCOCF_3_)_2_/(*S*)-BINAP as the catalyst (Table 1, entry 1). When the reaction was carried out in 2,2,2-trifluoroethanol (TFE), the reactivity and enantioselectivity dropped slightly (entry 2). Subsequently, the effects of other acids were investigated, and TFA was the most suitable choice in view of the reactivity and enantioselectivity.

**Table 1:** Optimization of the asymmetric hydrogenation of pyrazol-5-ol **1a** ^*a*^

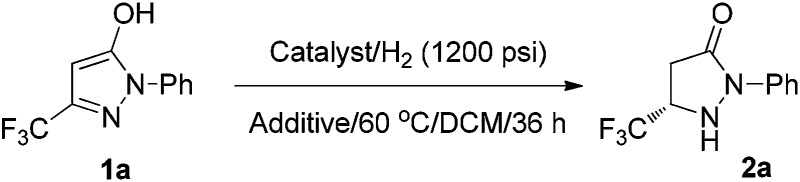				
Entry	Catalyst	Additive	Yield (%)^*b*^	Ee (%)^*c*^
1	Pd(OCOCF_3_)_2_ + **L1**	TFA	54	91
2^*d*^	Pd(OCOCF_3_)_2_ + **L1**	TFA	52	90
3	Pd(OCOCF_3_)_2_ + **L1**	l-CSA	46	90
4	Pd(OCOCF_3_)_2_ + **L1**	d-CSA	39	90
5	Pd(OCOCF_3_)_2_ + **L1**	TsOH·H_2_O	32	90
6	Pd(OCOCF_3_)_2_ + **L2**	TFA	81	96
7	Pd(OCOCF_3_)_2_ + **L3**	TFA	46	93
8	Pd(OCOCF_3_)_2_ + **L4**	TFA	29	90
9	Pd(OCOCF_3_)_2_ + **L5**	TFA	20	97
10^*e*^	Pd(OCOCF_3_)_2_ + **L2**	TFA	94	96
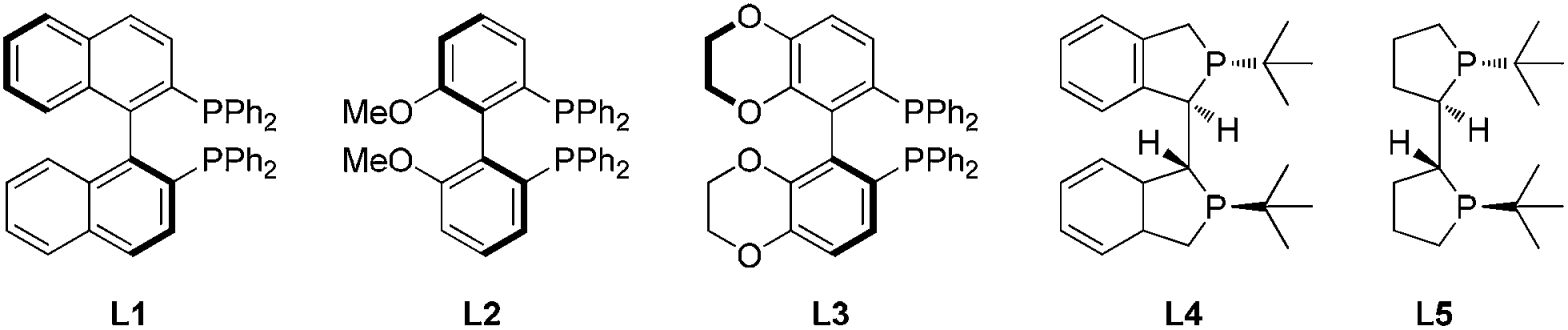				

^*a*^Reaction conditions: Pd(OCOCF_3_)_2_ (2 mol%), ligand (2.1 mol%), **1a** (0.3 mmol), additive (0.3 mmol), H_2_ (1200 psi), DCM (2 mL), 60 °C, 36 h.

^*b*^Isolated yields.

^*c*^Determined by HPLC.

^*d*^Using TFE as solvent.

^*e*^48 h.

Further examinations were focused on ligand screening. From the evaluation of the various commercially available chiral axial bisphosphine ligands, excellent enantioselectivity was obtained with ligand **L2** (entry 6), providing the product in 96% ee and 81% isolated yield. When the reaction time was prolonged to 48 hours, the yield was further improved without loss of enantioselectivity (entry 10). Therefore, the optimal condition was established as: Pd(OCOCF_3_)_2_/**L2**/TFA in dichloromethane.

With the optimal conditions in hand, exploration of the substrate scope was carried out, and the results are summarized in Table 2. Gratifyingly, a variety of 1-aryl substituted substrates were smoothly converted to the corresponding pyrazolidinones with excellent enantioselectivities (82–96% ee). The electronic properties of the substituents on the phenyl ring had little effect on the activity and enantioselectivity (entry 5 *vs.* entries 6–8). However, the hydrogenation of 2-*o*-tolyl-substituted pyrazol-5-ol **1b** gave a moderate 82% enantioselectivity and 67% yield (entry 2). When TangPhos **L5**, which was developed by Zhang's group in 2002,^28^ was employed and the temperature was elevated to 100 °C, the pyrazol-5-ol substrates (**1i–1j**) bearing a pentafluoroethyl substituent could also be hydrogenated with excellent enantioselectivities and yields (entries 9 and 10).

**Table 2:** Pd-catalyzed asymmetric hydrogenation of pyrazol-5-ols **1** ^*a*^

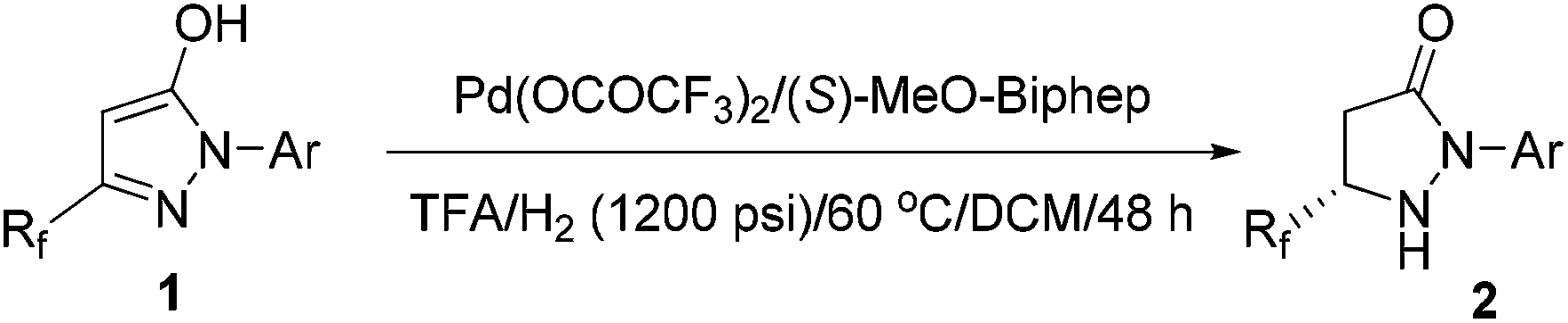				
Entry	*R*_F_	Ar	Yield (%)^*b*^	Ee (%)^*c*^
1	CF_3_	C_6_H_5_	94 (**2a**)	96 (*S*)
2^*d*^	CF_3_	2-MeC_6_H_4_	67 (**2b**)	82 (+)
3	CF_3_	3-MeC_6_H_4_	93 (**2c**)	95 (+)
4	CF_3_	4-MeC_6_H_4_	93 (**2d**)	96 (+)
5	CF_3_	4-MeOC_6_H_4_	94 (**2e**)	95 (+)
6	CF_3_	3-ClC_6_H_4_	89 (**2f**)	95 (+)
7	CF_3_	3,4-Cl_2_C_6_H_3_	90 (**2g**)	93 (+)
8	CF_3_	4-FC_6_H_4_	93 (**2h**)	94 (+)
9^*e*^	C_2_F_5_	C_6_H_5_	95 (**2i**)	94 (–)
10^*e*^	C_2_F_5_	4-MeC_6_H_4_	92 (**2j**)	95 (–)

^*a*^Pd(OCOCF_3_)_2_ (2 mol%), (*S*)-MeO-Biphep (2.1 mol%), **1** (0.3 mmol), H_2_ (1200 psi), TFA (0.3 mmol), DCM (2 mL), 60 °C, 48 h.

^*b*^Isolated yields.

^*c*^Determined by HPLC.

^*d*^Pd(OCOCF_3_)_2_ (4 mol%), (*S*)-MeO-Biphep (4.2 mol%), **1b**(0.2 mmol), H_2_ (1200 psi), TFA (0.2 mmol), DCM (2 mL), 60 °C, 48 h.

^*e*^Pd(OCOCF_3_)_2_ (4 mol%), (*S*,*S*′,*R*,*R*′)-TangPhos (5.2 mol%), 1 (0.2 mmol), H_2_ (1200 psi), l-CSA (0.2 mmol), TFE (2 mL), 100 °C, 48 h.

For the sake of further estimating the application possibility, a range of 4-substituted 3-(trifluoromethyl)-1*H*-pyrazol-5-ols (**3a–3g**) were also investigated (Table 3). The substrates with an alkyl-substituent at the 4-position could be hydrogenated smoothly, providing the corresponding the 2,4,5-trisubstituted pyrazolidinone derivatives with high enantioselectivity and diastereoselectivity. The high diastereoselectivity probably results from the thermodynamic stability of the *trans* products under these harsh acidic conditions. Substrates bearing long alkyl or bulky substituents at the C4 position gave slightly higher enantioselectivities (entry 1 *vs.* entries 3,6,7).

**Table 3:** Pd-catalyzed asymmetric hydrogenation of pyrazol-5-ols 3^*a*^

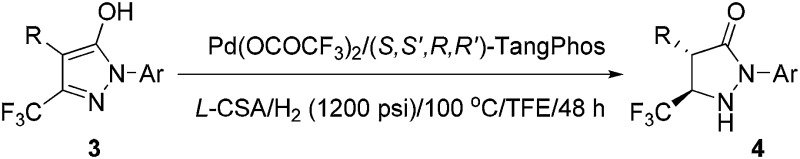				
Entry	R	Ar	Yield (%)^*b*^	Ee (%)^*c*^
1	Me	C_6_H_5_	92 (**4a**)	89 (–)
2	Me	4-MeC_6_H_4_	97 (**4b**)	88 (+)
3	Et	C_6_H_5_	93 (**4c**)	94 (+)
4	Et	4-MeC_6_H_4_	92 (**4d**)	93 (+)
5	Et	3-MeC_6_H_4_	90 (**4e**)	92 (+)
6	^*n*^Pr	C_6_H_5_	95 (**4f**)	93 (+)
7	Bn	C_6_H_5_	94 (**4g**)	95 (4*S*,5*R*)

^*a*^Pd(OCOCF_3_)_2_ (4 mol%), (*S*,*S*′,*R*,*R*′)-TangPhos (5.2 mol%), **3** (0.2 mmol), H_2_ (1200 psi), l-CSA (0.2 mmol), TFE (2 mL), 100 °C, 48 h. In all cases dr > 20 : 1.

^*b*^Isolated yields.

^*c*^Determined by HPLC.

The absolute configurations of the hydrogenation products **2a** and **4g** were determined by X-ray diffraction analysis by recrystallization from the solvent mixture dichloromethane–*n*-hexane.^29^ The configurations of the other chiral products were assigned by analogy.

In order to verify our hypothesis that the hydrogenation was carried out *via* capture of the active tautomer, we synthesized three compounds (form **A** type **5**, form **B** type **6** and form **C** type **8**) and subject them to identical hydrogenation reactions (Scheme 4). As expected, no reaction was observed for the substrate **5**; for substrate **6**, a low 10% ee and 14% yield were obtained; the CH–form substrate **8** gave an excellent 91% ee with 89% yield. Based on the experimental results and stereochemistry of the products, we proposed that the reaction experienced the process of Brønsted acid promoted tautomerization to form CH–form tautomer **C**, followed by Pd-catalyzed asymmetric hydrogenation of the active tautomer **C** to give the optically active pyrazolidinones. This preliminary result demonstrated the practicability of our strategy of asymmetric hydrogenation of the inseparable active isomers to realize hydrogenation of the intractable isomerisable substrates.

**Scheme 4:**
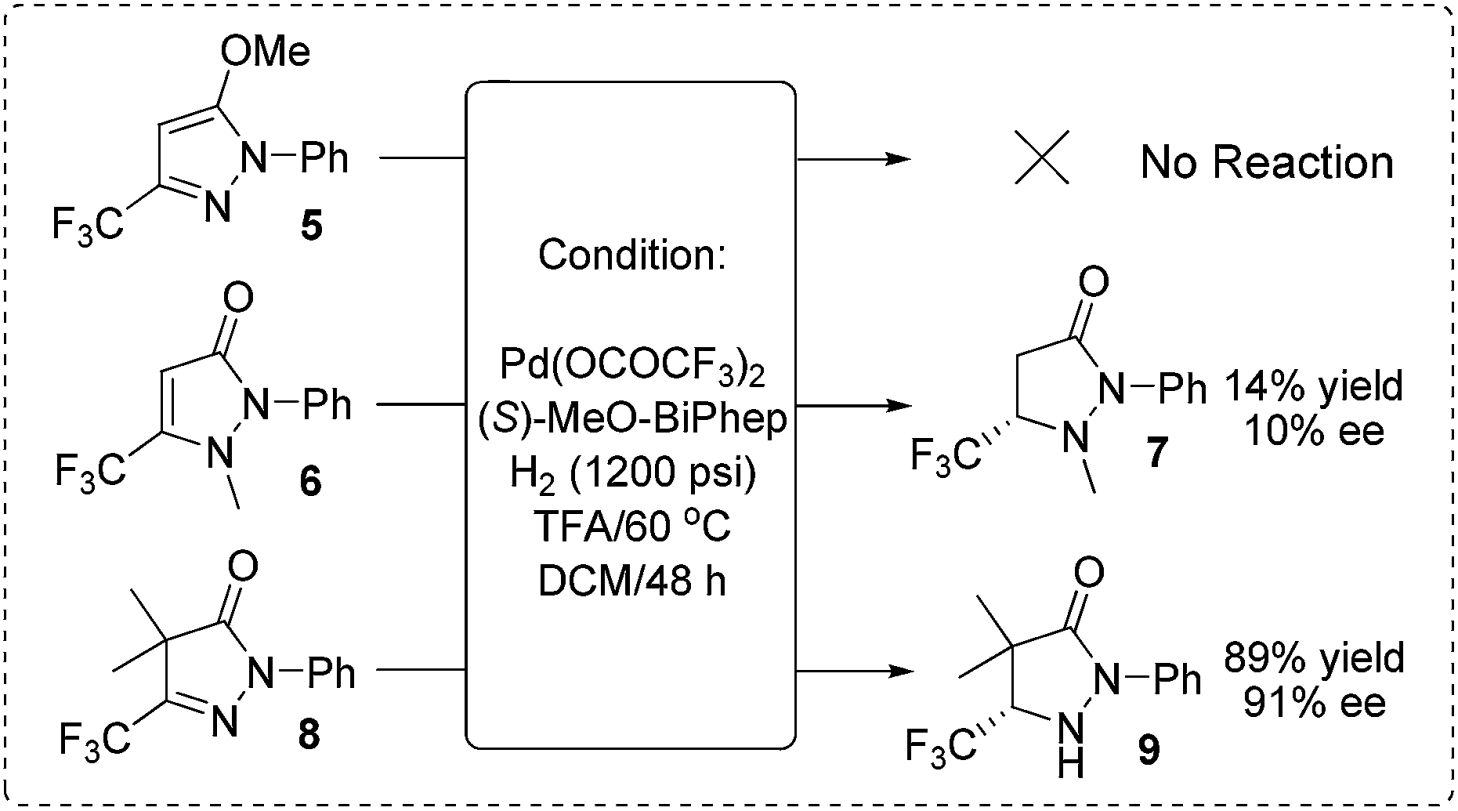
Reactions of the mechanistic investigation. All reactions were carried out under the conditions of Pd(OCOCF_3_)_2_ (4 mol%), (*S*)-MeO-Biphep (5.2 mol%), substrate (0.2 mmol), H_2_ (1200 psi), TFA (0.2 mmol), DCM (2 mL), 60 °C, 48 h.

## Conclusions

An efficient palladium-catalyzed asymmetric hydrogenation of fluorinated aromatic pyrazol-5-ols has been developed *via* capture of the active tautomers. A wide variety of 2,5-disubstituted and 2,4,5-trisubstituted pyrazolidinone derivatives have been synthesized with up to 96% and 95% ee, respectively. The hydrogenation pathway includes Brønsted acid promoted tautomerization of the pyrazol-5-ols and palladium-catalyzed asymmetric hydrogenation of the active tautomer. This study provides some enlightenment of the application of asymmetric hydrogenation and useful information for the design of new reactions. Further study on applying this novel strategy to other aromatic compounds and exploration of the applications of the chiral pyrazolidinones are in progress in our laboratory.
