# Supplementary material for: Pd-catalyzed asymmetric hydrogenation of fluorinated aromatic pyrazol-5-ols via capture of active tautomers
Source: Chem Sci. 2015 Mar 31;6(6):3415–9. doi: 10.1039/c5sc00835b (PMC5659069; doi:10.1039/c5sc00835b)
Supplement: Supplementary file 1 [file SC-006-C5SC00835B-s001.pdf]

Electronic Supplementary Material (ESI) for Chemical Science.
This journal is © The Royal Society of Chemistry 2015

Supporting Information

Pd-catalyzed asymmetric hydrogenation of fluorinated aromatic pyrazol-5-ols *via* capture of active tautomers

*Zhang-Pei Chen, Mu-Wang Chen, Lei Shi, Chang-Bin Yu, and Yong-Gui Zhou**

State Key Laboratory of Catalysis, Dalian Institute of Chemical Physics, Chinese Academy of Sciences, Dalian 116023, China. Collaborative Innovation Centre of Chemical Science and Engineering (Tianjin), Tianjin 300071, P. R. China

Table of Contents

1. **General and Materials…………………………………...…..….....................S1**
2. **General Procedure for Synthesis of Fluorinated Pyrazol-5-ols...................S1-4**
3. **General Procedure for Hydrogenation of Disubstituted Fluorinated Pyrazol-5-ols 1a-1h...S4-6**
4. **General Procedure for Hydrogenation of Disubstituted Fluorinated Pyrazol-5-ols 1i, 1j and Trisubstituted Fluorinated Pyrazol-5-ols 3……....S6-9**
5. **Mechanistic Investigation...…..S9-11**
6. **The X-ray Crystallographic Analysis of Substrate 3a and Products.......S11-12**
7. **References...S12**
8. **Copy of NMR of Substrates and Products..S13-120**
9. **Copy of HPLC for Racemic and Chiral Products................................S121-138**

1. General and Materials

General: Commercially available reagents were used without further purification. Solvents were treated prior to use according to the standard methods. ^{1}H NMR, ^{13}C NMR and ^{19}F NMR spectra were recorded at room temperature in $CDCl_3$ on 400 MHz instrument with tetramethylsilane (TMS) as internal standard. Enantiomeric excess was determined by HPLC analysis, using chiral column described below in detail. Optical rotations were measured by polarimeter. Flash column chromatography was performed on silica gel (200-300 mesh).

2. General Procedure for Synthesis of Fluorinated Pyrazol-5-ols

All fluorinated pyrazol-5-ols were prepared from the accessible starting materials appropriate fluorinated β-ketoesters and arylhydrazines according to the literature methods.[1] Pyrazol-5-ol **1a** is known and its NMR data matched the literature data.

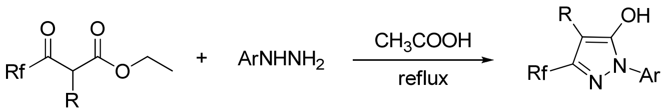

Fluorinated β-ketoesters (5.0 mmol) and arylhydrazines (5.0 mmol) were mixed and refluxed in acetic acid (30 mL) for 6 h. Then the mixture was cooled to room temperature. After filtration, the residue was recrystallized from ethanol to give the pure fluorinated pyrazol-5-ols.

1-(*o*-Tolyl)-3-(trifluoromethyl)-1*H*-pyrazol-5-ol (1b): Pale yellow solid; mp = 204-205 °C, yield 58%; ^{1}H NMR (400 MHz, DMSO-d_6) δ 12.03 (br, 1H), 7.45–7.37 (m, 2H), 7.37–7.29 (m, 2H),

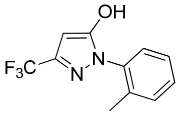

5.91 (s, 1H), 2.07 (s, 3H); ^{13}C NMR (100 MHz, DMSO-d_6) δ 154.4, 140.7 (q, J_{C-F} = 37 Hz), 136.7, 135.6, 131.2, 129.8, 128.1, 127.0, 121.9 (q, J_{C-F} = 267 Hz), 84.7, 17.5; ^{19}F NMR (377 MHz, DMSO-d_6) δ -61.6 (s, 3F); IR (film): 2808, 2714, 1558, 1504, 1422, 1259, 1218, 1198, 1157, 1136; HRMS Calculated For $C_{11}H_{10}F_3N_2O$ $[M+H]^+$ 243.0745, found: 243.0740.

1-(*m*-Tolyl)-3-(trifluoromethyl)-1*H*-pyrazol-5-ol (1c): Pale yellow solid; mp = 210-211 °C, yield 62%; ^{1}H NMR (400 MHz, DMSO-d_6) δ 12.42 (br, 1H), 7.57-7.52 (m, 2H), 7.36 (t, J = 7.7 Hz,

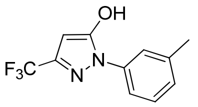

1H), 7.16 (d, J = 7.4 Hz, 1H), 5.97 (s, 1H), 2.36 (s, 3H); ^{13}C NMR (100 MHz, DMSO-d_6) δ 154.1, 140.8 (q, J_{C-F} = 37 Hz), 139.1, 138.2, 129.2, 128.2, 123.2, 121.4 (q, J_{C-F} = 266 Hz), 119.8, 86.0, 21.3; ^{19}F NMR (377 MHz, DMSO-d_6) δ -62.0 (s, 3F); IR (film): 2826, 1612, 1565, 1490, 1408, 1333, 1245, 1150, 1116; HRMS Calculated For $C_{11}H_{10}F_3N_2O$ $[M+H]^+$ 243.0745, found: 243.0740.

1-(*p*-Tolyl)-3-(trifluoromethyl)-1*H*-pyrazol-5-ol (1d): Pale yellow solid; mp = 226-227 °C, yield 63%; ^{1}H NMR (400 MHz, DMSO-d_6) δ 12.38 (br, 1H), 7.59 (d, J = 8.4 Hz, 2H), 7.30 (d, J = 8.3

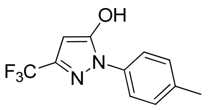

Hz, 2H), 5.93 (s, 1H), 2.34 (s, 3H); ^{13}C NMR (100 MHz, DMSO-d_6) δ 154.0, 140.6 (q, J_{C-F} = 37 Hz), 137.1, 135.8, 129.9, 122.7, 122.0 (q, J_{C-F} = 267 Hz), 86.0, 21.0; ^{19}F NMR (377 MHz, DMSO-d_6) δ -61.8 (s, 3F); IR (film): 2883, 1572, 1490, 1415, 1347, 1259, 1415, 1347, 1259, 1218, 1157, 1110; HRMS Calculated For $C_{11}H_{10}F_3N_2O$ $[M+H]^+$ 243.0745, found: 243.0740.

1-(4-Methoxyphenyl)-3-(trifluoromethyl)-1*H*-pyrazol-5-ol (1e): Pale yellow solid; mp = 209-210 °C, yield 53%; ^{1}H NMR (400 MHz, DMSO-d_6) δ 12.29 (br, 1H), 7.58 (d, J = 9.0 Hz, 2H), 7.07–7.04 (m, 2H), 5.91 (s, 1H), 3.80 (s, 3H); ^{13}C NMR (100 MHz, DMSO-d_6) δ 158.7, 153.8, 140.2 (q, $J_{C\text{-}F}$ = 37 Hz), 131.2, 124.6, 122.4 (q, $J_{C\text{-}F}$ = 267 Hz), 114.6, 85.8, 55.9; ^{19}F NMR (377 MHz, DMSO-d_6) δ -61.7 (s, 3F); IR (film): 2937, 2822, 1613, 1591, 1562, 1499, 1416, 1354, 1306, 1254, 1130, 1096, 1025; HRMS Calculated For $C_{11}H_{10}F_3N_2O_2$ $[M+H]^+$ 259.0694, found 259.0689.

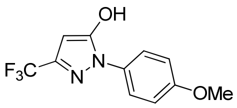

1-(3-Chlorophenyl)-3-(trifluoromethyl)-1*H*-pyrazol-5-ol (1f): Pale yellow solid; mp = 220-221 °C, yield 57%; ^{1}H NMR (400 MHz, DMSO-d_6) δ 12.80 (br, 1H), 7.81 (d, J = 1.7 Hz, 1H), 7.75 (d, J = 8.7 Hz, 1H), 7.52 (t, J = 8.1 Hz, 1H), 7.41 (d, J = 8.1 Hz, 1H), 5.96 (s, 1H); ^{13}C NMR (100 MHz, DMSO-d_6) δ 154.6, 141.5 (q, $J_{C\text{-}F}$ = 37 Hz), 139.4, 133.9, 131.2, 127.3, 121.9, 121.5 (q, $J_{C\text{-}F}$ = 267 Hz), 120.7, 86.3; ^{19}F NMR (377 MHz, DMSO-d_6) δ -62.2 (s, 3F); IR (film): 3169, 2802, 1585, 1559, 1505, 1473, 1429, 1401, 1330, 1256, 1139, 1105, 1044; HRMS Calculated For $C_{10}H_7ClF_3N_2O$ $[M+H]^+$ 263.0199, found: 263.0194.

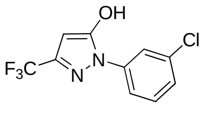

1-(3,4-Dichlorophenyl)-3-(trifluoromethyl)-1*H*-pyrazol-5-ol (1g): Pale yellow solid; mp = 213-214 °C, yield 57%; ^{1}H NMR (400 MHz, DMSO-d_6) δ 12.90 (br, 1H), 7.98 (d, J = 2.2 Hz, 1H), 7.79-7.75 (m, 1H), 7.74–7.68 (m, 1H), 5.94 (s, 1H); ^{13}C NMR (100 MHz, DMSO-d_6) δ 154.8, 141.7 (q, $J_{C\text{-}F}$ = 37 Hz), 137.9, 132.0, 131.5, 129.7, 123.4, 121.9, 121.9 (q, $J_{C\text{-}F}$ = 267 Hz), 86.3; ^{19}F NMR (377 MHz, DMSO-d_6) δ -62.3 (s, 3F); IR (film): 3159, 3090, 2914, 1594, 1573, 1557, 1502, 1472, 1385, 1261, 1247, 1202, 1160; HRMS Calculated For $C_{10}H_6Cl_2F_3N_2O$ $[M+H]^+$ 296.9809, found: 296.9804.

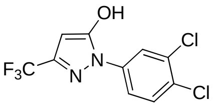

1-(4-Fluorophenyl)-3-(trifluoromethyl)-1*H*-pyrazol-5-ol (1h) Pale yellow solid; mp = 216-217 °C, yield 60%; ^{1}H NMR (400 MHz, DMSO-d_6) δ 12.54 (br, 1H), 7.77–7.70 (m, 2H), 7.35–7.29 (m, 2H), 5.94 (s, 1H); ^{13}C NMR (100 MHz, DMSO-d_6) δ 161.2 (d, $J_{C\text{-}F}$ = 245 Hz), 154.1, 140.9 (q, $J_{C\text{-}F}$ = 37 Hz), 134.5 (d, $J_{C\text{-}F}$ = 2.8 Hz), 125.0 (d, $J_{C\text{-}F}$ = 8.7 Hz), 122.8 (q, $J_{C\text{-}F}$ = 267 Hz), 116.4 (d, $J_{C\text{-}F}$ = 22.9 Hz), 86.0; ^{19}F NMR (377 MHz, DMSO-d_6) δ -62.0 (s, 3F), -114.8 (s, 1F); IR (film): 3084, 1610, 1571, 1499, 1405, 1331, 1217, 1191, 1135, 1096; HRMS Calculated For $C_{10}H_7F_4N_2O$ $[M+H]^+$ 247.0495, found: 247.0489.

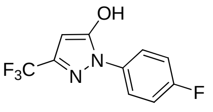

3-(Perfluoroethyl)-1-phenyl-1*H*-pyrazol-5-ol (1i): Pale yellow solid; mp = 190-191 °C, yield 69%; ^{1}H NMR (400 MHz, DMSO-d_6) δ 12.51 (br, 1H), 7.75 (d, J = 7.8 Hz, 2H), 7.50 (t, J = 7.7 Hz, 2H), 7.36 (t, J = 7.3 Hz, 1H), 5.97 (s, 1H); ^{13}C NMR (100 MHz, DMSO-d_6) δ 154.4, 139.6 (t, $J_{C\text{-}F}$ = 28 Hz), 138.2, 129.5, 127.6, 122.6, 118.7 (qt, $J_{C\text{-}F}$ = 284 Hz, 39 Hz,), 111.2 (tq, $J_{C\text{-}F}$ = 248 Hz, 38 Hz,), 87.1; ^{19}F NMR (377 MHz, DMSO-d_6) δ -83.8 (s, 3F), -112.7 (s, 2F); IR (film): 2917, 1598, 1569, 1409, 1334, 1185, 1125, 1103; HRMS Calculated For $C_{11}H_8F_5N_2O$ $[M+H]^+$ 279.0557, found: 279.0551.

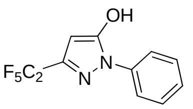

3-(Perfluoroethyl)-1-(*p*-tolyl)-1*H*-pyrazol-5-ol (1j): Pale yellow solid; mp = 209-210 °C, yield 60%; ^{1}H NMR (400 MHz, DMSO-d_6) δ 12.41 (br, 1H), 7.59 (d, J = 8.2 Hz, 2H), 7.30 (d, J = 8.2 Hz, 2H), 5.93 (s, 1H), 2.34 (s, 3H); ^{13}C NMR (100 MHz, DMSO-d_6) δ 154.2, 139.2 (t, $J_{C\text{-}F}$ = 28 Hz), 137.2, 135.8, 129.9, 122.6, 119.4 (qt, $J_{C\text{-}F}$ = 286 Hz, 38 Hz,), 111.2 (tq, $J_{C\text{-}F}$ = 249 Hz, 38 Hz,), 87.0, 20.9; ^{19}F NMR (377 MHz, DMSO-d_6) δ -83.6 (s, 3F), -112.4 (s, 2F); IR (film): 3115, 2955, 1591, 1565, 1523, 1486, 1408,

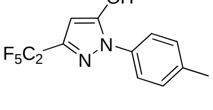

1336, 1227, 1192, 1138, 1112, 1044; HRMS Calculated For $C_{12}H_{10}F_5N_2O$ $[M+H]^+$ 293.0713, found: 293.0708.

4-Methyl-1-phenyl-3-(trifluoromethyl)-1*H*-pyrazol-5-ol (3a): Pale yellow solid; mp = 148-149 °C, yield 58%; ^{1}H NMR (400 MHz, DMSO-d_6) δ 11.51 (br, 1H), 7.69 (d, J = 7.3 Hz, 2H), 7.51 (t, J = 7.8 Hz, 2H), 7.38 (t, J = 7.3 Hz, 1H), 2.03 (s, 3H); ^{13}C NMR (100 MHz, DMSO-d_6) δ 151.2, 139.4 (q, J_{C-F} = 36 Hz), 138.4, 129.6, 127.6, 122.9, 122.5 (q, J_{C-F} = 268 Hz), 96.1, 6.7; ^{19}F NMR (377 MHz, DMSO-d_6) δ -61.4 (s, 3F); IR (film): 2924, 1597, 1579, 1543, 1487, 1455, 1394, 1263, 1151; HRMS Calculated For $C_{11}H_{10}F_3N_2O$ $[M+H]^+$ 243.0745, found: 243.0740.

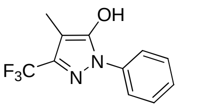

4-Methyl-1-*p*-tolyl-3-(trifluoromethyl)-1*H*-pyrazol-5-ol (3b): Pale yellow solid; mp = 170-171 °C, yield 68%; ^{1}H NMR (400 MHz, DMSO-d_6) δ 11.44 (br, 1H), 7.55 (d, J = 8.1 Hz, 2H), 7.30 (d, J = 7.9 Hz, 2H), 2.35 (s, 3H), 2.02 (s, 3H); ^{13}C NMR (100 MHz, DMSO-d_6) δ 151.0, 139.0 (q, J_{C-F} = 36 Hz), 137.1, 136.0, 129.9, 122.9, 122.5 (q, J_{C-F} = 267 Hz), 96.0, 21.0, 6.7; ^{19}F NMR (377 MHz, DMSO-d_6) δ -61.3 (s, 3F); IR (film): 2922, 1595, 1396, 1280, 1164, 1120, 1052; HRMS Calculated For $C_{12}H_{12}F_3N_2O$ $[M+H]^+$ 257.0902, found: 257.0896.

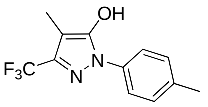

4-Ethyl-1-phenyl-3-(trifluoromethyl)-1*H*-pyrazol-5-ol (3c): Pale yellow solid; mp = 140-141 °C, yield 48%; ^{1}H NMR (400 MHz, DMSO-d_6) δ 11.46 (br, 1H), 7.71–7.68 (m, 2H), 7.53–7.49 (m, 2H), 7.40–7.36 (m, 1H), 2.50–2.48 (m, 2H), 1.10 (t, J = 7.4 Hz, 3H); ^{13}C NMR (100 MHz, DMSO-d_6) δ 150.8, 138.7 (q, J_{C-F} = 35 Hz), 138.3, 129.5, 127.7, 123.0, 122.6 (q, J_{C-F} = 268 Hz), 103.1, 15.7, 15.0; ^{19}F NMR (377 MHz, DMSO-d_6) δ -60.9 (s, 3F); IR (film): 2978, 2872, 1594, 1565, 1477, 1393, 1312, 1266, 1152, 1073; HRMS Calculated For $C_{12}H_{12}F_3N_2O$ $[M+H]^+$ 257.0902, found: 257.0896.

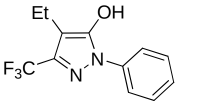

4-Ethyl-1-*p*-tolyl-3-(trifluoromethyl)-1*H*-pyrazol-5-ol (3d): Pale yellow solid; mp = 153-154 °C, yield 68%; ^{1}H NMR (400 MHz, DMSO-d_6) δ 11.34 (br, 1H), 7.59 (d, J = 8.3 Hz, 2H), 7.29 (d, J = 8.3 Hz, 2H), 2.55-2.49 (m, 2H), 2.34 (s, 3H), 1.12 (t, J = 7.5 Hz, 3H); ^{13}C NMR (100 MHz, DMSO-d_6) δ 150.7, 138.6 (q, J_{C-F} = 35 Hz), 137.0, 136.1, 129.8, 122.8, 122.6 (q, J_{C-F} = 267 Hz), 103.1, 20.9, 15.6, 15.0; ^{19}F NMR (377 MHz, DMSO-d_6) δ -61.0 (s, 3F); IR (film): 2978, 2937, 1593, 1527, 1521, 14901, 1396, 1314, 1287, 1159, 1075; HRMS Calculated For $C_{13}H_{14}F_3N_2O$ $[M+H]^+$ 271.1058, found: 271.1053.

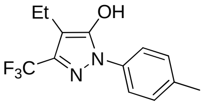

4-Ethyl-1-*m*-tolyl-3-(trifluoromethyl)-1*H*-pyrazol-5-ol (3e): Pale yellow solid; mp = 118-119 °C, yield 63%; ^{1}H NMR (400 MHz, DMSO-d_6) δ 11.37 (br, 1H), 7.53 (d, J = 7.6 Hz, 2H), 7.37 (t, J = 7.4 Hz, 1H), 7.17 (d, J = 7.0 Hz, 1H), 2.53 (q, J = 7.2 Hz, 2H), 2.37 (s, 3H), 1.13 (t, J = 7.1 Hz, 3H); ^{13}C NMR (100 MHz, DMSO-d_6) δ 150.8, 139.1, 138.6 (t, J_{C-F} = 28 Hz), 138.3, 129.3, 128.3, 123.5, 122.3 (q, J_{C-F} = 268 Hz), 120.1, 103.1, 21.4, 15.7, 15.0; ^{19}F NMR (377 MHz, DMSO-d_6) δ -61.0 (s, 3F); IR (film): 2966, 2917, 1573, 1483, 1390, 1286, 1162, 1112, 1076; HRMS Calculated For $C_{13}H_{14}F_3N_2O$ $[M+H]^+$ 271.1058, found: 271.1053.

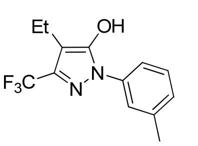

1-Phenyl-4-propyl-3-(trifluoromethyl)-1*H*-pyrazol-5-ol (3f): Pale yellow solid; mp = 123-124 °C, yield 48%; ^{1}H NMR (400 MHz, DMSO-d_6) δ 11.42 (br, 1H), 7.70 (d, *J* = 8.2 Hz, 2H), 7.51 (t, *J* = 7.8 Hz, 2H), 7.38 (t, *J* = 7.4 Hz, 1H), 2.44 (t, *J* = 7.6 Hz, 2H), 1.53–1.49 (m, 2H), 0.91 (t, *J* = 7.3 Hz, 3H); ^{13}C NMR (100 MHz, DMSO-d_6) δ 151.1, 139.0 (q, J_{C-F} = 35 Hz), 138.4, 129.5, 127.6, 122.9, 122.6 (q, J_{C-F} = 267 Hz), 101.5, 23.8, 23.6, 14.1; ^{19}F NMR (377 MHz, DMSO-d_6) δ -60.7 (s, 3F); IR (film): 3243, 2856, 2414, 1593, 1570, 1481, 1456, 1400, 1270, 1137, 1084; HRMS Calculated For $C_{13}H_{14}F_3N_2O$ $[M+H]^+$ 271.1058, found: 271.1053.

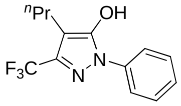

4-Benzyl-1-phenyl-3-(trifluoromethyl)-1*H*-pyrazol-5-ol (3g): Pale yellow solid; mp = 147-148 °C, yield 23%; ^{1}H NMR (400 MHz, DMSO-d_6) δ 11.78 (br, 1H), 7.76–7.72 (m, 2H), 7.55–7.51 (m, 2H), 7.42–7.38 (m, 1H), 7.31–7.27 (m, 2H), 7.20–7.16 (m, 3H), 3.89 (s, 2H); ^{13}C NMR (100 MHz, DMSO-d_6) δ 151.7, 140.6, 139.0 (q, J_{C-F} = 36 Hz), 138.3, 129.6, 128.7, 128.2, 127.8, 126.4, 123.0, 122.4 (q, J_{C-F} = 268 Hz), 99.9, 27.1; ^{19}F NMR (377 MHz, DMSO-d_6) δ -60.7 (s, 3F); IR (film): 2917, 1595, 1577, 1536, 1457, 1390, 1275, 1132, 1028; HRMS Calculated For $C_{17}H_{14}F_3N_2O$ $[M+H]^+$ 319.1058, found: 319.1053.

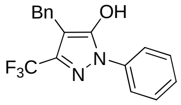

3. General Procedure for Hydrogenation of Disubstituted Fluorinated Pyrazol-5-ols 1a-1h

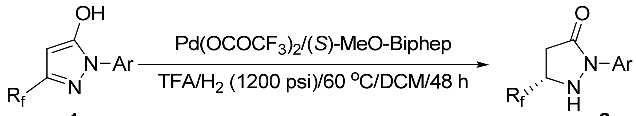

1 **2**

General procedure: (*S*)-MeO-Biphep (3.8 mg, 0.0066 mmol) and $Pd(OCOCF_3)_2$ (2.0 mg, 0.006 mmol) were placed in a dried Schlenk tube under nitrogen atmosphere, and degassed anhydrous acetone was added. The mixture was stirred at room temperature for 1 h, and then solvent was removed under vacuum to give the catalyst. In a glovebox, pyrazol-5-ol **1** (0.30 mmol) and the above catalyst together with dichloromethane (2 mL) were stirred at room temperature for 1 min. Subsequently, trifluoroacetic acid (TFA, 34.2 mg, 22.3 μL, 0.3 mmol) was added to the reaction mixture. The hydrogenation was performed at 60 ^{o}C under H_2 (1200 psi) in a stainless steel autoclave for 48 h. After carefully releasing the hydrogen, the resulting mixture was concentrated under vacuum and dissolved in saturated aqueous sodium bicarbonate (5 mL). After stirring for 10 min, the mixture was extracted with dichloromethane and dried over sodium sulfate. After purification by silica gel chromatography using petroleum ether/dichloromethane (1:1) as eluent, the enantiomeric excess of the products were determined by HPLC with chiral column.

(*S*)-2-Phenyl-5-(trifluoromethyl)pyrazolidin-3-one (2a): Pale yellow solid; mp = 104-105 °C, yield 94%, 96% ee, $[\alpha]^{20}_D$ = +20.0 (*c* 0.50, $CHCl_3$); ^{1}H NMR (400 MHz, $CDCl_3$) δ 7.77 (d, *J* = 8.1 Hz, 2H), 7.36 (t, *J* = 7.9 Hz, 2H), 7.16 (t, *J* = 7.4 Hz, 1H), 5.15 (d, *J* = 7.3 Hz, 1H), 4.13–4.02 (m, 1H), 3.18 (dd, *J* = 17.5, 9.7 Hz, 1H), 2.84 (dd, *J* = 17.6, 3.1 Hz, 1H); ^{13}C NMR (100 MHz, $CDCl_3$) δ 167.5, 137.8, 128.9, 125.4 (q, J_{C-F} = 278 Hz), 125.2, 118.9, 53.9 (q, J_{C-F} = 32 Hz), 33.7; ^{19}F NMR (377 MHz, $CDCl_3$) δ -78.4 (s, 3F); IR (film): 3257, 2919, 1597, 1494, 1464, 1316, 1188; HRMS Calculated For $C_{10}H_{10}F_3N_2O$ $[M+H]^+$ 231.0745, found: 231.0740; HPLC (AD-H, elute: Hexanes/*i*-PrOH = 70/30, detector: 254 nm, flow rate: 0.7 mL/min), t_1 = 6.2 min (maj), t_2 = 7.5 min.

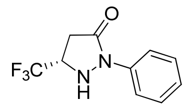

(+)-2-*o*-Tolyl-5-(trifluoromethyl)pyrazolidin-3-one (2b): Pale yellow solid; mp = 104-105 °C, yield 67%, 82% ee, $[\alpha]^{20}_D$ = +18.4 (*c* 0.50, $CHCl_3$); ^1H NMR (400 MHz, $CDCl_3$) δ 7.34–7.25 (m, 4H), 5.17 (d, *J* = 7.4 Hz, 1H), 4.15 (d, *J* = 7.1 Hz, 1H), 3.23–3.16 (m, 1H), 2.89–2.83 (m, 1H), 2.30 (s, 3H); ^{13}C NMR (100 MHz, $CDCl_3$) δ 167.3, 135.4, 135.3, 131.3, 128.7, 126.8, 126.4, 125.8 (q, J_{C-F} = 277 Hz), 54.4 (q, J_{C-F} = 32 Hz), 32.3, 18.2; ^{19}F NMR (377 MHz, $CDCl_3$) δ -78.0 (s, 3F); IR (film): 2940, 1698, 1534, 1471, 1350, 1234, 1165; HRMS Calculated For $C_{11}H_{12}F_3N_2O$ $[M+H]^+$ 245.0902, found: 245.0896; HPLC (AD-H, elute: Hexanes/*i*-PrOH = 80/20, detector: 254 nm, flow rate: 0.7 mL/min), t_1 = 8.0 min (maj), t_2 = 8.8 min.

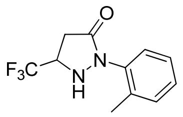

(+)-2-*m*-Tolyl-5-(trifluoromethyl)pyrazolidin-3-one (2c): Pale yellow solid; mp = 89-90 °C, yield 93%, 95% ee, $[\alpha]^{20}_D$ = +14.4 (*c* 0.50, $CHCl_3$); ^1H NMR (400 MHz, $CDCl_3$) δ 7.57-7.50 (m, 2H), 7.26–7.20 (m, 1H), 6.97 (d, *J* = 7.7 Hz, 1H), 5.14 (br, 1H), 4.11–4.01 (m, 1H), 3.15 (dd, *J* = 17.5, 9.7 Hz, 1H), 2.82 (dd, *J* = 17.5, 3.1 Hz, 1H), 2.35 (s, 3H); ^{13}C NMR (100 MHz, $CDCl_3$) δ 167.5, 138.9, 137.7, 128.7, 126.1, 125.5 (q, J_{C-F} = 278 Hz), 119.5, 116.2, 53.8 (q, J_{C-F} = 33 Hz), 33.8, 21.5; ^{19}F NMR (377 MHz, $CDCl_3$) δ -78.4 (s, 3F); IR (film): 2920, 1707, 1604, 1491, 1353, 1278, 1170, 1126; HRMS Calculated For $C_{11}H_{12}F_3N_2O$ $[M+H]^+$ 245.0902, found: 245.0896; HPLC (AD-H, elute: Hexanes/*i*-PrOH = 70/30, detector: 254 nm, flow rate: 0.7 mL/min), t_1 = 6.3 min (maj), t_2 = 8.4 min.

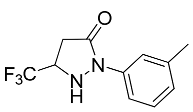

(+)-2-*p*-Tolyl-5-(trifluoromethyl)pyrazolidin-3-one (2d): Pale yellow solid; mp = 140-141 °C, yield 93%, 96% ee, $[\alpha]^{20}_D$ = +15.0 (*c* 0.50, $CHCl_3$); ^1H NMR (400 MHz, $CDCl_3$) δ 7.61 (d, *J* = 8.6 Hz, 2H), 7.14 (d, *J* = 8.4 Hz, 2H), 5.18 (d, *J* = 6.6 Hz, 1H), 4.04 (d, *J* = 6.7 Hz, 1H), 3.13 (dd, *J* = 17.5, 9.7 Hz, 1H), 2.79 (dd, *J* = 17.5, 3.1 Hz, 1H), 2.31 (s, 3H); ^{13}C NMR (100 MHz, $CDCl_3$) δ 167.3, 135.3, 135.1, 129.4, 124.6 (q, J_{C-F} = 278 Hz), 119.1, 53.9 (q, J_{C-F} = 33 Hz), 33.7, 20.9; ^{19}F NMR (377 MHz, $CDCl_3$) δ -78.5 (s, 3F); IR (film): 3156, 2937, 1707, 1510, 1138, 1170, 1123, 1041; HRMS Calculated For $C_{11}H_{12}F_3N_2O$ $[M+H]^+$ 245.0902, found: 245.0896; HPLC (AD-H, elute: Hexanes/*i*-PrOH = 70/30, detector: 254 nm, flow rate: 0.7 mL/min), t_1 = 6.7 min (maj), t_2 = 8.4 min.

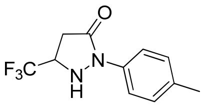

(+)-2-(4-Methoxyphenyl)-5-(trifluoromethyl)pyrazolidin-3-one (2e): Pale yellow solid; mp = 122-123 °C, yield 94%, 95% ee, $[\alpha]^{20}_D$ = +15.6 (c 0.50, $CHCl_3$); ^1H NMR (400 MHz, $CDCl_3$) δ 7.66 (d, *J* = 9.1 Hz, 2H), 6.90 (d, *J* = 9.1 Hz, 2H), 5.11 (d, *J* = 7.6 Hz, 1H), 4.13–4.07 (m, 1H), 3.80 (s, 3H), 3.19 (dd, *J* = 17.5, 9.7 Hz, 1H), 2.85 (dd, *J* = 17.5, 3.1 Hz, 1H); ^{13}C NMR (100 MHz, $CDCl_3$) δ 167.0, 157.2, 131.0, 123.9 (q, J_{C-F} = 278 Hz), 121.1, 114.1, 55.5, 53.9 (q, J_{C-F} = 33 Hz), 33.5; ^{19}F NMR (377 MHz, $CDCl_3$) δ -78.4 (s, 3F); IR (film): 2931, 1796, 1551, 1449, 1256, 1211; HRMS Calculated For $C_{11}H_{12}F_3N_2O_2$ $[M+H]^+$ 261.0851, found: 261.0845; HPLC (AD-H, elute: Hexanes/*i*-PrOH = 70/30, detector: 254 nm, flow rate: 0.7 mL/min), t_1 = 7.7 min (maj), t_2 = 8.8 min.

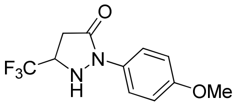

(+)-2-(3-Chlorophenyl)-5-(trifluoromethyl)pyrazolidin-3-one (2f): Pale yellow solid; mp = 115-116 °C, yield 89%, 95% ee, $[\alpha]^{20}_D$ = +16.6 (*c* 0.50, $CHCl_3$); ^1H NMR (400 MHz, $CDCl_3$) δ 7.83 (s, 1H), 7.73 (d, *J* = 8.4 Hz, 1H), 7.31-7.26 (m, 1H), 7.13 (d, *J* = 8.0 Hz, 1H), 5.16 (d, *J* = 7.4 Hz, 1H), 4.16–4.10 (m, 1H), 3.22 (dd, *J* = 17.5, 9.7 Hz, 1H), 2.87 (dd, *J* = 17.5, 3.1 Hz, 1H); ^{13}C NMR (100 MHz, $CDCl_3$) δ 167.9, 138.9, 134.7, 129.9, 125.1, 124.5 (q, J_{C-F} = 278 Hz), 118.7, 116.6, 54.0 (q, J_{C-F} = 33 Hz), 33.8; ^{19}F NMR (377 MHz, $CDCl_3$) δ -78.4 (s, 3F); IR (film): 3227, 2925, 1726, 1592, 1547, 1482, 1254, 1171, 1125;

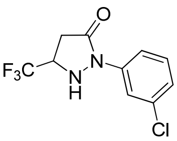

HRMS Calculated For $C_{10}H_9ClF_3N_2O$ $[M+H]^+$ 265.0356, found: 265.0350; HPLC (AD-H, elute: Hexanes/*i*-PrOH = 70/30, detector: 254 nm, flow rate: 0.7 mL/min), t_1 = 6.0 min (maj), t_2 = 7.1 min.

(+)-2-(3,4-Dichlorophenyl)-5-(trifluoromethyl)pyrazolidin-3-one (2g): Pale yellow solid; mp = 122-123 °C, yield 90%, 93% ee, $[\alpha]^{20}_D$ = +5.8 (*c* 0.50, $CHCl_3$); ^1H NMR (400 MHz, $CDCl_3$) δ

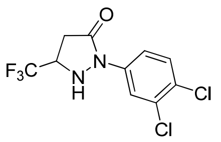

7.93 (d, *J* = 2.5 Hz, 1H), 7.70–7.67 (m, 1H), 7.39 (d, *J* = 8.9 Hz, 1H), 5.20 (d, *J* = 7.3 Hz, 1H), 4.17–4.10 (m, 1H), 3.21 (dd, *J* = 17.7, 9.6 Hz, 1H), 2.85 (dd, *J* = 17.7, 2.8 Hz, 1H); ^{13}C NMR (100 MHz, $CDCl_3$) δ 168.0, 137.2, 132.8, 130.5, 128.3, 124.3 (q, $J_{C\text{-}F}$ = 278 Hz), 120.1, 117.7, 54.0 (q, $J_{C\text{-}F}$ = 33 Hz), 33.7; ^{19}F NMR (377 MHz, $CDCl_3$) δ -78.4 (s, 3F); IR (film): 3235, 2960, 1715, 1556, 1503, 1355, 1259, 1170, 1106; HRMS Calculated For $C_{10}H_8Cl_2F_3N_2O$ $[M+H]^+$ 298.9966, found: 298.9960; HPLC (AD-H, elute: Hexanes/*i*-PrOH = 85/15, detector: 254 nm, flow rate: 0.7 mL/min), t_1 = 9.1 min (maj), t_2 = 10.1 min.

(+)-2-(4-Fluorophenyl)-5-(trifluoromethyl)pyrazolidin-3-one (2h): Pale yellow solid; mp = 111-112 °C, yield 93%, 94% ee, $[\alpha]^{20}_D$ = +21.4 (*c* 0.50, $CHCl_3$); ^1H NMR (400 MHz, $CDCl_3$) δ

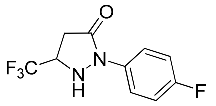

7.77–7.73 (m, 2H), 7.07–7.02 (m, 2H), 5.16 (d, *J* = 7.4 Hz, 1H), 4.15–4.08 (m, 1H), 3.21 (dd, *J* = 17.6, 9.7 Hz, 1H), 2.85 (dd, *J* = 17.6, 2.8 Hz, 1H); ^{13}C NMR (100 MHz, $CDCl_3$) δ 167.4, 159.9 (d, $J_{C\text{-}F}$ = 245 Hz), 133.9, 123.6 (q, $J_{C\text{-}F}$ = 275 Hz), 120.8 (d, $J_{C\text{-}F}$ = 8.0 Hz), 115.6 (d, $J_{C\text{-}F}$ = 23 Hz), 53.9 (q, $J_{C\text{-}F}$ = 32 Hz), 33.6; ^{19}F NMR (377 MHz, $CDCl_3$) δ -78.5 (s, 3F), 116.6 (s, 1F); IR (film): 2926, 1721, 1549, 1509, 1353, 1254, 1125, 1006; HRMS Calculated For $C_{10}H_9F_4N_2O$ $[M+H]^+$ 249.0651, found: 249.0646; HPLC (OJ-H, elute: Hexanes/*i*-PrOH = 70/30, detector: 254 nm, flow rate: 0.7 mL/min), t_1 = 6.9 min (maj), t_2 = 7.4 min.

4. General Procedure for Hydrogenation of Disubstituted Fluorinated Pyrazol-5-ols 1i, 1j and Trisubstituted Fluorinated Pyrazol-5-ols 3

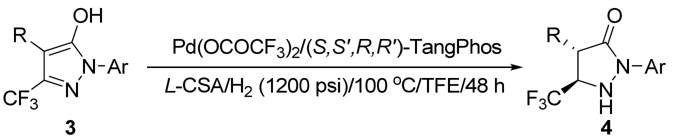

General procedure: (*S,S',R,R'*)-TangPhos (3.0 mg, 0.00104 mmol) and $Pd(OCOCF_3)_2$ (2.7 mg, 0.008 mmol) were placed in a dried Schlenk tube under nitrogen atmosphere, and degassed anhydrous acetone was added. The mixture was stirred at room temperature for 1 h, and then solvent was removed under vacuum to give the catalyst. In a glovebox, pyrazol-5-ol **3** (0.2 mmol) and the above catalyst together with trifluoroethanol (TFE, 2 mL) were stirred in 1 mL solvent at room temperature for 1 min. Subsequently, *L*-CSA (46.4 mg, 0.2 mmol) was added to the reaction mixture. The hydrogenation was performed at 100 °C under H_2 (1200 psi) in a stainless steel autoclave for 48 h. After carefully releasing the hydrogen, the resulting mixture was concentrated under vacuum and dissolved in saturated aqueous sodium bicarbonate (5 mL). After stirring for 10 min, the mixture was extracted with dichloromethane and dried over sodium sulfate. After purification by silica gel chromatography using petroleum ether/ dichloromethane (1:1) as eluent, the enantiomeric excess of the products were determined by HPLC with chiral column.

(-)-5-(Perfluoroethyl)-2-phenylpyrazolidin-3-one (2i): Pale yellow solid; mp = 120-121 °C, yield 95%, 94% ee, $[\alpha]^{20}_{D}$ = -8.4 (*c* 0.50, $CHCl_3$); ^{1}H NMR (400 MHz, $CDCl_3$) δ 7.77 (d, *J* = 7.8 Hz, 2H), 7.36 (t, *J* = 8.0 Hz, 2H), 7.16 (t, *J* = 7.4 Hz, 1H), 5.08 (d, *J* = 7.9 Hz, 1H), 4.27–4.17 (m, 1H), 3.16 (dd, *J* = 17.5, 9.3 Hz, 1H), 2.96 (dd, *J* = 17.4, 4.0 Hz, 1H); ^{13}C NMR (100 MHz, $CDCl_3$) δ 167.7, 137.80, 128.9, 125.2, 118.7, 52.8 (dd, $J_{C\text{-}F}$ = 29.0, 21.8 Hz), 33.3; ^{19}F NMR (377 MHz, $CDCl_3$) δ -81.7 (s, 3F) -121.6 (d, *J* = 278 Hz), -131.1 (d, *J* = 278 Hz); IR (film): 3243, 2938, 2852, 1684, 1592, 1547, 1496, 1367, 1213, 1127; HRMS Calculated For $C_{11}H_{10}F_5N_2O$ $[M+H]^+$ 281.0713, found: 281.0708; HPLC (AD-H, elute: Hexanes/*i*-PrOH = 70/30, detector: 254 nm, flow rate: 0.7 mL/min), t_1 = 5.6 min, t_2 = 7.0 min (maj).

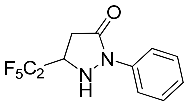

(-)-5-(Perfluoroethyl)-2-(*p*-tolyl)pyrazolidin-3-one (2j): Pale yellow solid; mp = 153-154 °C, yield 92%, 95% ee, $[\alpha]^{20}_{D}$ = -5.8 (*c* 0.50, $CHCl_3$); ^{1}H NMR (400 MHz, $CDCl_3$) δ 7.63 (d, *J* = 8.5 Hz, 2H), 7.15 (d, *J* = 8.3 Hz, 2H), 5.06 (d, *J* = 7.9 Hz, 1H), 4.26–4.13 (m, 1H), 3.14 (dd, *J* = 17.4, 9.4 Hz, 1H), 2.94 (dd, *J* = 17.4, 3.9 Hz, 1H), 2.32 (s, 3H); ^{13}C NMR (100 MHz, $CDCl_3$) δ 167.4, 135.3, 135.0, 129.4, 118.8, 52.8 (dd, *J* = 29.0, 21.8 Hz), 33.2, 20.9; ^{19}F NMR (377 MHz, $CDCl_3$) δ -81.7 (s, 3F), -121.7 (d, *J* = 278 Hz, 1F), -131.0 (d, *J* = 278 Hz, 1F); IR (film): 3259, 2923, 2862, 1695, 1550, 1510, 1348, 1216, 1125; HRMS Calculated For $C_{12}H_{12}F_5N_2O$ $[M+H]^+$ 295.0870, found: 295.0864; HPLC (OG-H, elute: Hexanes/*i*-PrOH = 90/10, detector: 254 nm, flow rate: 0.7 mL/min), t_1 = 14.8 min, t_2 = 17.1 min (maj).

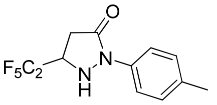

(-)-4-Methyl-2-phenyl-5-(trifluoromethyl)pyrazolidin-3-one (4a): Pale yellow solid; mp = 84-85 °C, yield 92%, 89% ee, $[\alpha]^{20}_{D}$ = -28.4 (*c* 0.50, $CHCl_3$); ^{1}H NMR (400 MHz, $CDCl_3$) δ 7.79 (d, *J* = 7.8 Hz, 2H), 7.36 (t, *J* = 8.0 Hz, 2H), 7.15 (t, *J* = 7.4 Hz, 1H), 5.02 (d, *J* = 8.8 Hz, 1H), 3.77–3.67 (m, 1H), 3.00–2.90 (m, 1H), 1.46 (d, *J* = 7.3 Hz, 3H); ^{13}C NMR (100 MHz, $CDCl_3$) δ 170.4, 137.9, 128.9, 125.1, 124.6 (q, $J_{C\text{-}F}$ = 277 Hz), 118.7, 61.6 (q, $J_{C\text{-}F}$ = 31 Hz), 39.9, 14.8; ^{19}F NMR (377 MHz, $CDCl_3$) δ -76.5 (s, 3F); IR (film): 2985, 2931, 1741, 1544, 1497, 1374, 1245, 1048; HRMS Calculated For $C_{11}H_{12}F_3N_2O$ $[M+H]^+$ 245.0902, found: 245.0896; HPLC (AD-H, elute: Hexanes/*i*-PrOH = 70/30, detector: 254 nm, flow rate: 0.7 mL/min), t_1 = 5.5 min, t_2 = 6.2 min (maj).

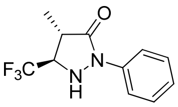

(+)-4-Methyl-2-(p-tolyl)-5-(trifluoromethyl)pyrazolidin-3-one (4b): Pale yellow oil; yield 97%, 88% ee, $[\alpha]^{20}_{D}$ = +5.8 (*c* 0.50, $CHCl_3$); ^{1}H NMR (400 MHz, $CDCl_3$) δ 7.66 (d, *J* = 8.1 Hz, 2H), 7.26–7.15 (m, 2H), 4.96 (d, *J* = 8.8 Hz, 1H), 3.75–3.68 (m, 1H), 3.00-2.92 (m, 1H), 2.33 (s, 3H), 1.45 (d, *J* = 7.3 Hz, 3H); ^{13}C NMR (100 MHz, $CDCl_3$) δ 170.1, 135.4, 134.9, 129.4, 123.7 (q, $J_{C\text{-}F}$ = 278 Hz), 118.8, 61.6 (q, $J_{C\text{-}F}$ = 31 Hz), 39.8, 20.9, 14.9; ^{19}F NMR (377 MHz, $CDCl_3$) δ -76.5 (s, 3F); IR (film): 3243, 2999, 2925, 1677, 1574, 1504, 1336, 1266, 1160; HRMS Calculated For $C_{12}H_{14}F_3N_2O$ $[M+H]^+$ 259.1058, found: 259.1053; HPLC (AD-H, elute: Hexanes/*i*-PrOH = 70/30, detector: 254 nm, flow rate: 0.7 mL/min), t_1 = 6.0 min, t_2 = 6.9 min (maj).

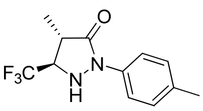

(+)-4-Ethyl-2-phenyl-5-(trifluoromethyl)pyrazolidin-3-one (4c): Pale yellow solid; mp = 58-59 °C, yield 93%, 94% ee, $[\alpha]^{20}_{D}$ = +0.4 (*c* 0.50, $CHCl_3$); ^{1}H NMR (400 MHz, $CDCl_3$) δ 7.78 (d, *J* = 7.8 Hz, 2H), 7.35 (t, *J* = 8.0 Hz, 2H), 7.14 (t, *J* = 7.4 Hz, 1H), 5.18 (d, *J* = 7.5 Hz, 1H), 3.76–3.71 (m, 1H), 2.76–2.73 (m, 1H), 1.96–1.94 (m, 1H), 1.79–1.75 (m, 1H), 1.08 (t, *J* = 7.4 Hz, 3H); ^{13}C NMR (100 MHz, $CDCl_3$) δ 170.0, 137.9, 128.8, 125.1,

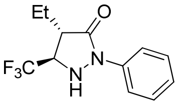

124.7 (q, $J_{C\text{-}F}$ = 278 Hz), 119.0, 58.5 (q, $J_{C\text{-}F}$ = 31 Hz), 46.3, 23.1, 11.1; ^{19}F NMR (377 MHz, $CDCl_3$) δ -77.7 (s, 3F); IR (film): 3241, 2972, 1687, 1593, 1494, 1359, 1281, 1172, 1145; HRMS Calculated For $C_{12}H_{14}F_3N_2O$ $[M+H]^+$ 259.1058, found: 259.1053; HPLC (AD-H, elute: Hexanes/*i*-PrOH = 70/30, detector: 254 nm, flow rate: 0.7 mL/min), t_1 = 5.5 min, t_2 = 6.4 min (maj).

(+)-4-Ethyl-2-*p*-tolyl-5-(trifluoromethyl)pyrazolidin-3-one (4d): Pale yellow solid; mp = 90-91 °C, yield 92%, 93% ee, $[\alpha]^{20}_D$ = +2.0 (*c* 0.50, $CHCl_3$); ^{1}H NMR (400 MHz, $CDCl_3$) δ 7.66 (d, *J* = 8.2 Hz, 2H), 7.16 (d, *J* = 8.1 Hz, 2H), 5.06 (d, *J* = 7.6 Hz, 1H), 3.76–3.72 (m, 1H), 2.79–2.76 (m, 1H), 2.32 (s, 3H), 2.01–1.90 (m, 1H), 1.84–1.78 (m, 1H), 1.10 (t, *J* = 7.3 Hz, 3H); ^{13}C NMR (100 MHz, $CDCl_3$) δ 169.6, 135.4, 135.0, 129.4, 124.6 (q, $J_{C\text{-}F}$ = 278 Hz), 119.0, 58.5 (q, $J_{C\text{-}F}$ = 32 Hz), 46.2, 23.1, 20.9, 11.1; ^{19}F NMR (377 MHz, $CDCl_3$) δ -77.7 (s, 3F); IR (film): 3230, 2965, 2870, 1675, 1579, 1513, 1359, 1265, 1170, 1149; HRMS Calculated For $C_{13}H_{16}F_3N_2O$ $[M+H]^+$ 273.1215, found: 273.1209; HPLC (OG-H, elute: Hexanes/*i*-PrOH = 95/5, detector: 254 nm, flow rate: 0.7 mL/min), t_1 = 18.6 min, t_2 = 21.8 min (maj).

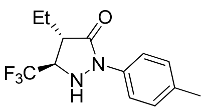

(+)-4-Ethyl-2-*m*-tolyl-5-(trifluoromethyl)pyrazolidin-3-one (4e): Pale yellow oil; yield 90%, 92% ee, $[\alpha]^{20}_D$ = +0.4 (*c* 0.50, $CHCl_3$); ^{1}H NMR (400 MHz, $CDCl_3$) δ 7.63–7.56 (m, 2H), 7.26-7.21 (m, 1H), 6.97 (d, *J* = 7.6 Hz, 1H), 5.06 (br, 1H), 3.75 (s, 1H), 2.80-2.75 (m, 1H), 2.36 (s, 3H), 2.00–1.91 (m, 1H), 1.83–1.76 (m, 1H), 1.10 (t, *J* = 7.4 Hz, 3H); ^{13}C NMR (100 MHz, $CDCl_3$) δ 169.8, 138.8, 137.8, 128.7, 126.0, 124.9 (q, $J_{C\text{-}F}$ = 278 Hz), 119.5, 116.1, 58.5 (q, $J_{C\text{-}F}$ = 31 Hz), 46.3, 23.1, 21.5, 11.1; ^{19}F NMR (377 MHz, $CDCl_3$) δ -77.7 (s, 3F); IR (film): 3255, 2969, 1713, 1548, 1490, 1360, 1283, 1184, 1148; HRMS Calculated For $C_{13}H_{16}F_3N_2O$ $[M+H]^+$ 273.1215, found: 273.1209; HPLC (AD-H, elute: Hexanes/*i*-PrOH = 70/30, detector: 254 nm, flow rate: 0.7 mL/min), t_1 = 5.4 min, t_2 = 6.9 min (maj).

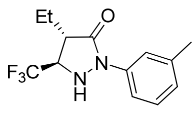

(+)-2-Phenyl-4-propyl-5-(trifluoromethyl)pyrazolidin-3-one (4f): Pale yellow solid; mp = 96-70 °C, yield 95%, 93% ee, $[\alpha]^{20}_D$ = +2.6 (*c* 0.50, $CHCl_3$); ^{1}H NMR (400 MHz, $CDCl_3$) δ 7.80 (d, *J* = 8.3 Hz, 2H), 7.36 (t, *J* = 7.9 Hz, 2H), 7.15 (t, *J* = 7.4 Hz, 1H), 5.08 (br, 1H), 3.77–3.73 (m, 1H), 2.86 (d, *J* = 3.1 Hz, 1H), 1.93–1.74 (m, 1H), 1.56–1.54 (m, 1H), 1.55–1.50 (m, 2H), 1.00 (t, *J* = 7.3 Hz, 3H); ^{13}C NMR (100 MHz, $CDCl_3$) δ 170.2, 137.9, 128.8, 126.4 (q, $J_{C\text{-}F}$ = 278 Hz), 125.1, 118.9, 58.9 (q, $J_{C\text{-}F}$ = 32 Hz), 44.7, 31.9, 20.1, 13.6; ^{19}F NMR (377 MHz, $CDCl_3$) δ -77.8 (s, 3F); IR (film): 3237, 2972, 2870, 1714, 1551, 1504, 1354, 1279, 1177, 1157, 1014; HRMS Calculated For $C_{13}H_{16}F_3N_2O$ $[M+H]^+$ 273.1215, found: 273.1209; HPLC (AD-H, elute: Hexanes/*i*-PrOH = 70/30, detector: 254 nm, flow rate: 0.7 mL/min), t_1 = 5.6 min, t_2 = 7.2 min (maj).

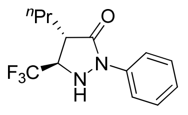

(4*S*,5*R*)-4-Benzyl-2-phenyl-5-(trifluoromethyl)pyrazolidin-3-one (4g): Pale yellow solid; mp = 117-118 °C, yield 94%, 95% ee, $[\alpha]^{20}_D$ = +122.6 (*c* 0.50, $CHCl_3$); ^{1}H NMR (400 MHz, $CDCl_3$) δ 7.71 (d, *J* = 8.0 Hz, 2H), 7.37–7.25 (m, 7H), 7.15 (t, *J* = 7.4 Hz, 1H), 4.19 (s, 1H), 3.76–3.73 (m, 1H), 3.37–3.31 (m, 1H), 3.23–3.19 (m, 1H), 3.13-3.08 (m, 1H); ^{13}C NMR (100 MHz, $CDCl_3$) δ 169.4, 137.6, 136.1, 129.1, 129.0, 128.8, 127.7, 125.3, 125.2 (q, $J_{C\text{-}F}$ = 279 Hz), 119.1, 57.7 (q, $J_{C\text{-}F}$ = 32 Hz), 45.7, 35.4; ^{19}F NMR (377 MHz, $CDCl_3$) δ -77.5 (s, 3F); IR (film): 3243, 3055, 2918, 1667, 1592, 1498, 1487, 1457, 1372, 1271, 1201, 1166, 1031; HRMS Calculated For $C_{17}H_{16}F_3N_2O$ $[M+H]^+$ 321.1215, found: 321.1209; HPLC (AD-H,

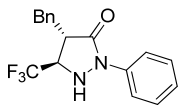

elute: Hexanes/*i*-PrOH = 70/30, detector: 254 nm, flow rate: 0.7 mL/min), t_1 = 6.5 min (maj), t_2 = 7.1 min.

5. Mechanistic Investigation

In order to further verify our hypothesis that the hydrogenation carried out *via* capture of the active tautomer, we synthesized three compounds (OH- form type **5**, NH- form type **6** and CH- form type **8**) and exposure them to hydrogenation reaction. 5-Methoxy-1-phenyl-3-(trifluoro-methyl)-1*H*-pyrazole **5** and 1-methyl-2-phenyl-5-(trifluoro-methyl)-1*H*-pyrazol-3(2*H*)- one **6** were prepared according to the literature methods and their NMR data matched the literature data.[2,3] The 4,4-dimethyl-1-phenyl-3-(trifluoromethyl)-1*H*-pyrazol-5(4*H*)-one **8** was prepared following the literature report.[4]

Synthesis of 4,4-Dimethyl-1-phenyl-3-(trifluoromethyl)-1*H*-pyrazol-5(4*H*)-one 8

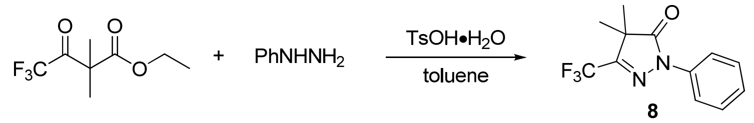

8

A solution of ethyl 4,4,4-trifluoro-2,2-dimethyl-3-oxobutanoate (0.849 g, 4.0 mmol) and phenylhydrazine (0.433 g, 4 mmol) in toluene (20 ml) was stirred and refluxed in the presence of a catalytic amount of *p*-toluenesulfonic acid monohydrate to remove water with a Dean-Stark apparatus. After 16 h reflux, the reaction mixture was concentrated under vacuum. The residue dissolved in acetic acid (5 ml) was refluxed for 4 h. After evaporation of the solvent, the residue was extracted with diethyl ether. The extract was washed successively with 3% hydrochloric acid, saturated aqueous sodium hydrogen carbonate and brine, and dried over sodium sulfate. The residue was purified by silica gel column chromatography (petroleum ester/CH_2Cl_2 = 1/1) to afford a yellow oil: 0.261 g, 26% yield; ^{1}H NMR (400 MHz, $CDCl_3$) δ 7.88–7.85 (m, 2H), 7.46–7.40 (m, 2H), 7.28–7.22 (m, 1H), 1.49 (s, 6H); ^{13}C NMR (100 MHz, $CDCl_3$) δ 175.5, 153.1 (q, $J_{C\text{-}F}$ = 37 Hz), 137.3, 129.1, 126.2, 119.5 (q, $J_{C\text{-}F}$ = 271 Hz), 119.1, 48.8, 21.3; ^{19}F NMR (377 MHz, $CDCl_3$) δ -65.1 (s, 3F); IR (film): 2964, 2892, 1650, 1408, 1319, 1251, 1216, 1049; HRMS Calculated For $C_{12}H_{12}F_3N_2O$ $[M+H]^+$ 257.0902, found: 257.0896.

General Procedure for Hydrogenation of Derivatives of Pyrazol-5-ols 5, 6 and 8: (*S*)-MeO-Biphep (6.1 mg, 0.0104 mmol) and $Pd(OCOCF_3)_2$ (2.7 mg, 0.008 mmol) were placed in a dried Schlenk tube under nitrogen atmosphere, and degassed anhydrous acetone was added. The mixture was stirred at room temperature for 1 h, and then solvent was removed under vacuum to give the catalyst. In a glovebox, **5**, **6** or **8** (0.2 mmol) and the above catalyst together with dichloromethane (2 mL) were stirred at room temperature for 1 min. Subsequently, trifluoroacetic acid (22.8 mg, 14.8 μL, 0.2 mmol) was added to the reaction mixture. The hydrogenation was performed at 60 ^{o}C under H_2 (1200 psi) in a stainless steel autoclave for 48 h. After carefully releasing the hydrogen, the resulting mixture was concentrated under vacuum and dissolved in saturated aqueous sodium bicarbonate (5 mL). After stirring for 10 min, the mixture was extracted with dichloromethane and dried over sodium sulfate. After purification by silica gel chromatography using petroleum ether/dichloromethane (1:1) as eluent, the enantiomeric excess of the products were determined by HPLC with chiral column.

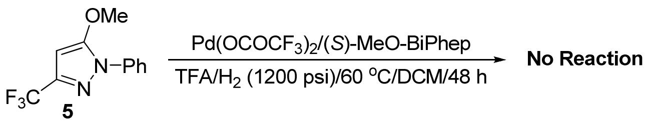

The exposure of the OH- form type substrate **5** with TFA in the presence of $Pd(OCOCF_3)_2$/(*S*)-MeO-BiPhep in dichloromethane failed to reaction.

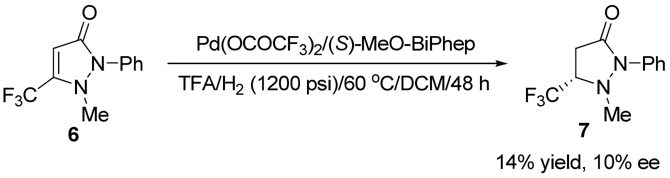

The NH-form type substrate **6** only obtained 10% ee with 14% yield. **(+)-1-Methyl-2-phenyl-5-(trifluoromethyl)pyrazolidin-3-one (7):** Pale yellow oil; yield 14%, 10% ee, $[\alpha]^{20}_D$ = +2.0 (*c* 0.20, $CHCl_3$); ^1H NMR (400 MHz, $CDCl_3$) δ 7.72–7.68 (m, 2H), 7.41–7.35 (m, 2H), 7.21–7.16 (m, 1H), 3.68–3.60 (m, 1H), 3.40–3.33 (m, 1H), 2.77–2.72 (m, 4H); ^{13}C NMR (100 MHz, $CDCl_3$) δ 166.6, 136.2, 129.0, 125.6, 124.0 (q, J_{C-F} = 278 Hz), 120.5, 61.5 (q, J_{C-F} = 31 Hz), 45.1, 30.6; ^{19}F NMR (377 MHz, $CDCl_3$) δ -78.7 (s, 3F); IR (film): 3017, 2927, 2851, 1710, 1546, 1496, 1264, 1215, 1112; HRMS Calculated For $C_{11}H_{12}F_3N_2O$ $[M+H]^+$ 245.0902, found: 245.0896; HPLC (OD-H, elute: Hexanes/*i*-PrOH = 90/10, detector: 254 nm, flow rate: 0.8 mL/min), t_1 = 9.2 min (maj), t_2 = 10.1 min.

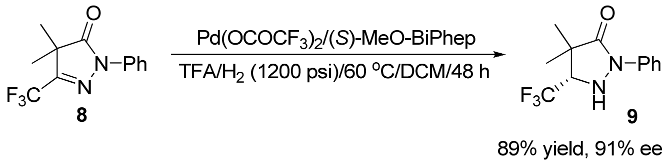

When employing the CH-form type **8** as the hydrogenation substrate, 91% ee and 89% yield was obtained. **(+)-4,4-Dimethyl-2-phenyl-5-(trifluoromethyl)pyrazolidin-3-one (9):** Yield 89%, Pale yellow oil; 91% ee, $[\alpha]^{20}_D$ = +3.0 (*c* 0.20, $CHCl_3$); ^1H NMR (400 MHz, $CDCl_3$) δ 7.84–7.81 (m, 2H), 7.39–7.34 (m, 2H), 7.15 (t, *J* = 7.4 Hz, 1H), 4.95 (d, *J* = 8.8 Hz, 1H), 3.78–3.69 (m, 1H), 1.42 (s, 3H), 1.36–1.34 (m, 3H); ^{13}C NMR (100 MHz, $CDCl_3$) δ 173.1, 138.2, 128.9, 125.4 (q, J_{C-F} = 279 Hz), 124.9, 118.6, 64.6 (q, J_{C-F} = 29 Hz), 44.2, 24.3, 18.3; ^{19}F NMR (377 MHz, $CDCl_3$) δ -71.0 (s, 3F); IR (film): 3021, 2987, 2834, 1680, 1533, 1459, 1390, 1120; HRMS Calculated For $C_{12}H_{14}F_3N_2O$ $[M+H]^+$ 259.1058, found: 259.1053; HPLC (OD-H, elute: Hexanes/*i*-PrOH = 90/10, detector: 254 nm, flow rate: 0.8 mL/min), t_1 = 11.2 min (maj), t_2 = 12.4 min.

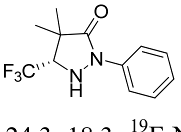

Based on the experimental results and stereochemistry of the products, we proposed that the reaction experienced the process of Brønsted acid promoted tautomerization of the three tautomeric forms and then Pd-catalyzed asymmetric hydrogenation of the active tautomer **C** to give the optically active pyrazolidinones.

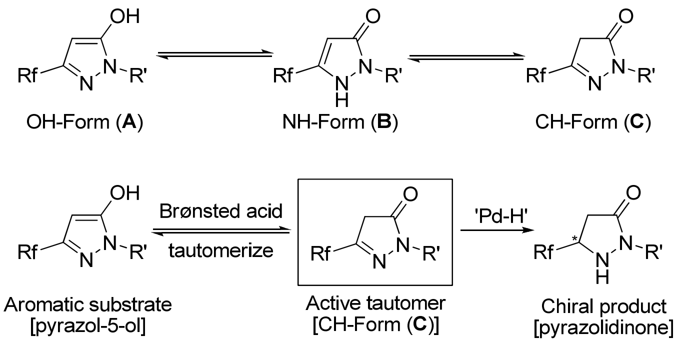

6. The X-ray Crystallographic Analysis of Substrate 3a and Products

The configuration of fluorinated 4-methyl-1-phenyl-3-(trifluoromethyl)-1*H*-pyrazol-5-ol **3a** was determined by X-ray diffraction analysis. The CCDC1040656 contains detail supplementary crystallographic data for this paper. These can be obtained free of charge from The Cambridge Crystallographic Data Centre via www.ccdc.cam.ac.uk.

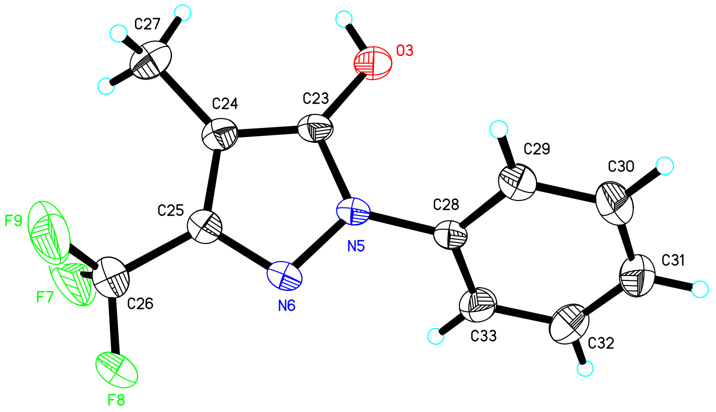

Figure 1. The X-ray structure of 4-methyl-1-phenyl-3-(trifluoromethyl)-1*H*-pyrazol-5-ol **3a**.

The absolute configuration of 2,5-disubstituted hydrogenation product **2a** was assigned as (*S*)-2-phenyl-5-(trifluoromethyl)pyrazolidin-3-one based on X-ray diffraction analysis after recrystallization from mixture solvent dichloromethane/*n*-hexane to upgrade ee to >99%. The configurations of the other chiral products are assigned by analogy. The CCDC1040657 contains detail supplementary crystallographic data for this paper. These can be obtained free of charge from The Cambridge Crystallographic Data Centre via www.ccdc.cam.ac.uk.

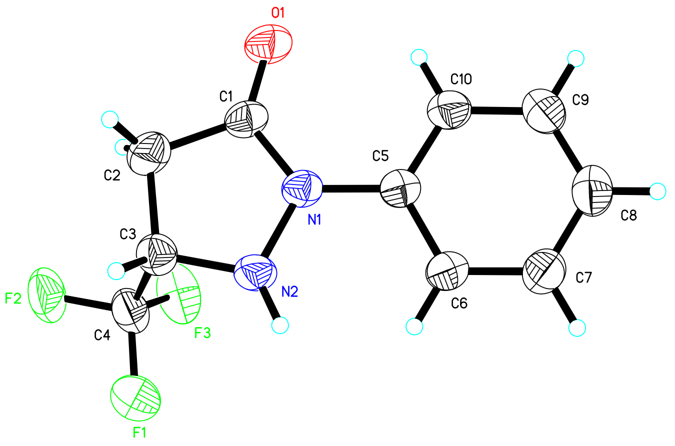

Figure 2. The X-ray structure of (*S*)-2-Phenyl-5-(trifluoromethyl)pyrazolidin-3-one **2a**.

The absolute configuration of 2,4,5-trisubstituted hydrogenation product **4g** was assigned as (4*S*,5*R*)-4-benzyl-2-phenyl-5-(trifluoromethyl)pyrazolidin-3-one based on X-ray diffraction analysis after recrystallization from mixture solvent dichloromethane/*n*-hexane to upgrade ee to >99%. The configurations of the other chiral products are assigned by analogy. The CCDC 1040658 contains detail supplementary crystallographic data for this paper. These can be obtained free of charge from The Cambridge Crystallographic Data Centre via www.ccdc.cam.ac.uk.

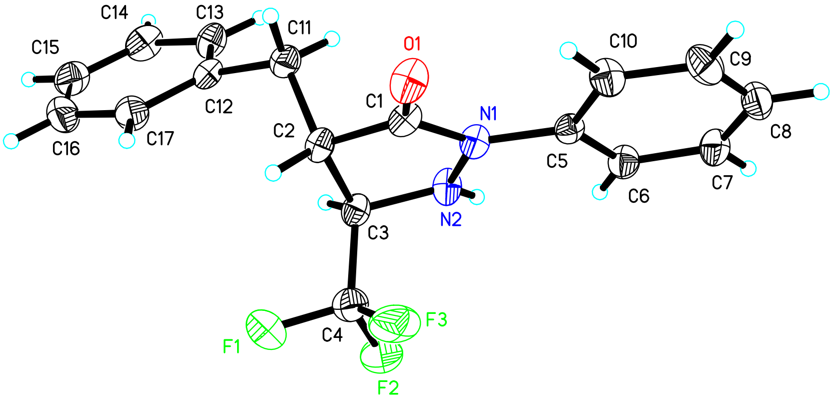

Figure 3. X-ray structure of (4*S*,5*R*)-4-benzyl-2-phenyl-5-(trifluoromethyl)pyrazolidin-3-one **4g**.

7. References

[1] J. Zhang, S. Yang, K. Zhang, J. Chen, H. Deng, M. Shao, H. Zhang, W. Cao, *Tetrahedron* **2012**, *9*, 2121.

[2] S. Bieringer, W. Holzer, *Heterocycles* **2006**, *68*, 1825.

[3] G. Grillot , S. Aftergut, D. Botteron, *J. Org. Chem.* **1958**, *23*, 119.

[4] T. Tada, M. Motoki, N. Takahashi, T. Miyata, T. Takechi, T. Uchida, Y. Takagi, *Pesticide Science* **1996**, *2*, 165.

8. Copy of NMR of Substrates and Products

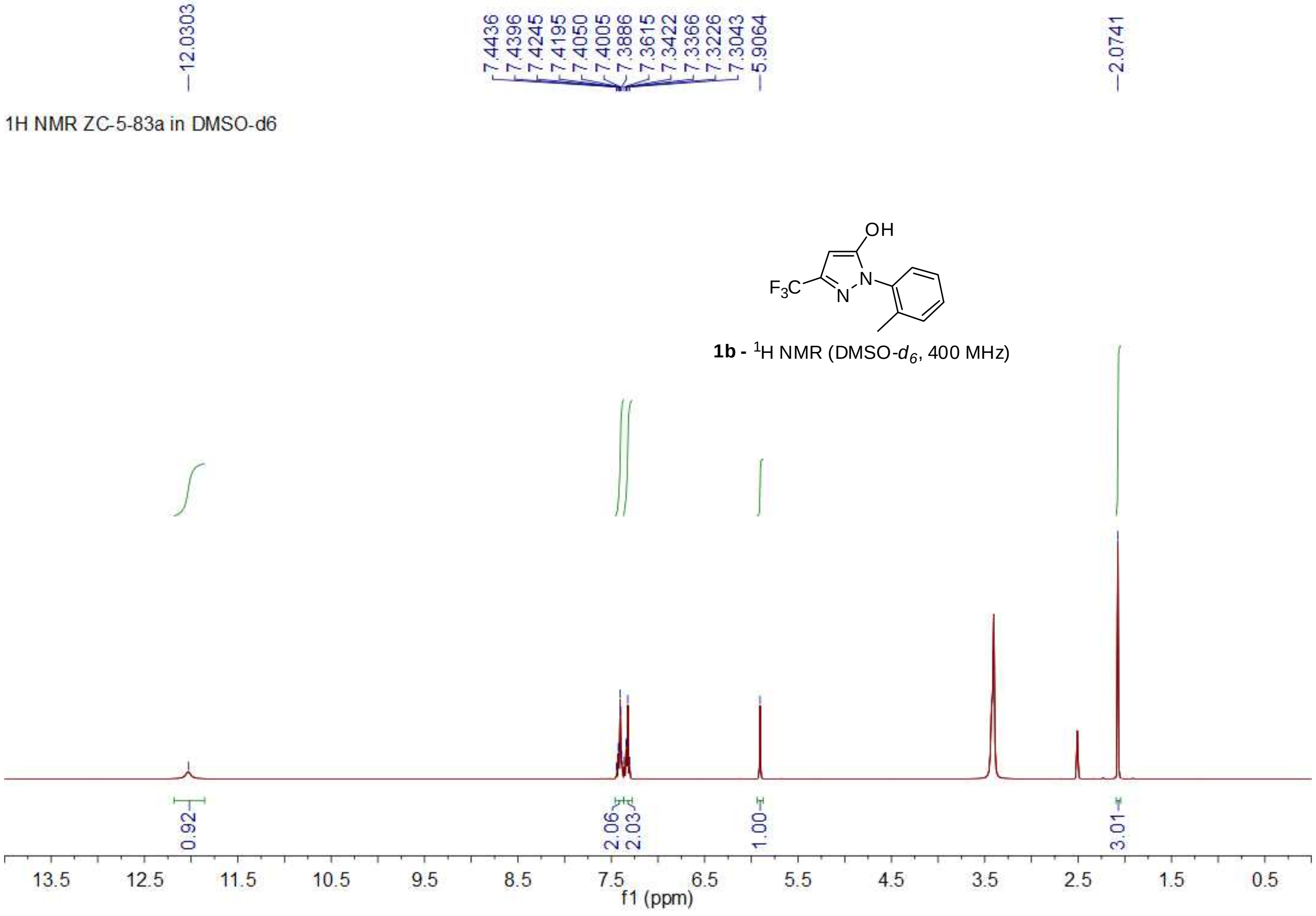

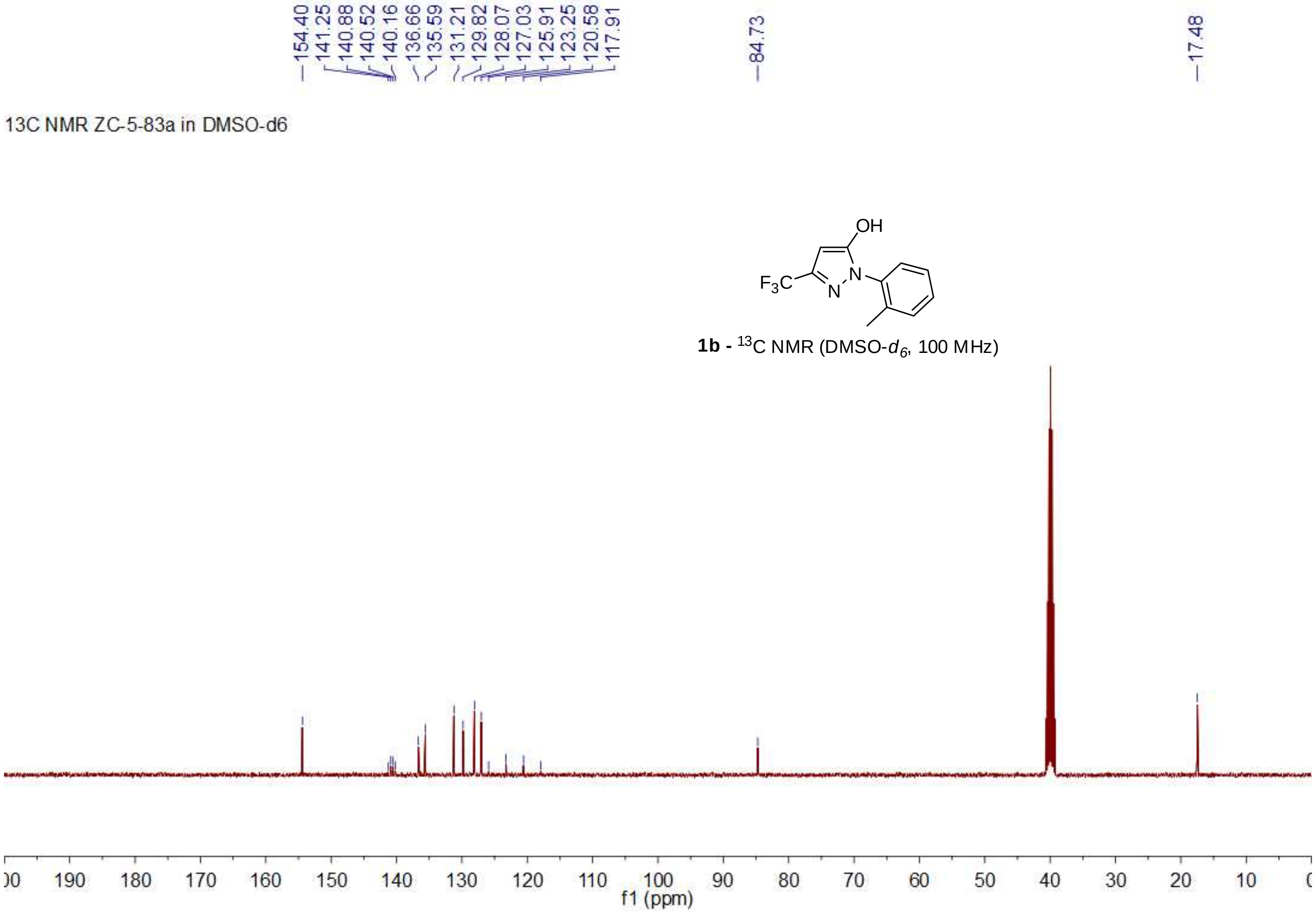

1b - ^{13}C NMR (DMSO-d_6, 100 MHz)

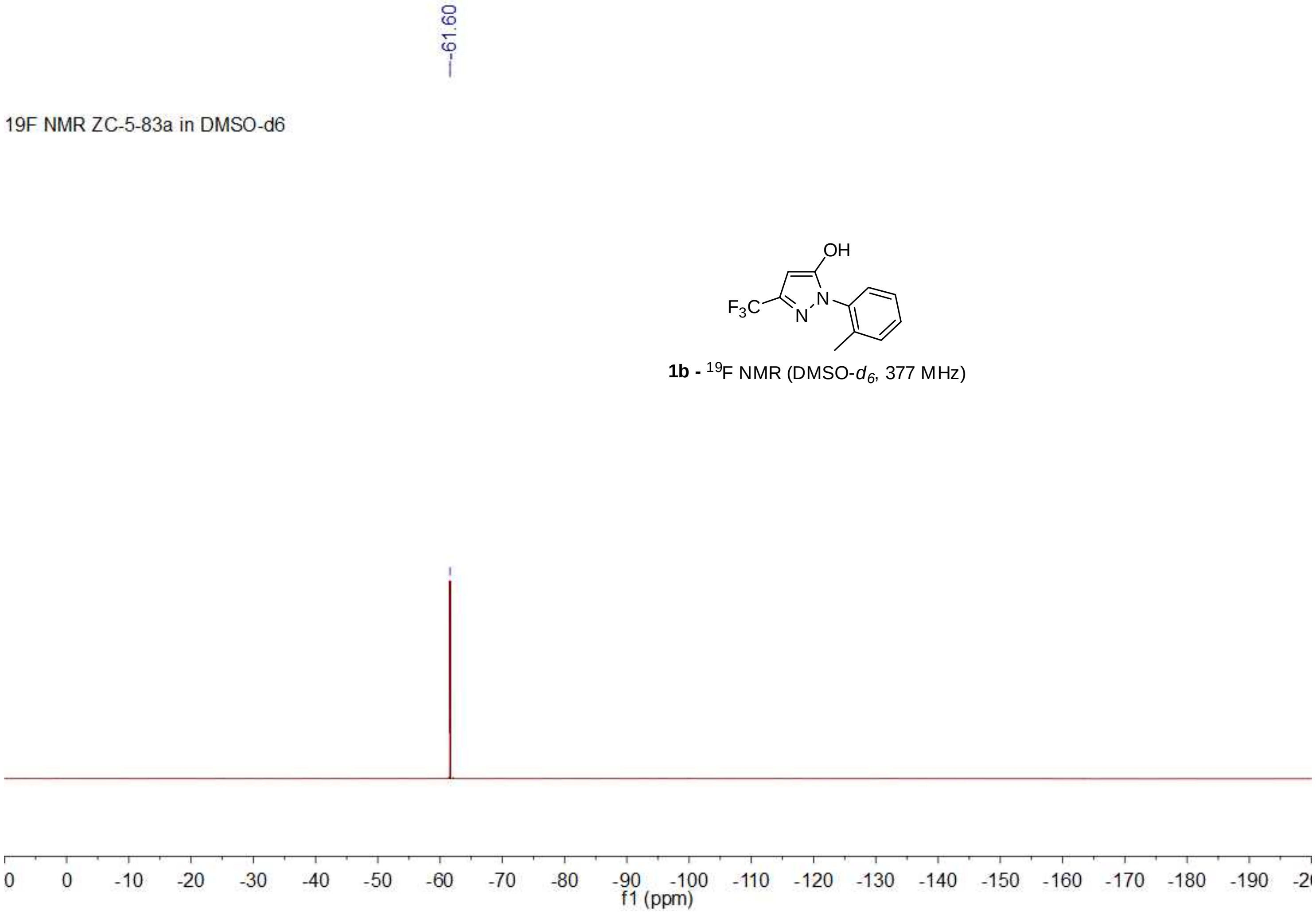

1b - ^{19}F NMR (DMSO-d_6, 377 MHz)

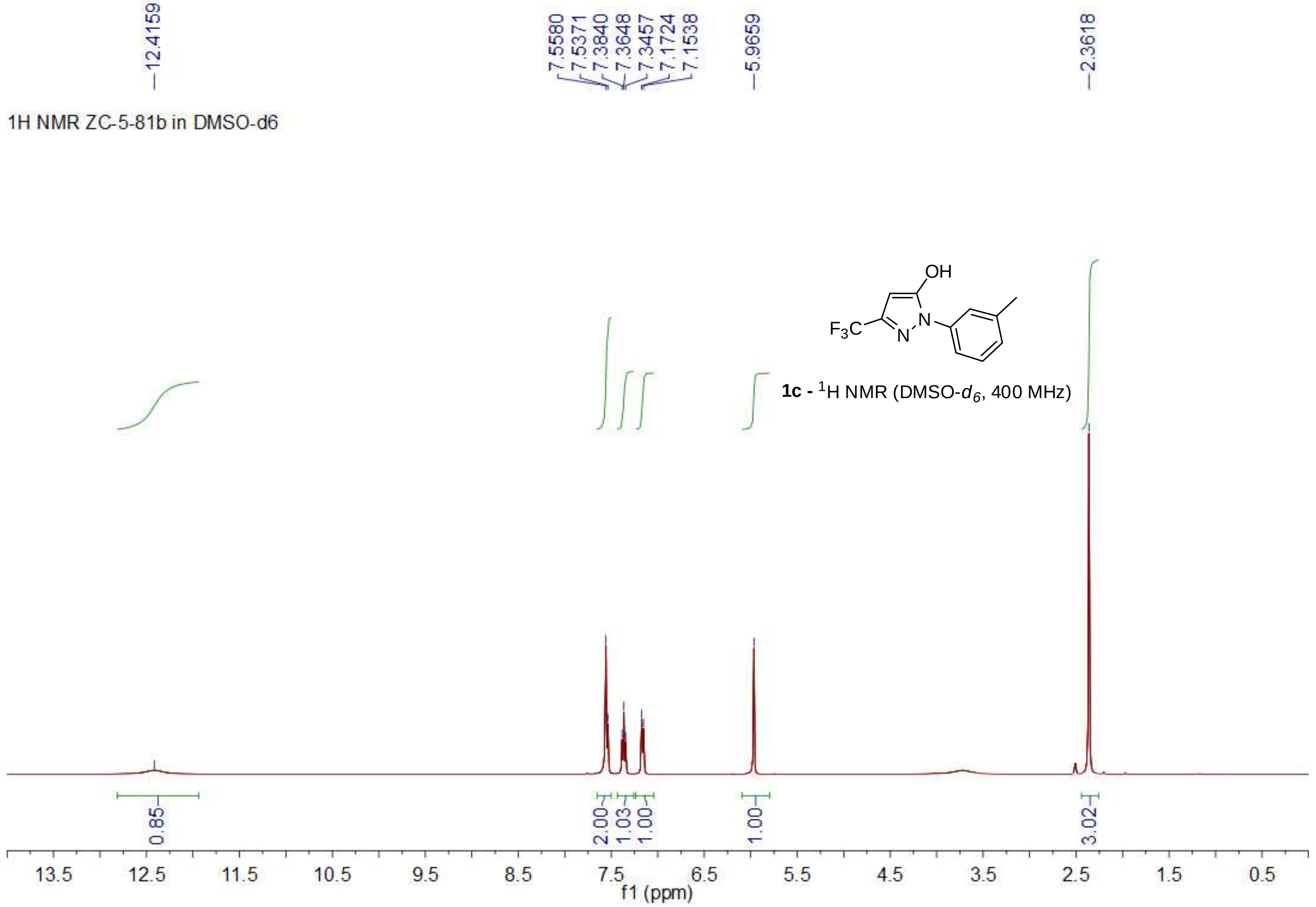
1H NMR ZC-5-81b in DMSO-d6
12.4159
7.5580
7.5371
7.3840
7.3648
7.3457
7.1724
7.1538
5.9659
2.3618
OH
N
N
F3C
1c - 1H NMR (DMSO-d6, 400 MHz)
0.85
2.00
1.03
1.00
1.00
3.02
13.5
12.5
11.5
10.5
9.5
8.5
7.5
6.5
5.5
4.5
3.5
2.5
1.5
0.5
f1 (ppm)

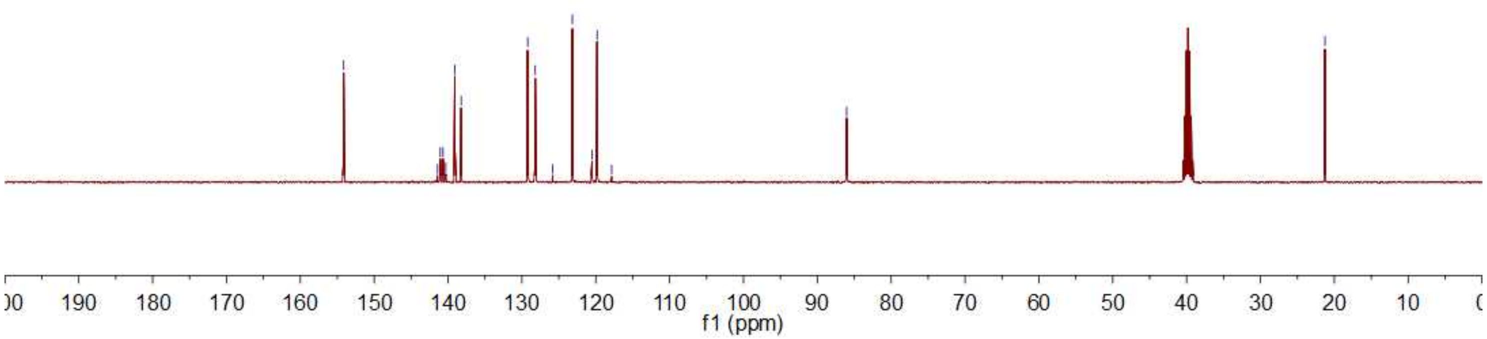

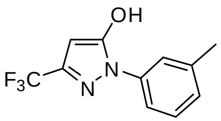

1c - ^{13}C NMR (DMSO-d_6, 100 MHz)

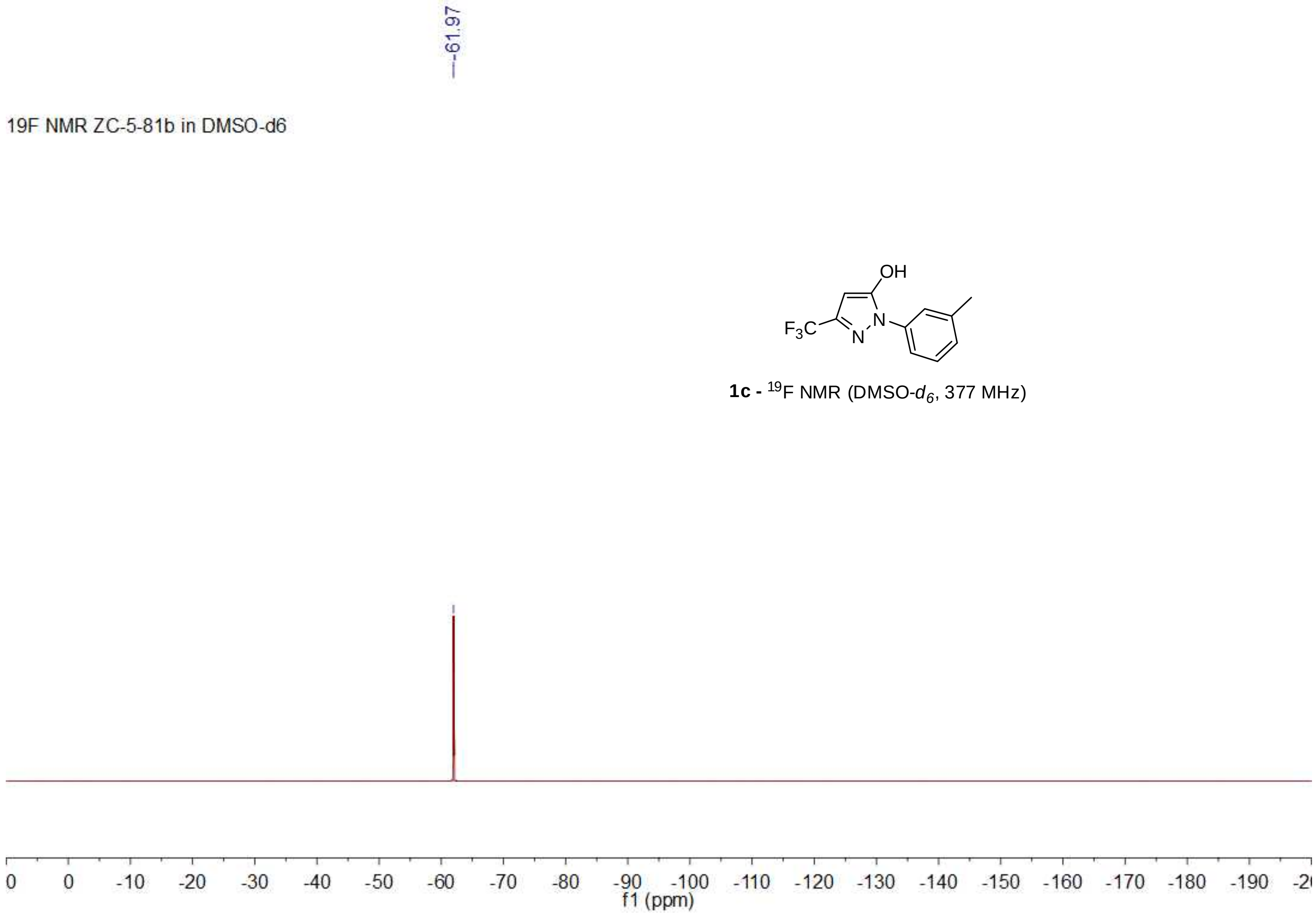

1c - ^{19}F NMR (DMSO-d_6, 377 MHz)

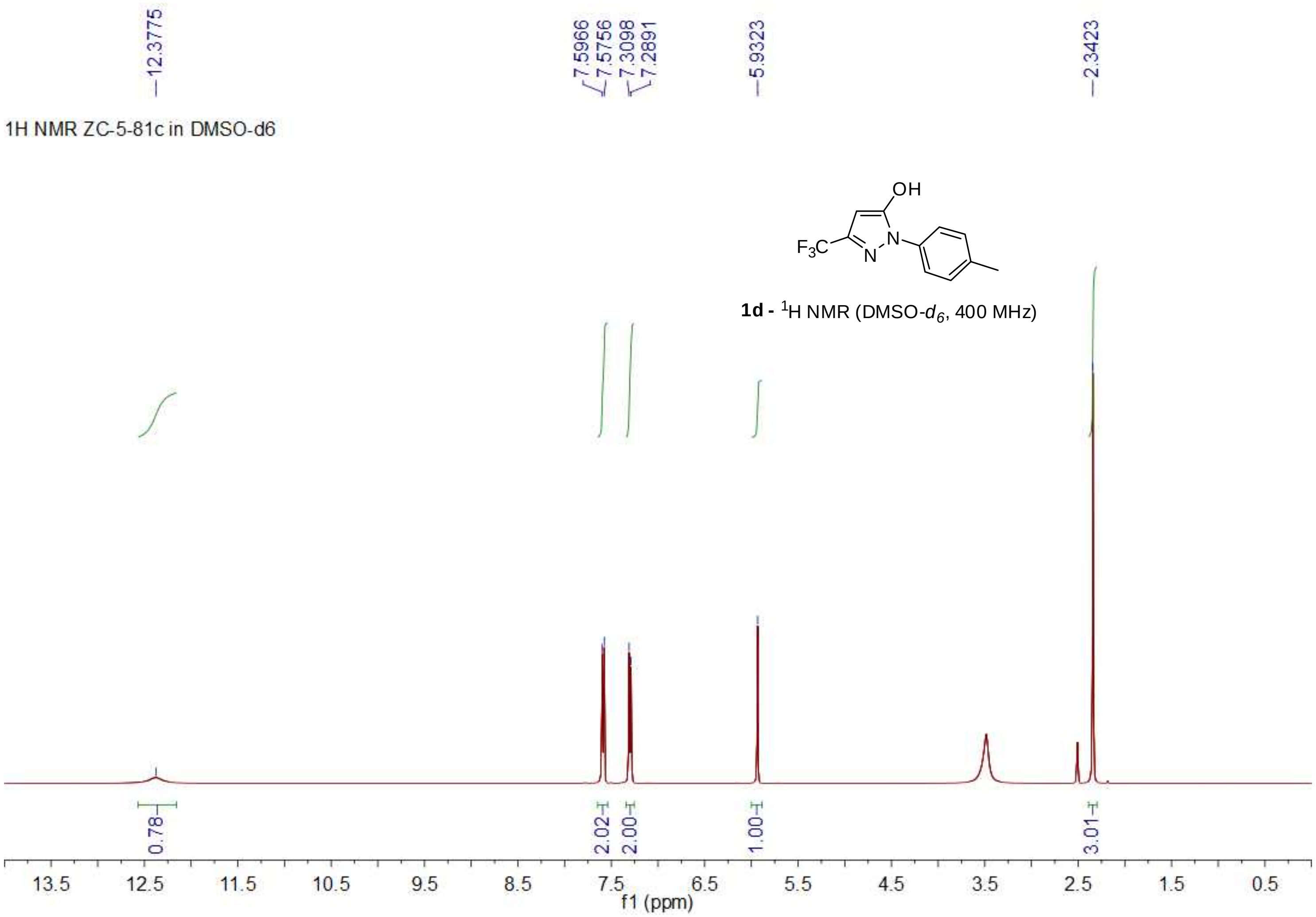
1H NMR ZC-5-81c in DMSO-d6
12.3775
7.5966
7.5756
7.3098
7.2891
5.9323
2.3423
OH
N
N
F3C
1d - 1H NMR (DMSO-d6, 400 MHz)
0.78
2.02
2.00
1.00
3.01
13.5
12.5
11.5
10.5
9.5
8.5
7.5
6.5
5.5
4.5
3.5
2.5
1.5
0.5
f1 (ppm)

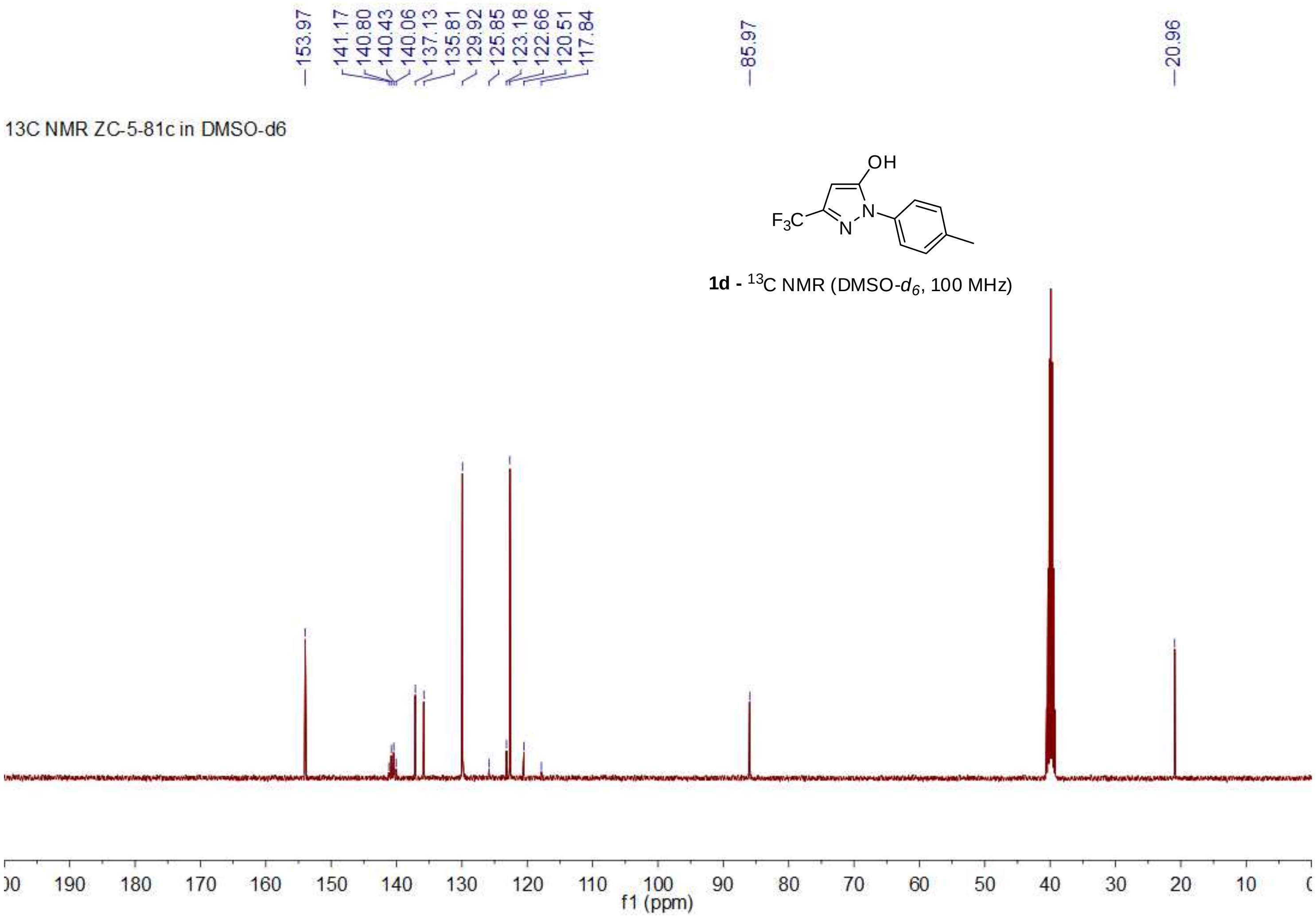

1d - ^{13}C NMR (DMSO-d_6, 100 MHz)

-61.80

19F NMR ZC-5-81c in DMSO-d6

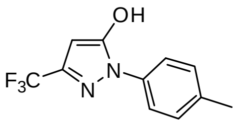

1d - ^{19}F NMR (DMSO-d_6, 377 MHz)

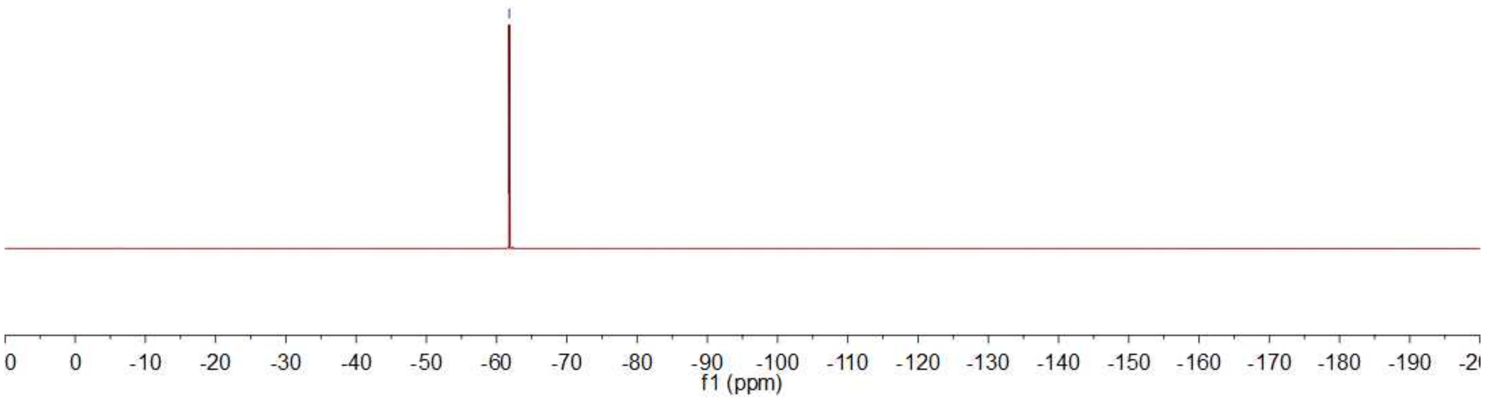

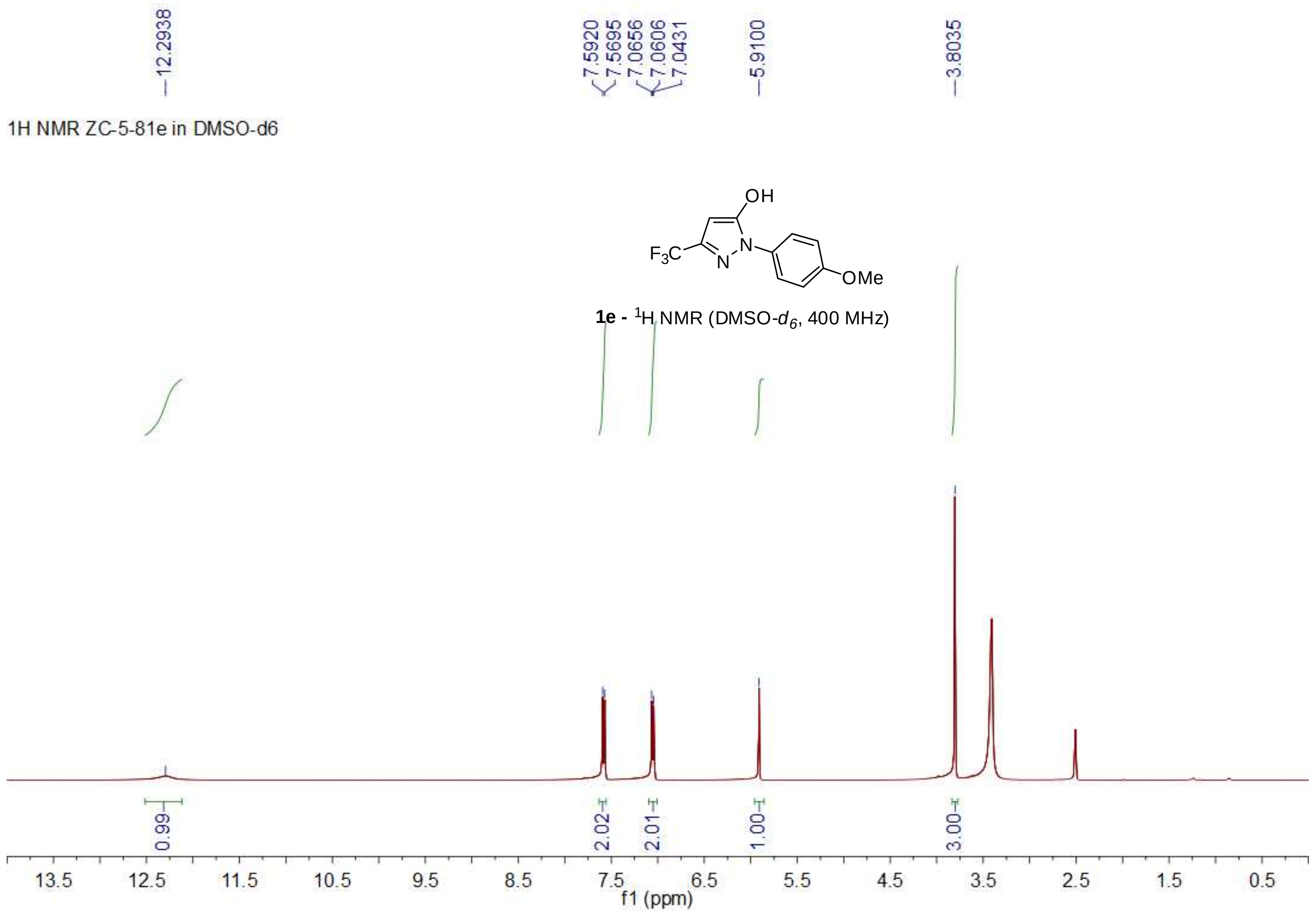

12.2938
7.5920
7.5695
7.0656
7.0606
7.0431
5.9100
3.8035
1H NMR ZC-5-81e in DMSO-d6
OH
F3C
N
N
OMe
1e - 1H NMR (DMSO-d6, 400 MHz)
0.99
2.02
2.01
1.00
3.00
13.5
12.5
11.5
10.5
9.5
8.5
7.5
6.5
5.5
4.5
3.5
2.5
1.5
0.5
f1 (ppm)

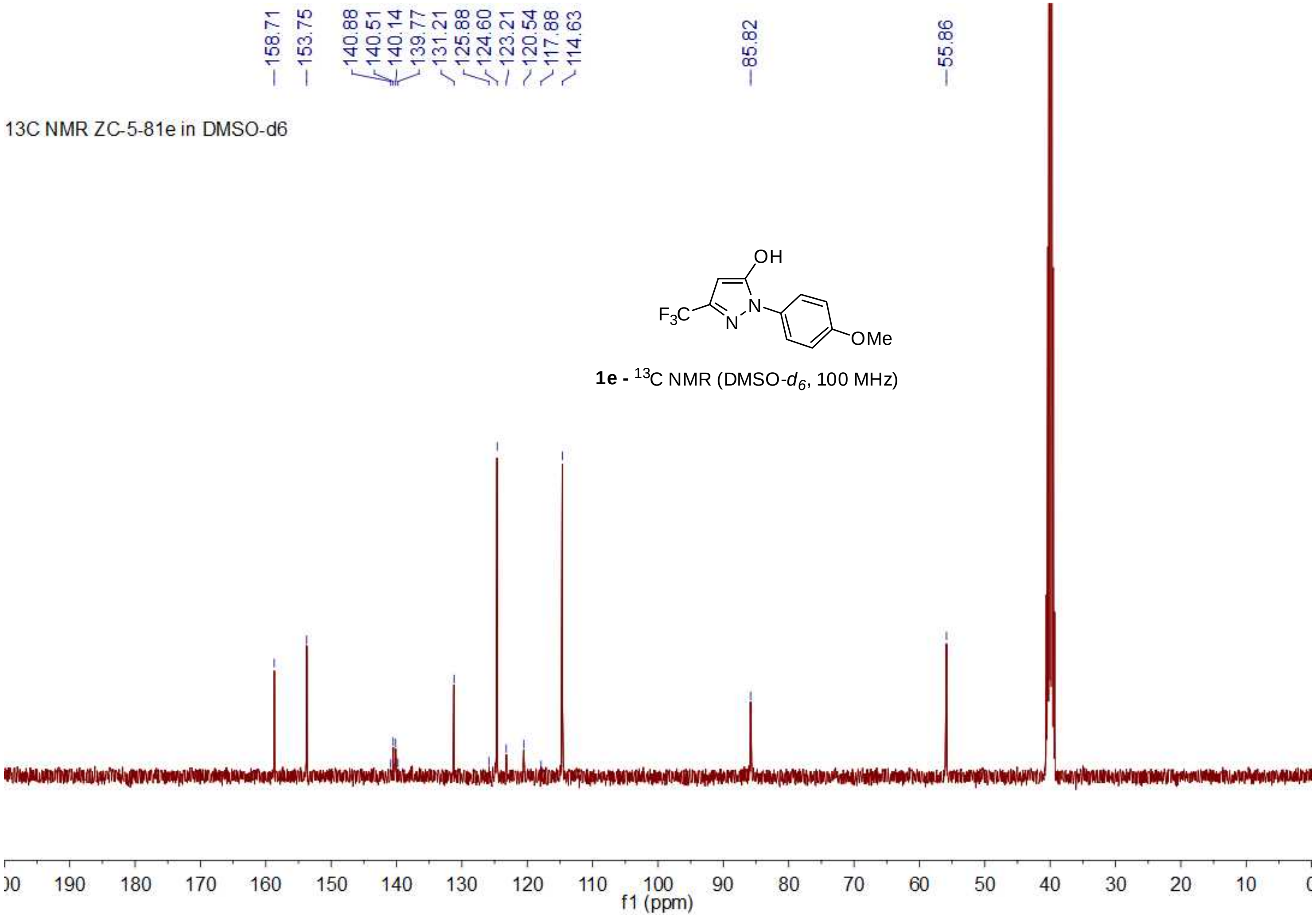
13C NMR ZC-5-81e in DMSO-d6
158.71
153.75
140.88
140.51
140.14
139.77
131.21
125.88
124.60
123.21
120.54
117.88
114.63
85.82
55.86
OH
N
N
F3C
OMe
1e - 13C NMR (DMSO-d6, 100 MHz)
f1 (ppm)
190
180
170
160
150
140
130
120
110
100
90
80
70
60
50
40
30
20
10

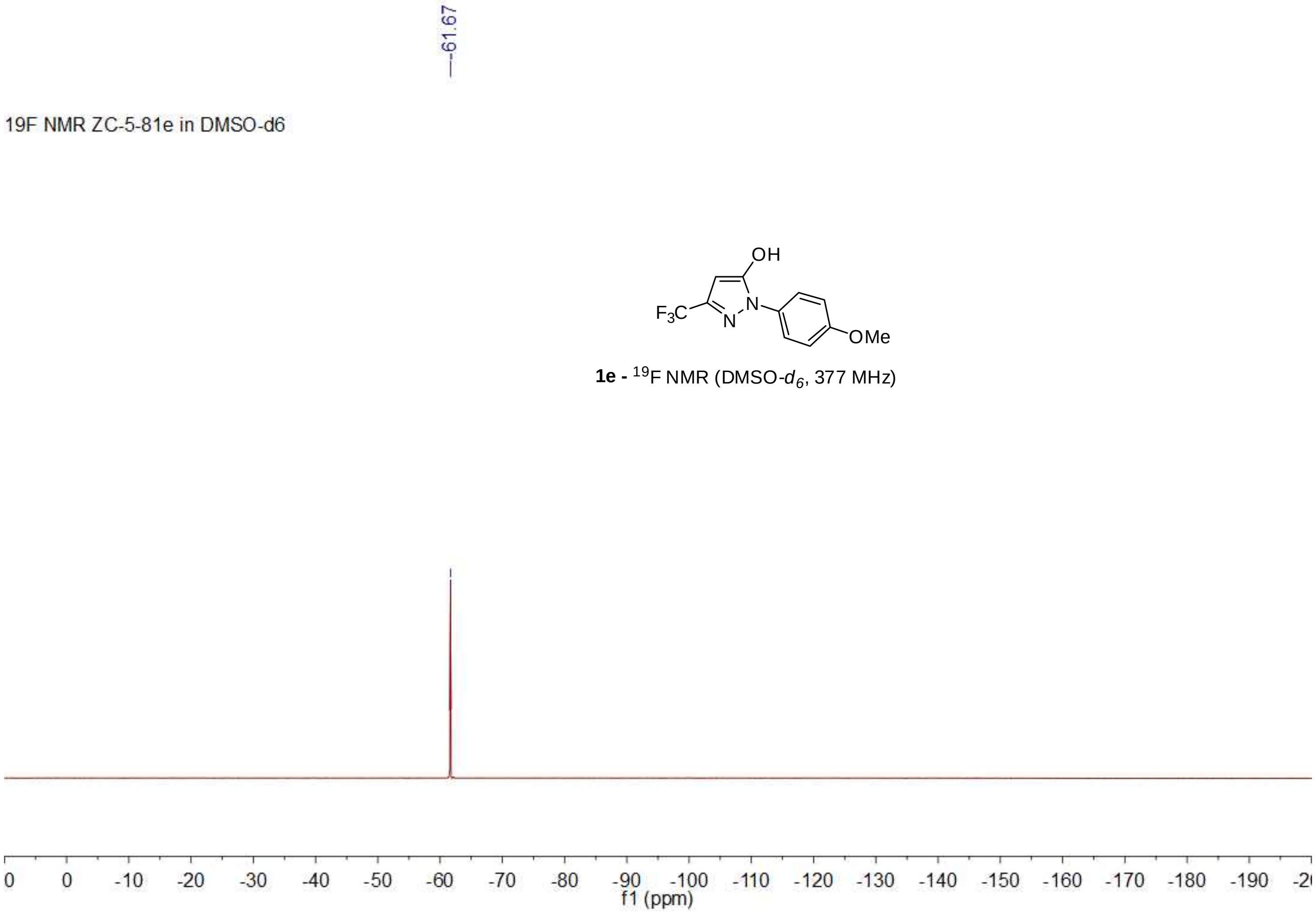

1e - ^{19}F NMR (DMSO-d_6, 377 MHz)

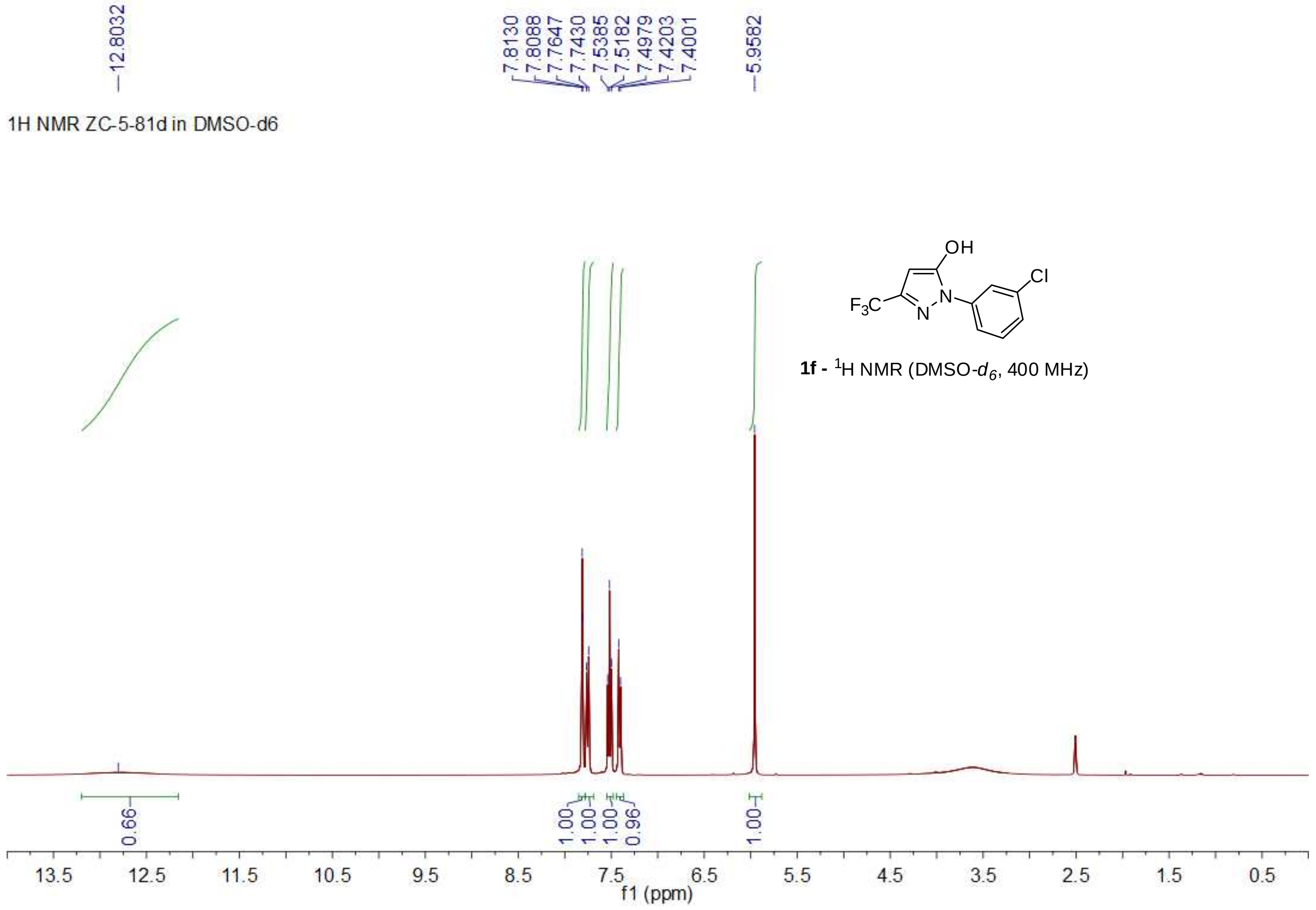

1H NMR ZC-5-81d in DMSO-d6
12.8032
7.8130
7.8088
7.7647
7.7430
7.5385
7.5182
7.4979
7.4203
7.4001
5.9582
OH
Cl
F3C
N
N
1f - 1H NMR (DMSO-d6, 400 MHz)
0.66
1.00
1.00
1.00
0.96
1.00
13.5
12.5
11.5
10.5
9.5
8.5
7.5
6.5
5.5
4.5
3.5
2.5
1.5
0.5
f1 (ppm)

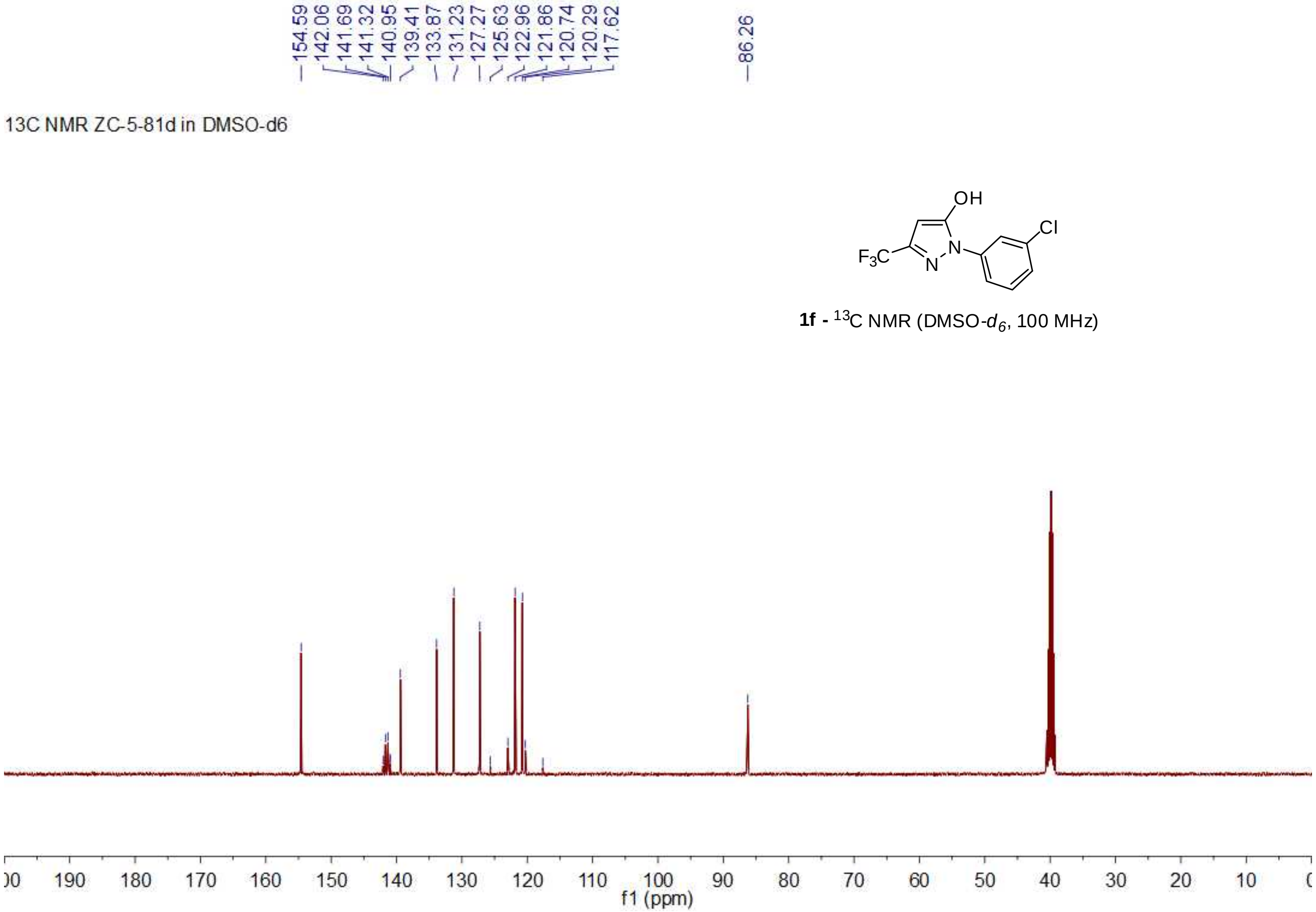

1f - ^{13}C NMR (DMSO-d_6, 100 MHz)

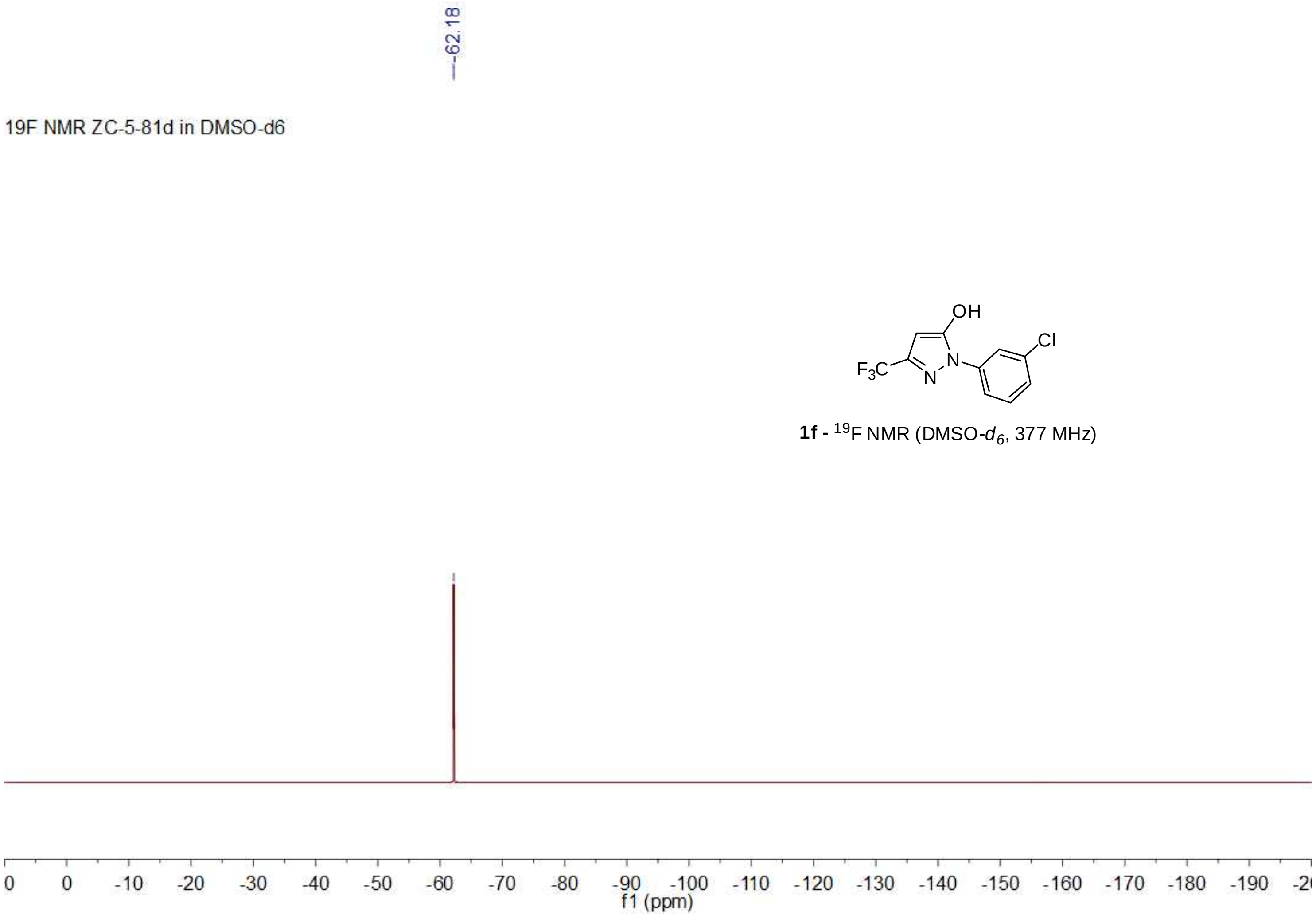

1f - ^{19}F NMR (DMSO-d_6, 377 MHz)

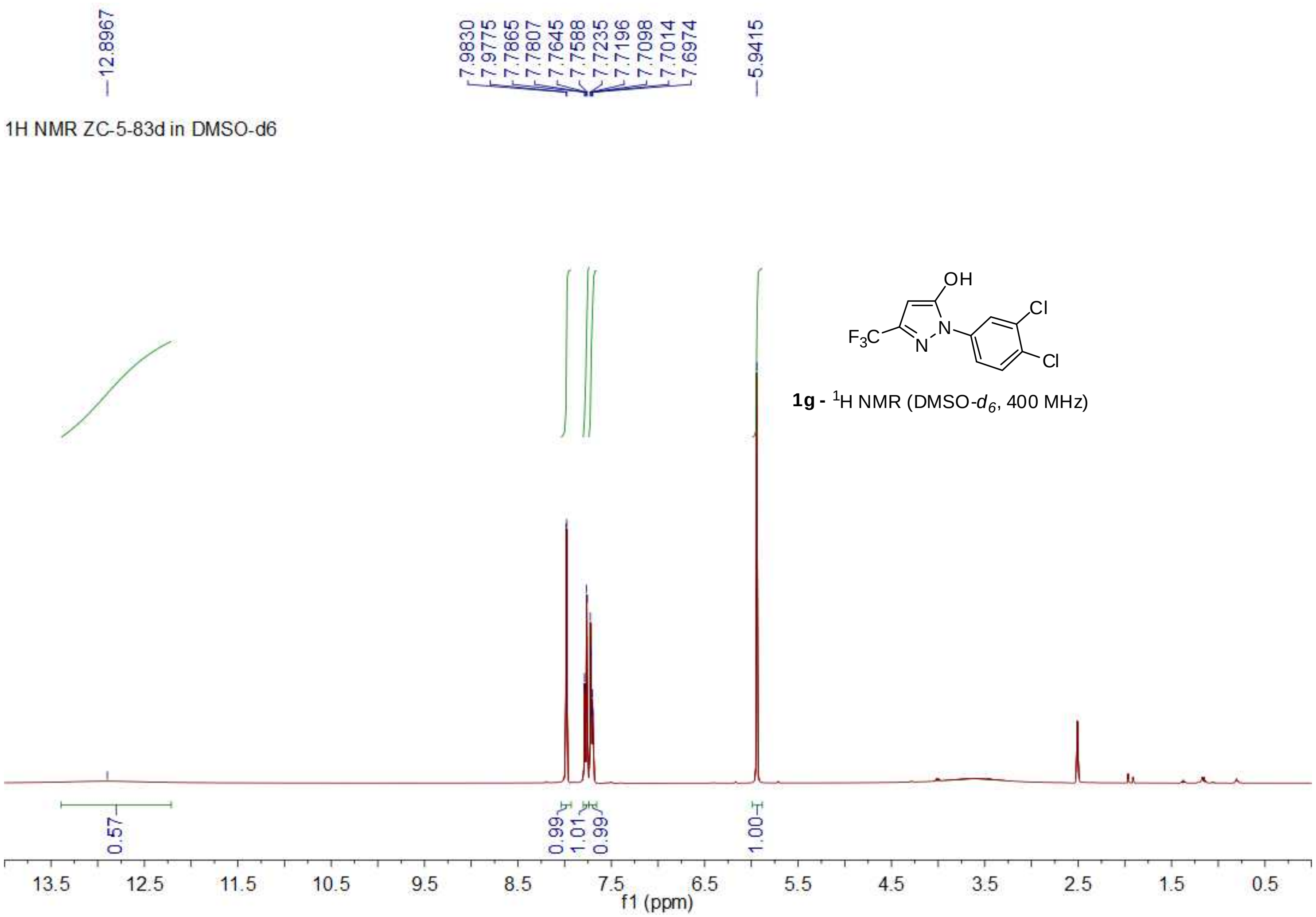
1H NMR ZC-5-83d in DMSO-d6
12.8967
7.9830
7.9775
7.7865
7.7807
7.7645
7.7588
7.7235
7.7196
7.7098
7.7014
7.6974
5.9415
OH
Cl
F3C
N
N
Cl
1g - 1H NMR (DMSO-d6, 400 MHz)
0.57
0.99
1.01
0.99
1.00
13.5
12.5
11.5
10.5
9.5
8.5
7.5
6.5
5.5
4.5
3.5
2.5
1.5
0.5
f1 (ppm)

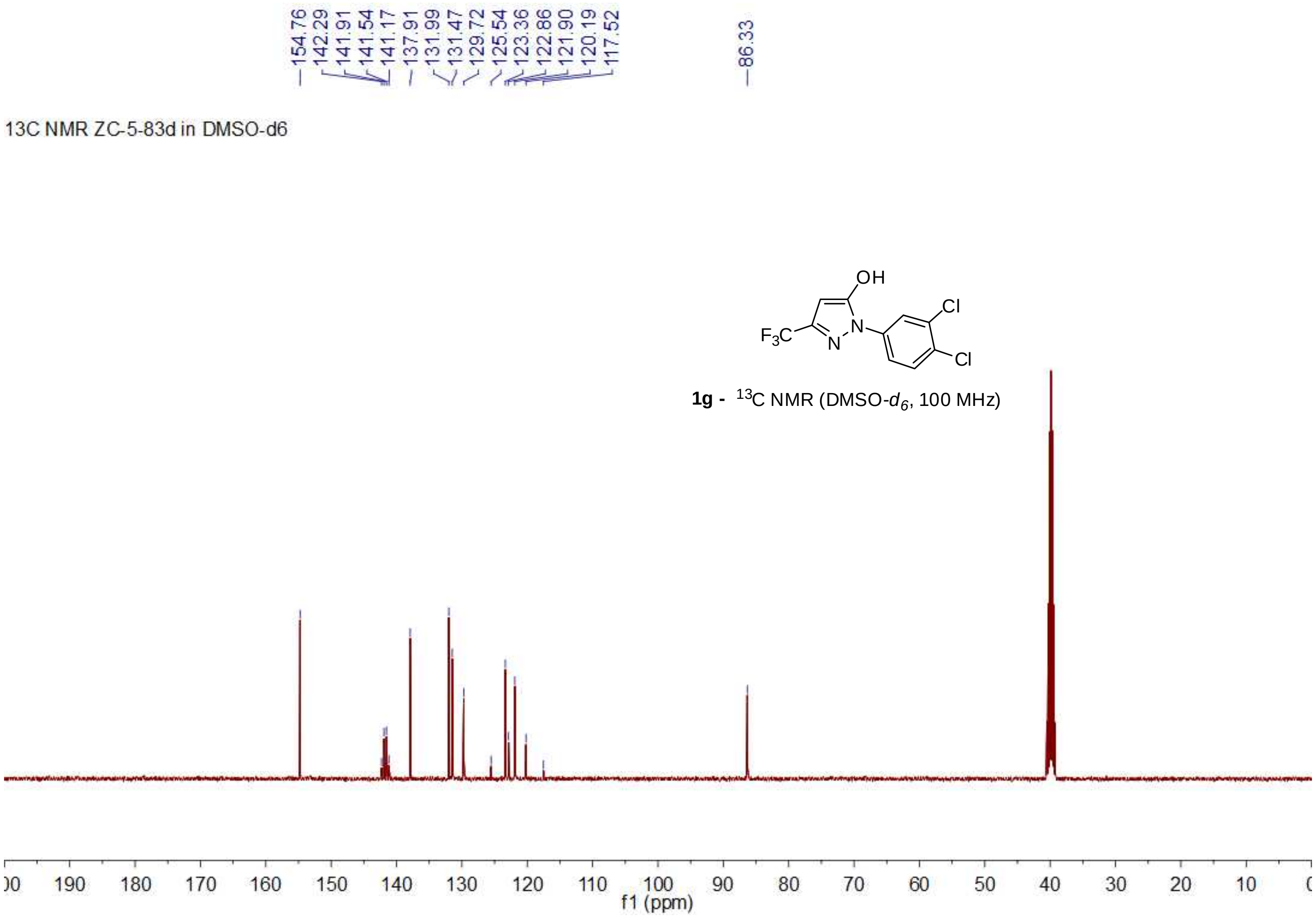
154.76
142.29
141.91
141.54
141.17
137.91
131.99
131.47
129.72
125.54
123.36
122.86
121.90
120.19
117.52
86.33
13C NMR ZC-5-83d in DMSO-d6
OH
Cl
Cl
F3C
N
N
1g - 13C NMR (DMSO-d6, 100 MHz)
190
180
170
160
150
140
130
120
110
100
90
80
70
60
50
40
30
20
10
f1 (ppm)

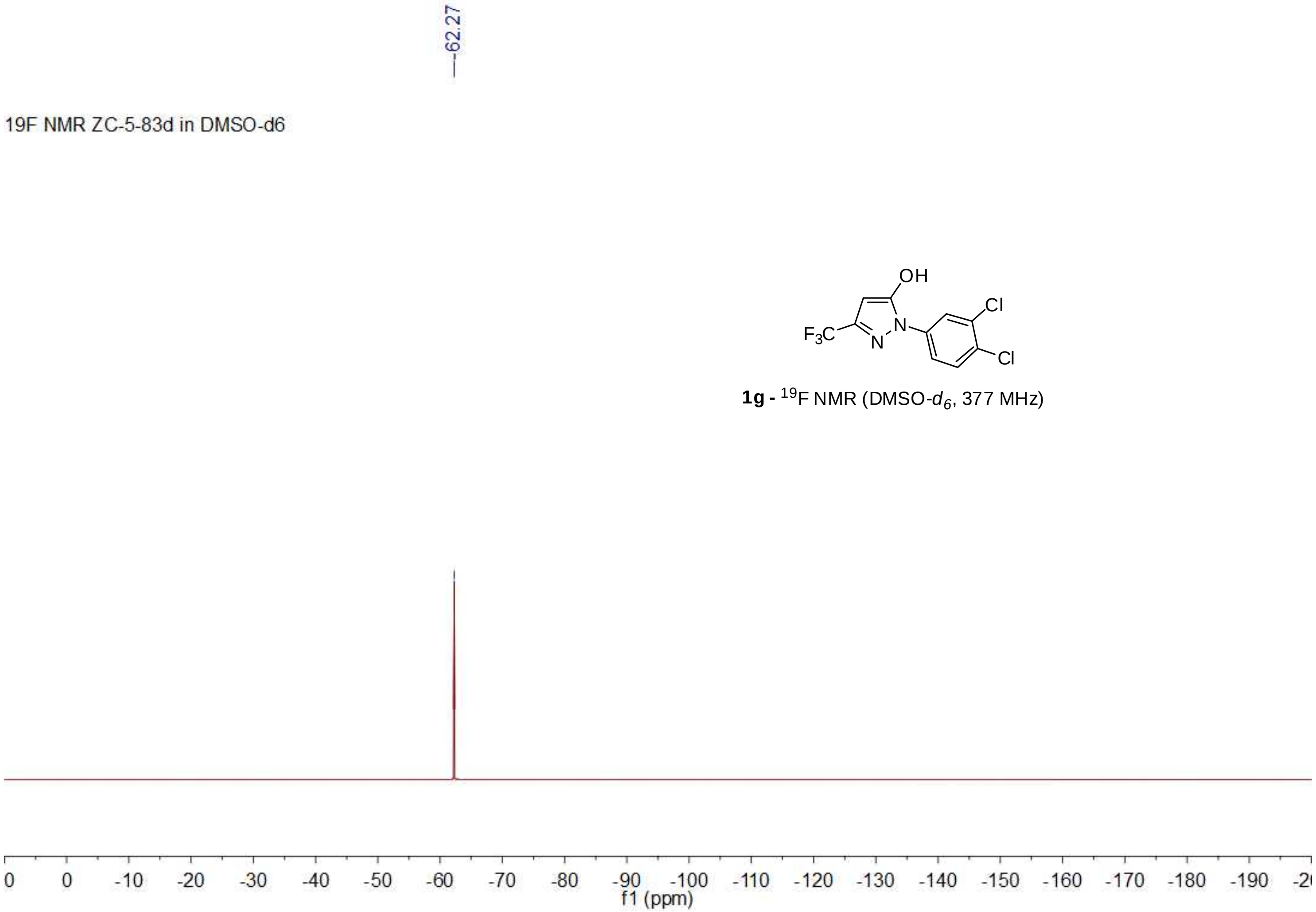

1g - ^{19}F NMR (DMSO-d_6, 377 MHz)

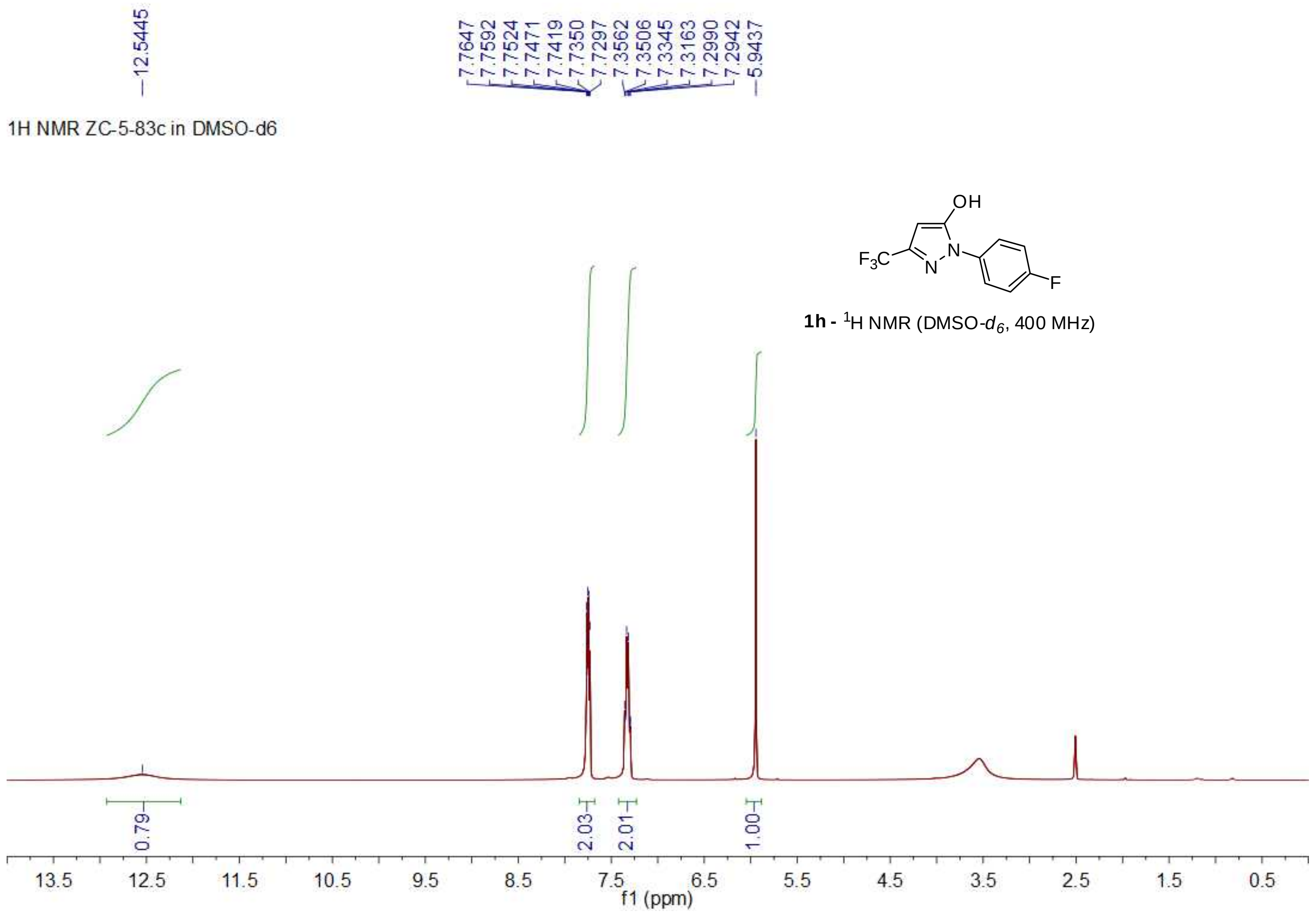

1h - ^{1}H NMR (DMSO-d_6, 400 MHz)

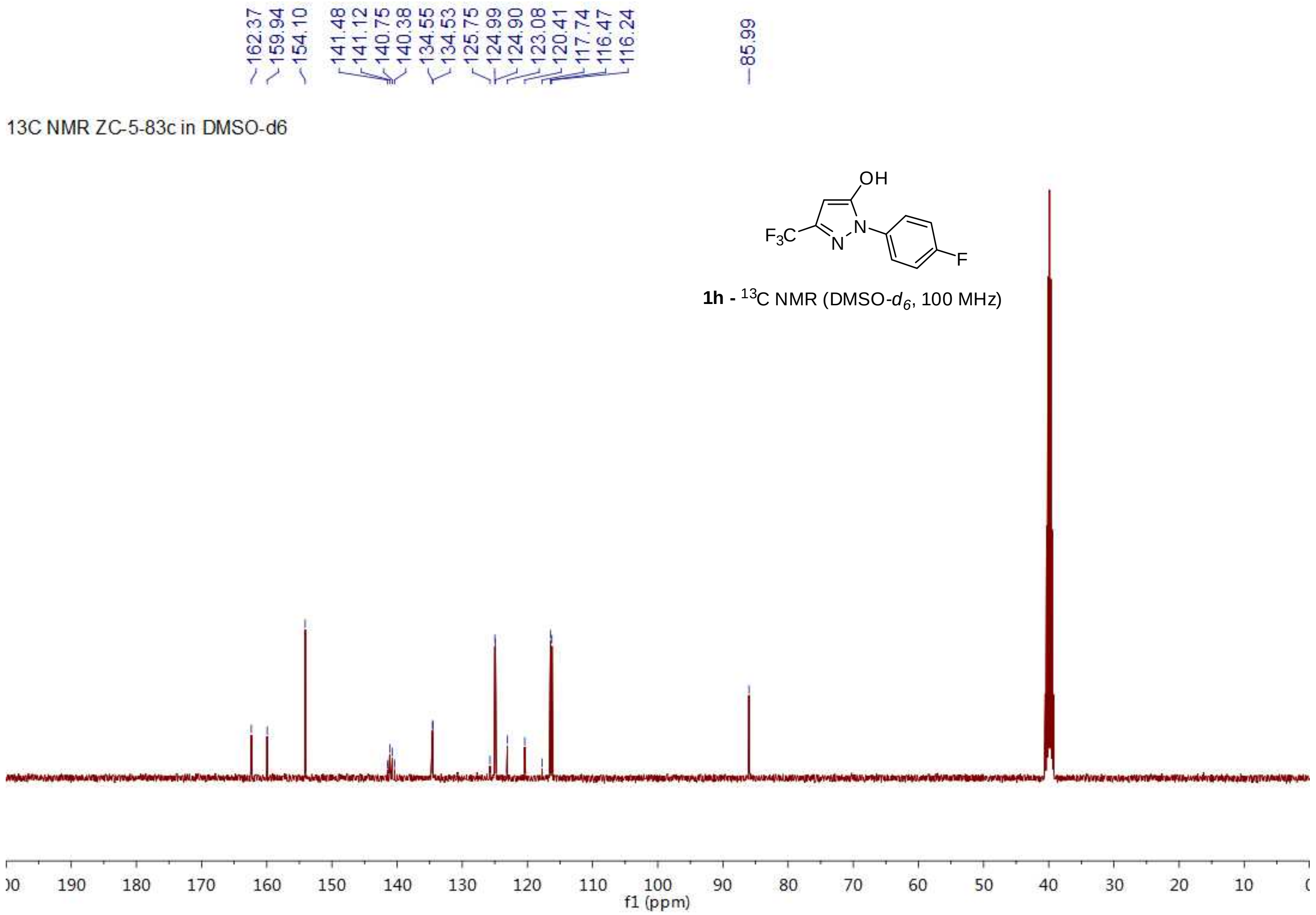

1h - ^{13}C NMR (DMSO-d_6, 100 MHz)

19F NMR ZC-5-83c in DMSO-d6

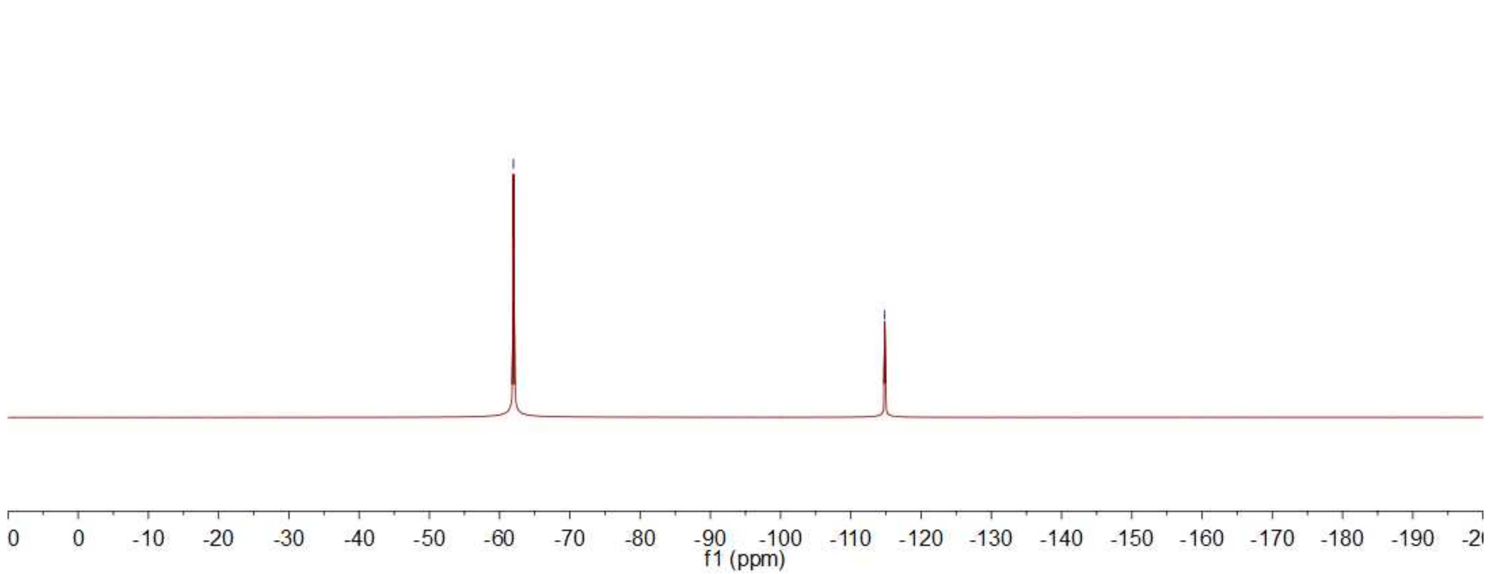

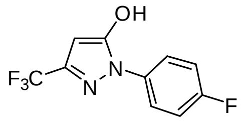

1h - ^{19}F NMR (DMSO-d_6, 377 MHz)

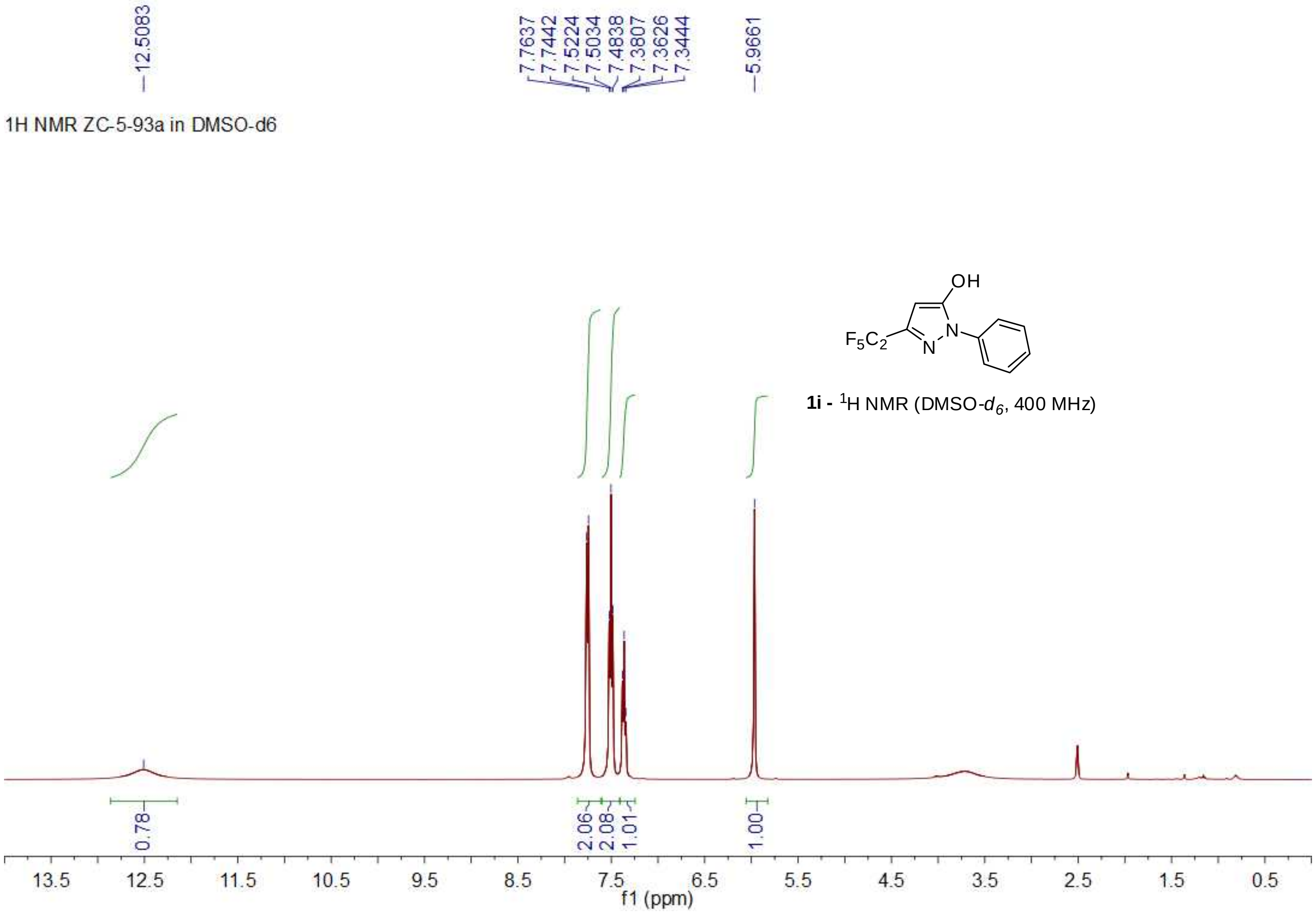

1i - ^{1}H NMR (DMSO-d_6, 400 MHz)

13C NMR ZC-5-93a in DMSO-d6

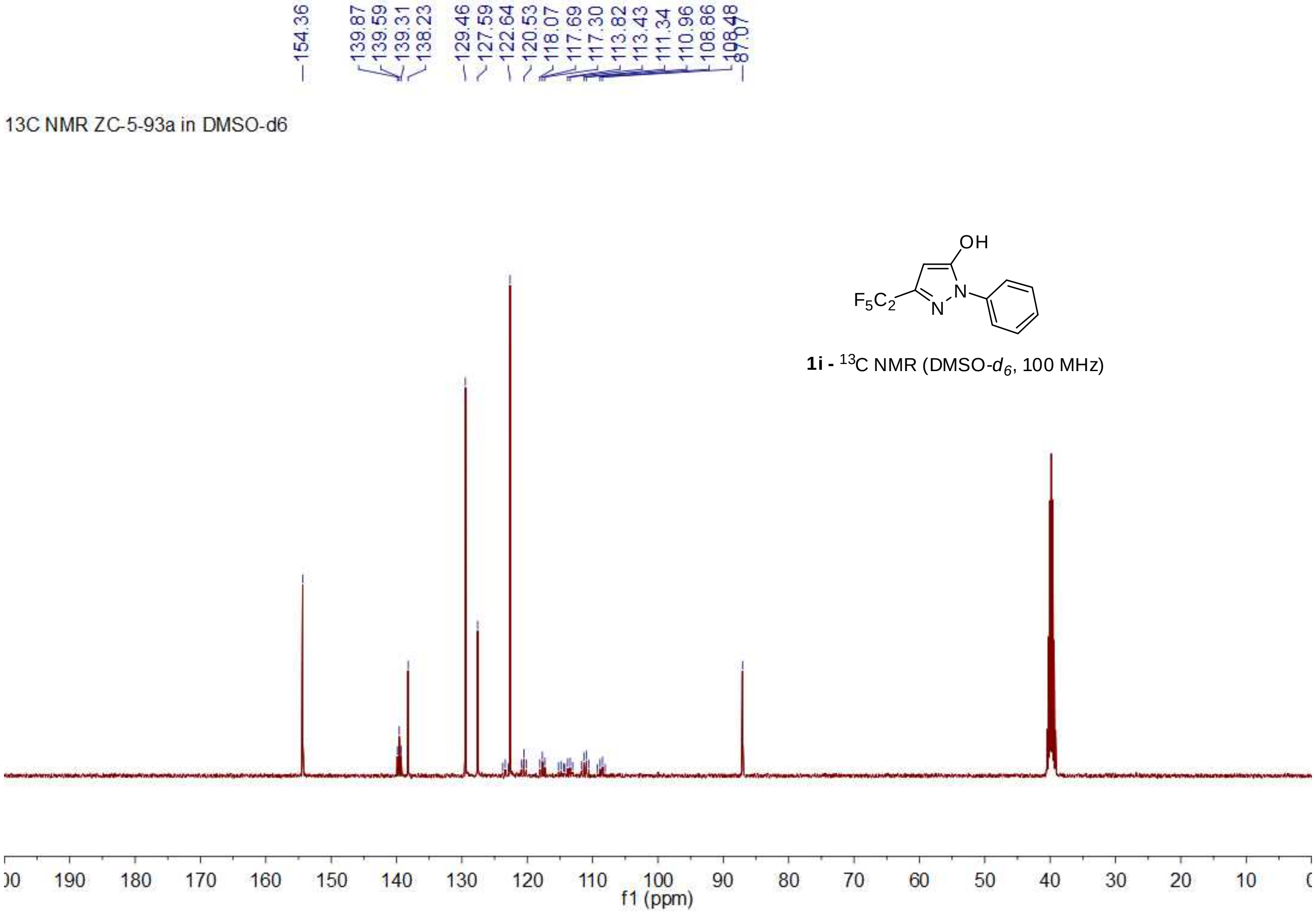

1i - ^{13}C NMR (DMSO-d_6, 100 MHz)

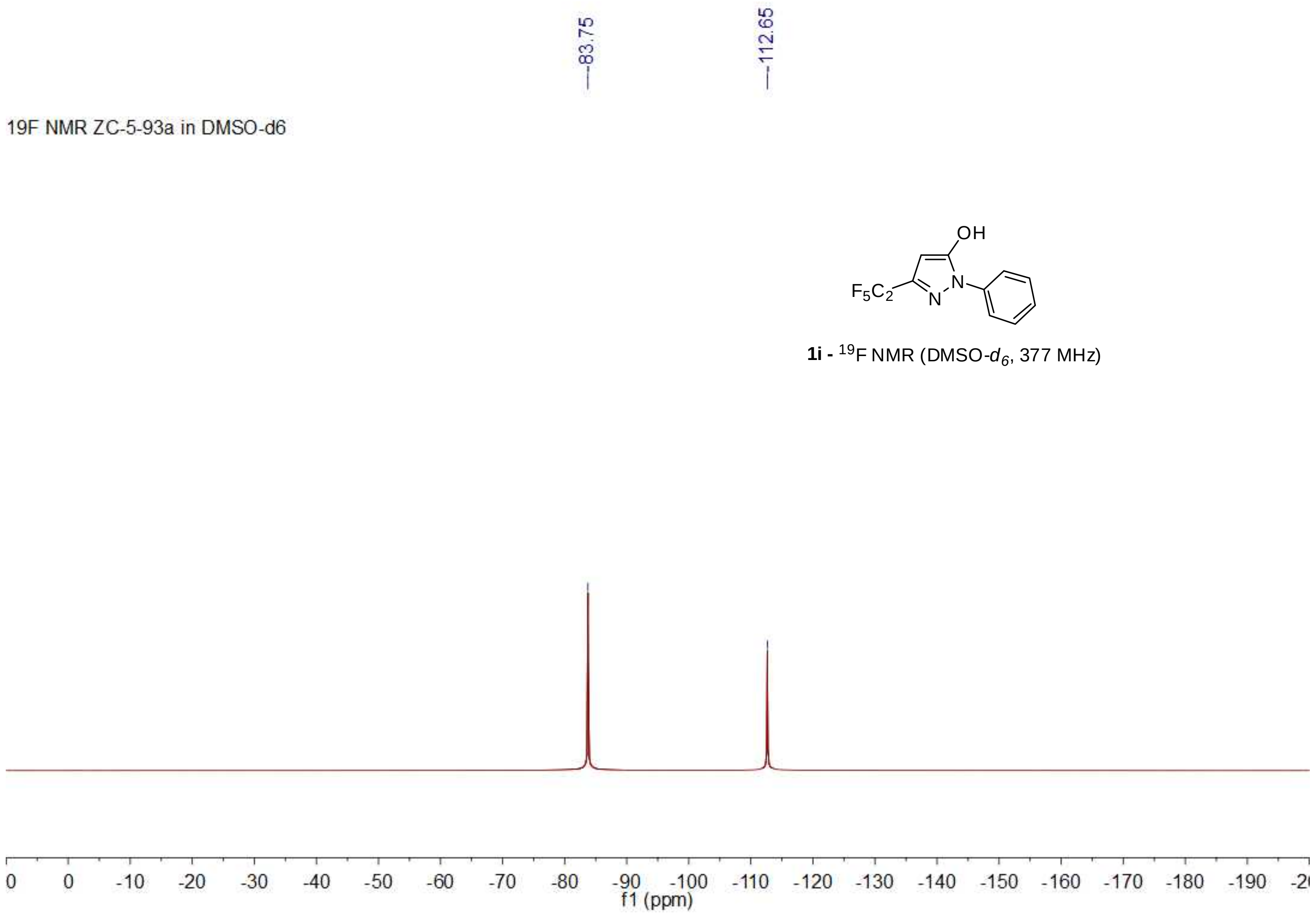

1i - ^{19}F NMR (DMSO-d_6, 377 MHz)

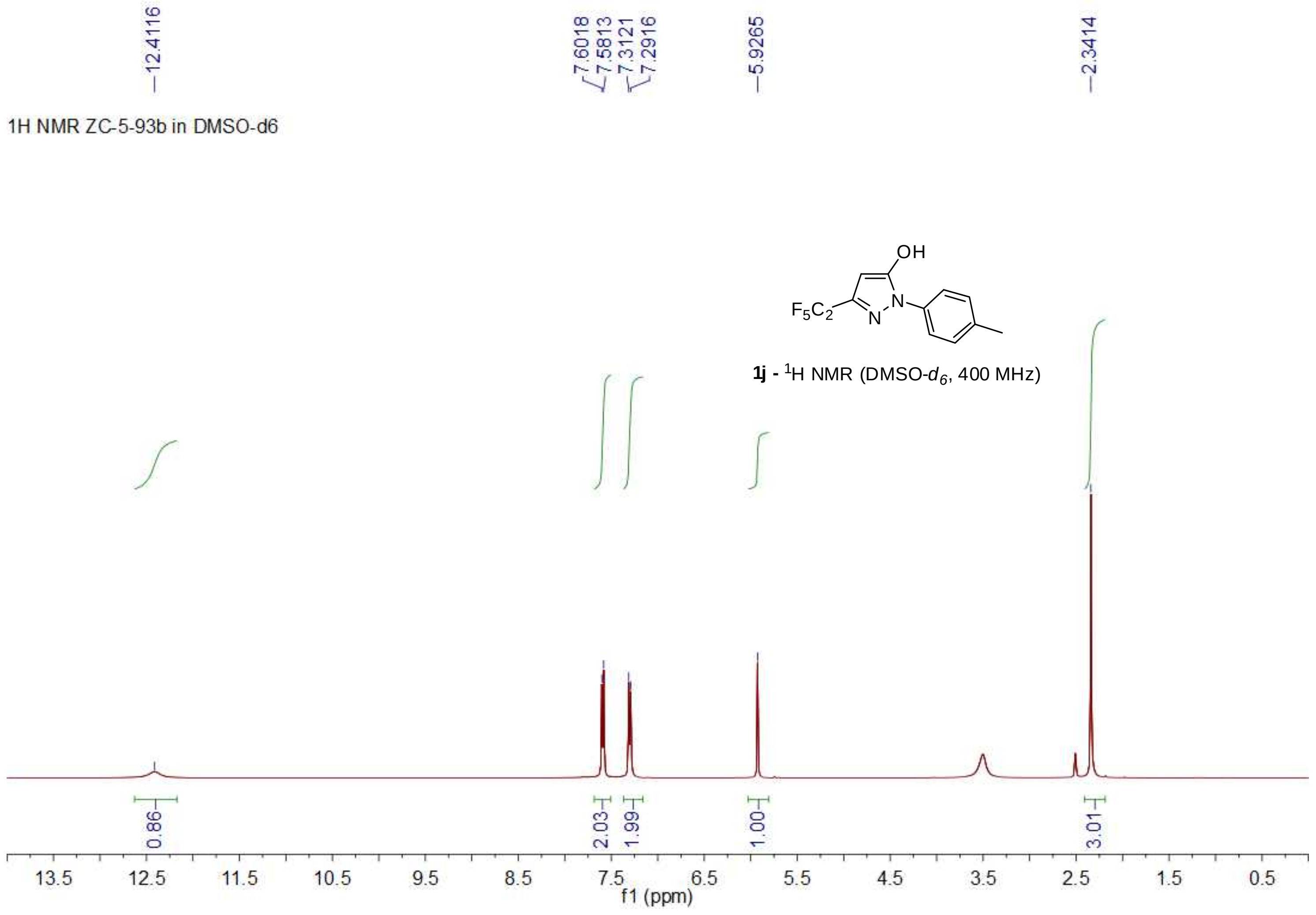
12.4116
7.6018
7.5813
7.3121
7.2916
5.9265
2.3414
1H NMR ZC-5-93b in DMSO-d6
OH
F5C2
N
N
1j - 1H NMR (DMSO-d6, 400 MHz)
0.86
2.03
1.99
1.00
3.01
13.5
12.5
11.5
10.5
9.5
8.5
7.5
6.5
5.5
4.5
3.5
2.5
1.5
0.5
f1 (ppm)

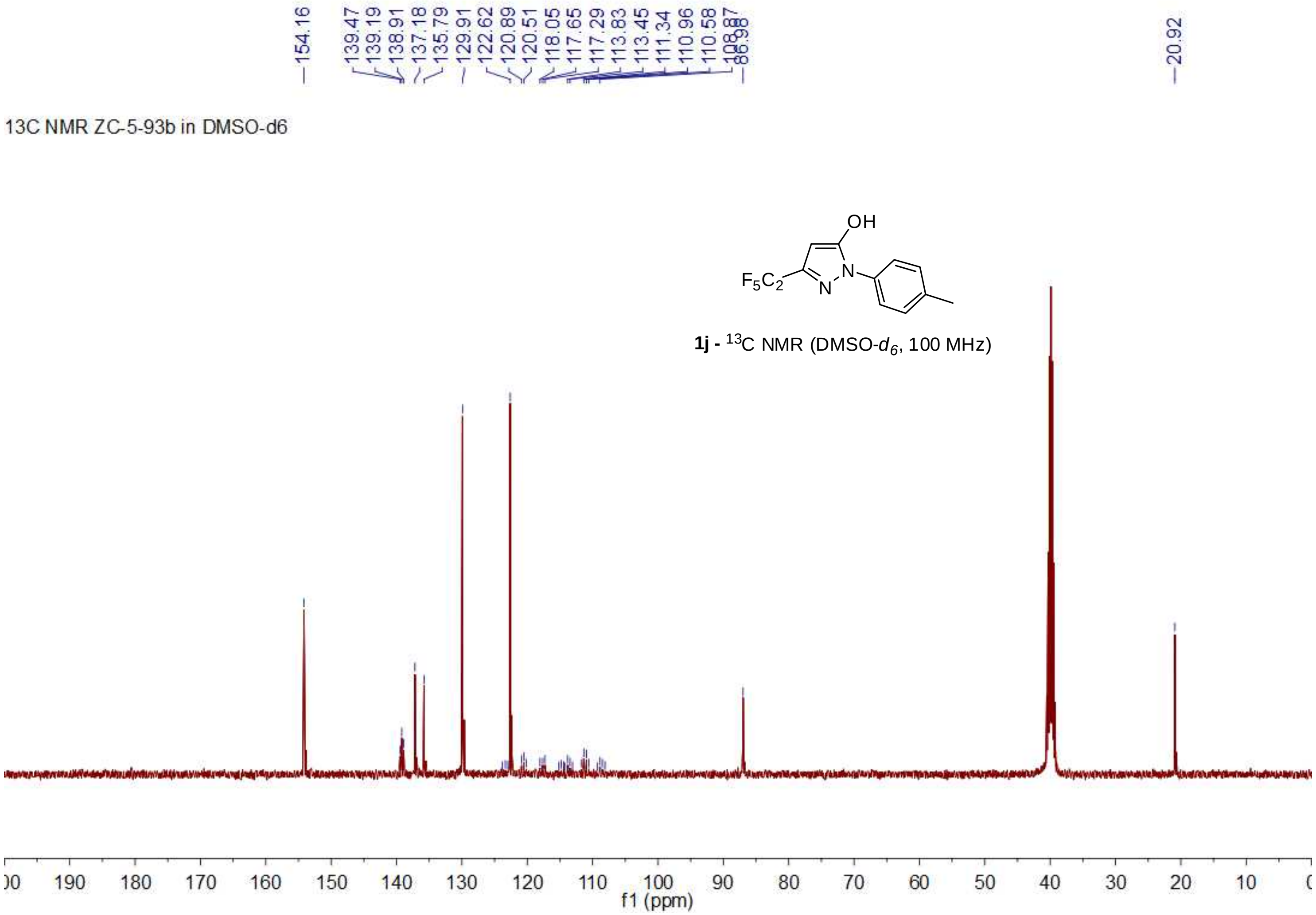
154.16
139.47
139.19
138.91
137.18
135.79
129.91
122.62
120.89
120.51
118.05
117.65
117.29
113.83
113.45
111.34
110.96
110.58
108.87
86.98
20.92
13C NMR ZC-5-93b in DMSO-d6
OH
N
N
F5C2
1j - 13C NMR (DMSO-d6, 100 MHz)
f1 (ppm)

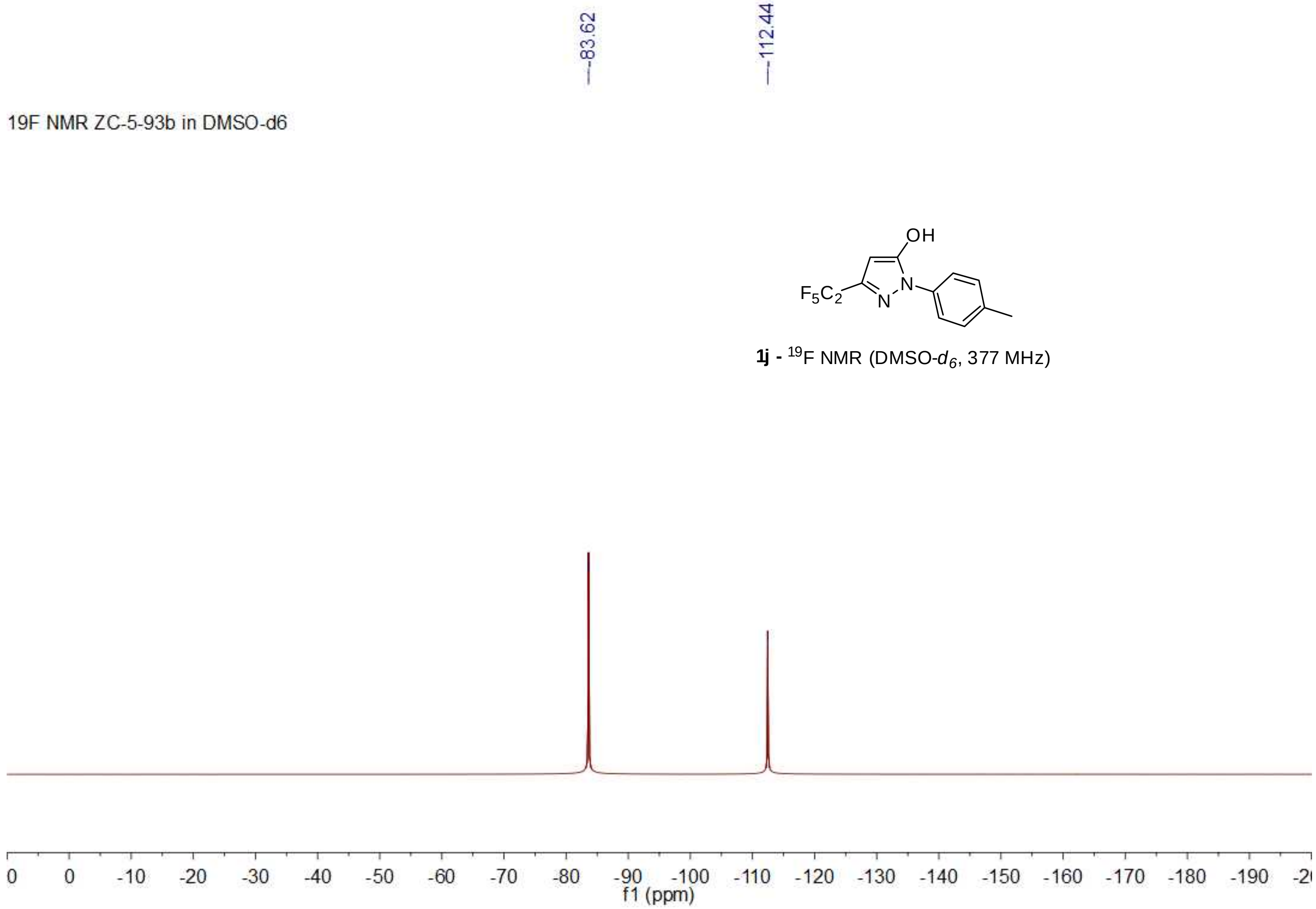

1j - ^{19}F NMR (DMSO-d_6, 377 MHz)

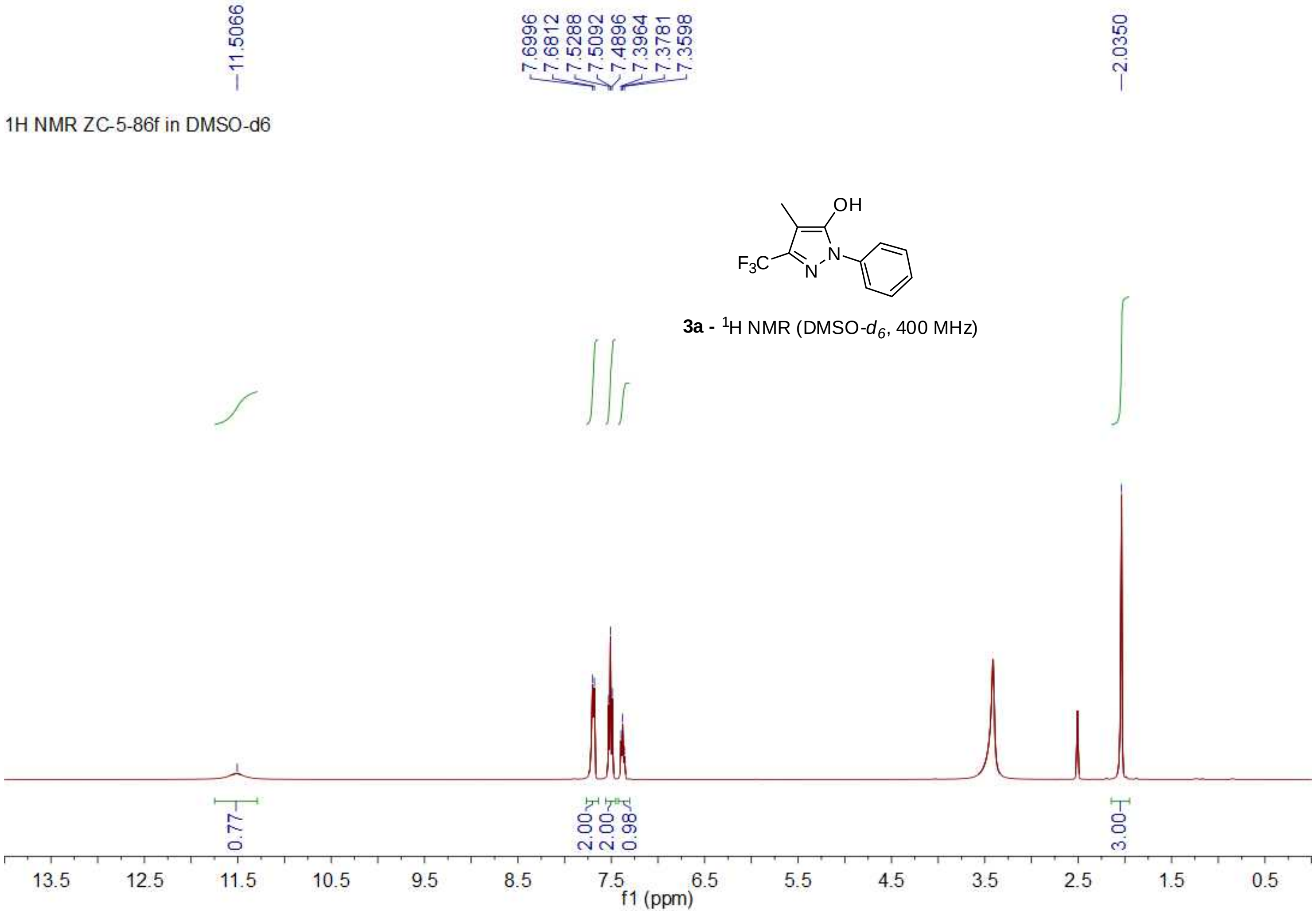

3a - ^{1}H NMR (DMSO-d_6, 400 MHz)

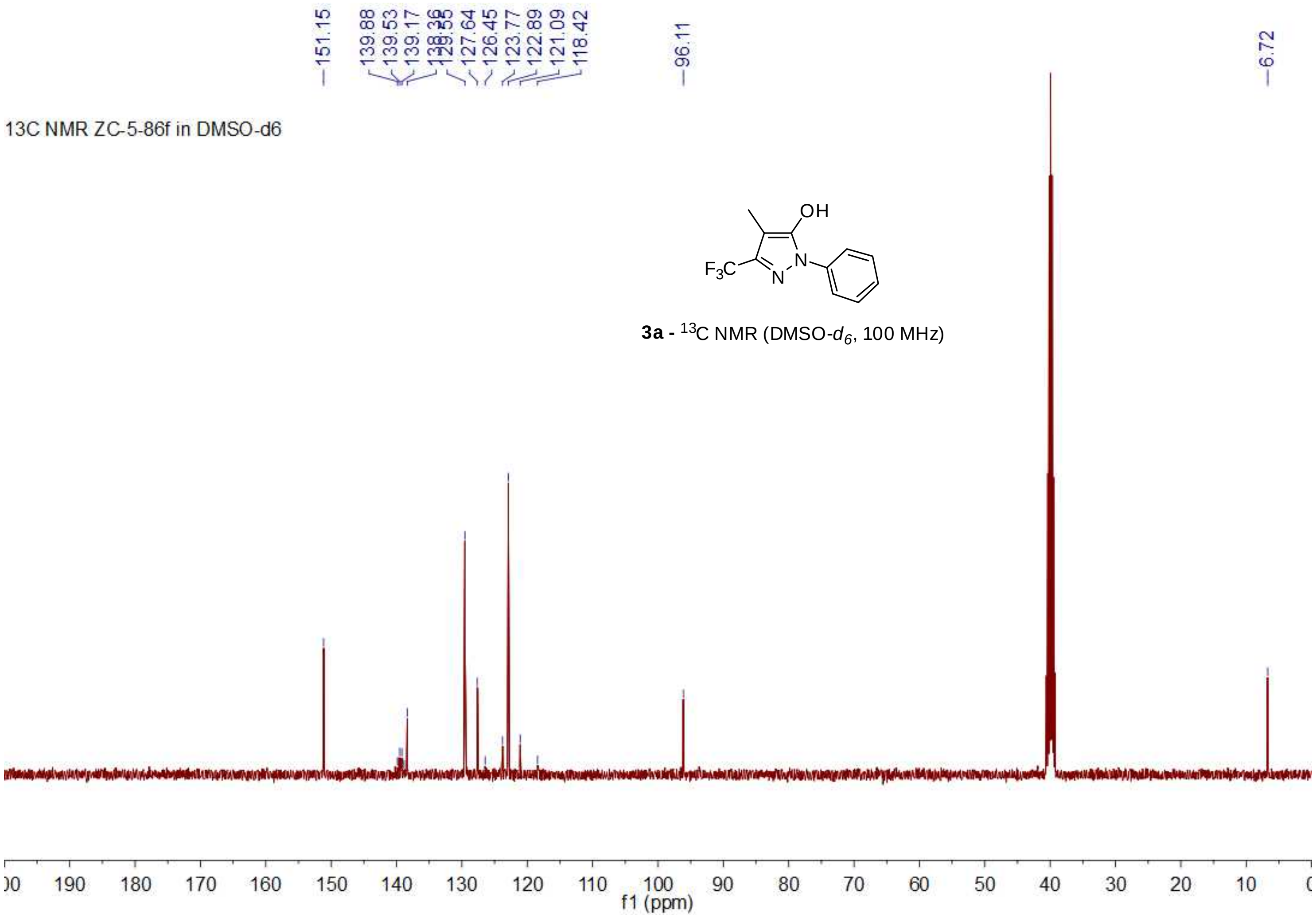

3a - ^{13}C NMR (DMSO-d_6, 100 MHz)

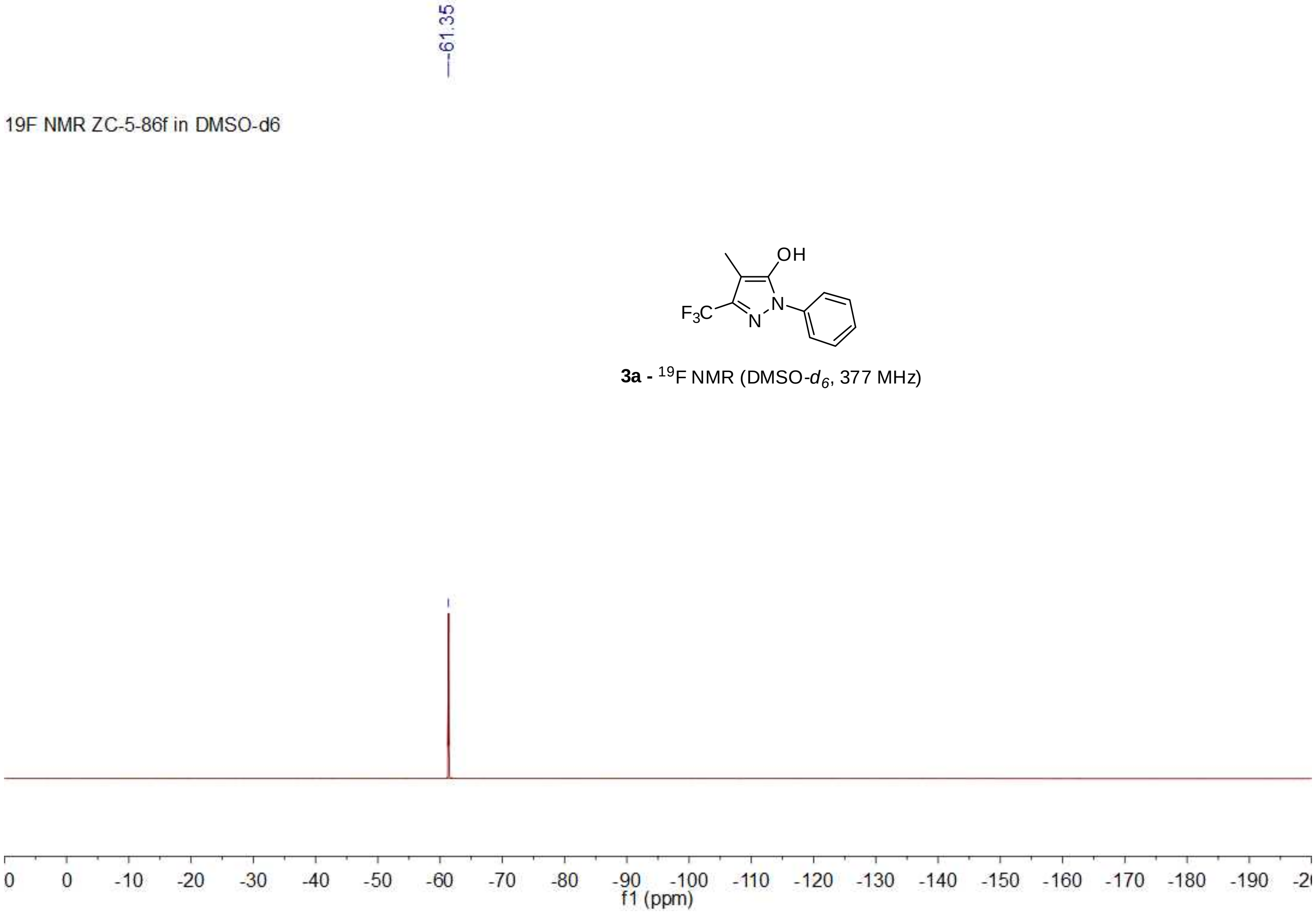

3a - ^{19}F NMR (DMSO-d_6, 377 MHz)

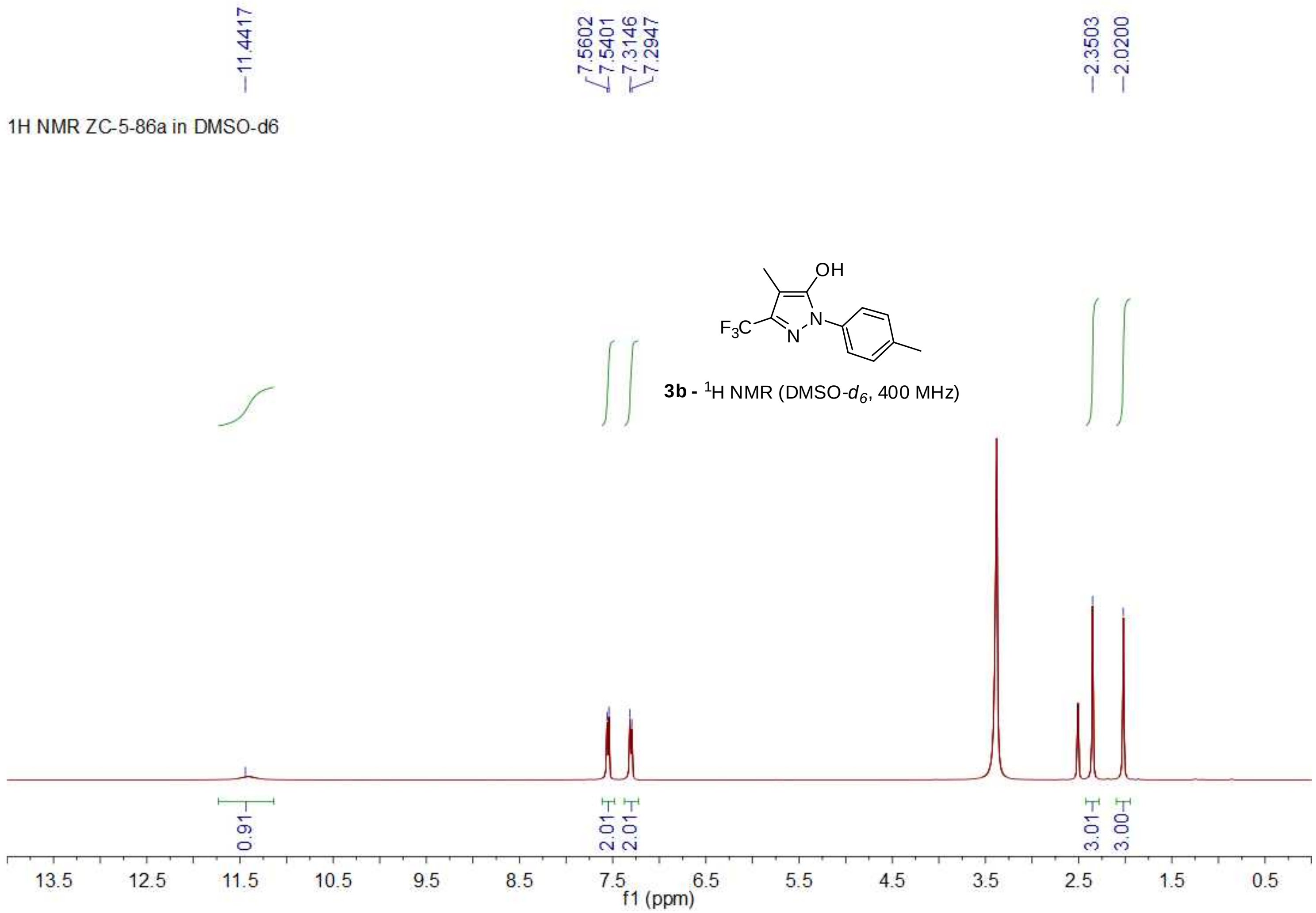
1H NMR ZC-5-86a in DMSO-d6
11.4417
7.5602
7.5401
7.3146
7.2947
2.3503
2.0200
OH
N
N
F3C
3b - 1H NMR (DMSO-d6, 400 MHz)
0.91
2.01
2.01
3.01
3.00
13.5
12.5
11.5
10.5
9.5
8.5
7.5
6.5
5.5
4.5
3.5
2.5
1.5
0.5
f1 (ppm)

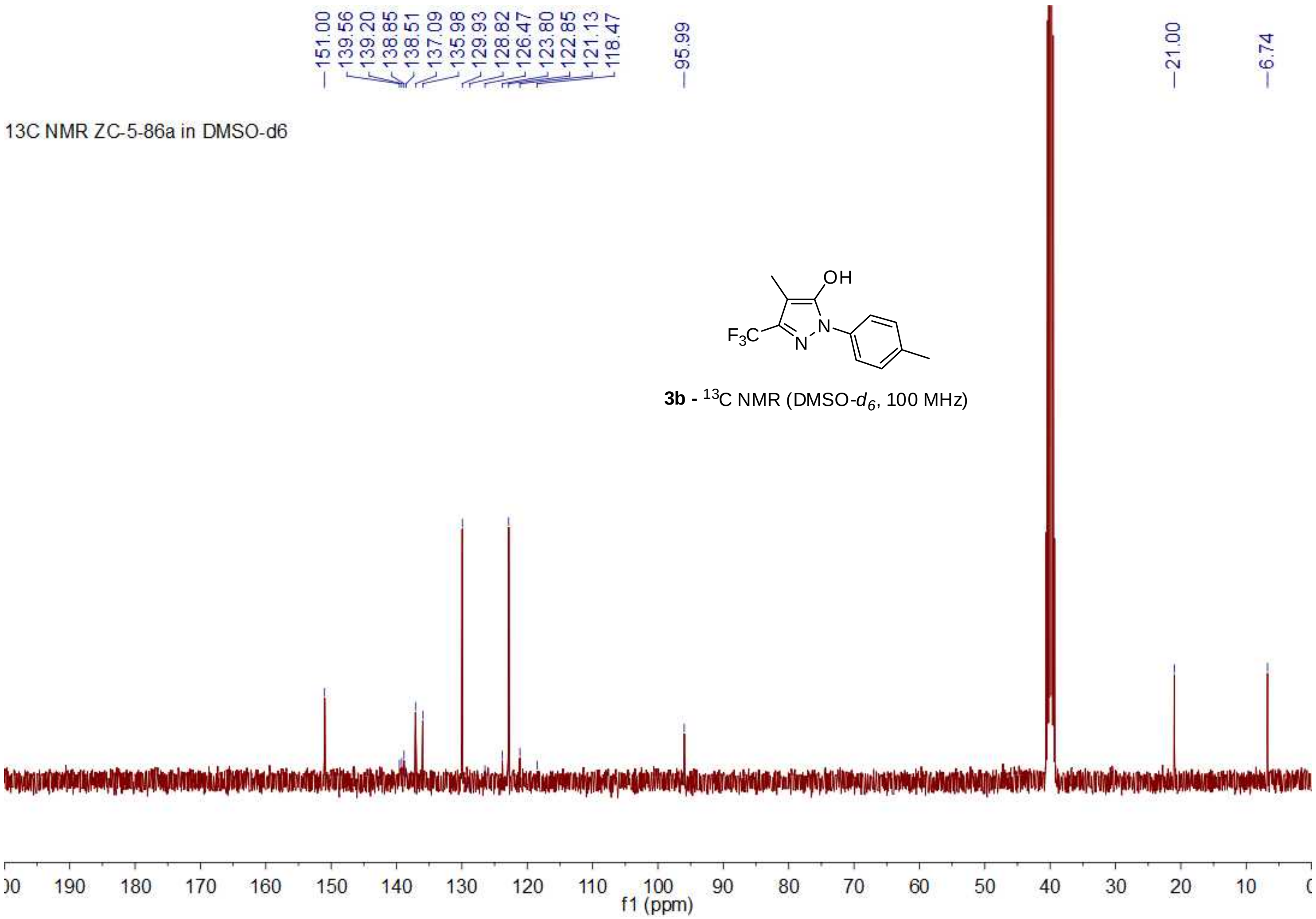
151.00
139.56
139.20
138.85
138.51
137.09
135.98
129.93
128.82
126.47
123.80
122.85
121.13
118.47
95.99
21.00
6.74
13C NMR ZC-5-86a in DMSO-d6
OH
N
N
F3C
3b - 13C NMR (DMSO-d6, 100 MHz)
190
180
170
160
150
140
130
120
110
100
90
80
70
60
50
40
30
20
10
f1 (ppm)

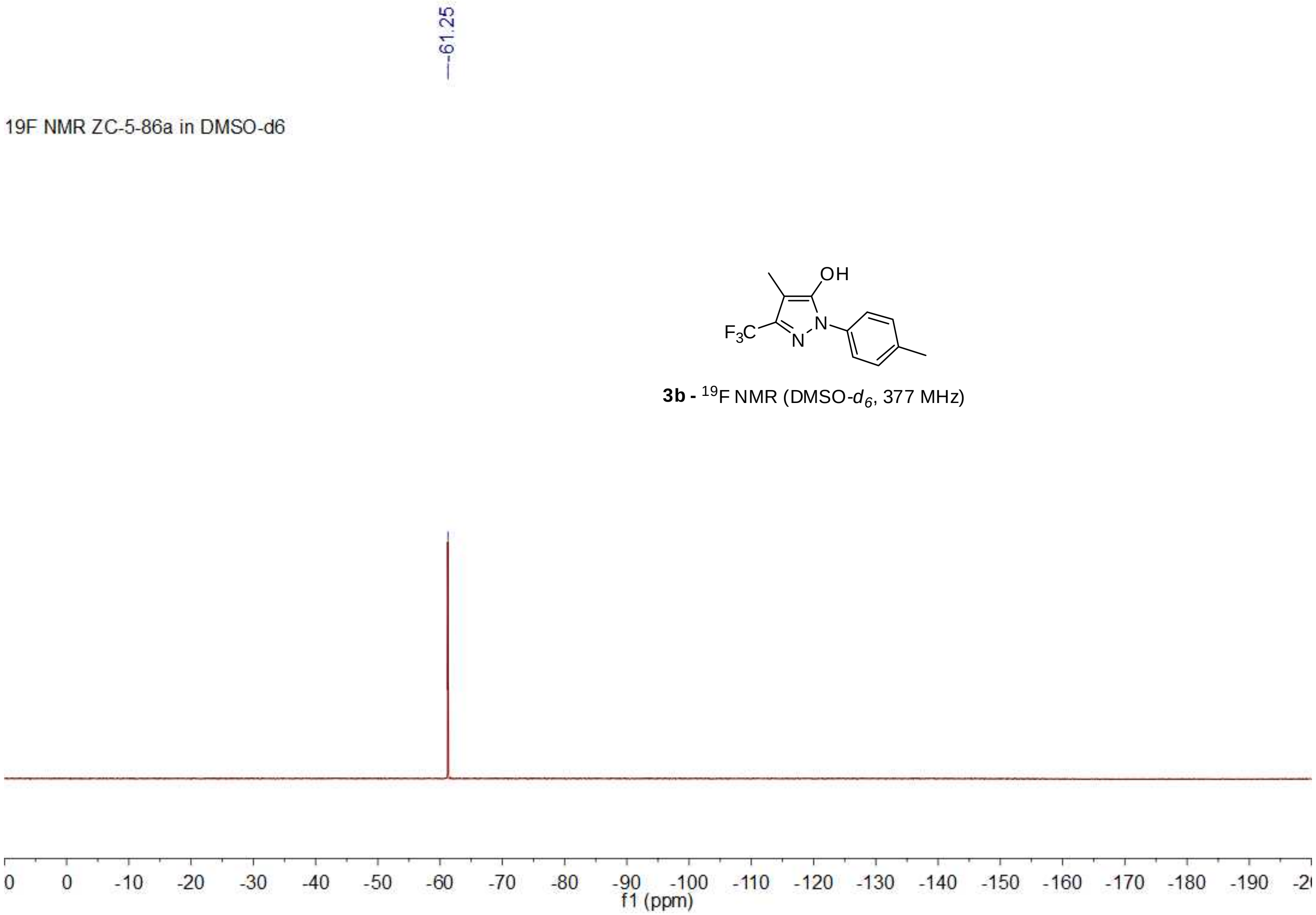

3b - ^{19}F NMR (DMSO-d_6, 377 MHz)

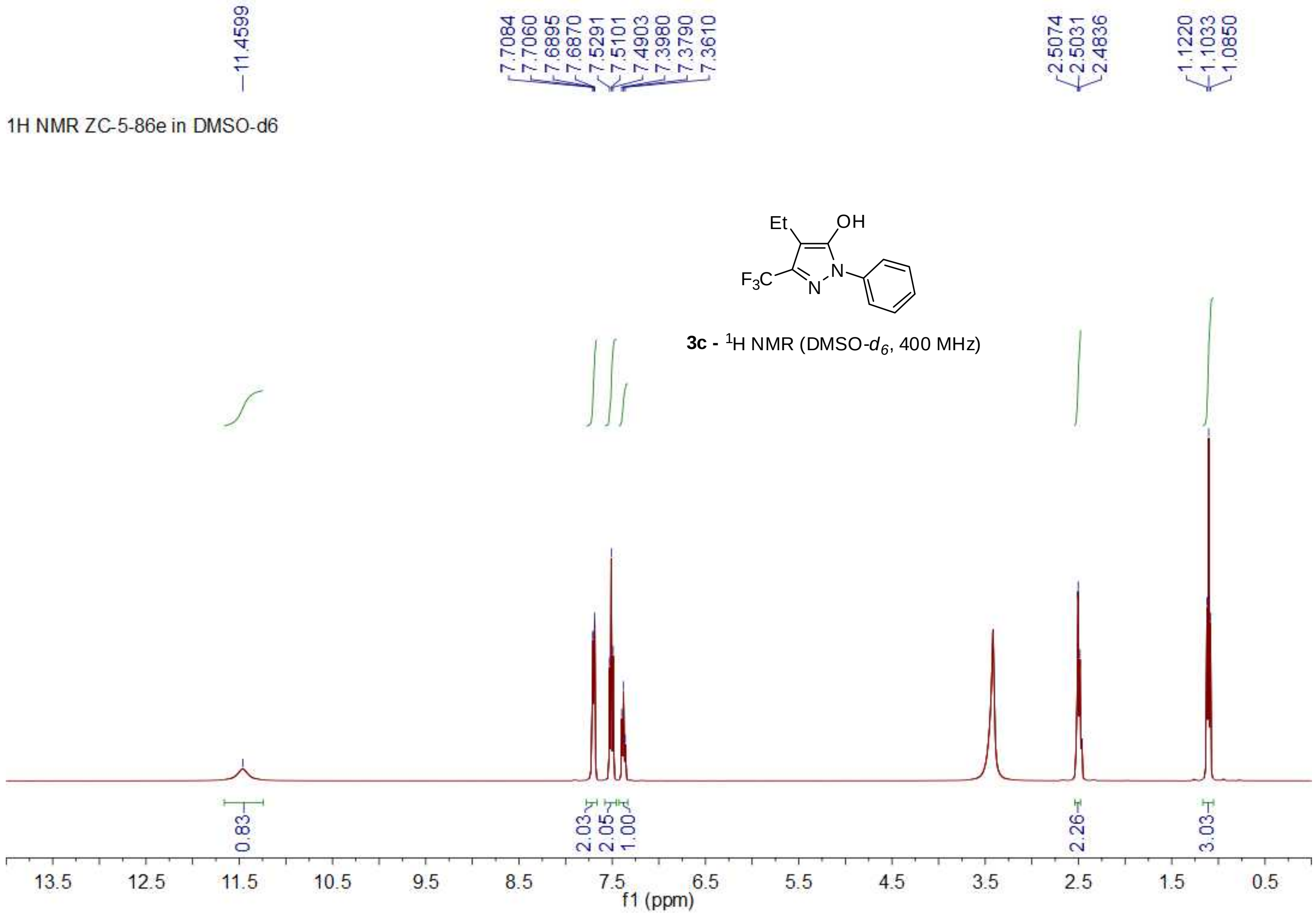

3c - ^{1}H NMR (DMSO-d_6, 400 MHz)

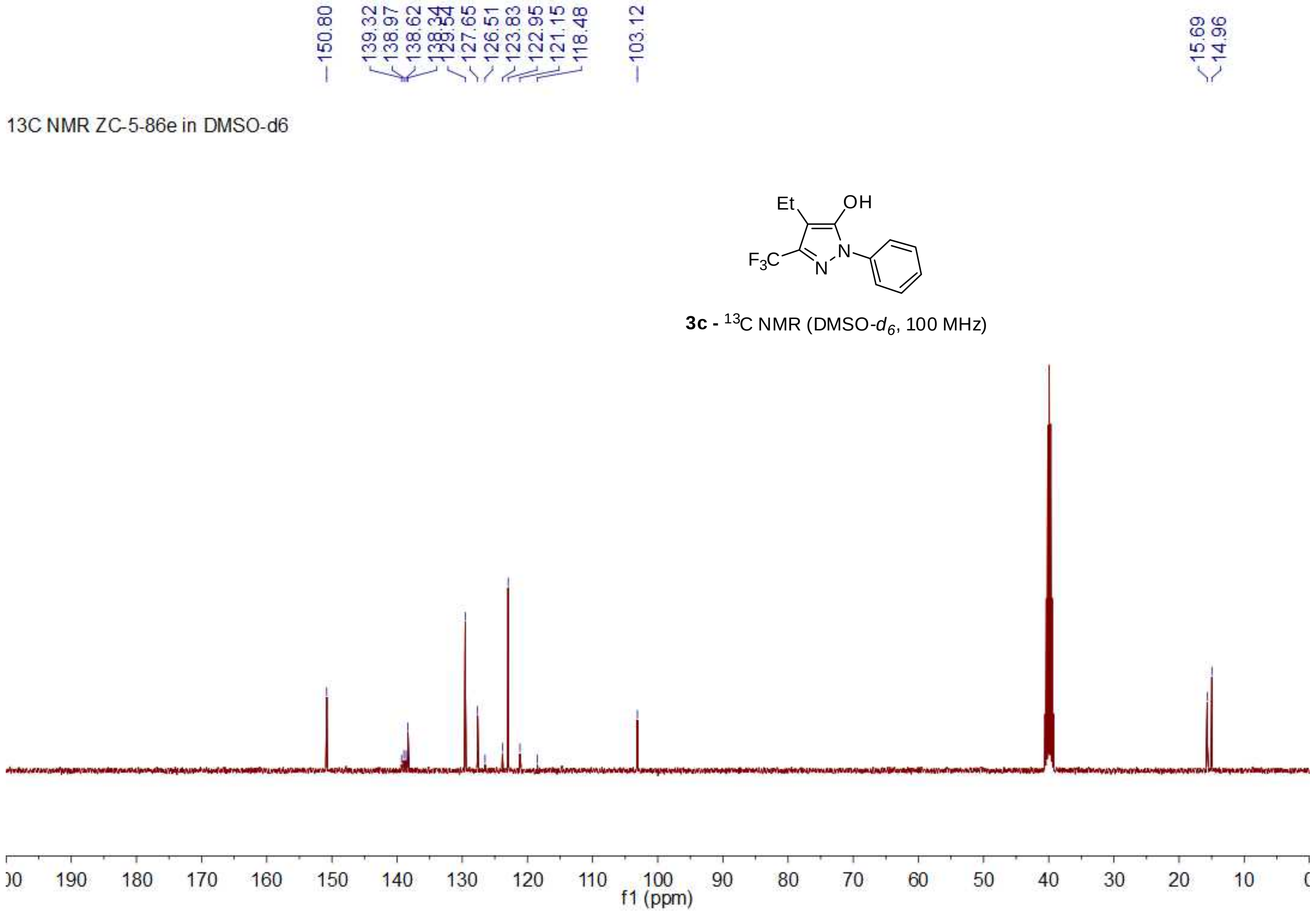
150.80
139.32
138.97
138.62
138.34
129.54
127.65
126.51
123.83
122.95
121.15
118.48
103.12
15.69
14.96
13C NMR ZC-5-86e in DMSO-d6
Et
OH
F3C
N
N
3c - 13C NMR (DMSO-d6, 100 MHz)
f1 (ppm)

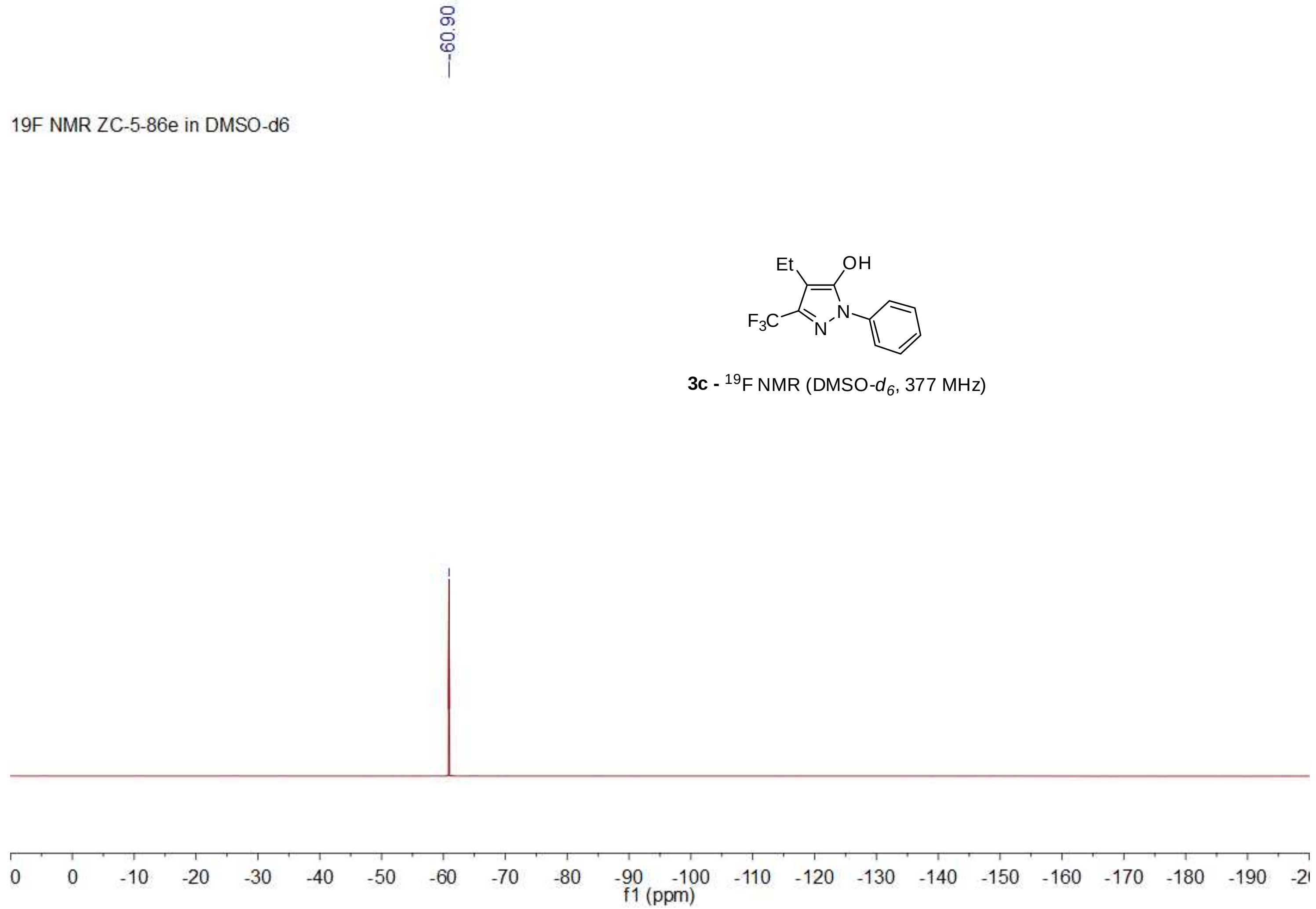

3c - ^{19}F NMR (DMSO-d_6, 377 MHz)

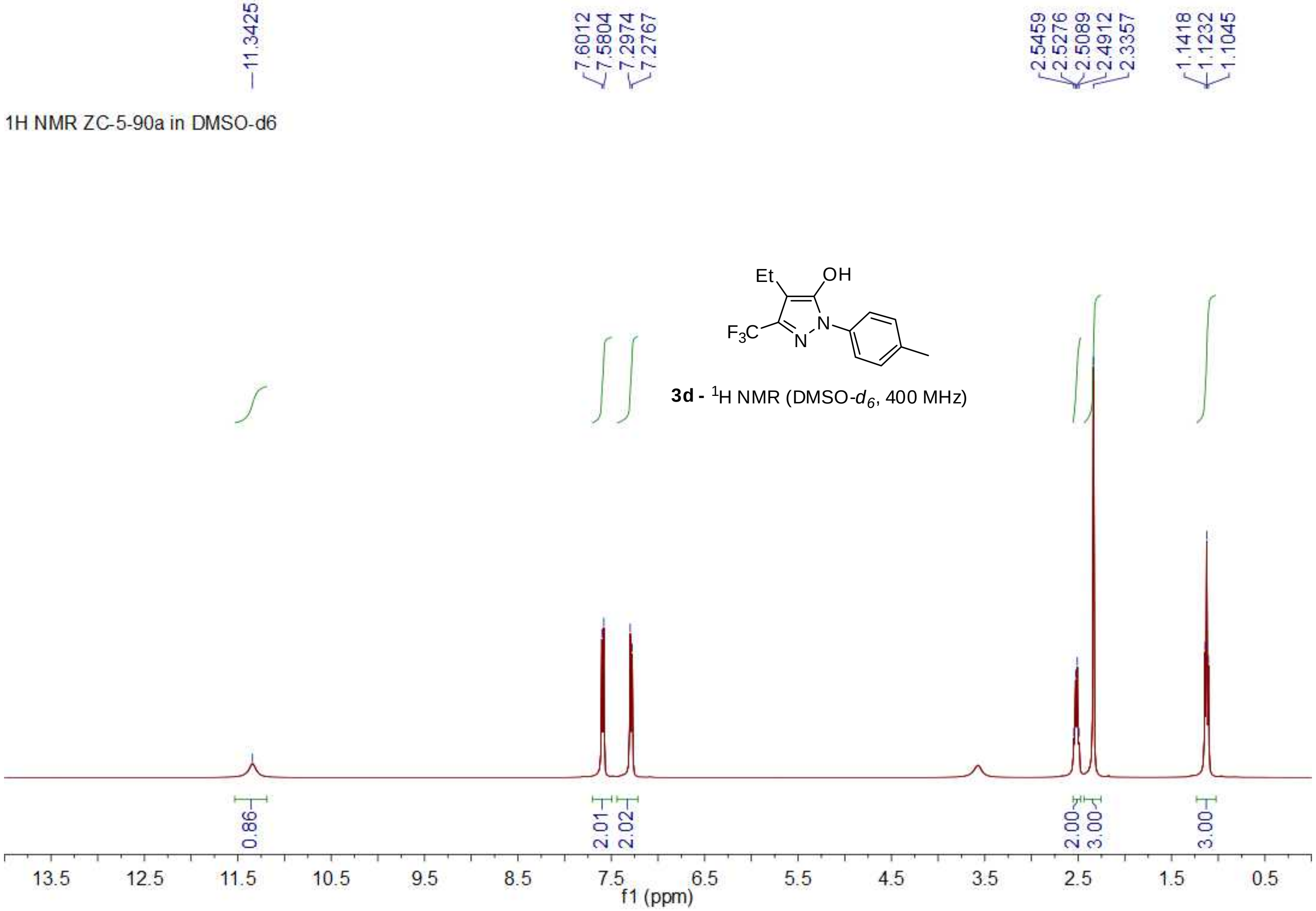

1H NMR ZC-5-90a in DMSO-d6
11.3425
7.6012
7.5804
7.2974
7.2767
2.5459
2.5276
2.5089
2.4912
2.3357
1.1418
1.1232
1.1045
Et
OH
F_3C
N
N
3d - ^{1}H NMR (DMSO-d_6, 400 MHz)
0.86
2.01
2.02
2.00
3.00
3.00
13.5
12.5
11.5
10.5
9.5
8.5
7.5
6.5
5.5
4.5
3.5
2.5
1.5
0.5
f1 (ppm)

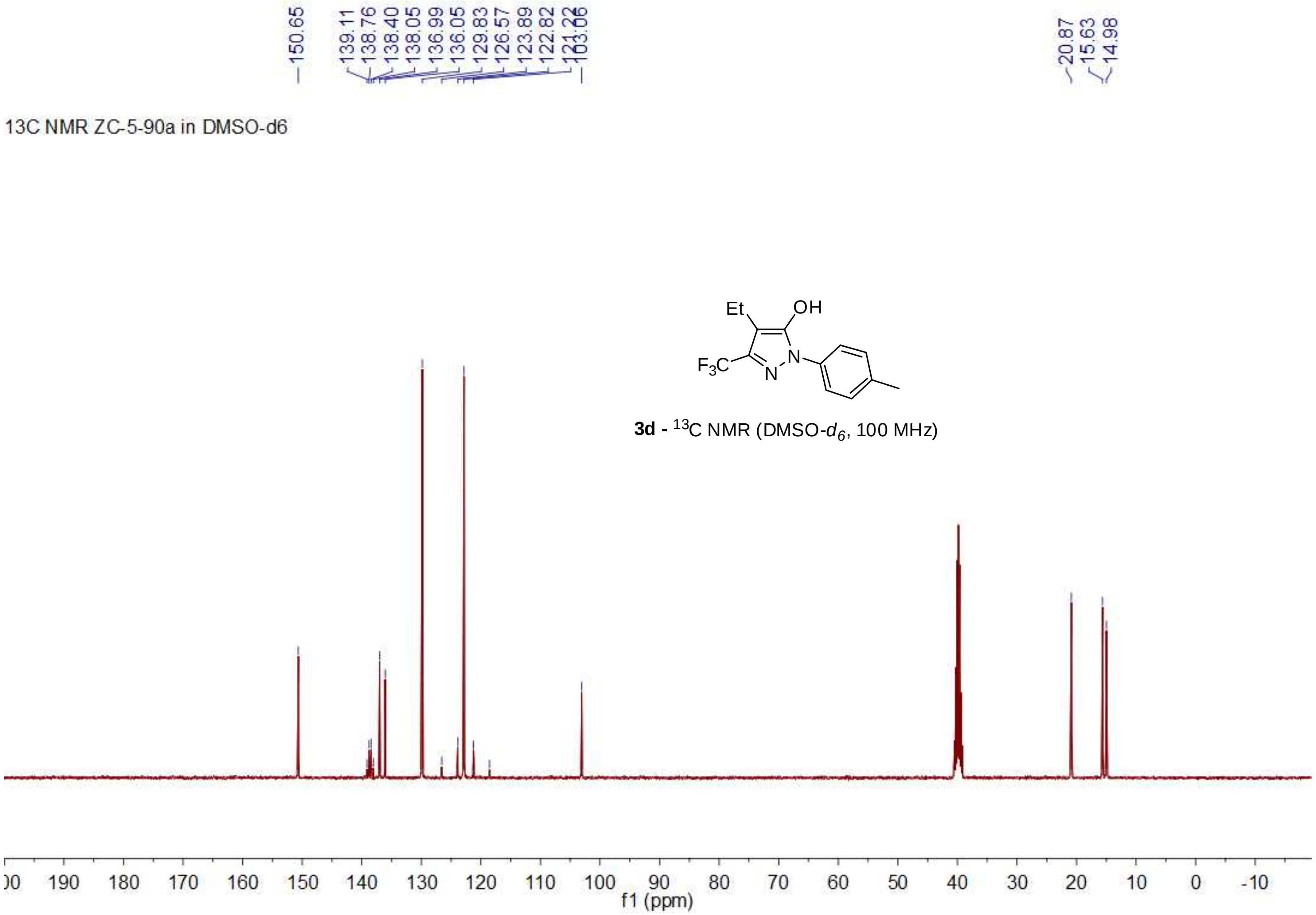

3d - ^{13}C NMR (DMSO-d_6, 100 MHz)

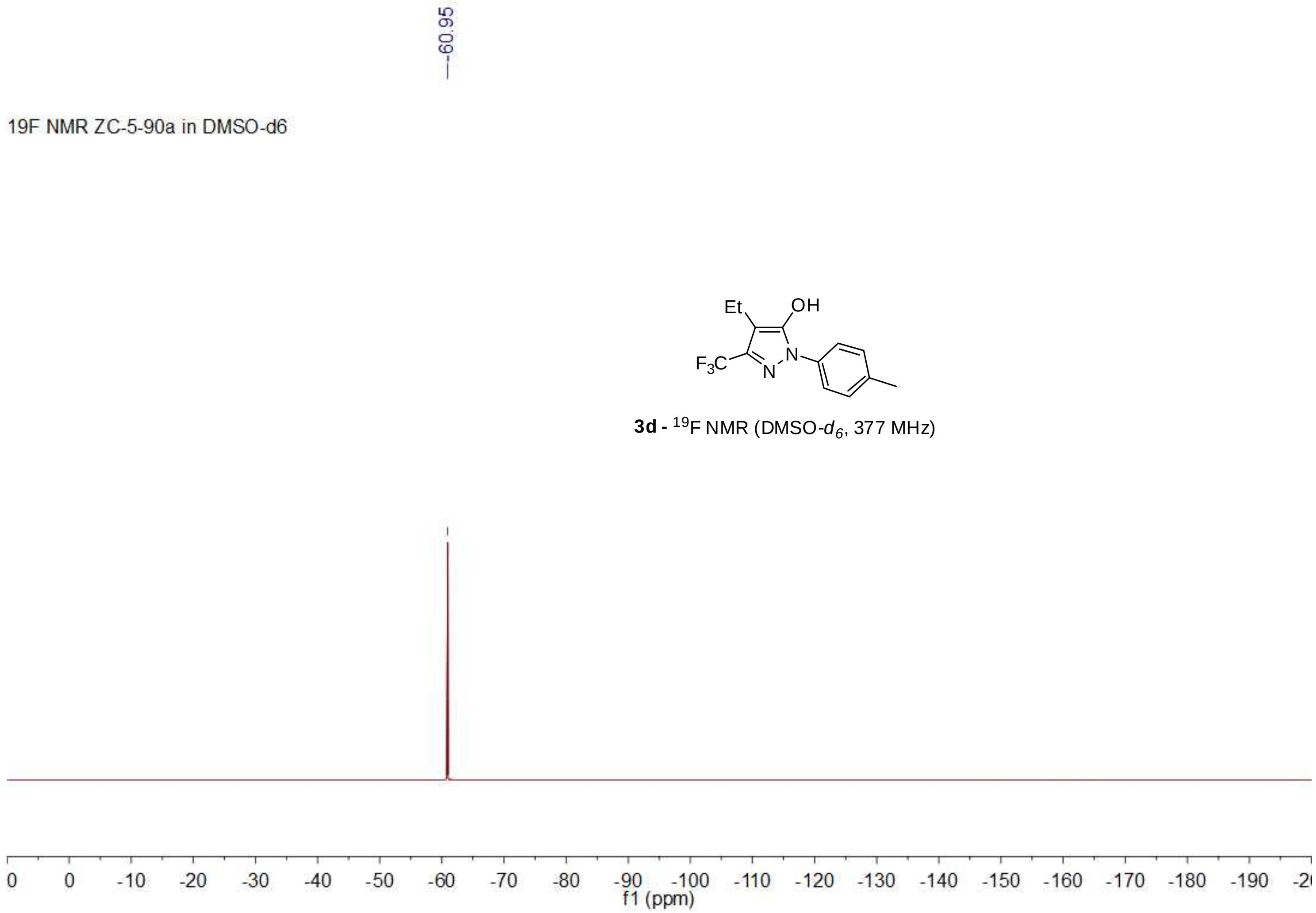

3d - ^{19}F NMR (DMSO-d_6, 377 MHz)

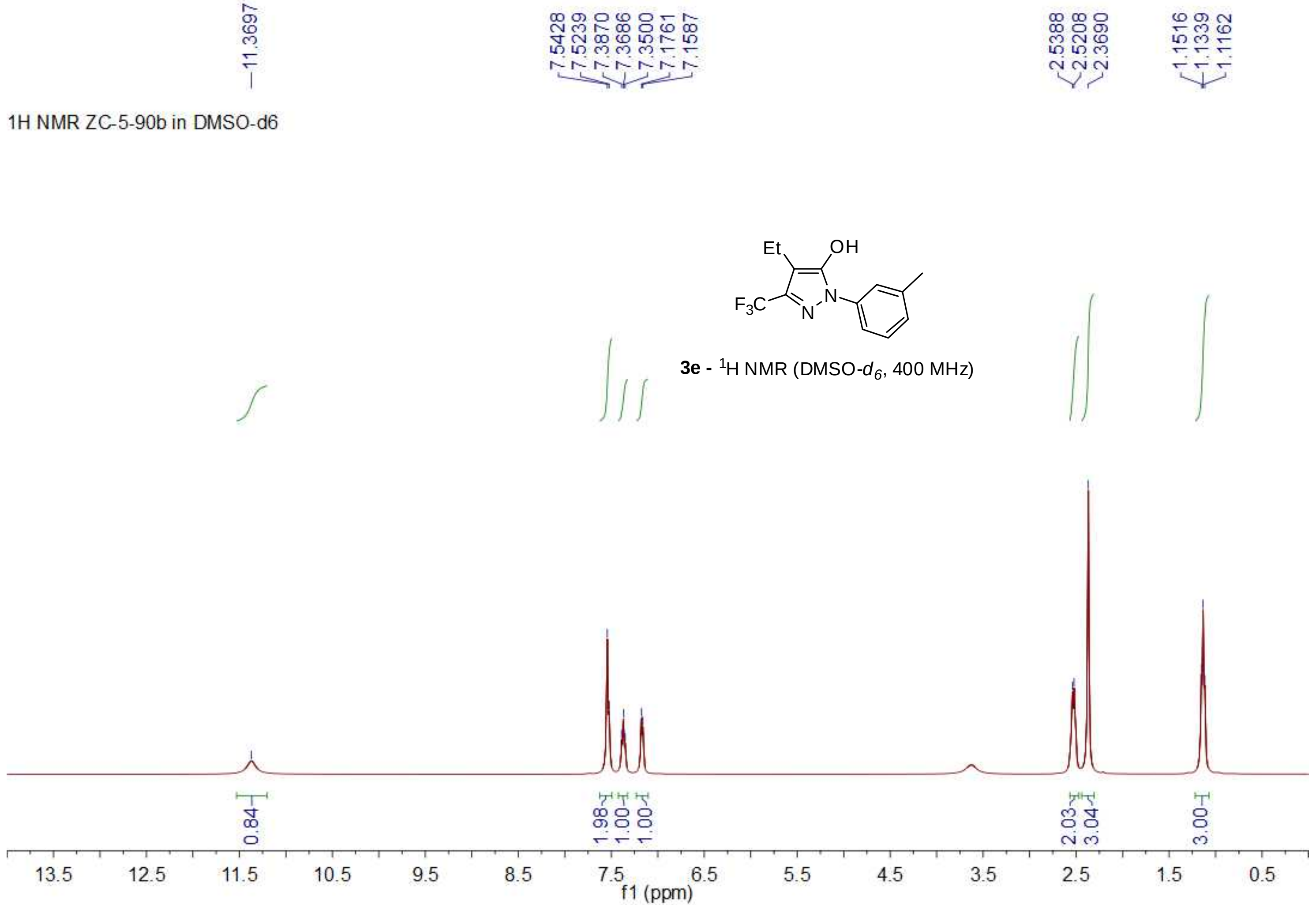

3e - ^{1}H NMR (DMSO-d_6, 400 MHz)

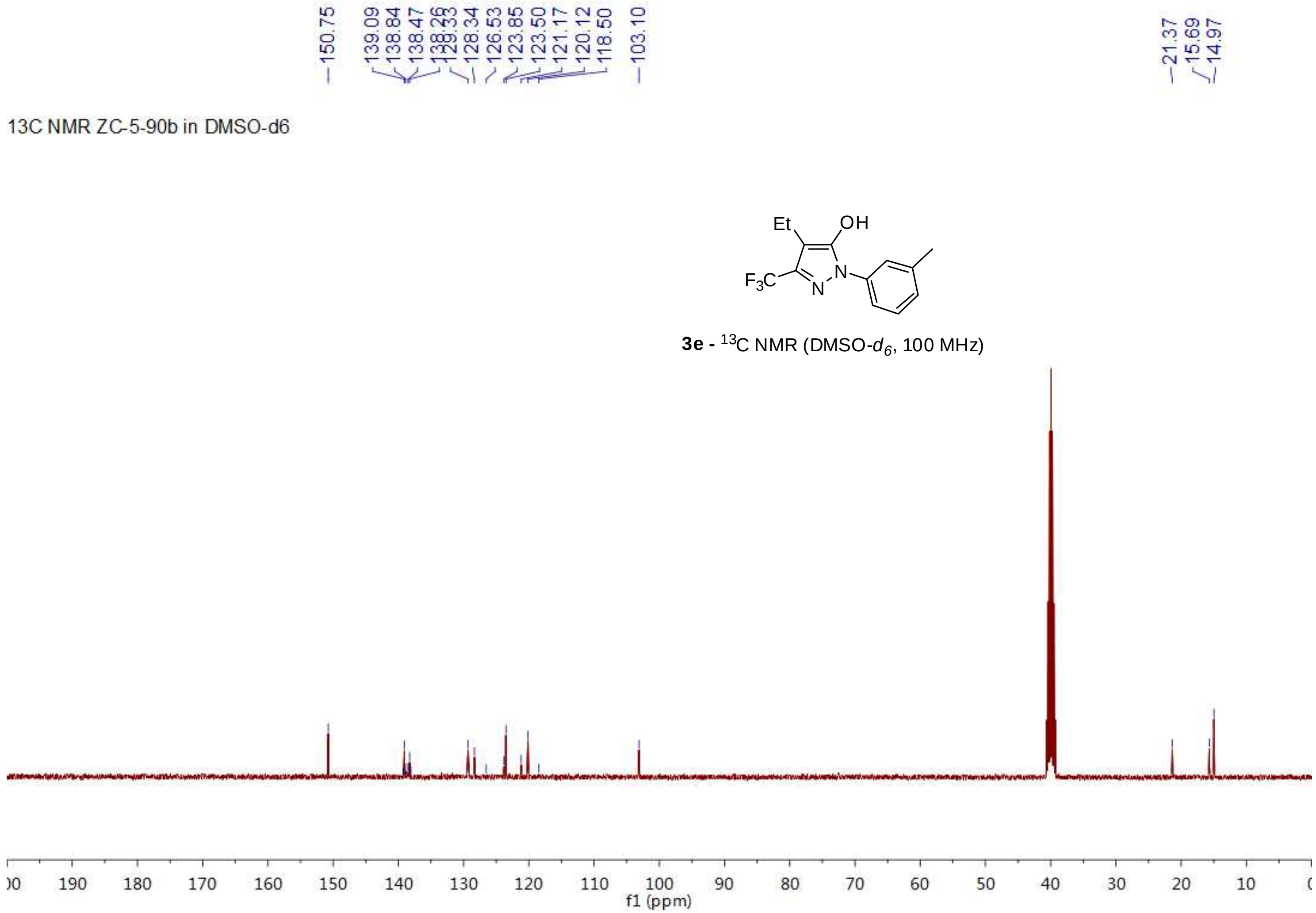
150.75
139.09
138.84
138.47
138.26
129.33
128.34
126.53
123.85
123.50
121.17
120.12
118.50
103.10
21.37
15.69
14.97
13C NMR ZC-5-90b in DMSO-d6
Et
OH
F_3C
N
N
3e - ^{13}C NMR (DMSO-d_6, 100 MHz)
f1 (ppm)

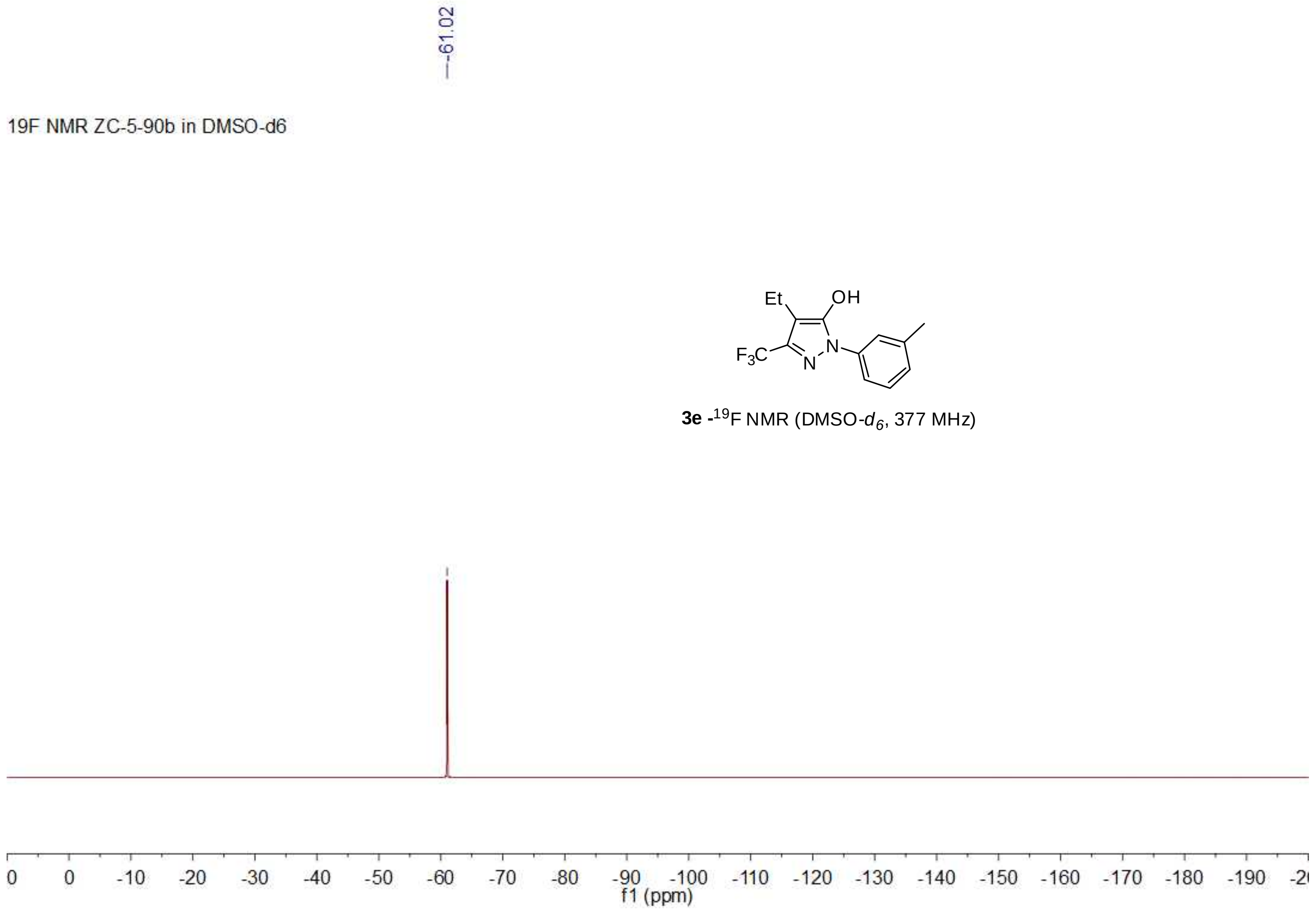

3e -^{19}F NMR (DMSO-d_6, 377 MHz)

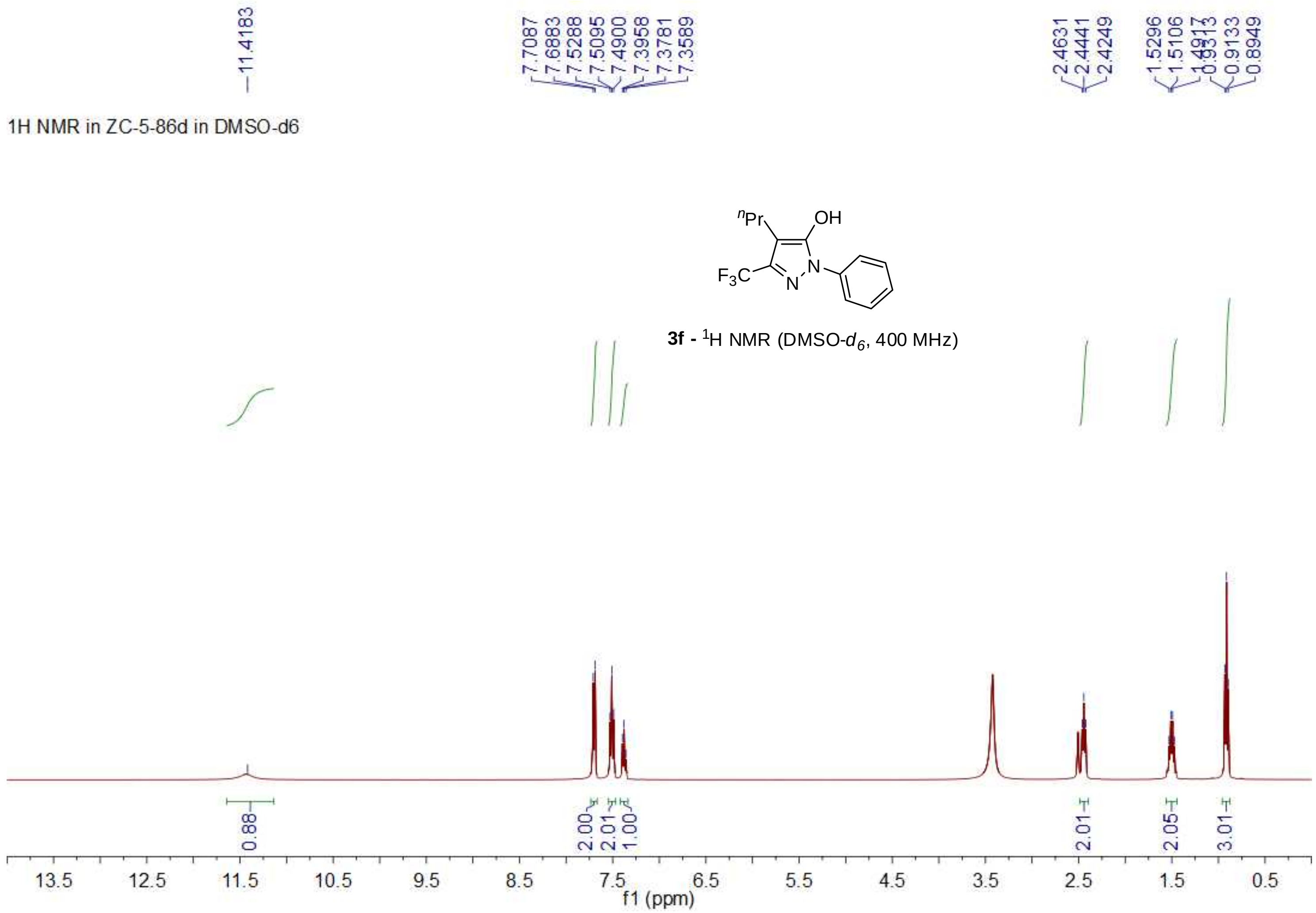

3f - ^{1}H NMR (DMSO-d_6, 400 MHz)

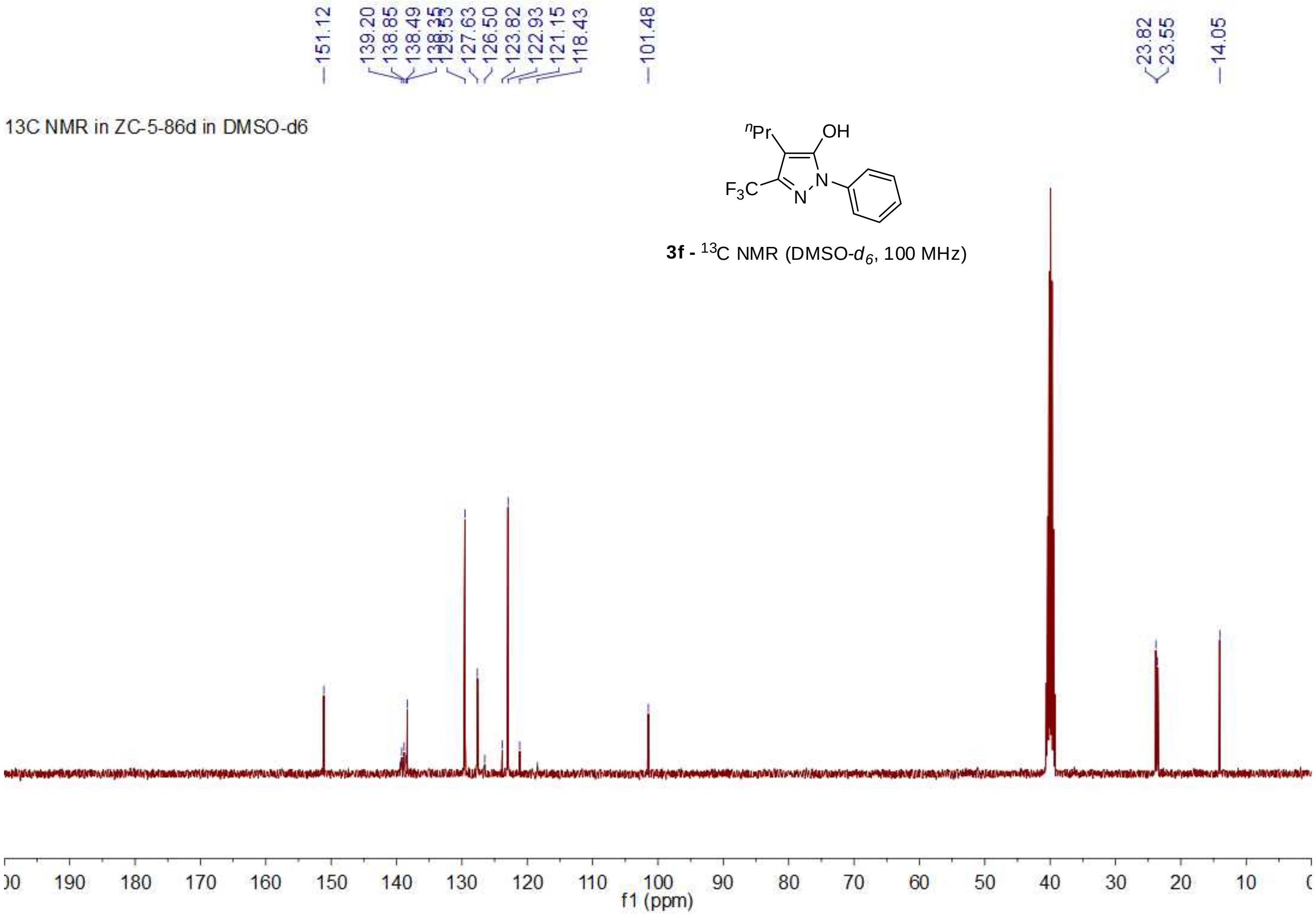
13C NMR in ZC-5-86d in DMSO-d6
151.12
139.20
138.85
138.49
138.35
129.53
127.63
126.50
123.82
122.93
121.15
118.43
101.48
23.82
23.55
14.05
^{n}Pr
OH
F_3C
N
N
3f - ^{13}C NMR (DMSO-d_6, 100 MHz)
f1 (ppm)

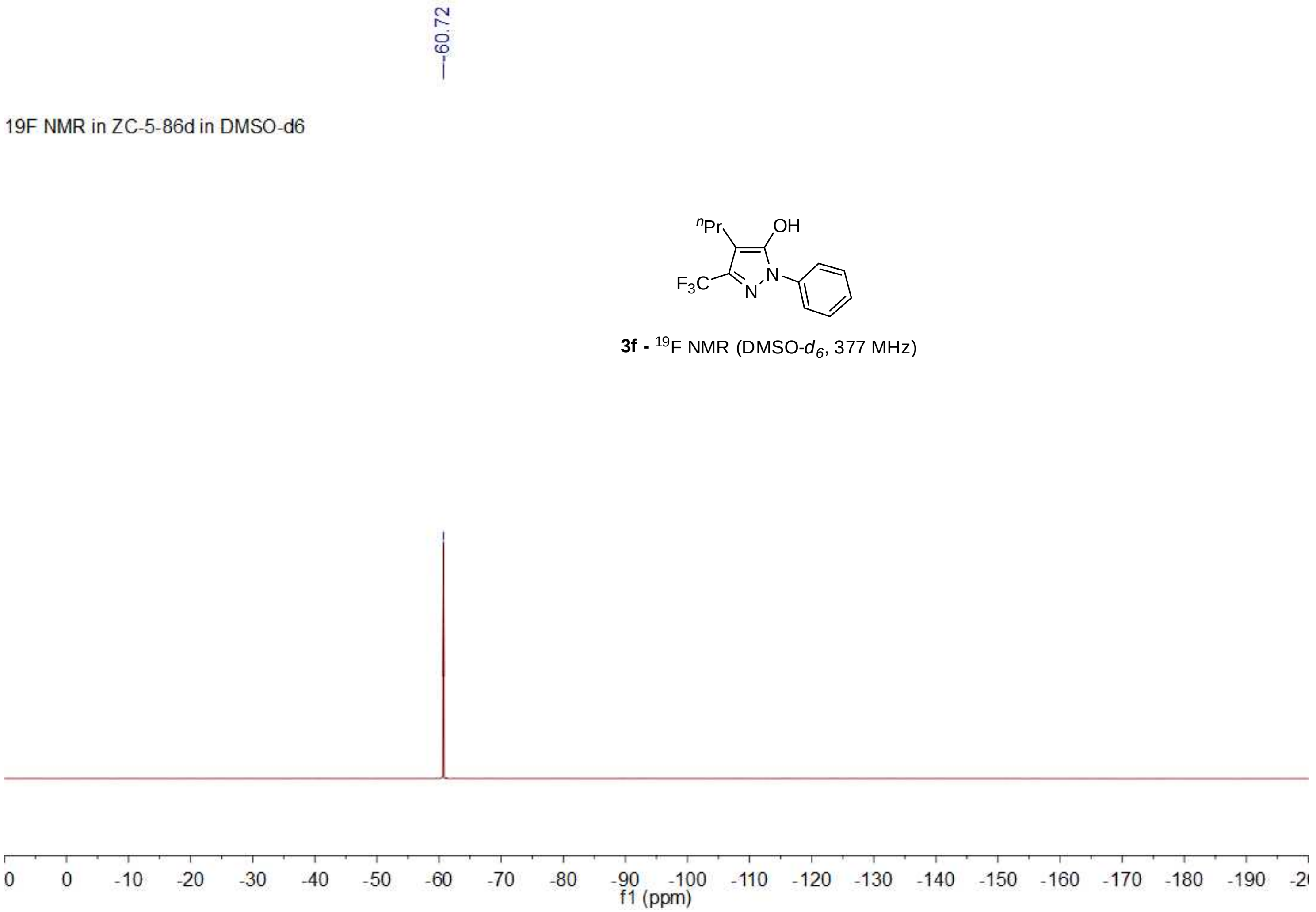

3f - ^{19}F NMR (DMSO-d_6, 377 MHz)

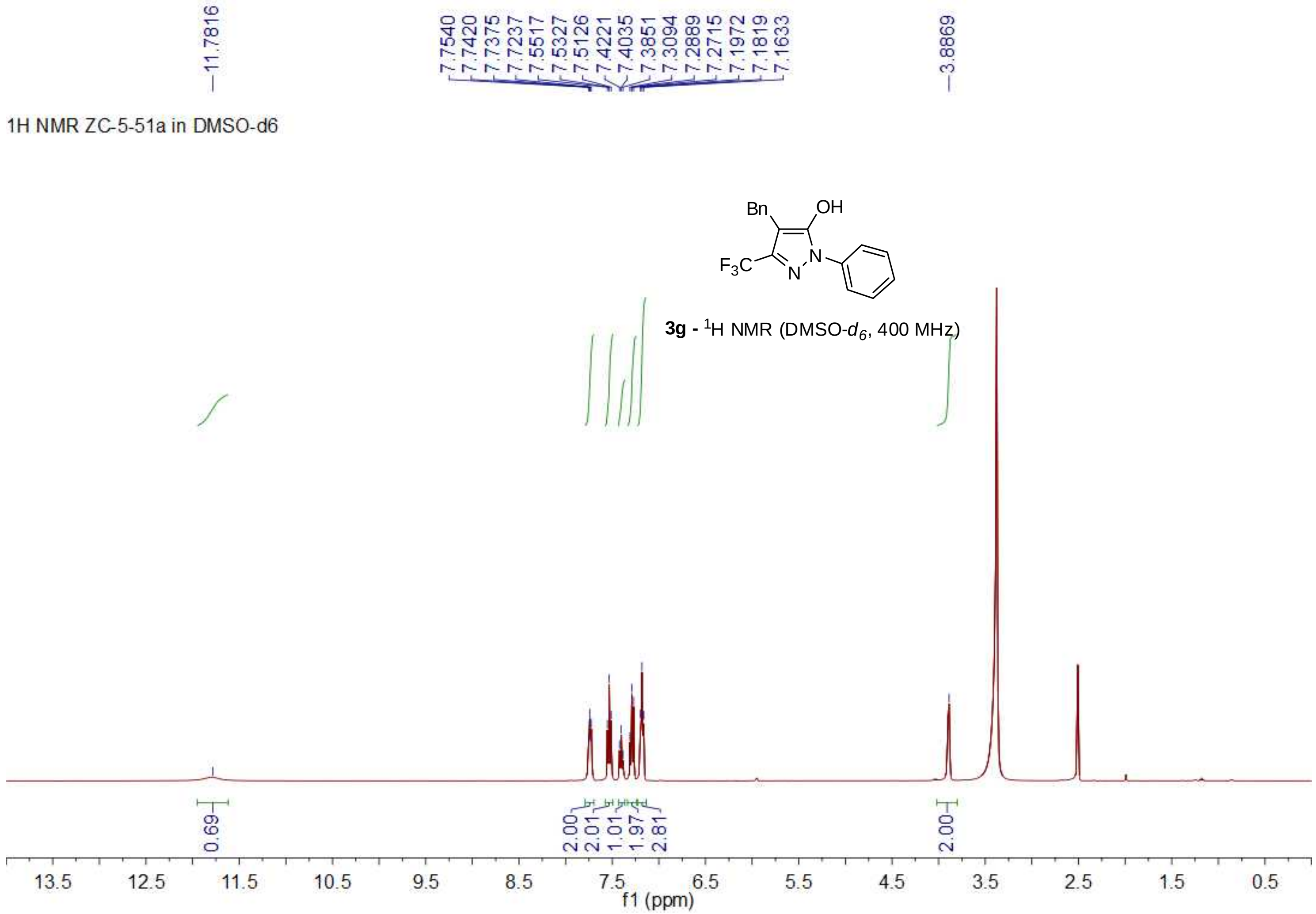

11.7816
7.7540
7.7420
7.7375
7.7237
7.5517
7.5327
7.5126
7.4221
7.4035
7.3851
7.3094
7.2889
7.2715
7.1972
7.1819
7.1633
3.8869
1H NMR ZC-5-51a in DMSO-d6
Bn
OH
F_3C
N
N
3g - 1H NMR (DMSO-d6, 400 MHz)
0.69
2.00
2.01
1.01
1.97
2.81
2.00
13.5
12.5
11.5
10.5
9.5
8.5
7.5
6.5
5.5
4.5
3.5
2.5
1.5
0.5
f1 (ppm)

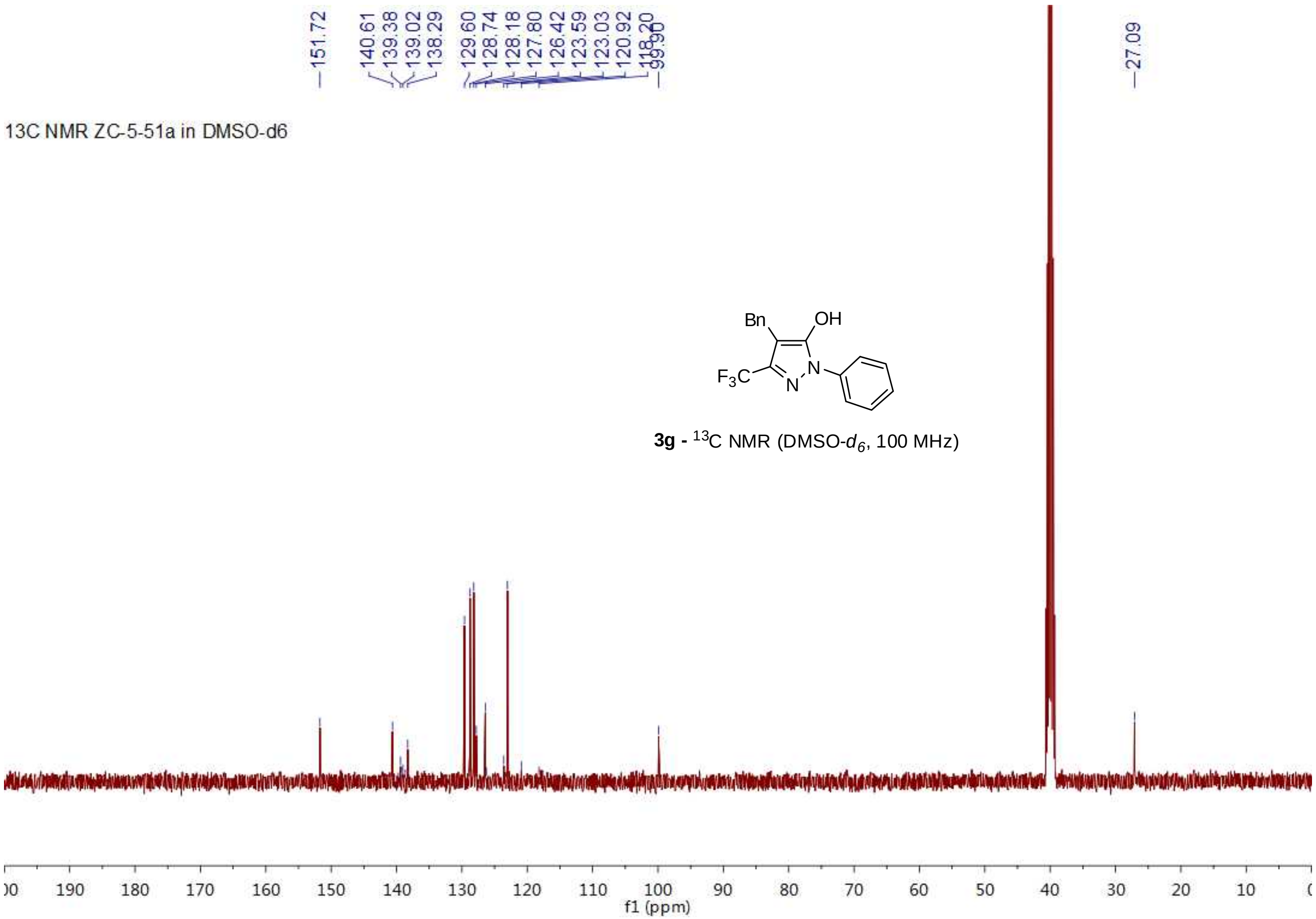

3g - ^{13}C NMR (DMSO-d_6, 100 MHz)

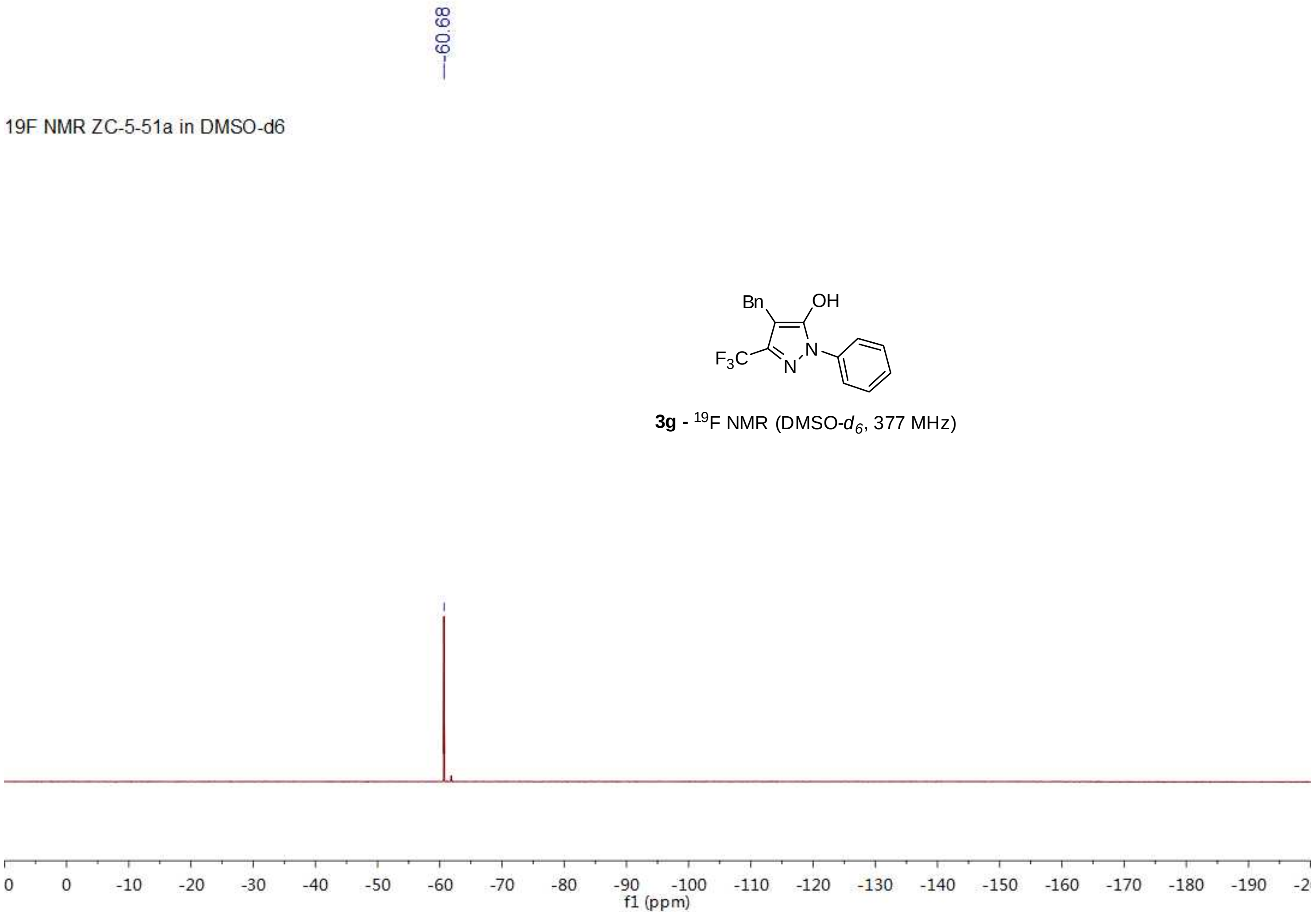

3g - ^{19}F NMR (DMSO-d_6, 377 MHz)

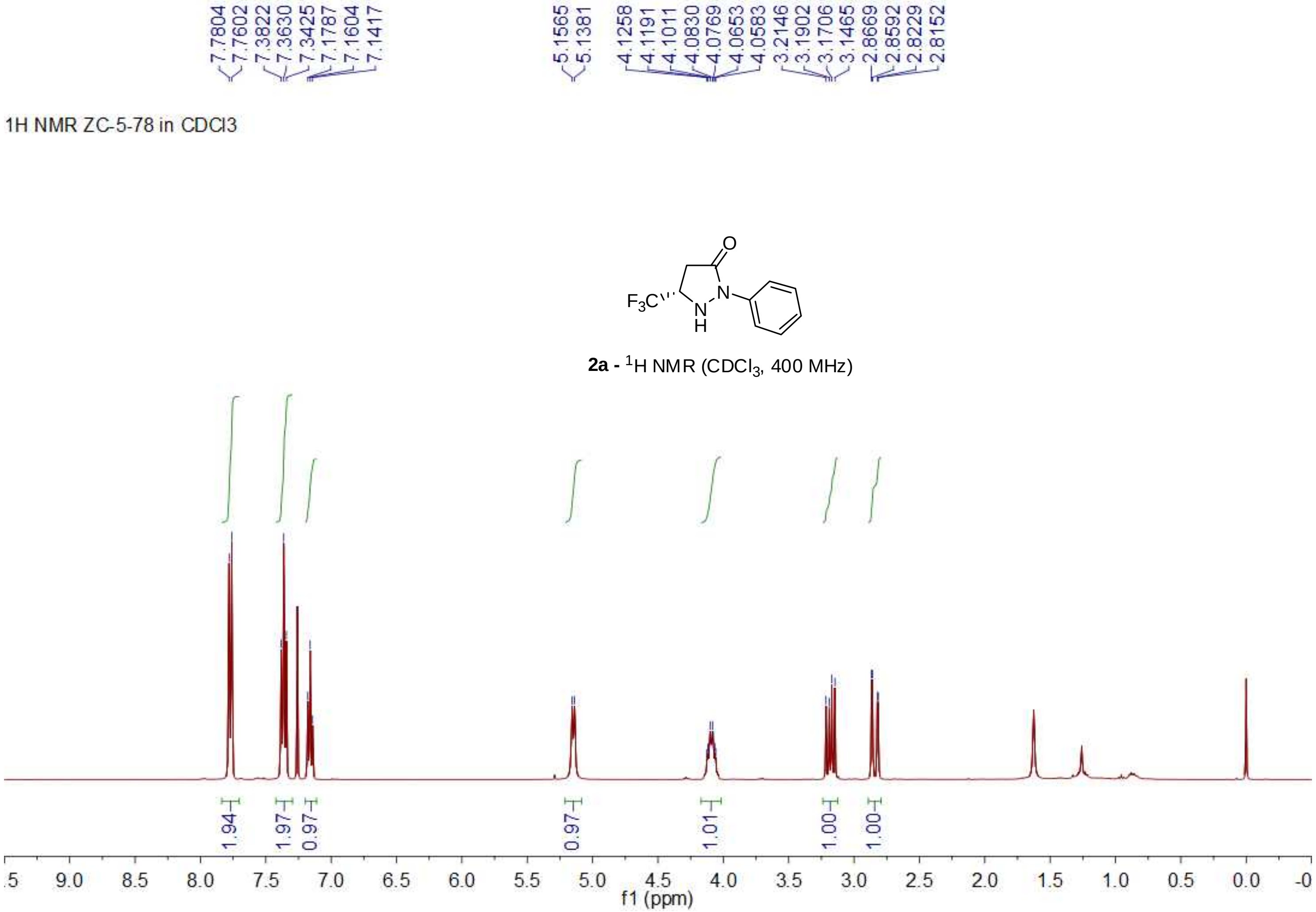

1H NMR ZC-5-78 in CDCl3
7.7804
7.7602
7.3822
7.3630
7.3425
7.1787
7.1604
7.1417
5.1565
5.1381
4.1258
4.1191
4.1011
4.0830
4.0769
4.0653
4.0583
3.2146
3.1902
3.1706
3.1465
2.8669
2.8592
2.8229
2.8152
F3C
N
H
N
O
2a - 1H NMR (CDCl3, 400 MHz)
1.94
1.97
0.97
0.97
1.01
1.00
1.00
.5
9.0
8.5
8.0
7.5
7.0
6.5
6.0
5.5
5.0
4.5
4.0
3.5
3.0
2.5
2.0
1.5
1.0
0.5
0.0
-0
f1 (ppm)

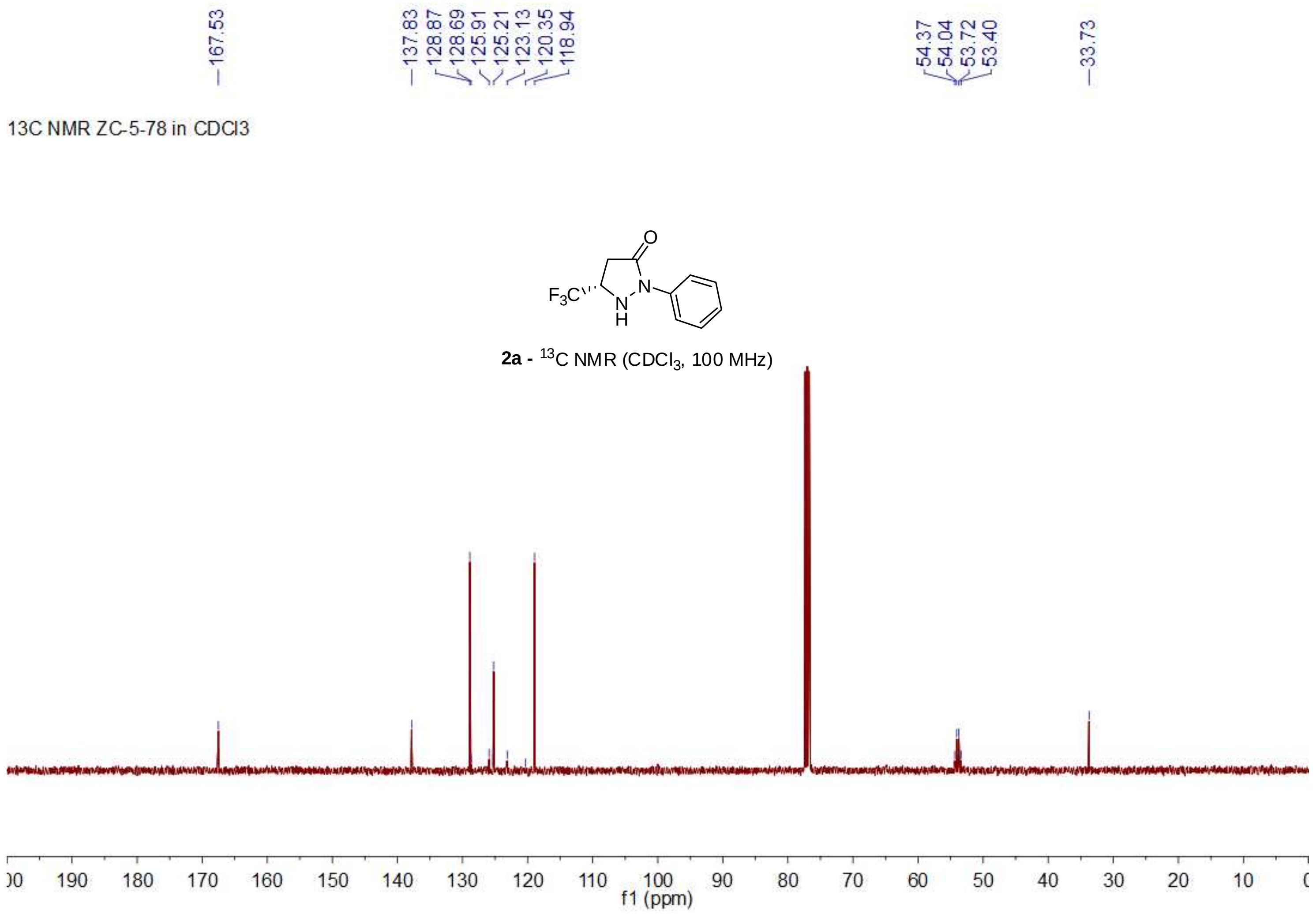

2a - ^{13}C NMR ($CDCl_3$, 100 MHz)

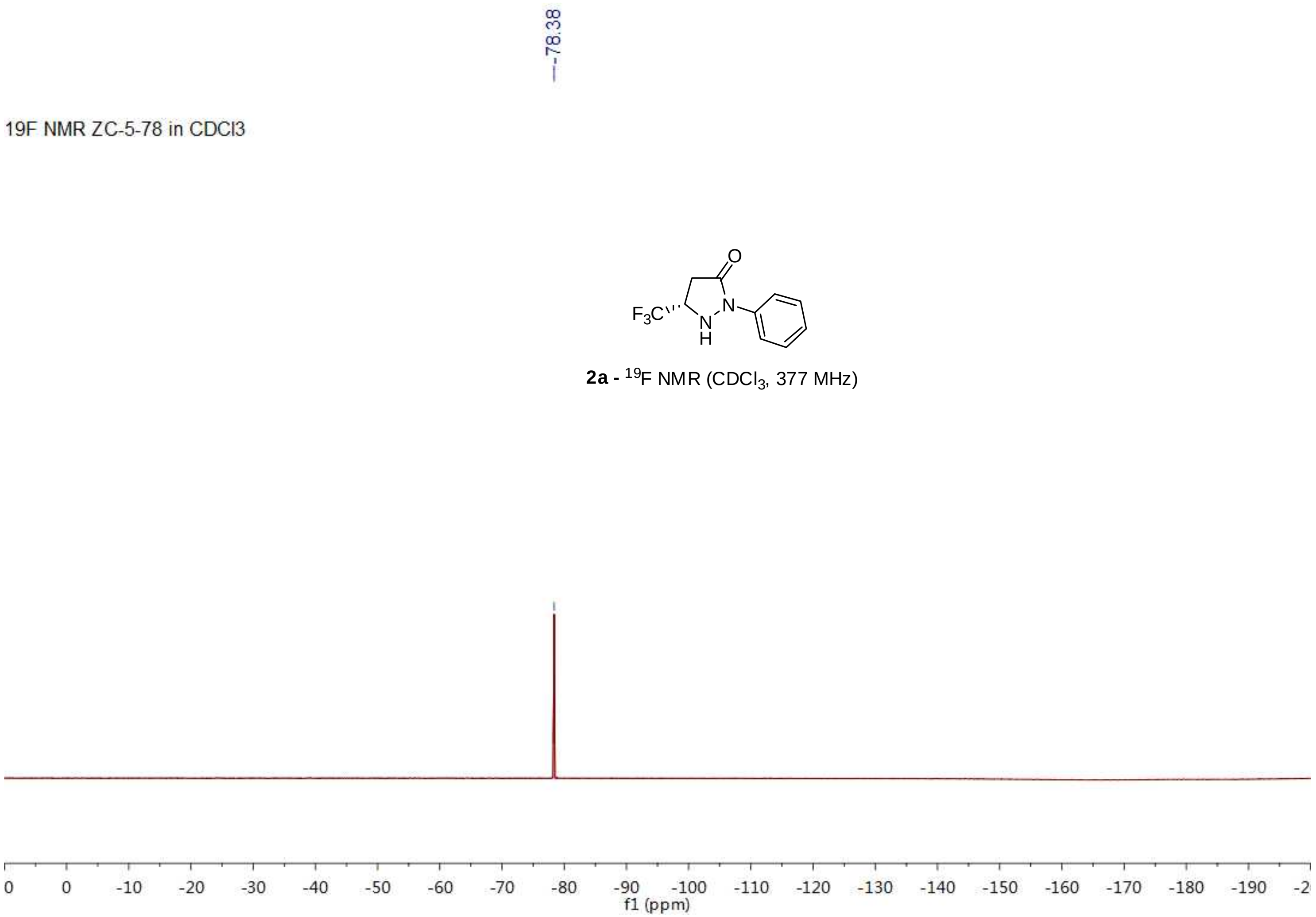

2a - ^{19}F NMR ($CDCl_3$, 377 MHz)

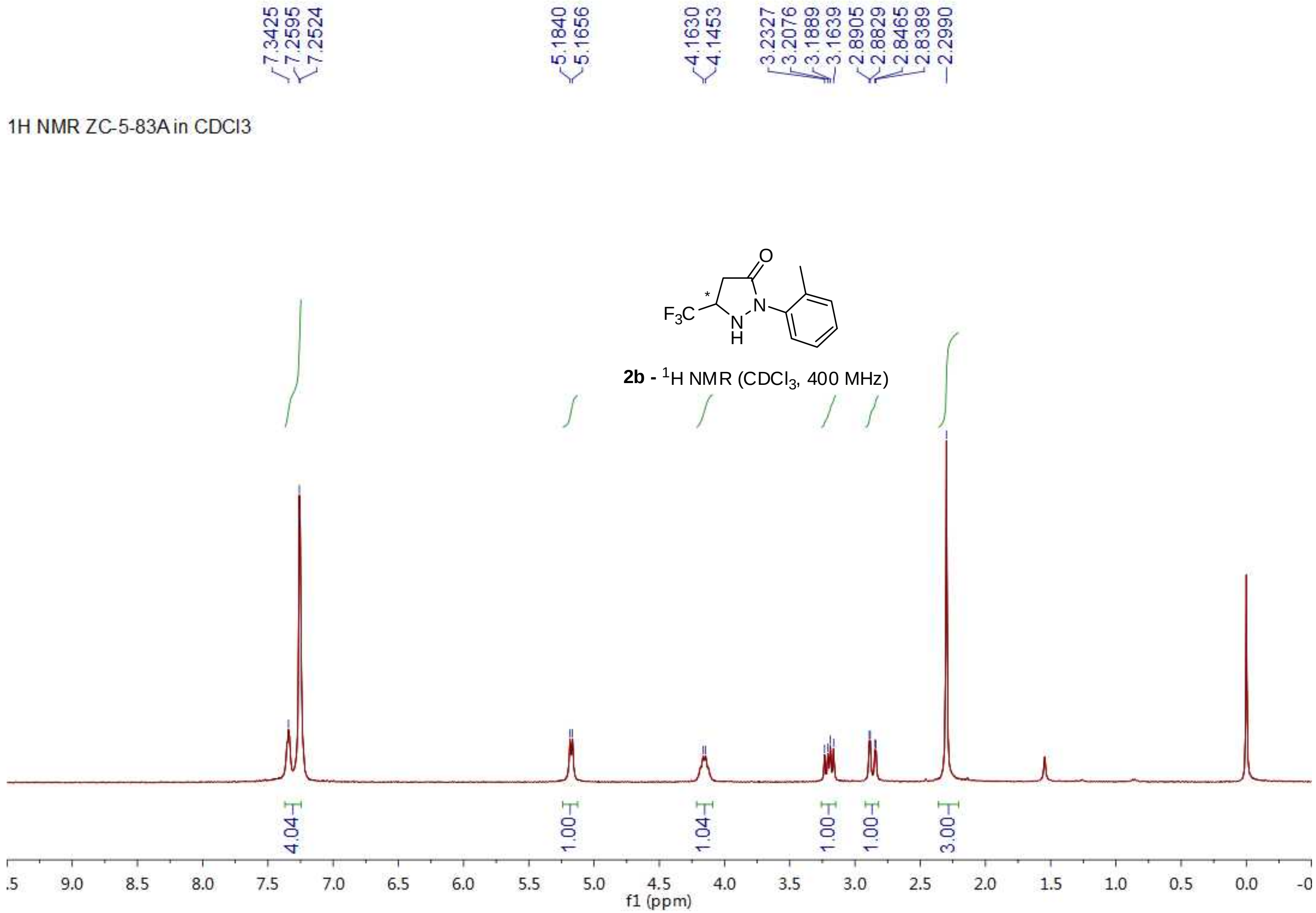
7.3425
7.2595
7.2524
5.1840
5.1656
4.1630
4.1453
3.2327
3.2076
3.1889
3.1639
2.8905
2.8829
2.8465
2.8389
2.2990
1H NMR ZC-5-83A in CDCl3
O
N
F3C
N
H
*
2b - 1H NMR (CDCl3, 400 MHz)
4.04
1.00
1.04
1.00
1.00
3.00
f1 (ppm)

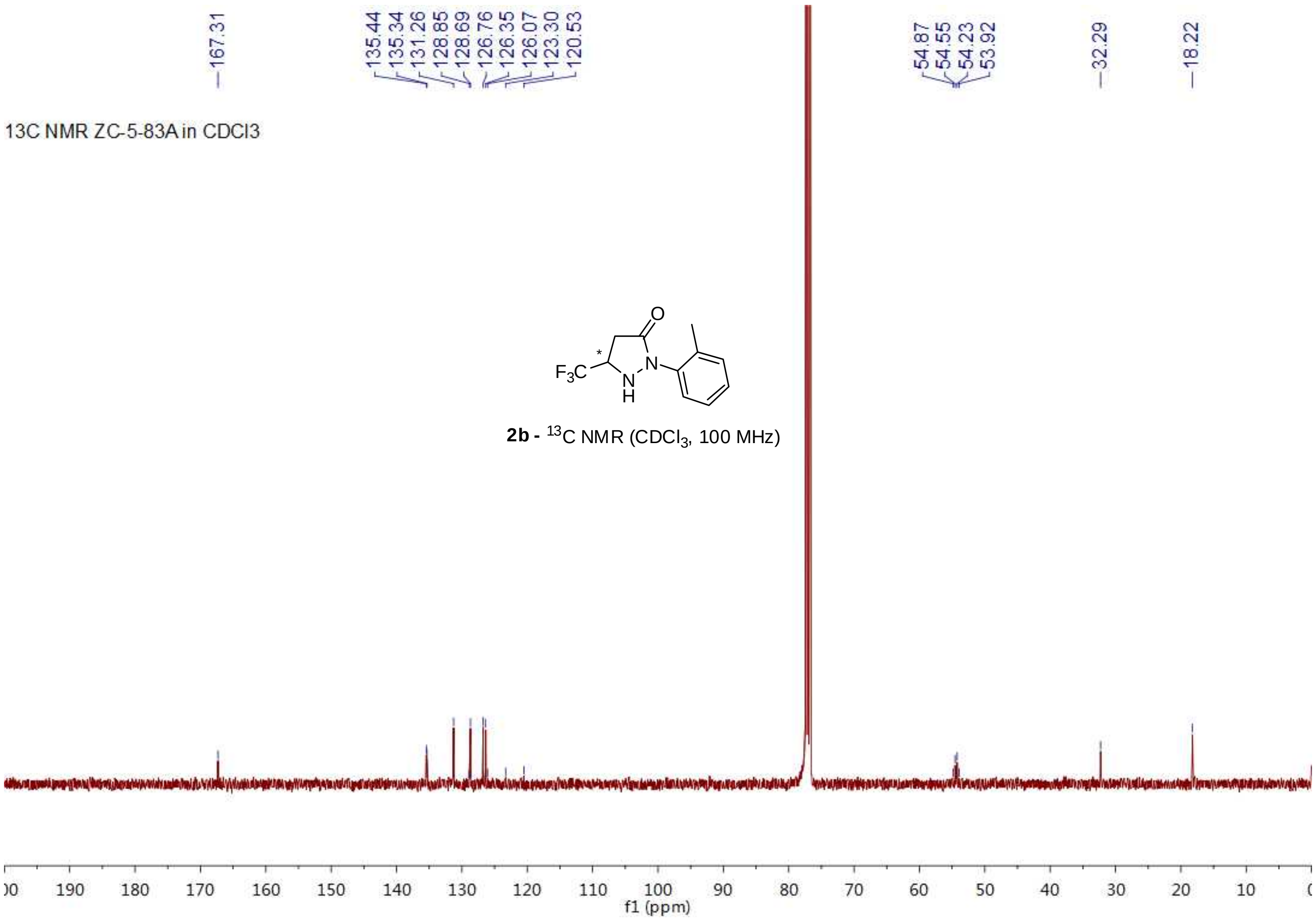

2b - ^{13}C NMR (CDCl$_3$, 100 MHz)

19F NMR ZC-5-83A in CDCl3

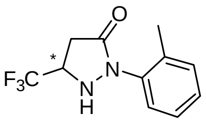

2b - ^{19}F NMR ($CDCl_3$, 377 MHz)

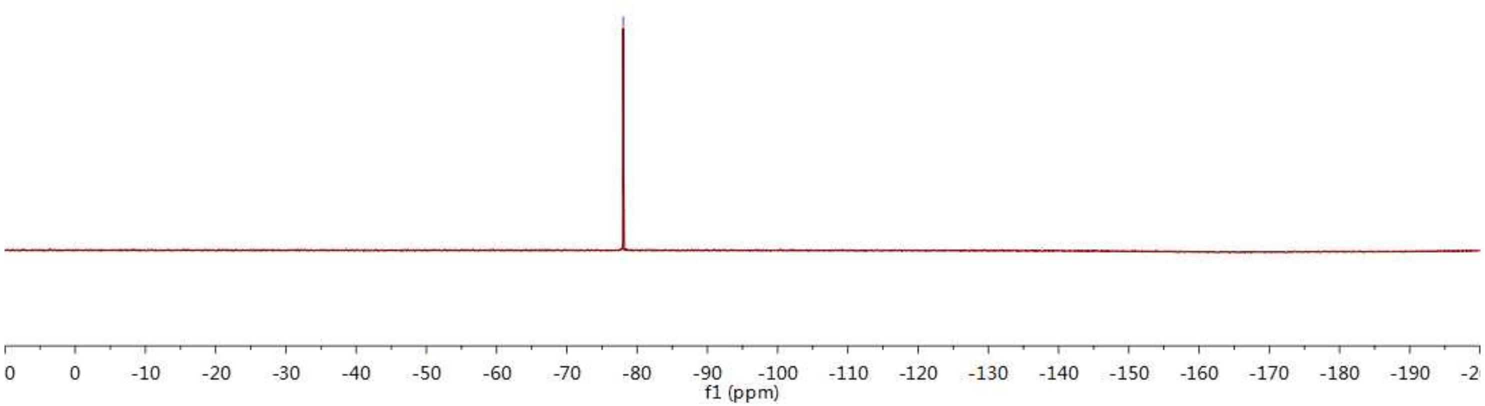

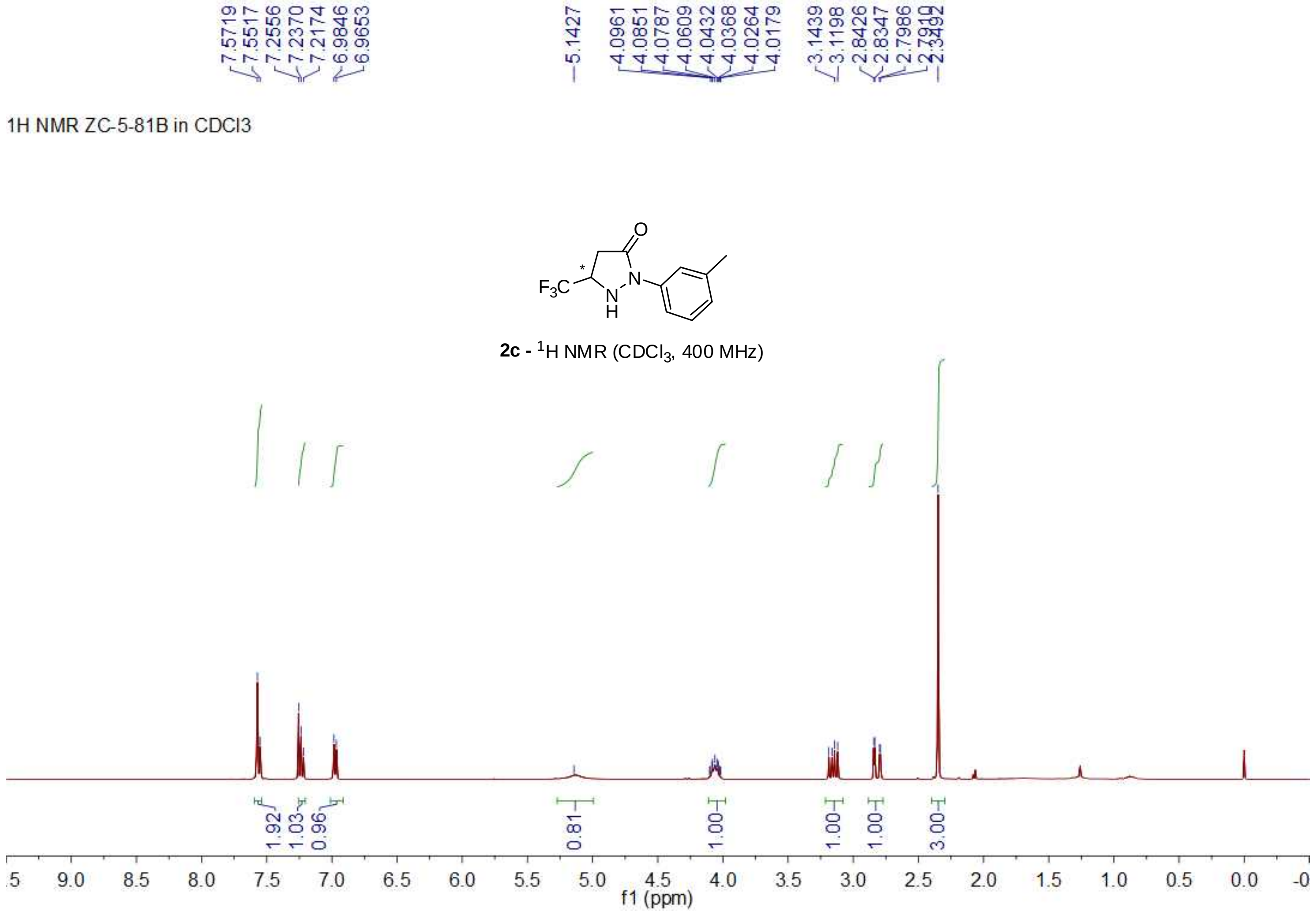
7.5719
7.5517
7.2556
7.2370
7.2174
6.9846
6.9653
5.1427
4.0961
4.0851
4.0787
4.0609
4.0432
4.0368
4.0264
4.0179
3.1439
3.1198
2.8426
2.8347
2.7986
2.7910
2.3492
1H NMR ZC-5-81B in CDCl3
O
F3C
*
N
N
H
2c - 1H NMR (CDCl3, 400 MHz)
1.92
1.03
0.96
0.81
1.00
1.00
1.00
3.00
f1 (ppm)

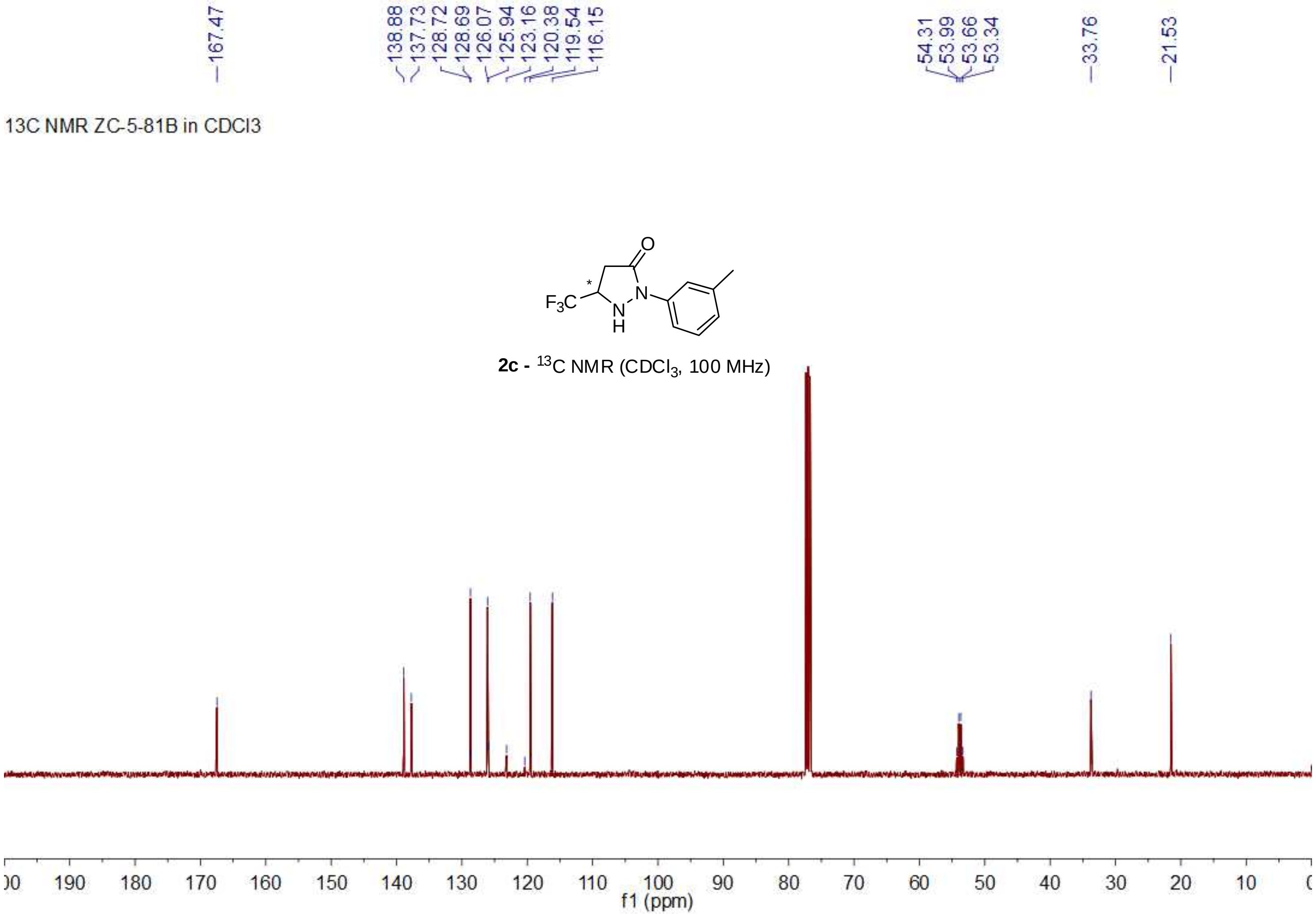

2c - ^{13}C NMR ($CDCl_3$, 100 MHz)

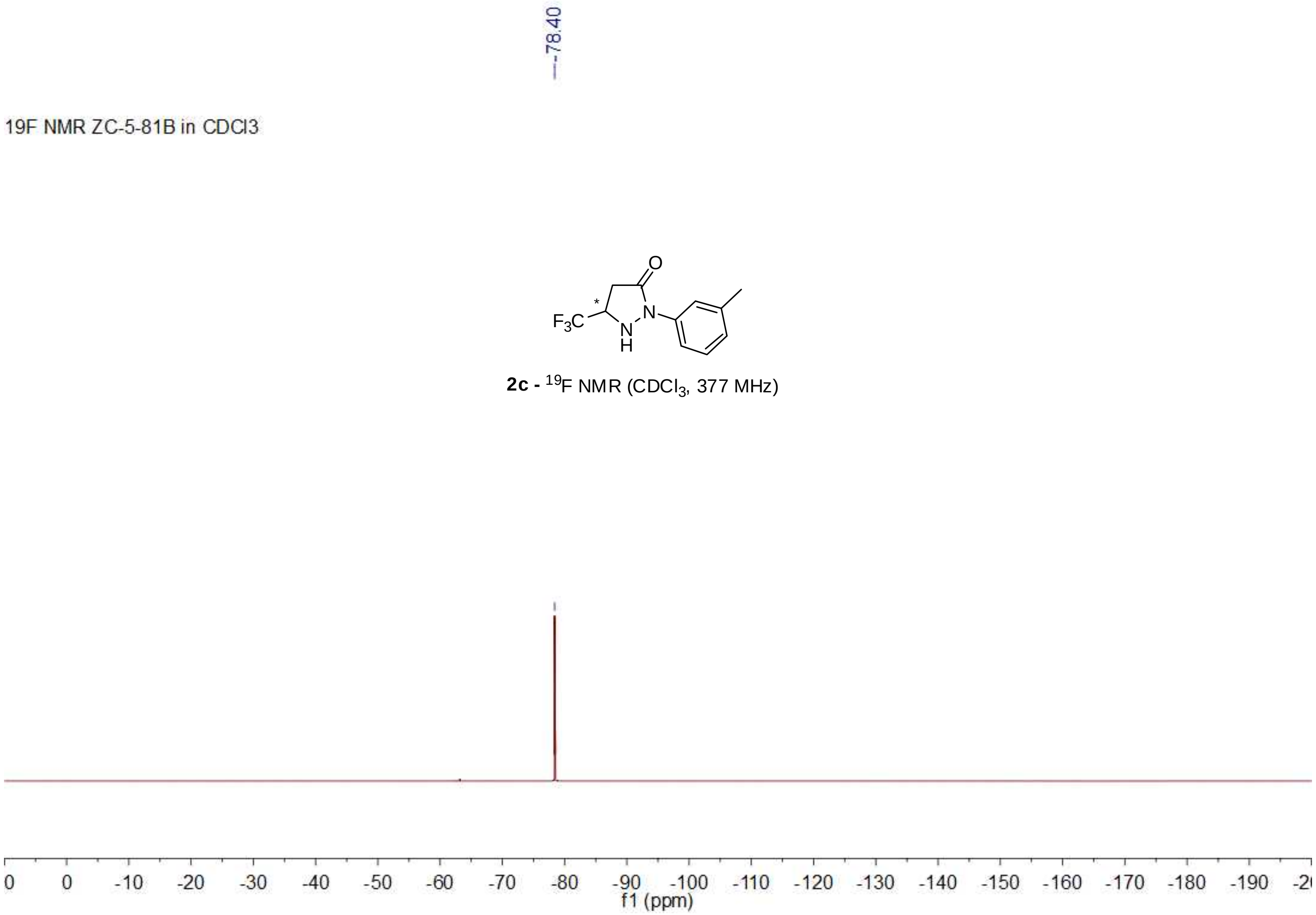

2c - ^{19}F NMR ($CDCl_3$, 377 MHz)

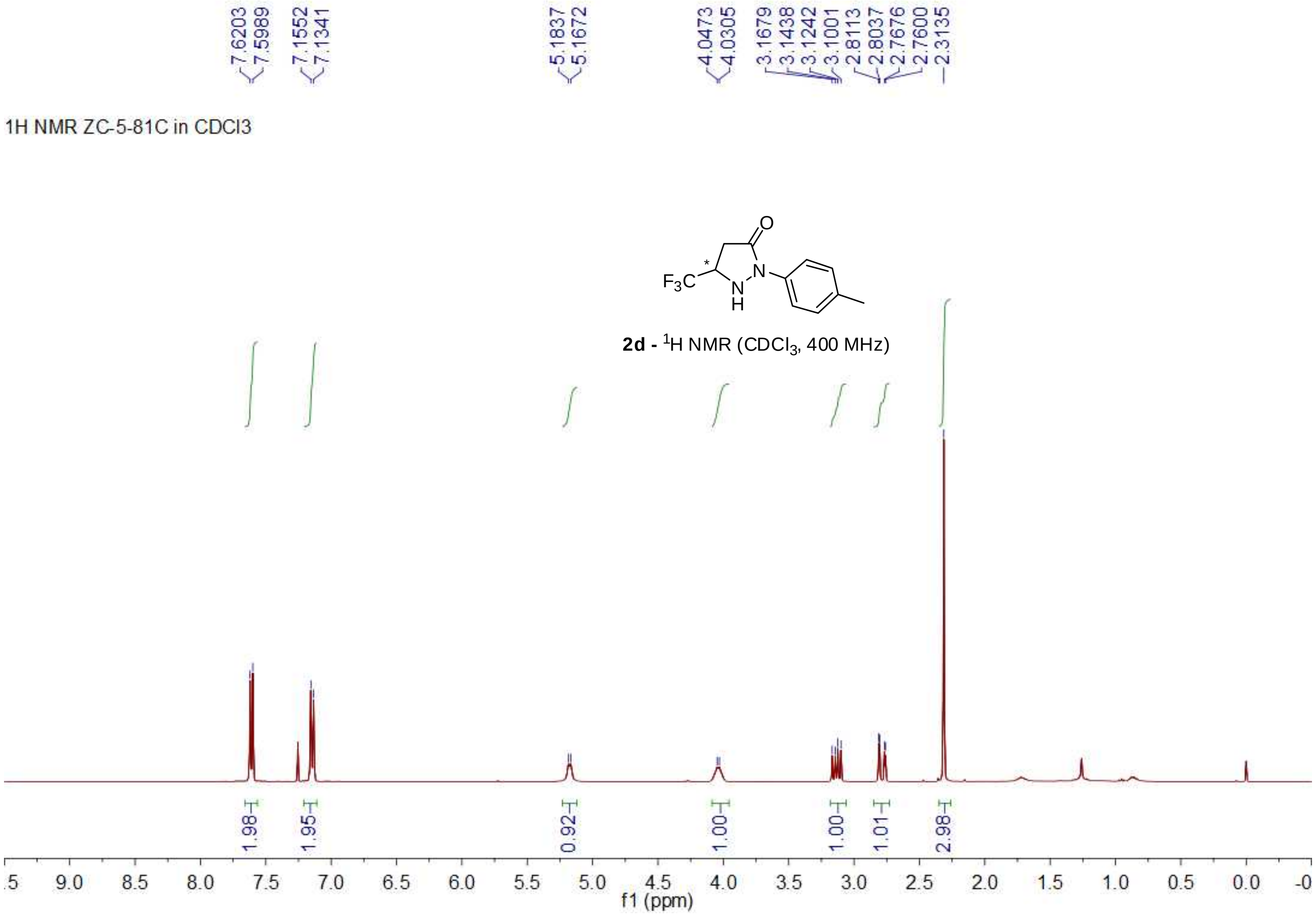

7.6203
7.5989
7.1552
7.1341
5.1837
5.1672
4.0473
4.0305
3.1679
3.1438
3.1242
3.1001
2.8113
2.8037
2.7676
2.7600
2.3135
1H NMR ZC-5-81C in CDCl3
F3C
N
N
H
O
2d - 1H NMR (CDCl3, 400 MHz)
1.98
1.95
0.92
1.00
1.00
1.01
2.98
f1 (ppm)

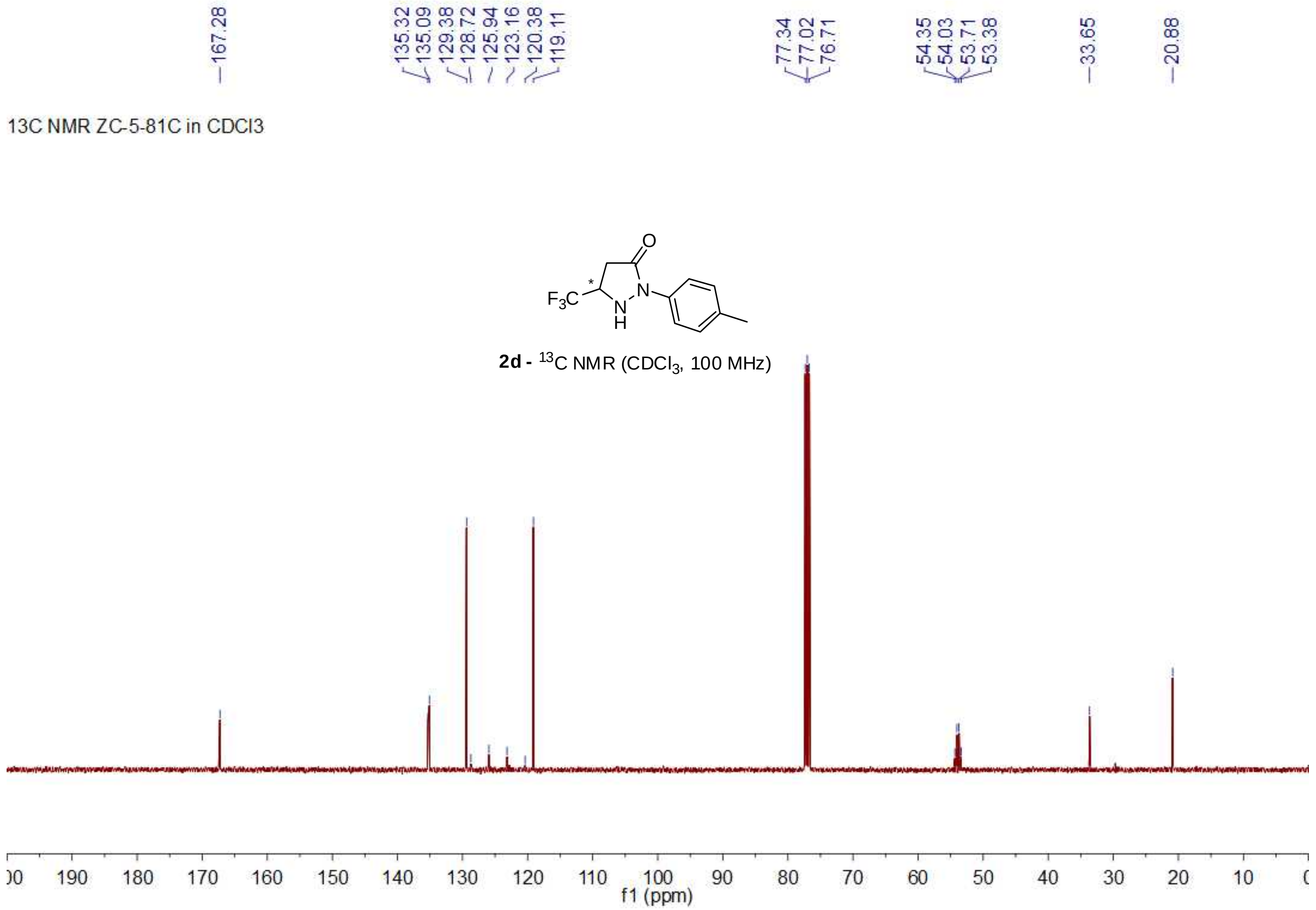
13C NMR ZC-5-81C in CDCl3
167.28
135.32
135.09
129.38
128.72
125.94
123.16
120.38
119.11
77.34
77.02
76.71
54.35
54.03
53.71
53.38
33.65
20.88
O
F3C
*
N
N
H
2d - 13C NMR (CDCl3, 100 MHz)
190 180 170 160 150 140 130 120 110 100 90 80 70 60 50 40 30 20 10
f1 (ppm)

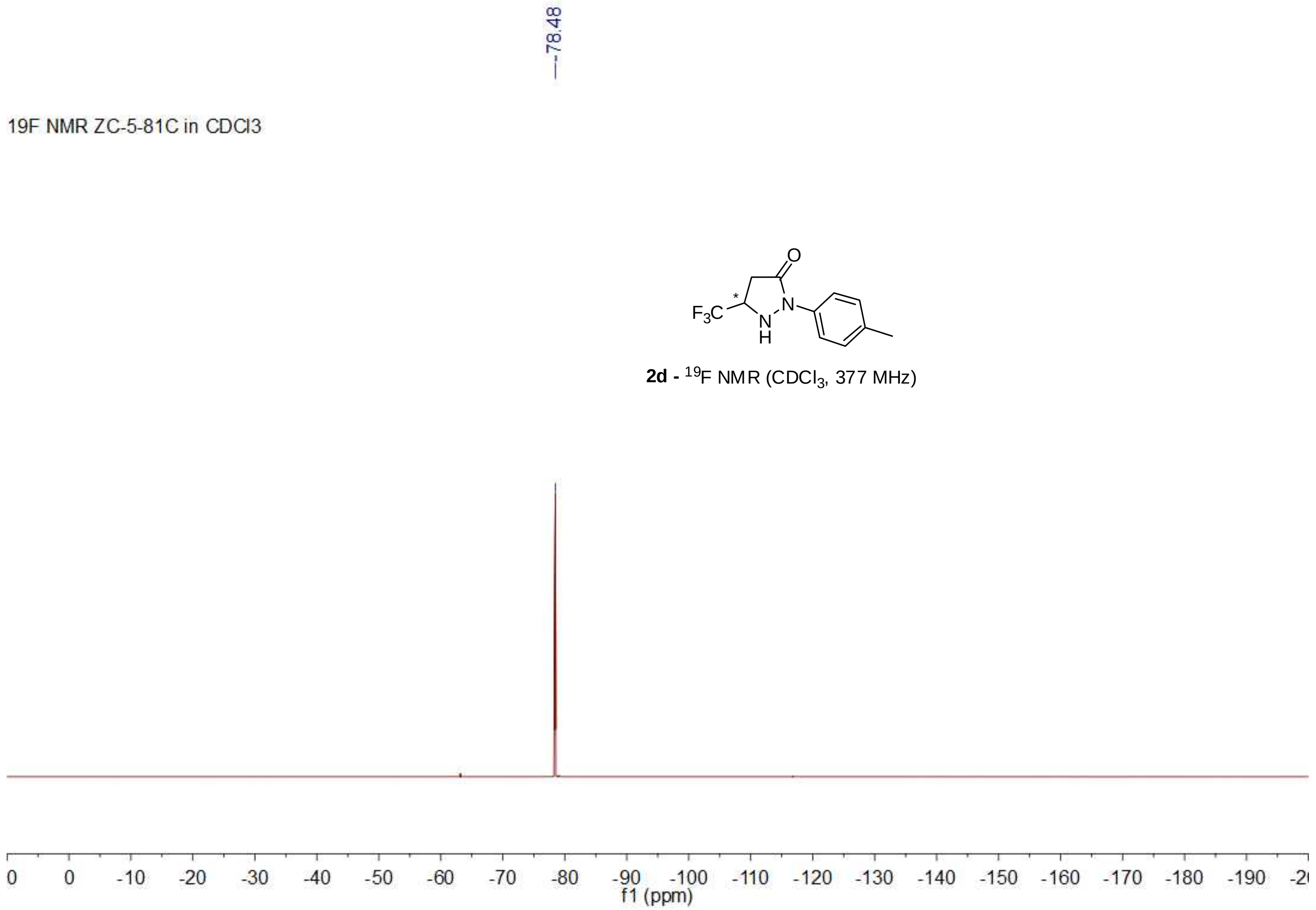

-78.48
19F NMR ZC-5-81C in CDCl3
O
F3C
*
N
N
H
2d - 19F NMR (CDCl3, 377 MHz)
0
0
-10
-20
-30
-40
-50
-60
-70
-80
-90
-100
-110
-120
-130
-140
-150
-160
-170
-180
-190
f1 (ppm)

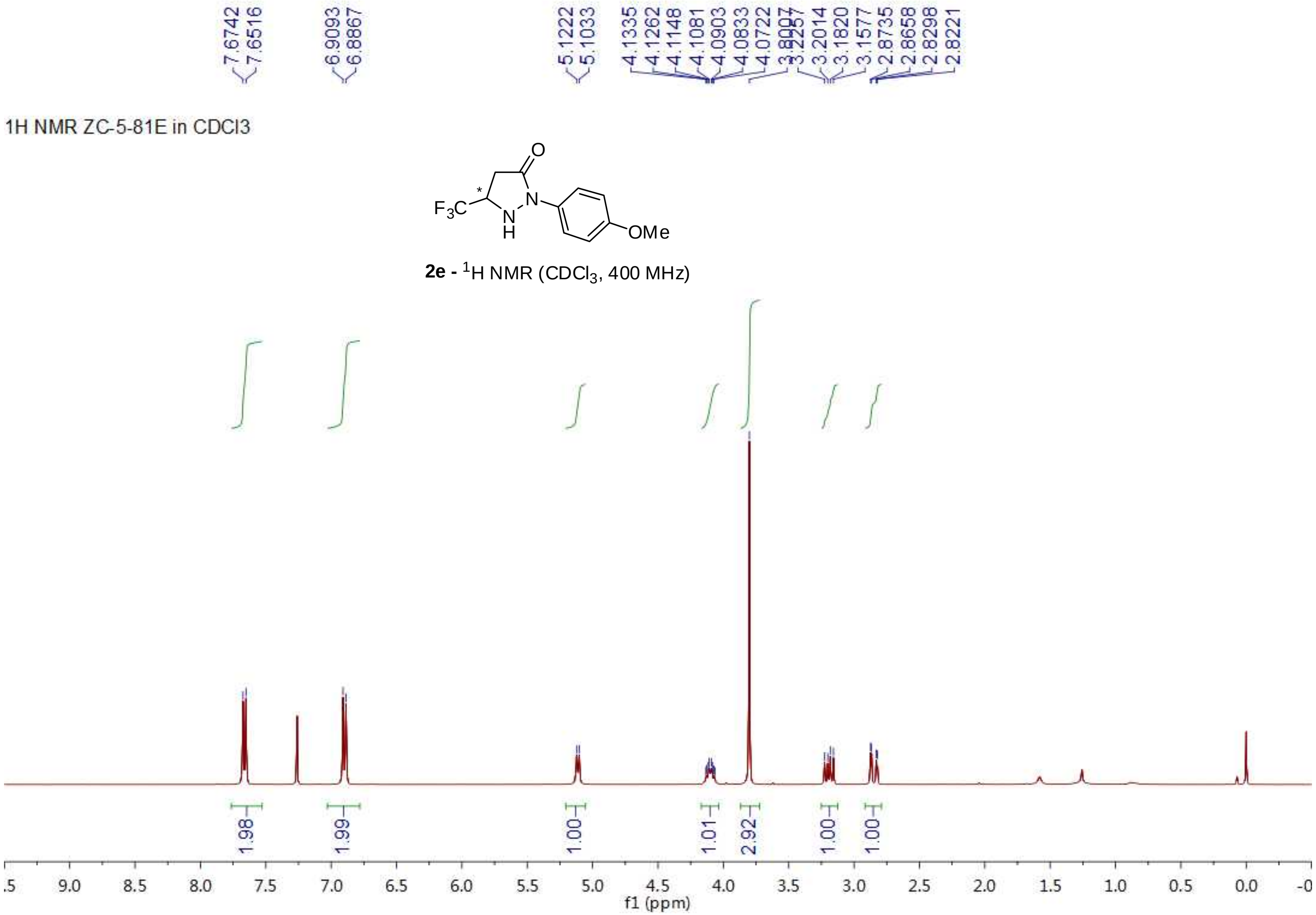

2e - ^{1}H NMR ($CDCl_3$, 400 MHz)

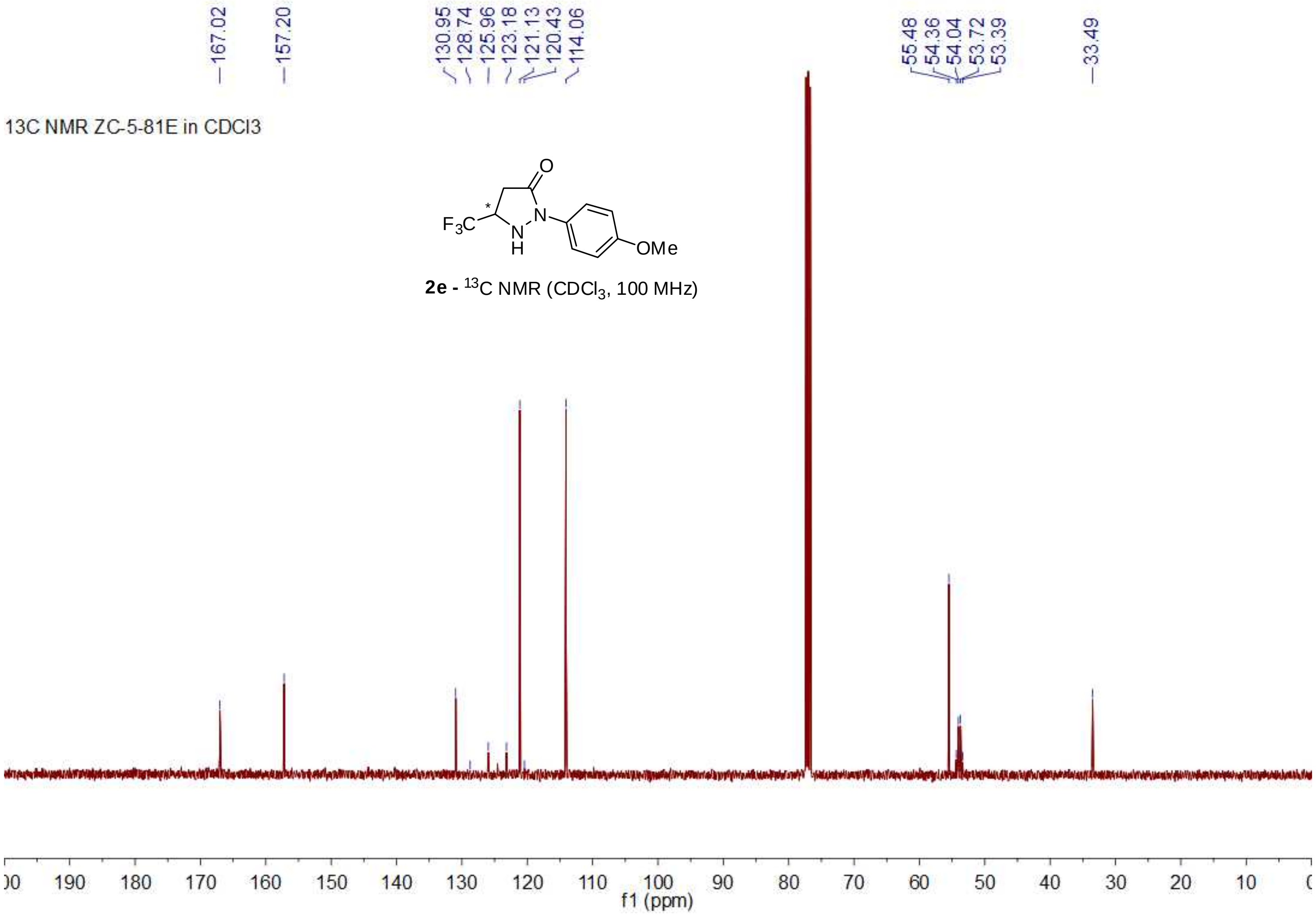
167.02
157.20
130.95
128.74
125.96
123.18
121.13
120.43
114.06
55.48
54.36
54.04
53.72
53.39
33.49
13C NMR ZC-5-81E in CDCl3
O
F3C
*
N
N
H
OMe
2e - 13C NMR (CDCl3, 100 MHz)
f1 (ppm)
190
180
170
160
150
140
130
120
110
100
90
80
70
60
50
40
30
20
10

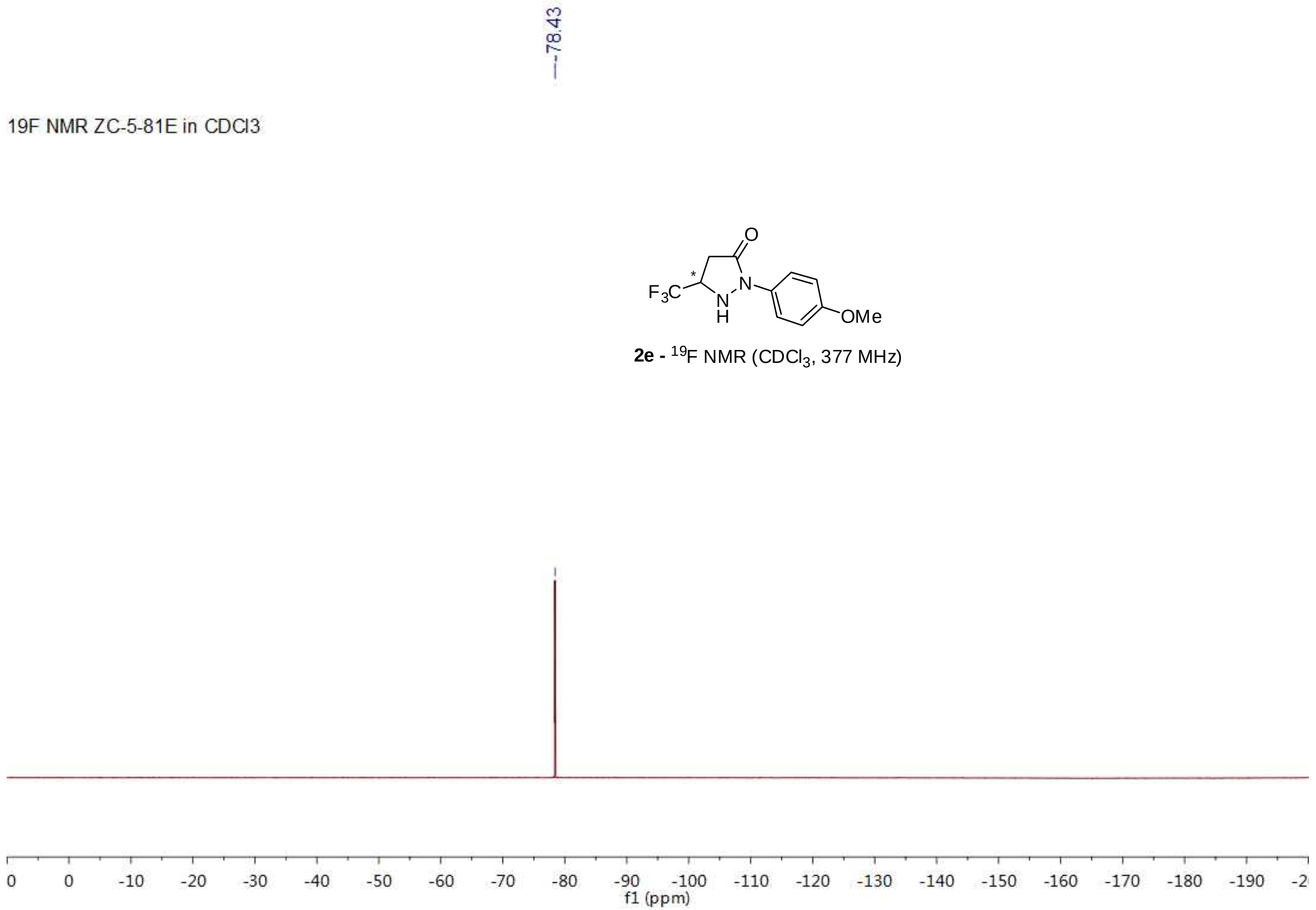

2e - ^{19}F NMR ($CDCl_3$, 377 MHz)

1H NMR ZC-5-81D in CDCl3

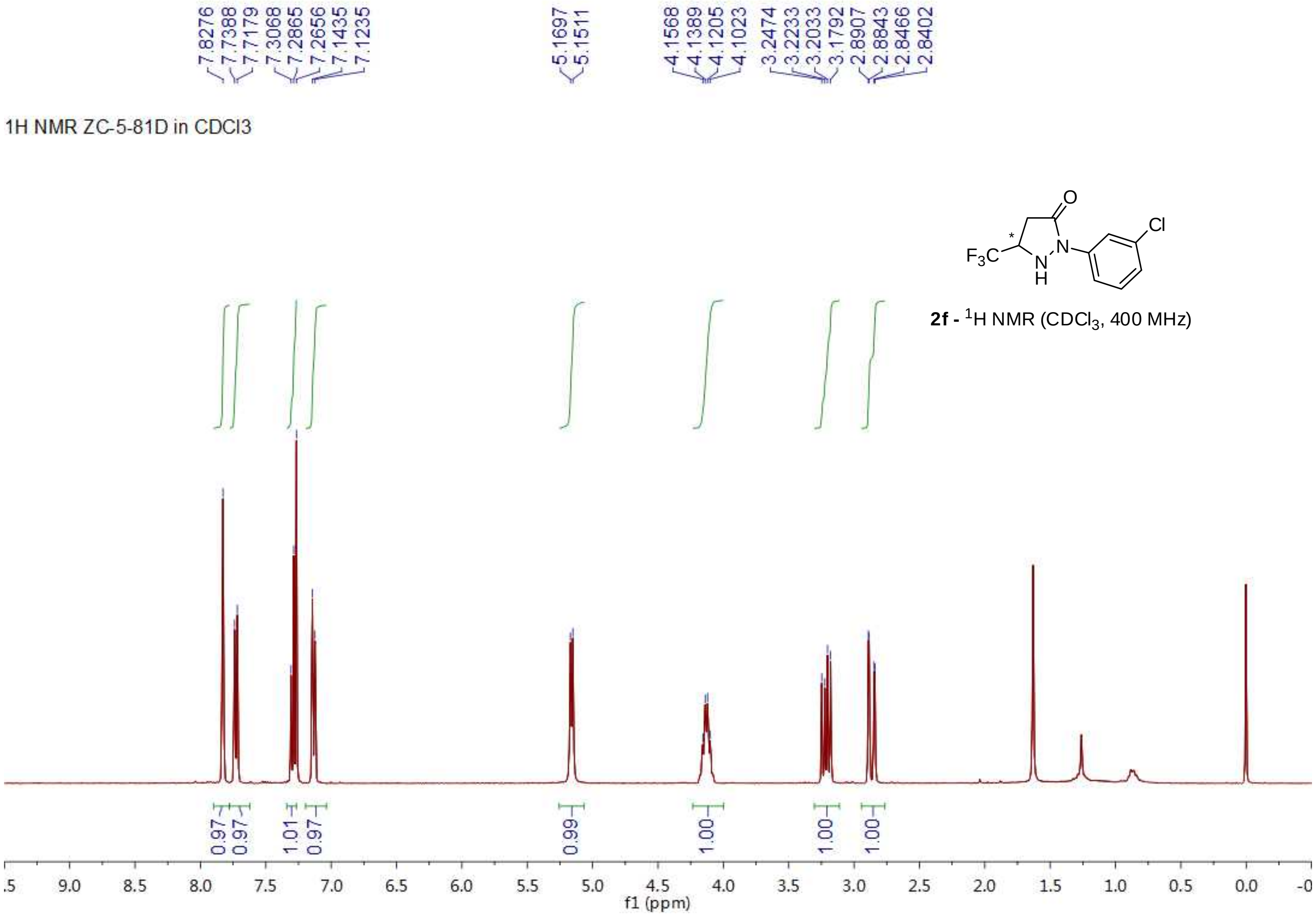

2f - ^{1}H NMR ($CDCl_3$, 400 MHz)

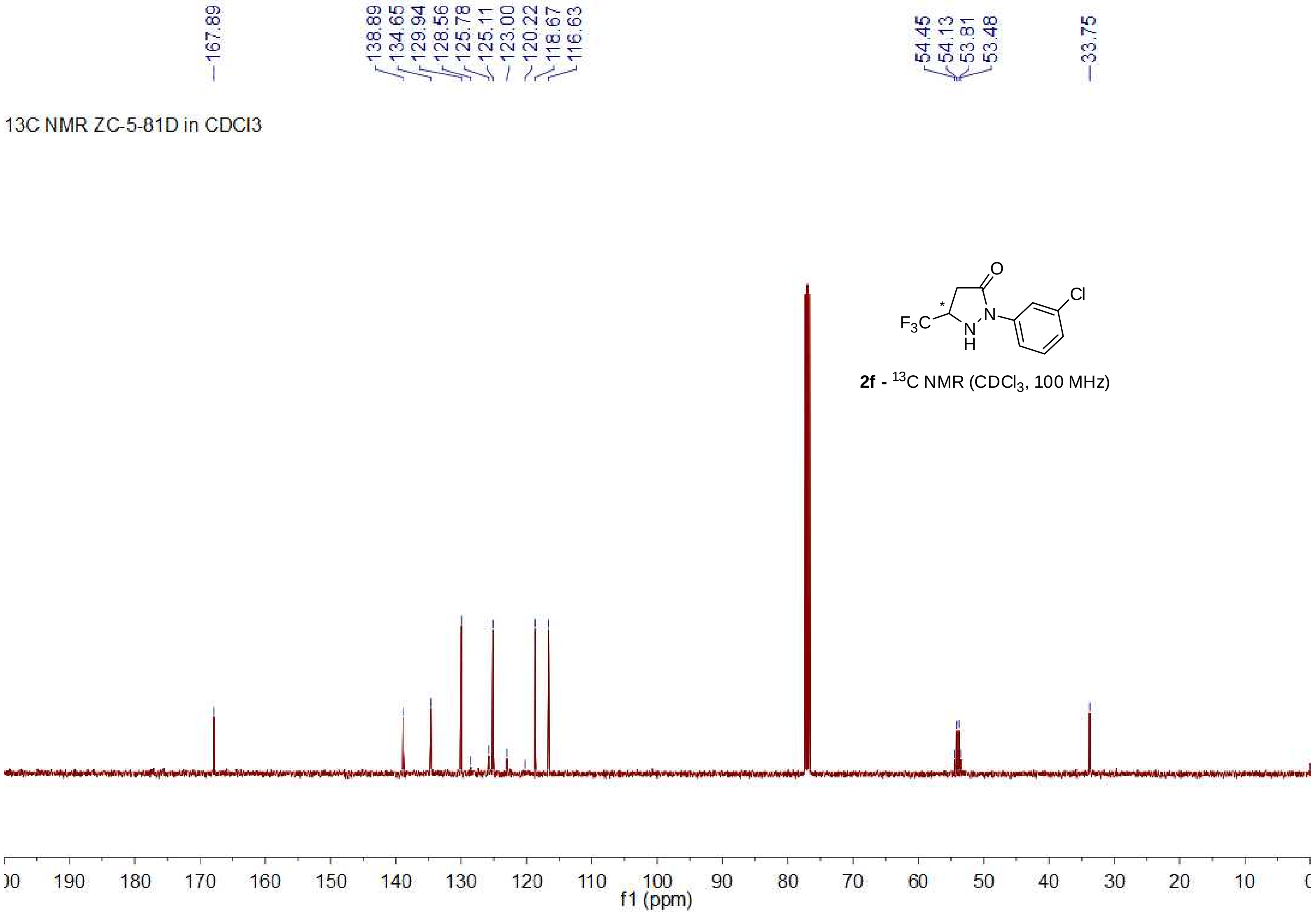
167.89
138.89
134.65
129.94
128.56
125.78
125.11
123.00
120.22
118.67
116.63
54.45
54.13
53.81
53.48
33.75
13C NMR ZC-5-81D in CDCl3
O
Cl
*
F3C
N
N
H
2f - 13C NMR (CDCl3, 100 MHz)
190
180
170
160
150
140
130
120
110
100
90
80
70
60
50
40
30
20
10
f1 (ppm)

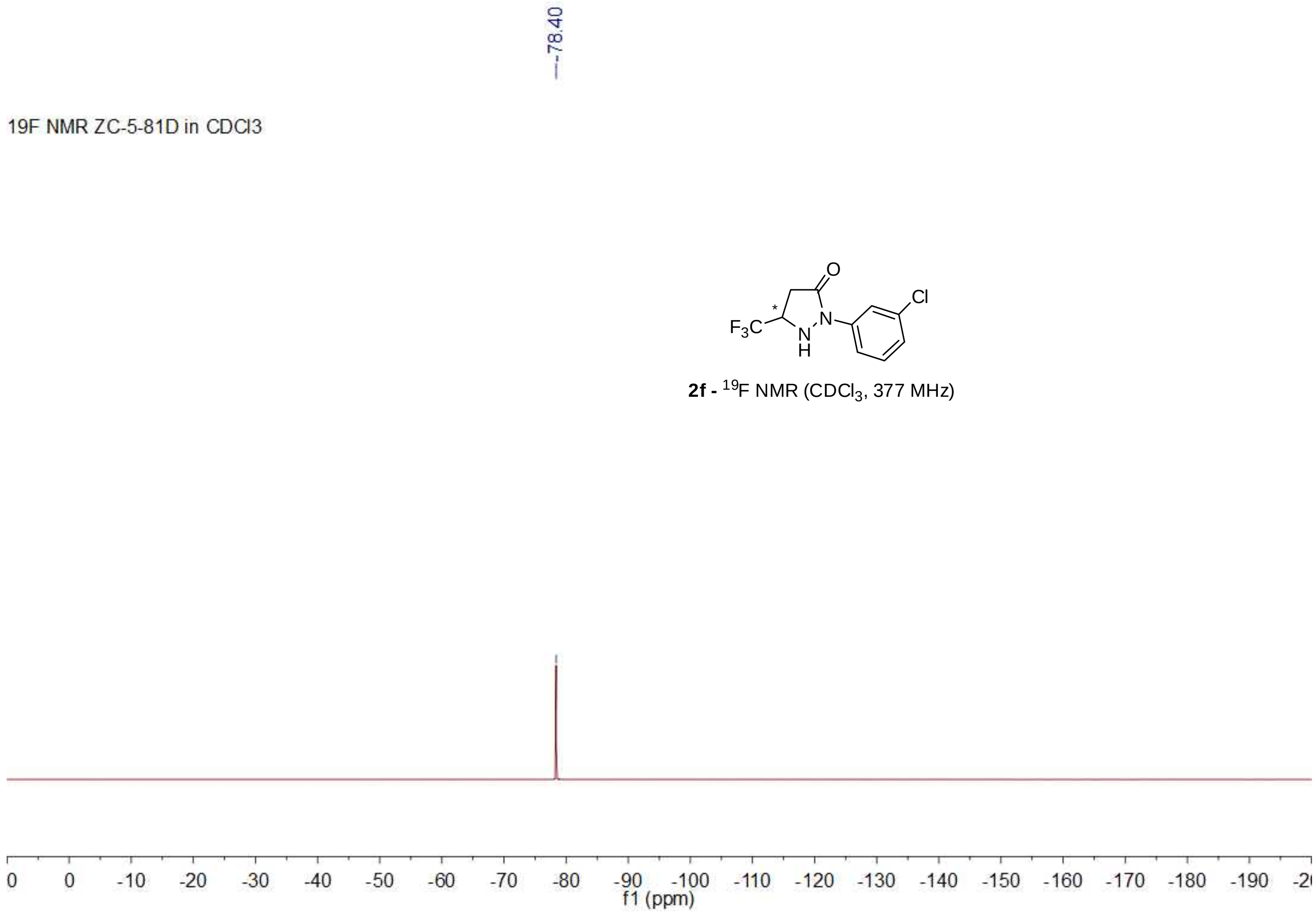

2f - ^{19}F NMR ($CDCl_3$, 377 MHz)

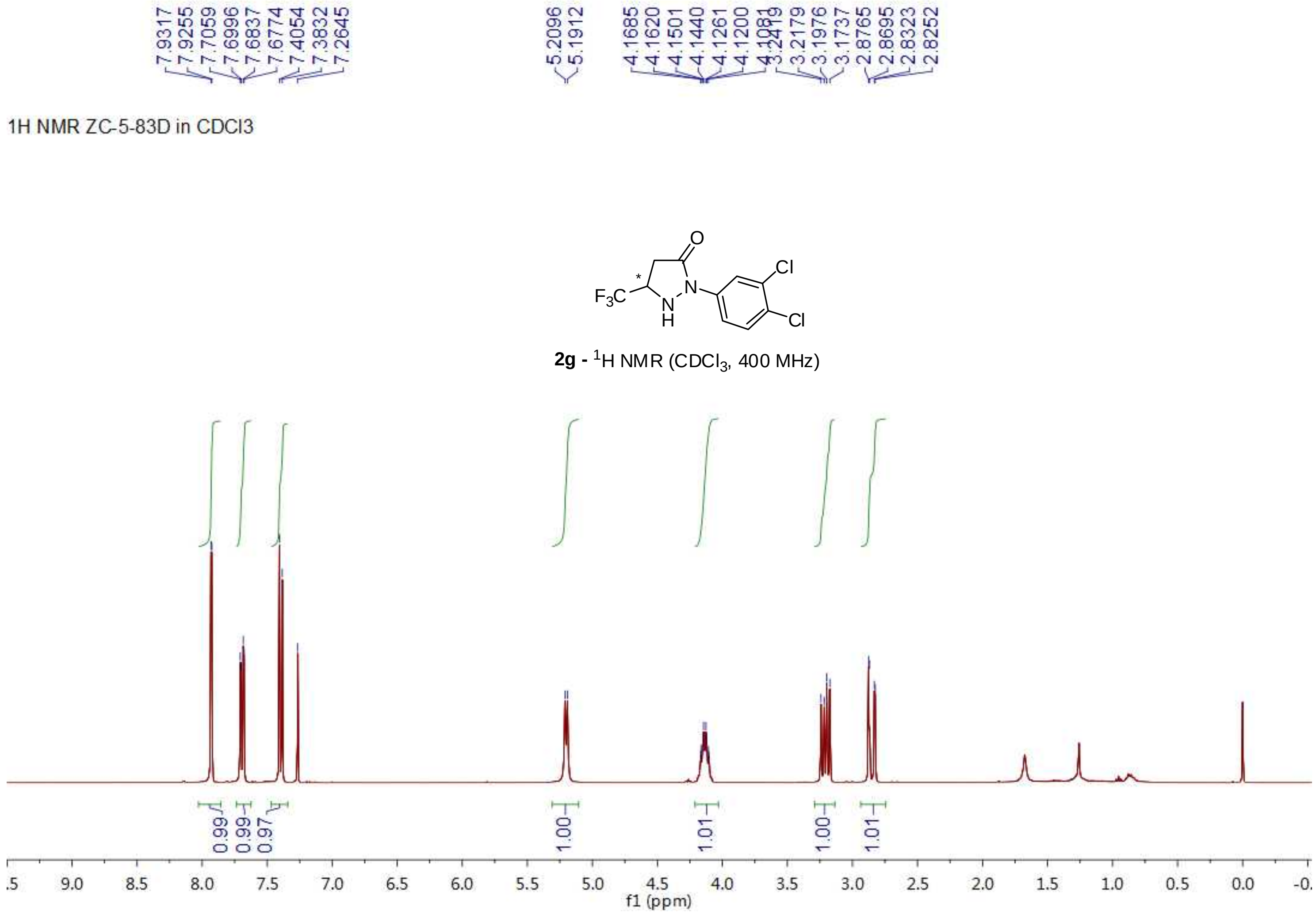

7.9317
7.9255
7.7059
7.6996
7.6837
7.6774
7.4054
7.3832
7.2645
5.2096
5.1912
4.1685
4.1620
4.1501
4.1440
4.1261
4.1200
4.1081
3.2419
3.2179
3.1976
3.1737
2.8765
2.8695
2.8323
2.8252
1H NMR ZC-5-83D in CDCl3
O
Cl
F3C
N
N
H
Cl
2g - 1H NMR (CDCl3, 400 MHz)
0.99
0.99
0.97
1.00
1.01
1.00
1.01
f1 (ppm)

13C NMR ZC-5-83D in DMSO-d6

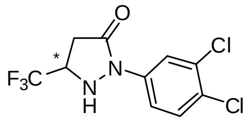

2g - ^{13}C NMR ($CDCl_3$, 100 MHz)

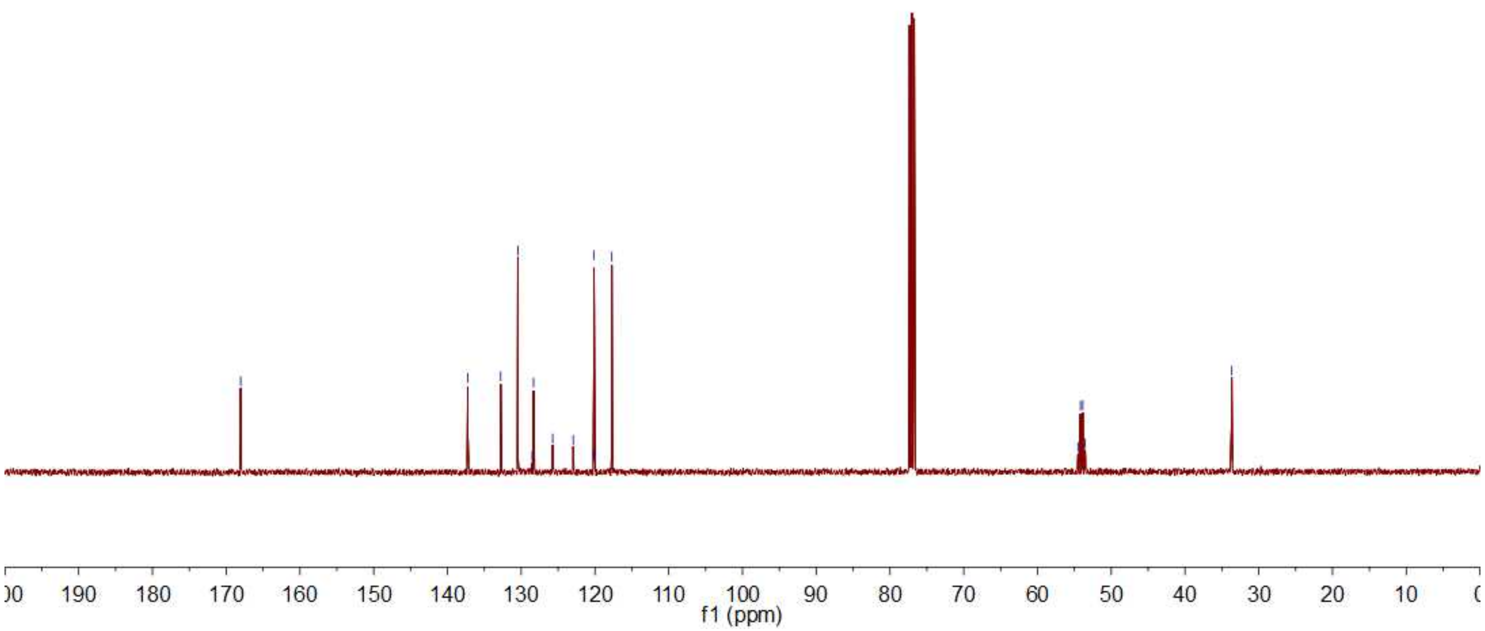

19F NMR ZC-5-83D in CDCl3

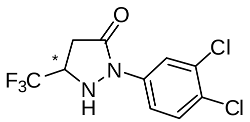

2g - ^{19}F NMR ($CDCl_3$, 377 MHz)

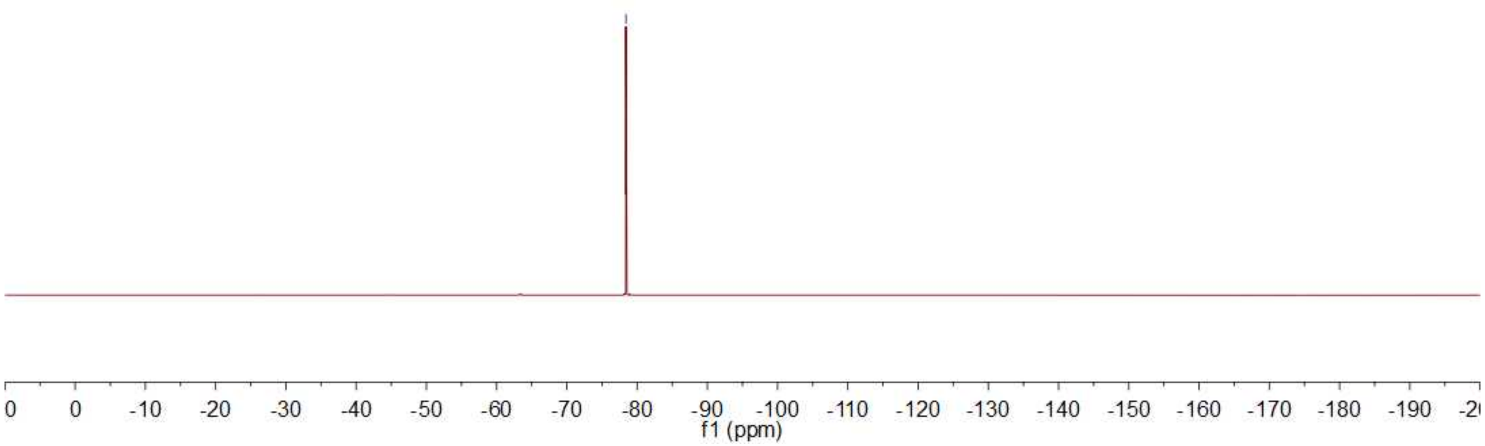

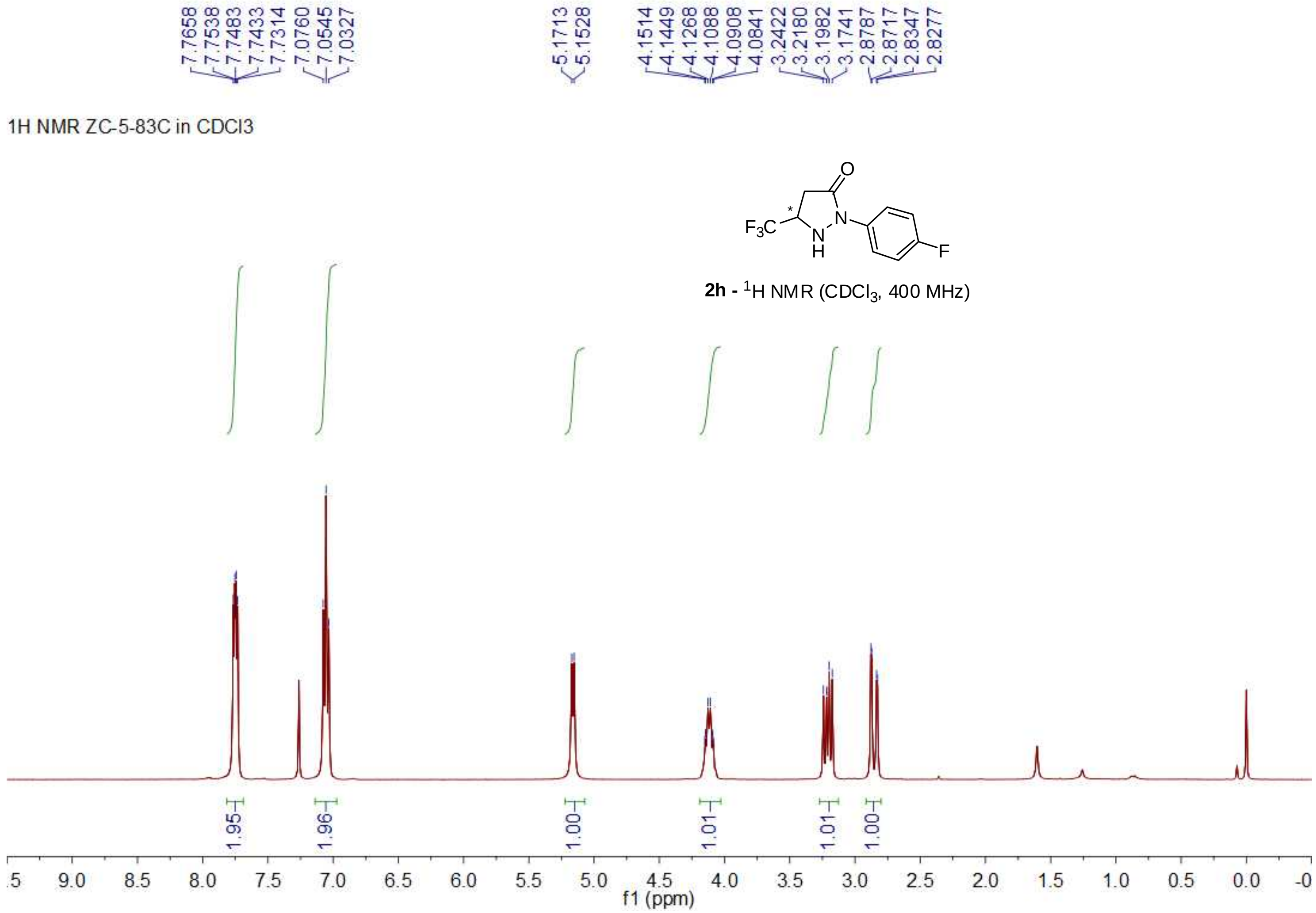

2h - ^{1}H NMR ($CDCl_3$, 400 MHz)

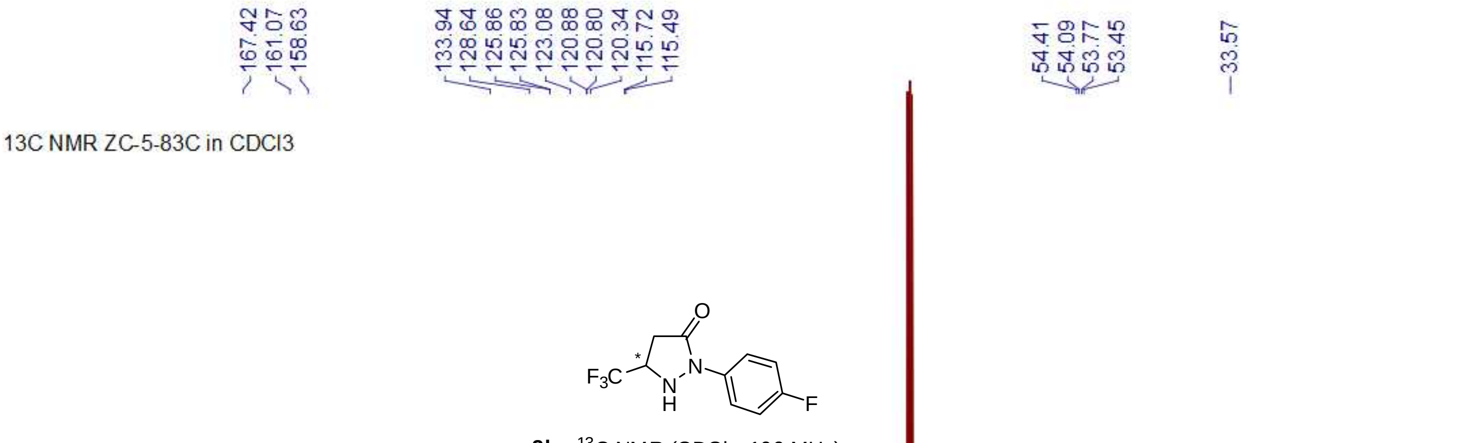

2h - ^{13}C NMR ($CDCl_3$, 100 MHz)

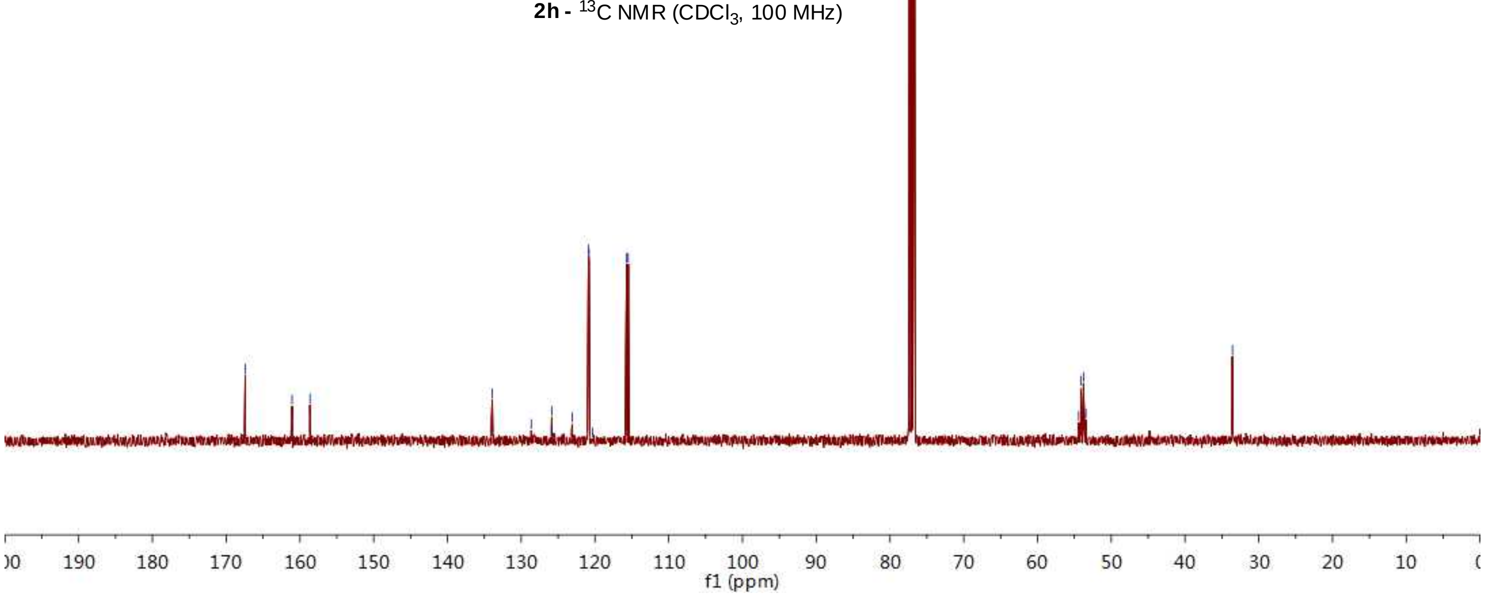

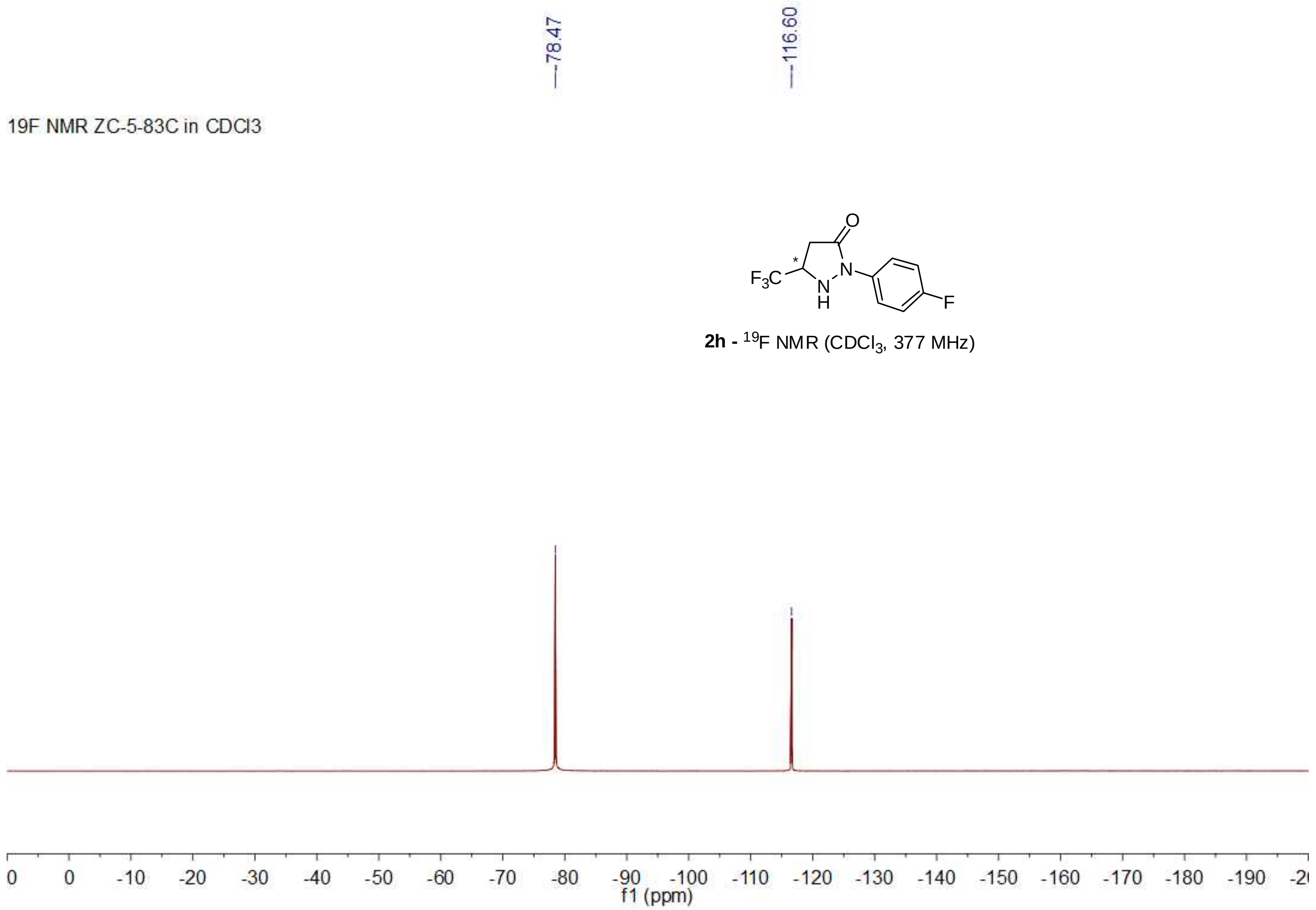

2h - ^{19}F NMR ($CDCl_3$, 377 MHz)

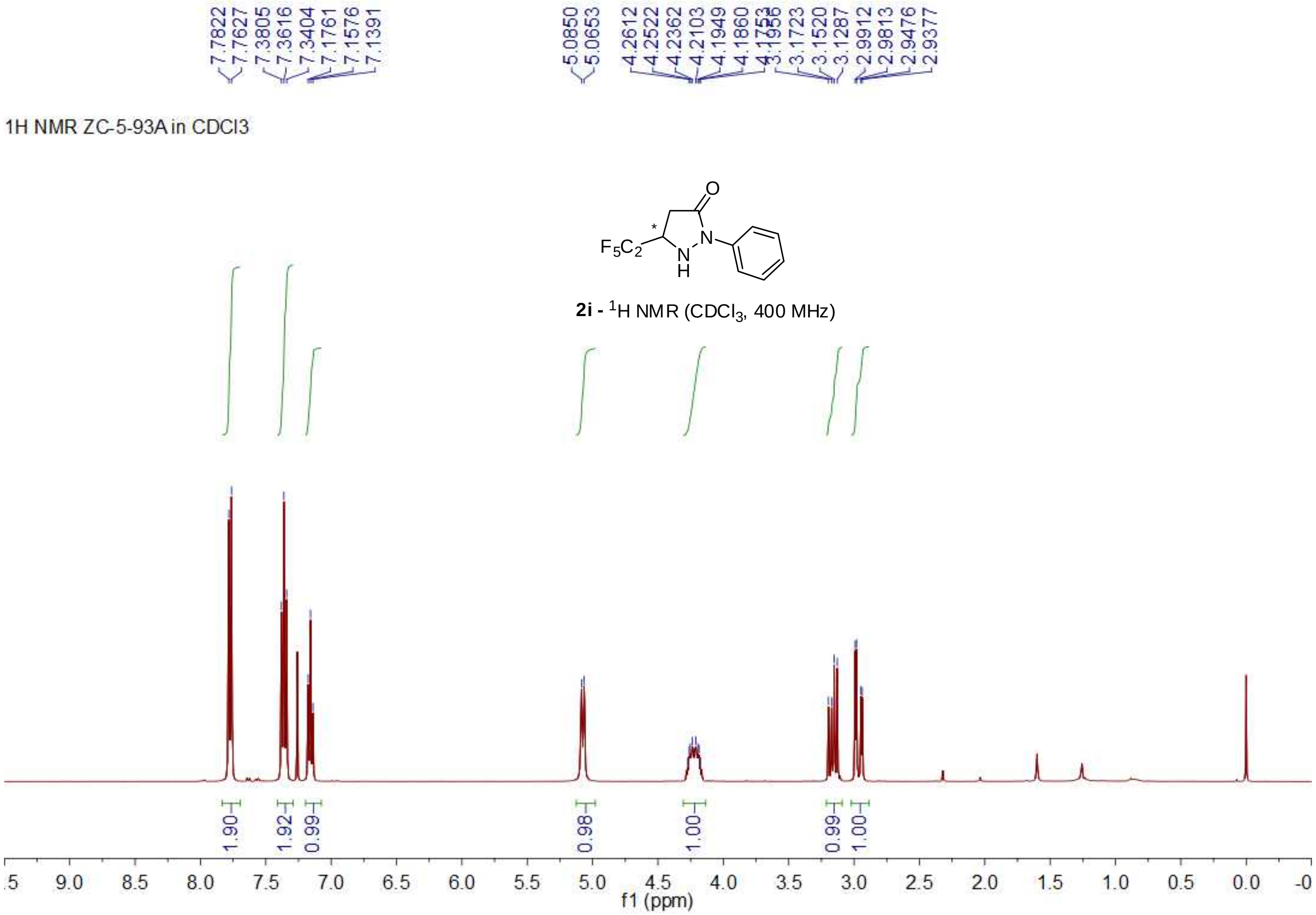
7.7822
7.7627
7.3805
7.3616
7.3404
7.1761
7.1576
7.1391
5.0850
5.0653
4.2612
4.2522
4.2362
4.2103
4.1949
4.1860
3.1723
3.1520
3.1287
2.9912
2.9813
2.9476
2.9377
1H NMR ZC-5-93A in CDCl3
O
F5C2
N
N
H
2i - 1H NMR (CDCl3, 400 MHz)
1.90
1.92
0.99
0.98
1.00
0.99
1.00
9.0
8.5
8.0
7.5
7.0
6.5
6.0
5.5
5.0
4.5
4.0
3.5
3.0
2.5
2.0
1.5
1.0
0.5
0.0
f1 (ppm)

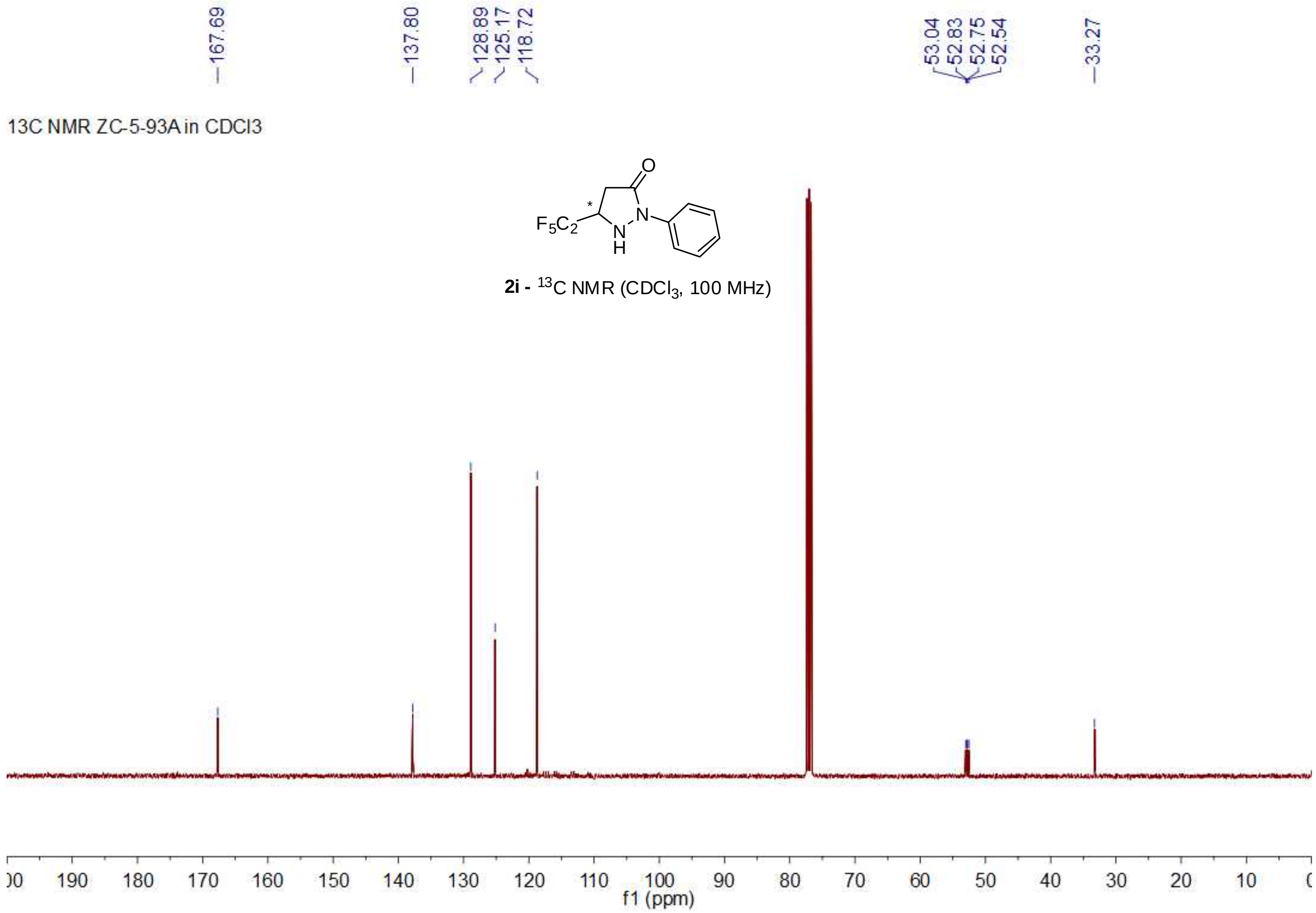
167.69
137.80
128.89
125.17
118.72
53.04
52.83
52.75
52.54
33.27
13C NMR ZC-5-93A in CDCl3
O
F_5C_2
*
N
N
H
2i - ^{13}C NMR ($CDCl_3$, 100 MHz)
f1 (ppm)

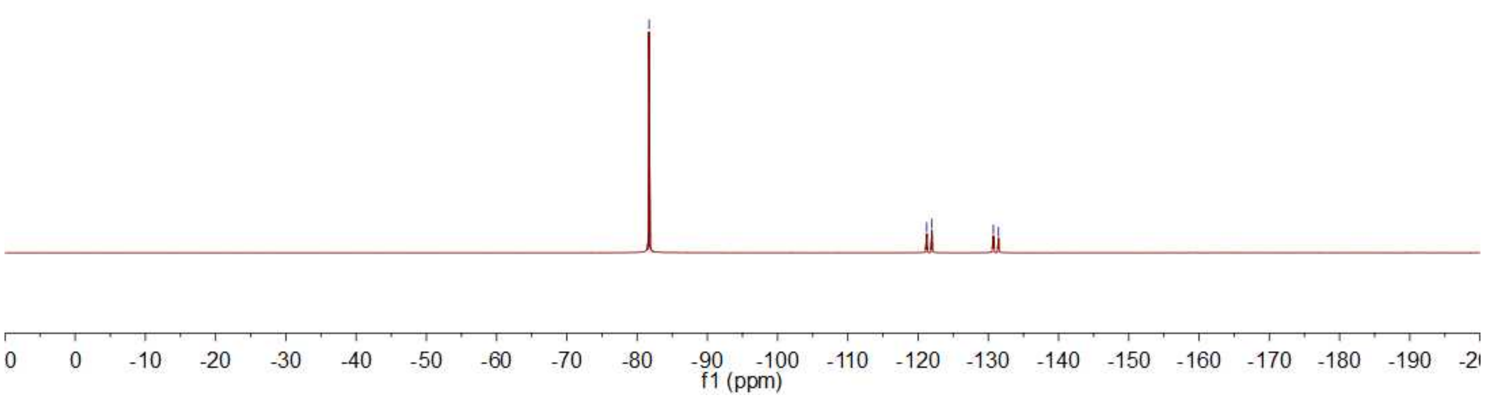

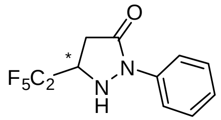

2i - ^{19}F NMR (CDCl$_3$, 377 MHz)

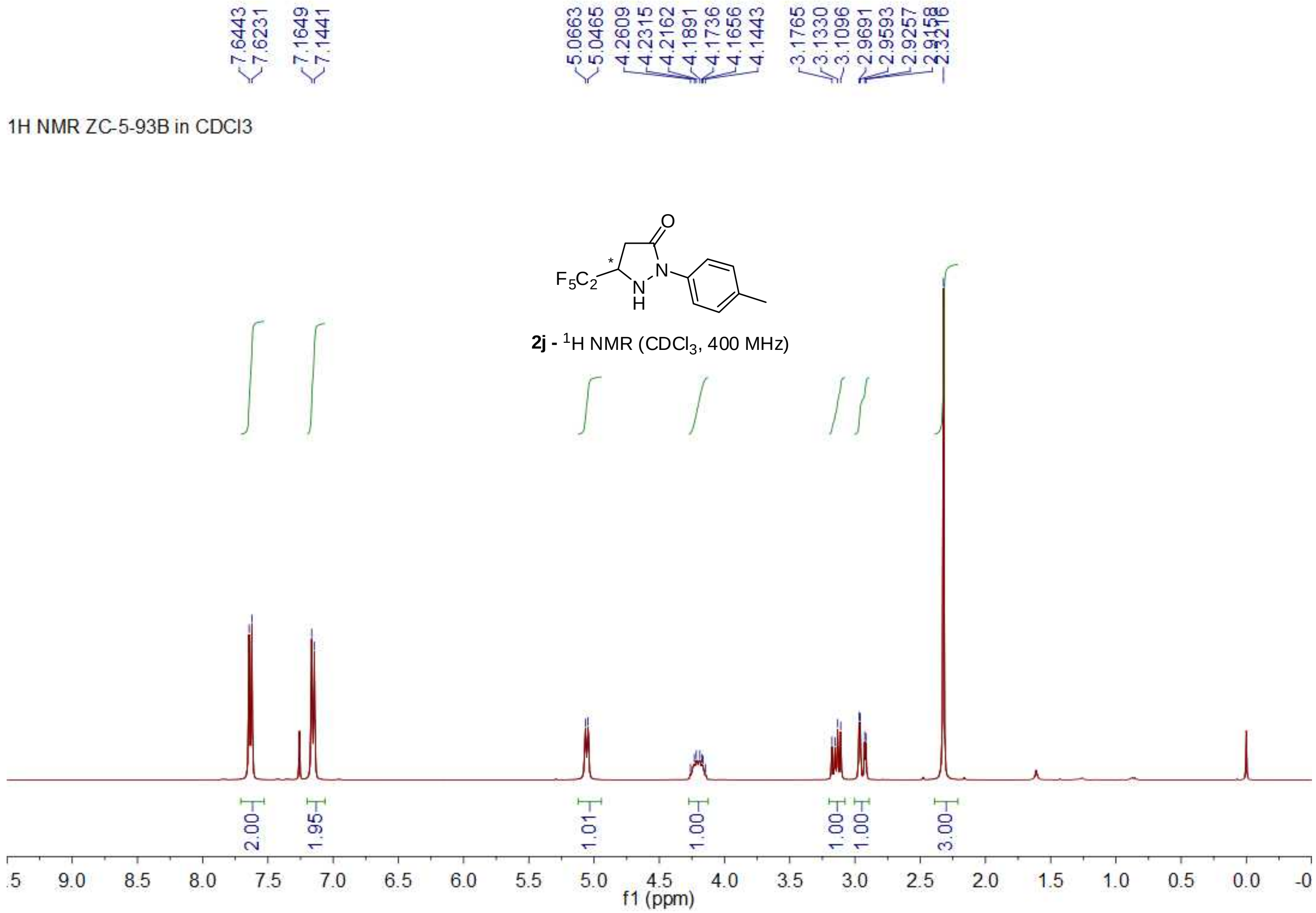
7.6443
7.6231
7.1649
7.1441
5.0663
5.0465
4.2609
4.2315
4.2162
4.1891
4.1736
4.1656
4.1443
3.1765
3.1330
3.1096
2.9691
2.9593
2.9257
2.9158
2.3216
1H NMR ZC-5-93B in CDCl3
O
N
F5C2
*
N
H
2j - 1H NMR (CDCl3, 400 MHz)
2.00
1.95
1.01
1.00
1.00
1.00
3.00
.5
9.0
8.5
8.0
7.5
7.0
6.5
6.0
5.5
5.0
4.5
4.0
3.5
3.0
2.5
2.0
1.5
1.0
0.5
0.0
-0
f1 (ppm)

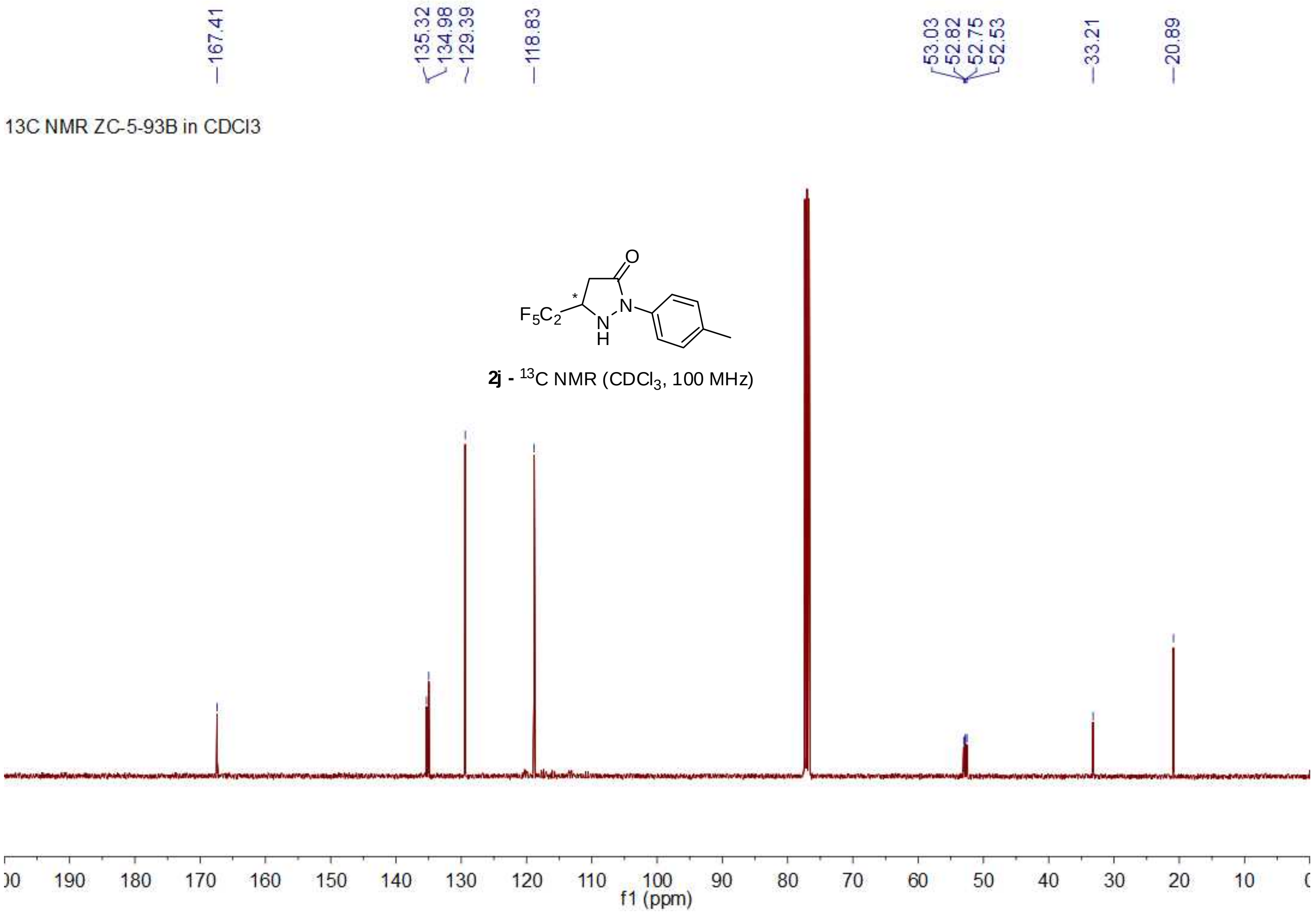
167.41
135.32
134.98
129.39
118.83
53.03
52.82
52.75
52.53
33.21
20.89
13C NMR ZC-5-93B in CDCl3
O
N
N
H
F_5C_2
*
2j - ^{13}C NMR ($CDCl_3$, 100 MHz)
190
180
170
160
150
140
130
120
110
100
90
80
70
60
50
40
30
20
10
f1 (ppm)

19F NMR ZC-5-93B in CDCl3

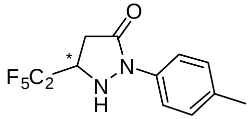

2 j - ^{19}F NMR ($CDCl_3$, 377 MHz)

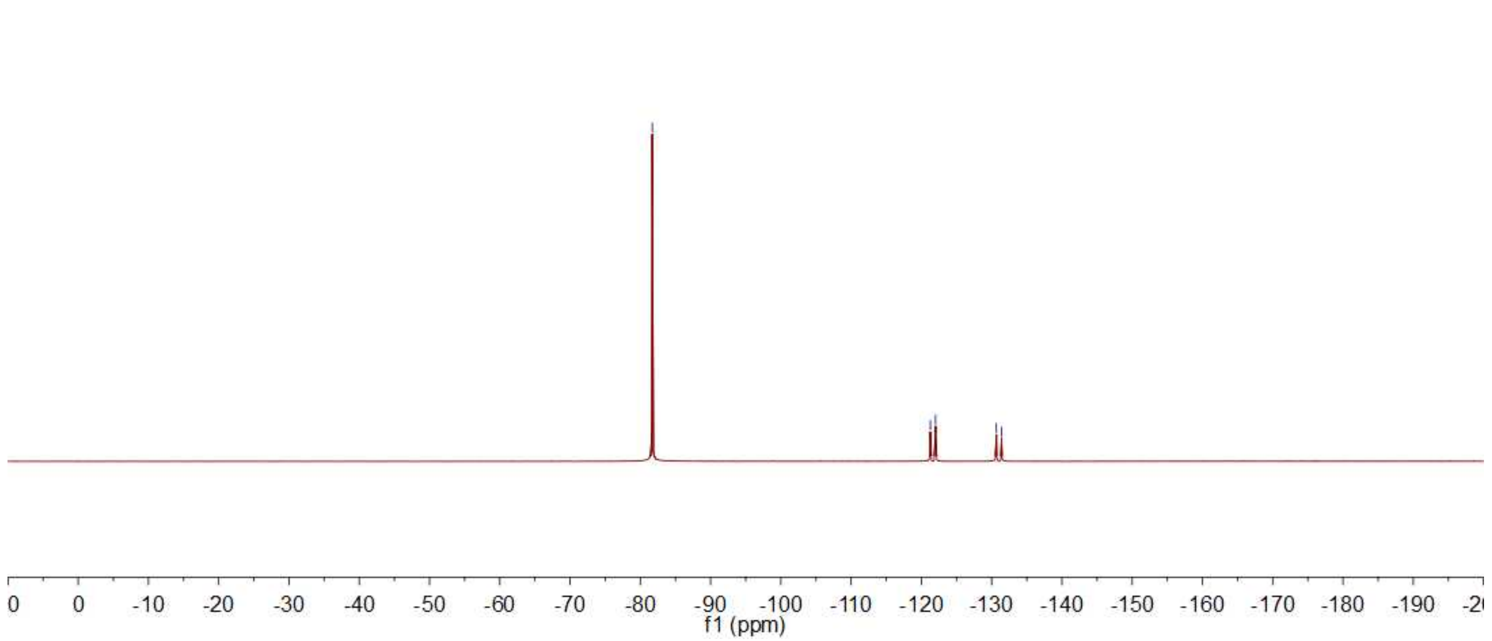

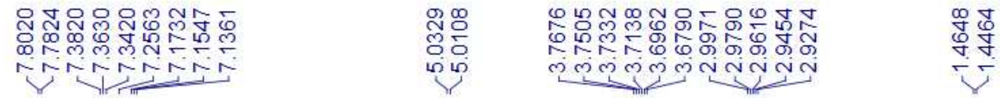

1H NMR ZC-5-86F in CDCl3

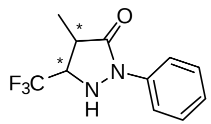

4a - ^{1}H NMR ($CDCl_3$, 400 MHz)

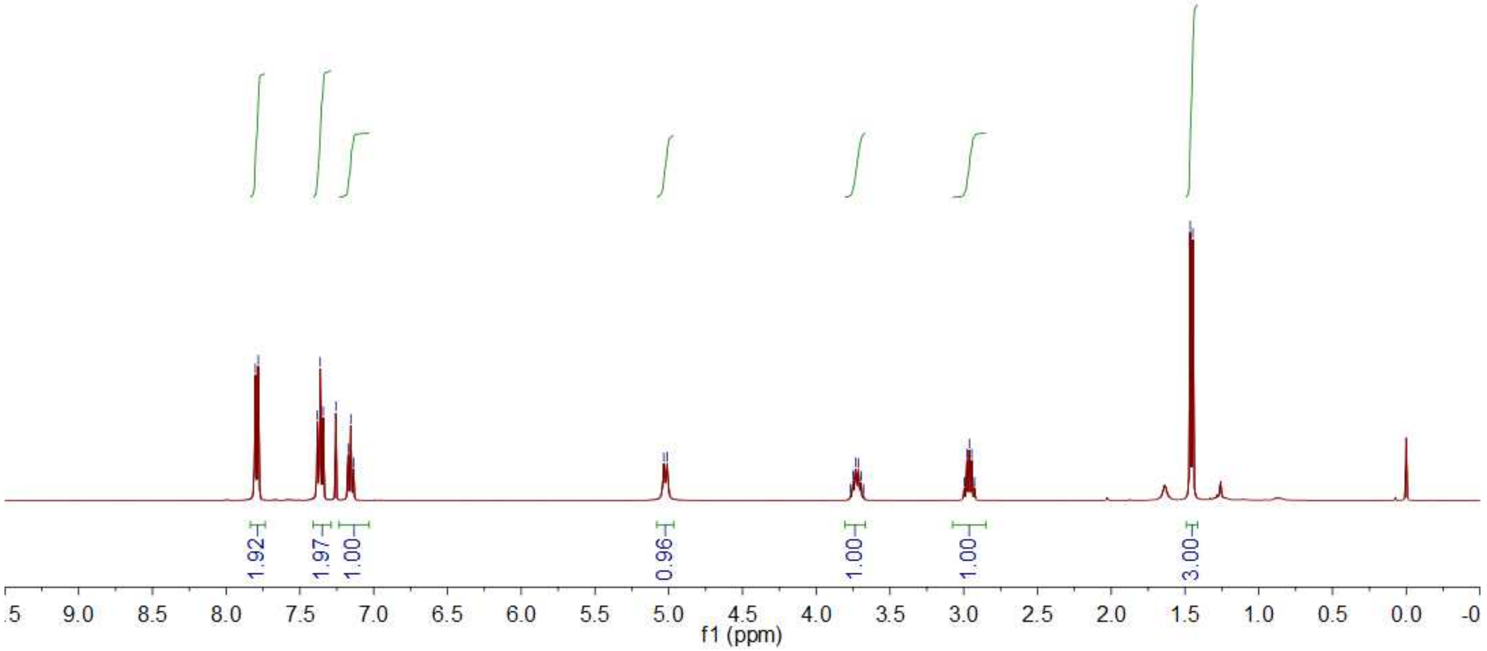

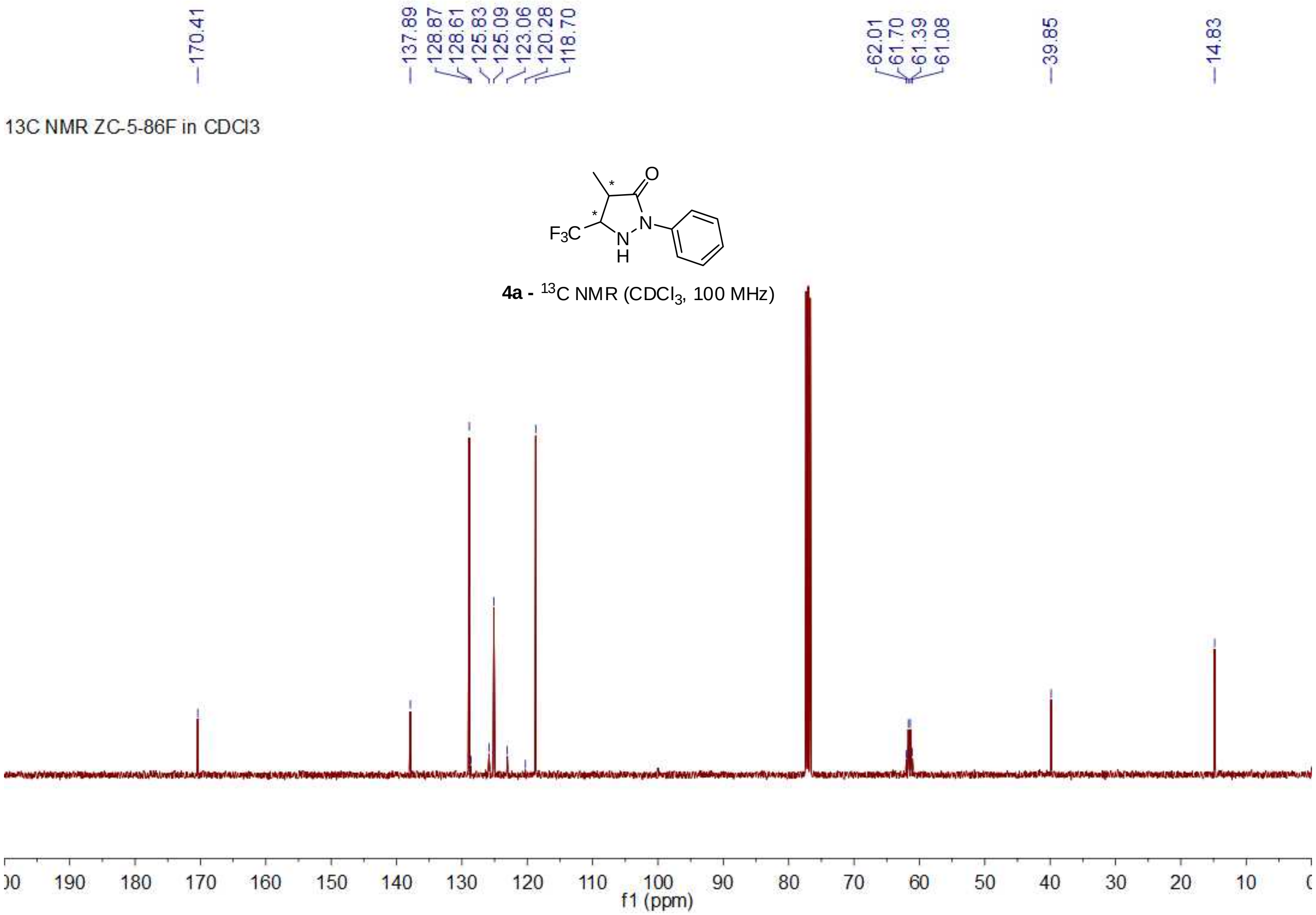

4a - ^{13}C NMR ($CDCl_3$, 100 MHz)

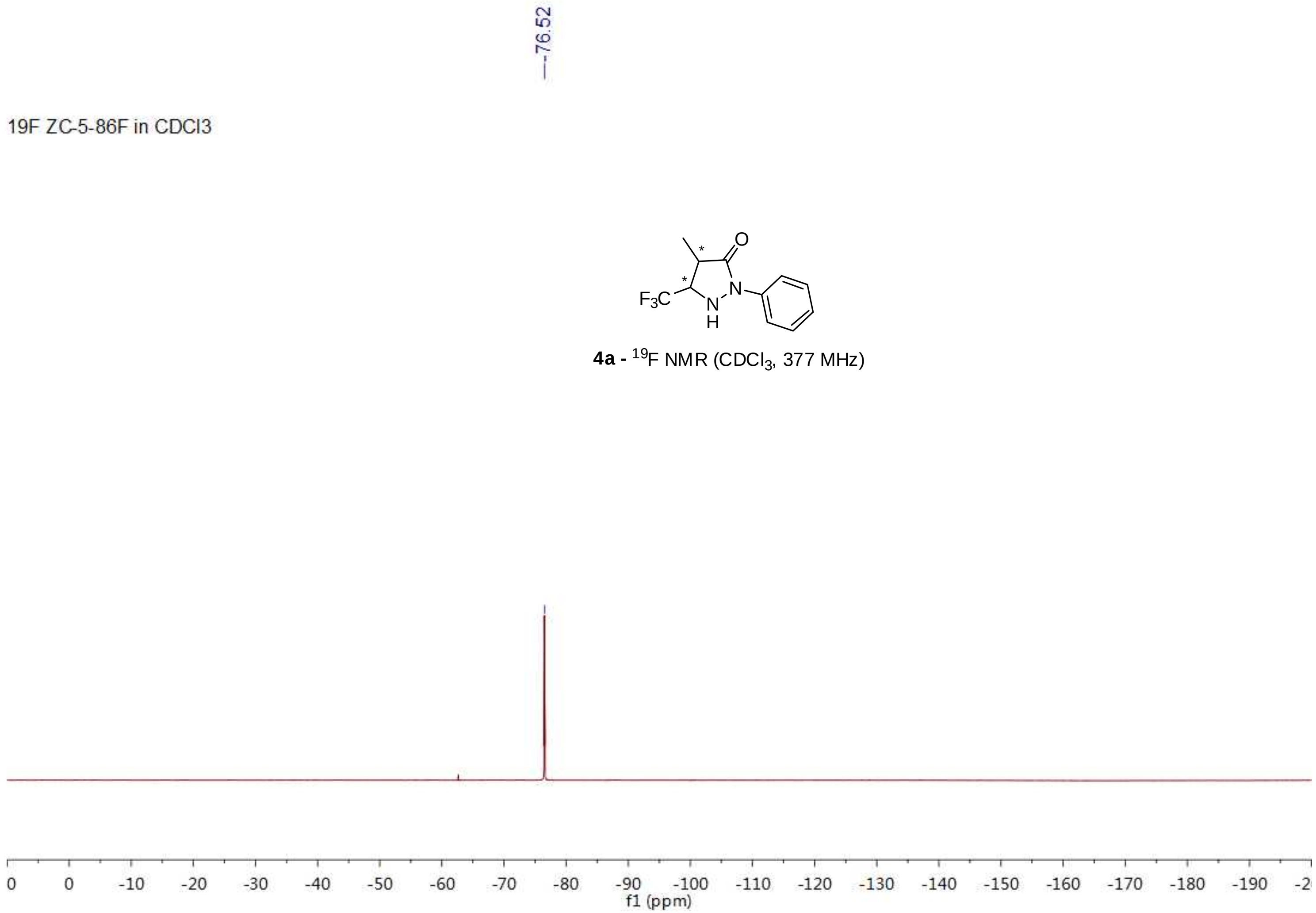

4a - ^{19}F NMR ($CDCl_3$, 377 MHz)

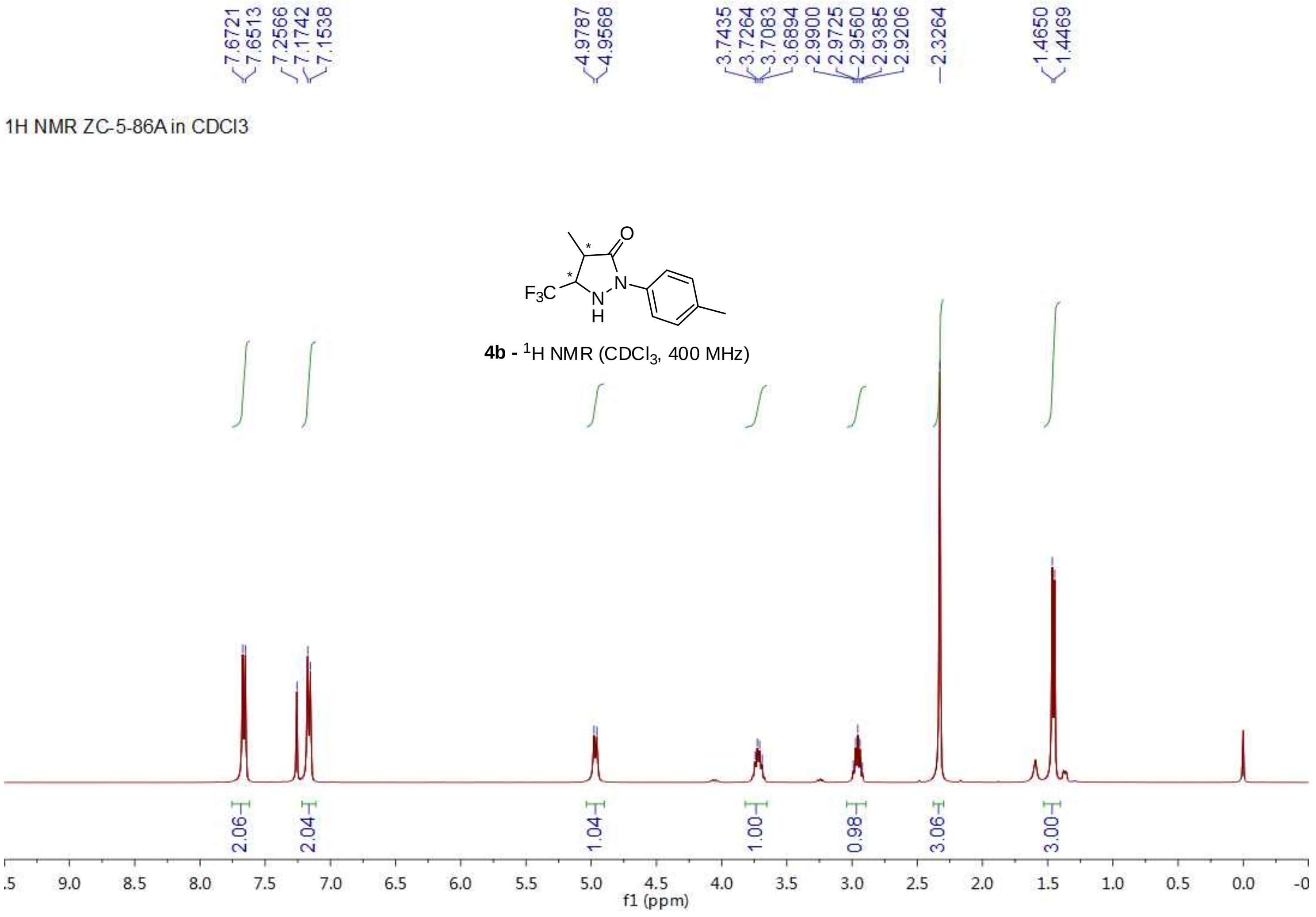

7.6721
7.6513
7.2566
7.1742
7.1538
4.9787
4.9568
3.7435
3.7264
3.7083
3.6894
2.9900
2.9725
2.9560
2.9385
2.9206
2.3264
1.4650
1.4469
1H NMR ZC-5-86A in CDCl3
O
N
N
H
F3C
4b - ¹H NMR (CDCl3, 400 MHz)
2.06
2.04
1.04
1.00
0.98
3.06
3.00
f1 (ppm)

13C NMR ZC-5-86A in CDCl3

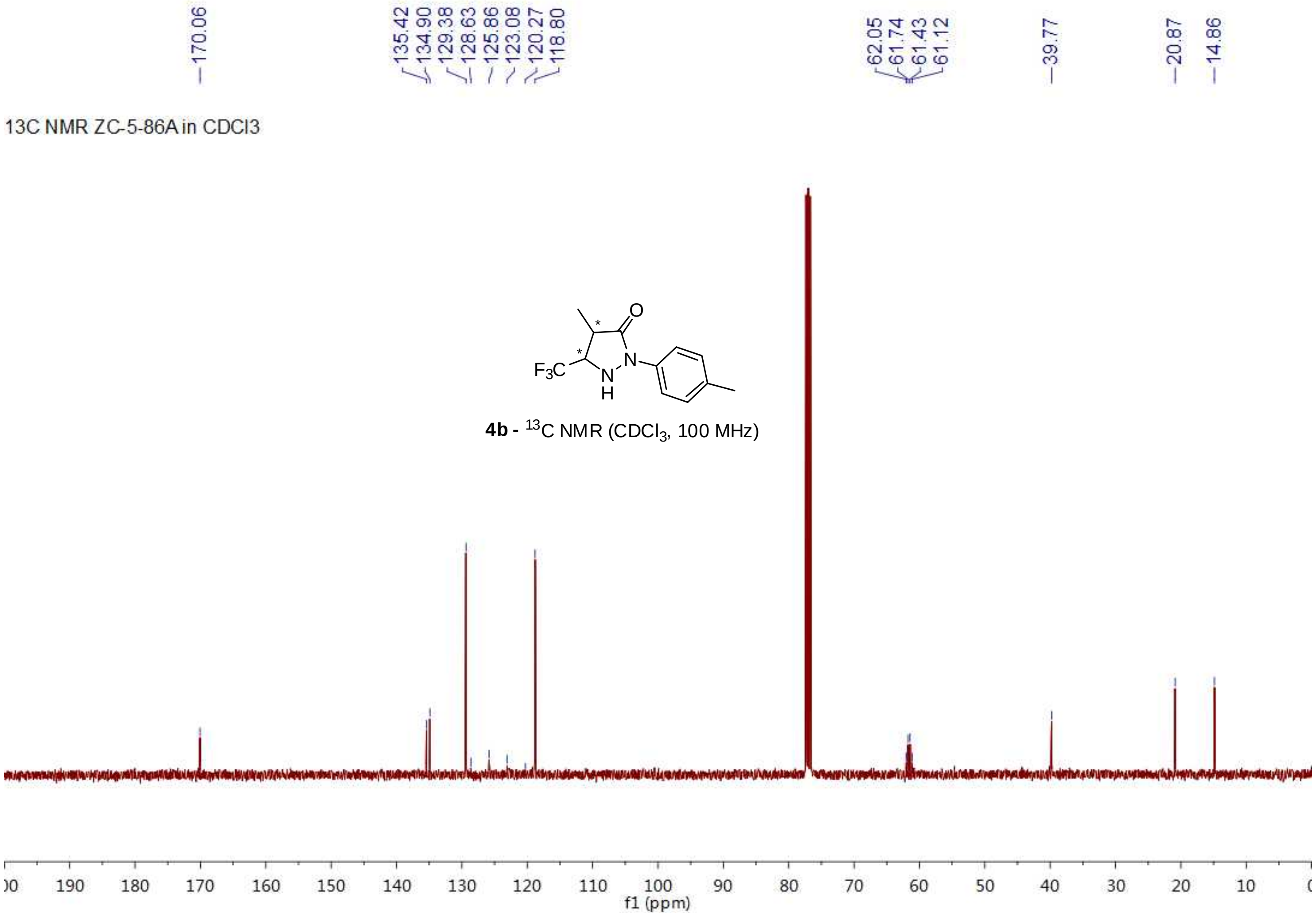

4b - ^{13}C NMR ($CDCl_3$, 100 MHz)

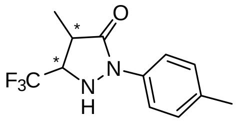

4b - ^{19}F NMR ($CDCl_3$, 377 MHz)

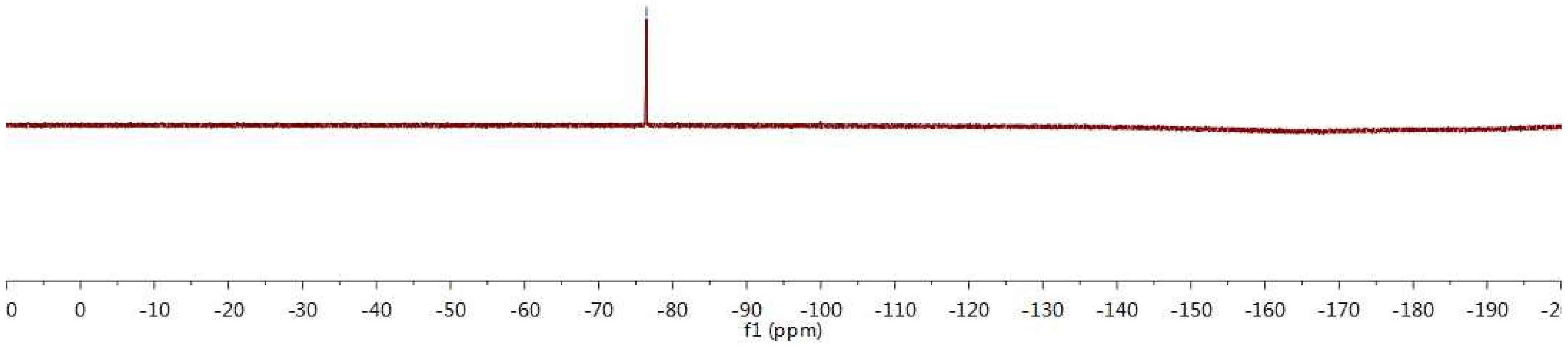

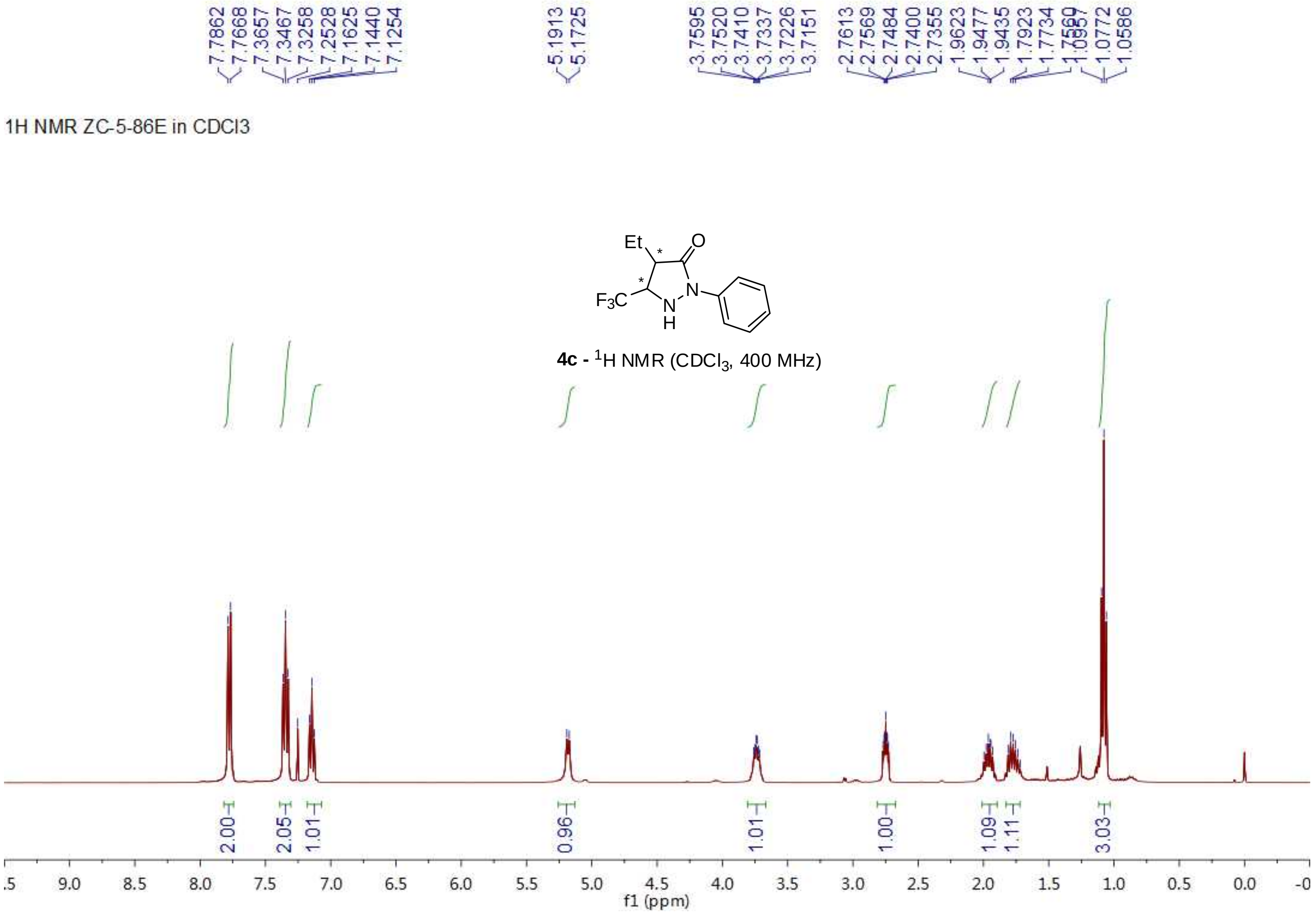
1H NMR ZC-5-86E in CDCl3
7.7862
7.7668
7.3657
7.3467
7.3258
7.2528
7.1625
7.1440
7.1254
5.1913
5.1725
3.7595
3.7520
3.7410
3.7337
3.7226
3.7151
2.7613
2.7569
2.7484
2.7400
2.7355
1.9623
1.9477
1.9435
1.7923
1.7734
1.0957
1.0772
1.0586
Et
O
N
F3C
N
H
4c - 1H NMR (CDCl3, 400 MHz)
2.00
2.05
1.01
0.96
1.01
1.00
1.09
1.11
3.03
9.0
8.5
8.0
7.5
7.0
6.5
6.0
5.5
5.0
4.5
4.0
3.5
3.0
2.5
2.0
1.5
1.0
0.5
0.0
f1 (ppm)

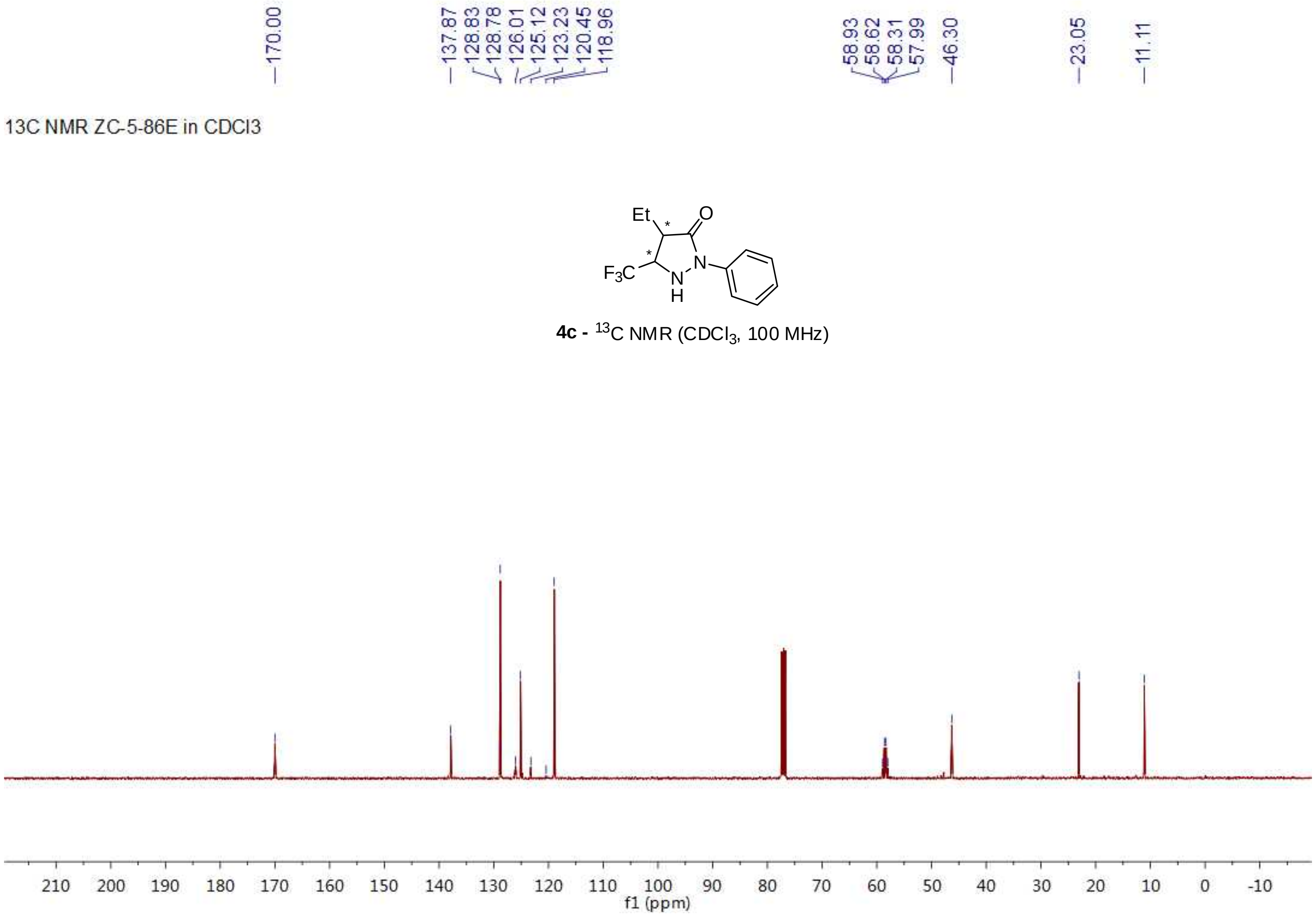

4c - ^{13}C NMR ($CDCl_3$, 100 MHz)

19F NMR ZC-5-86E in CDCl3

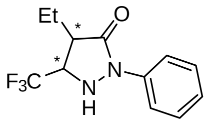

4c - ^{19}F NMR ($CDCl_3$, 377 MHz)

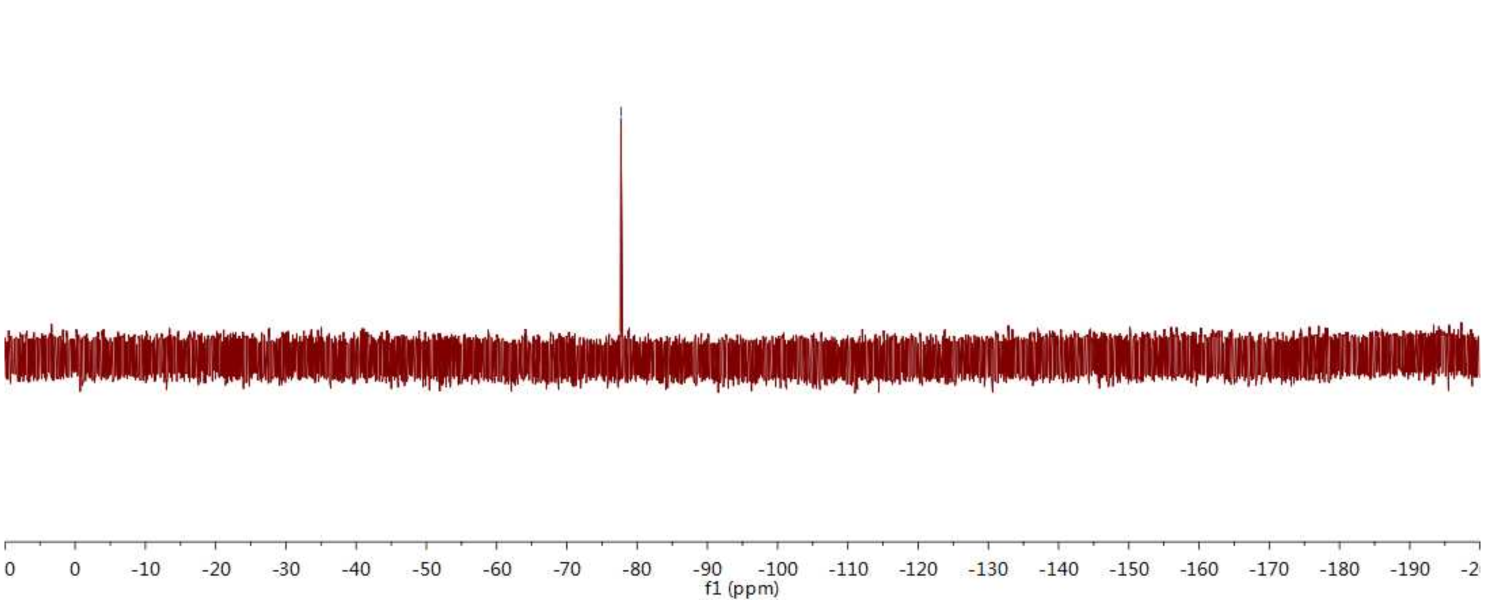

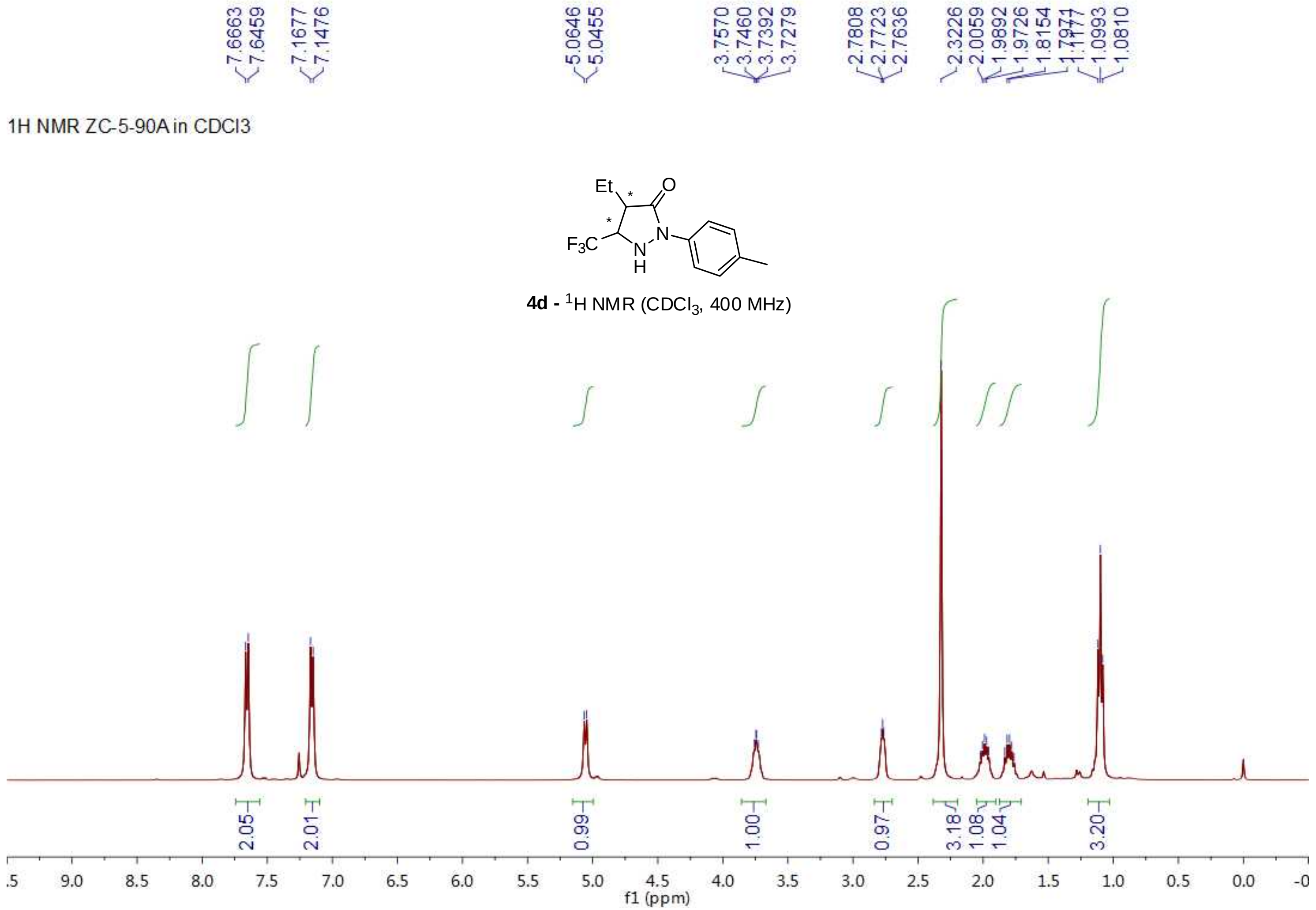
7.6663
7.6459
7.1677
7.1476
5.0646
5.0455
3.7570
3.7460
3.7392
3.7279
2.7808
2.7723
2.7636
2.3226
2.0059
1.9892
1.9726
1.8154
1.7971
1.1177
1.0993
1.0810
1H NMR ZC-5-90A in CDCl3
Et
O
F3C
N
N
H
4d - 1H NMR (CDCl3, 400 MHz)
2.05
2.01
0.99
1.00
0.97
3.18
1.08
1.04
3.20
f1 (ppm)

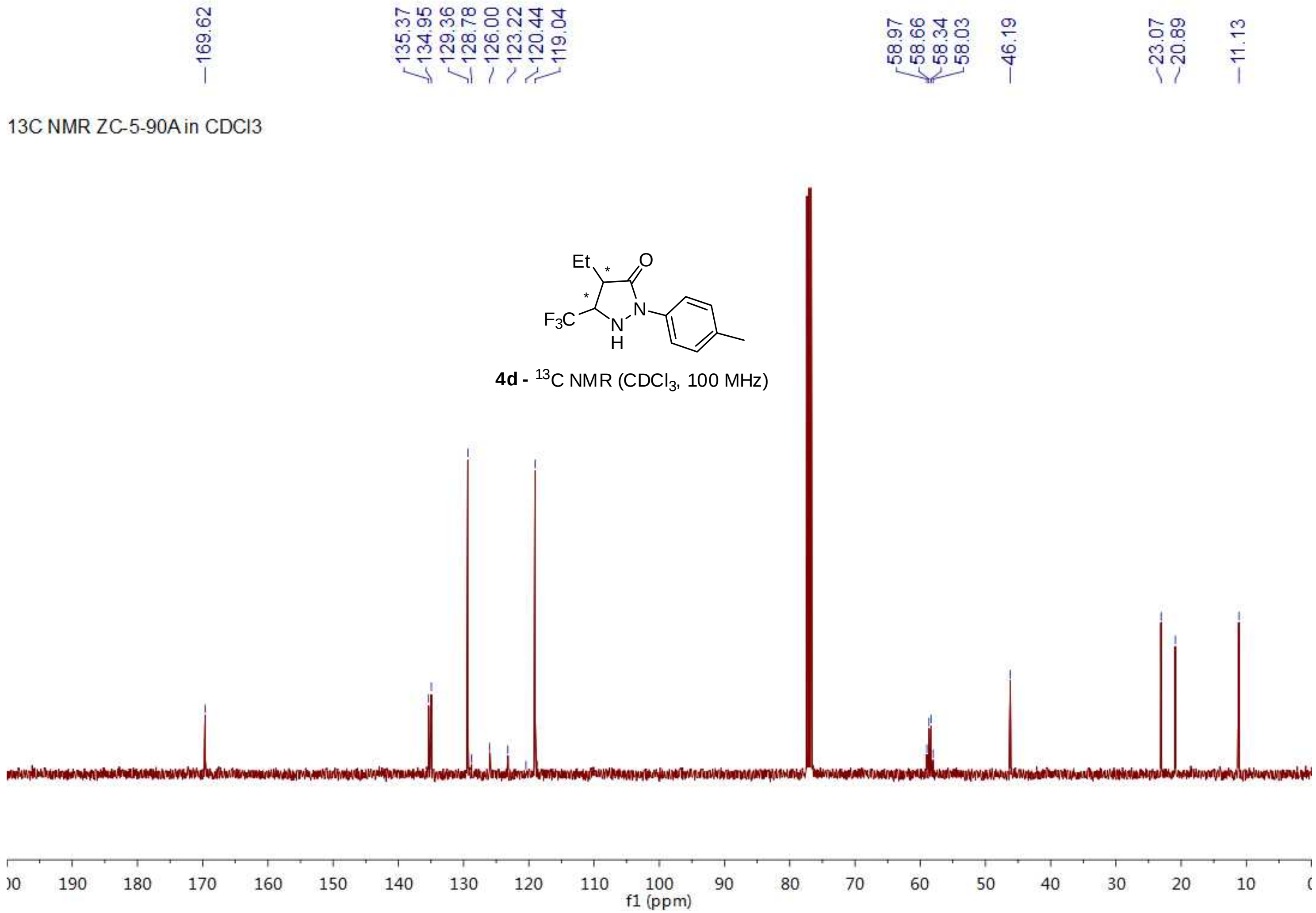

169.62
135.37
134.95
129.36
128.78
126.00
123.22
120.44
119.04
58.97
58.66
58.34
58.03
46.19
23.07
20.89
11.13
13C NMR ZC-5-90A in CDCl3
Et
O
F3C
N
N
H
4d - 13C NMR (CDCl3, 100 MHz)
190
180
170
160
150
140
130
120
110
100
90
80
70
60
50
40
30
20
10
f1 (ppm)

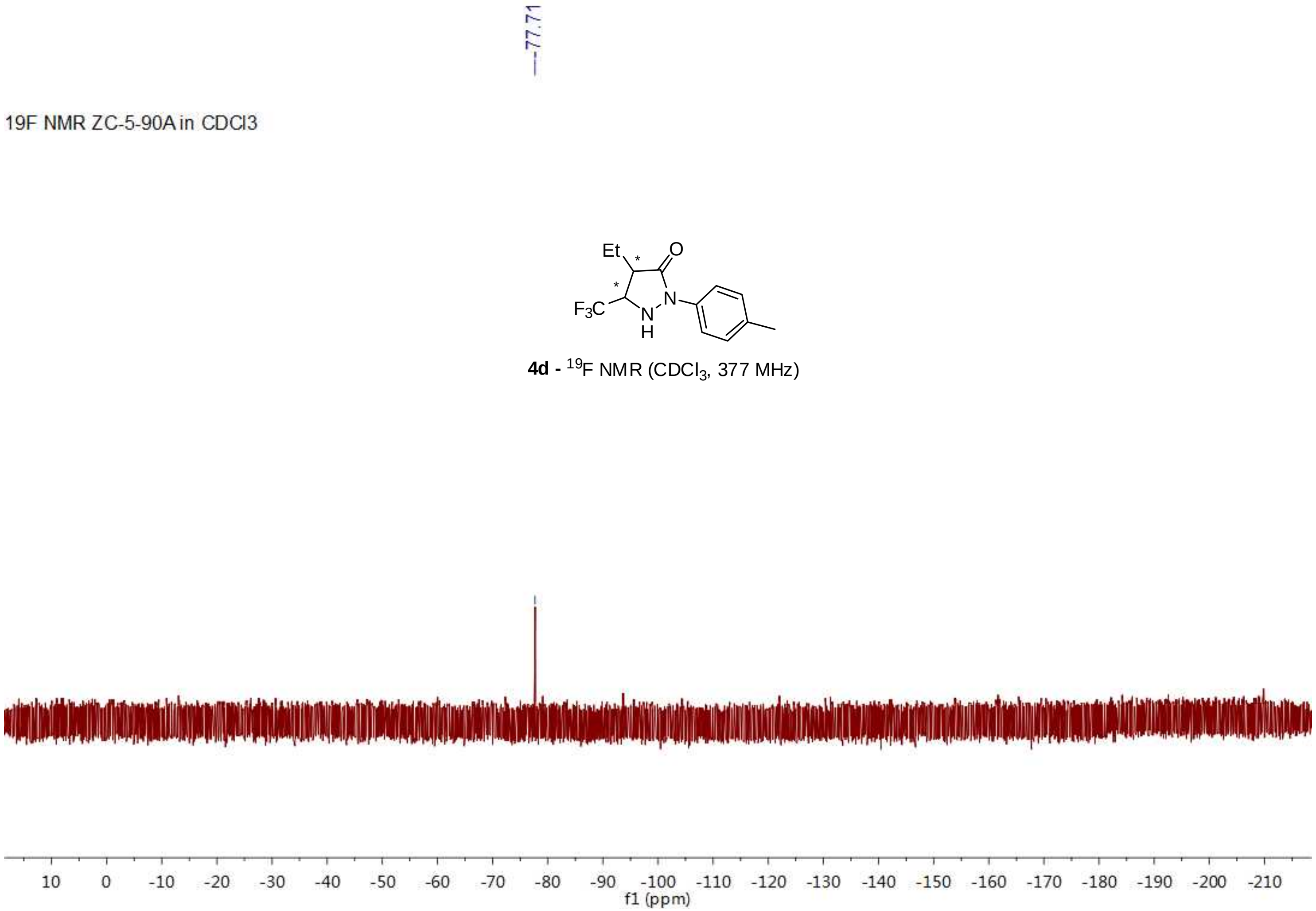

4d - ^{19}F NMR ($CDCl_3$, 377 MHz)

1H NMR ZC-5-90B in CDCl3

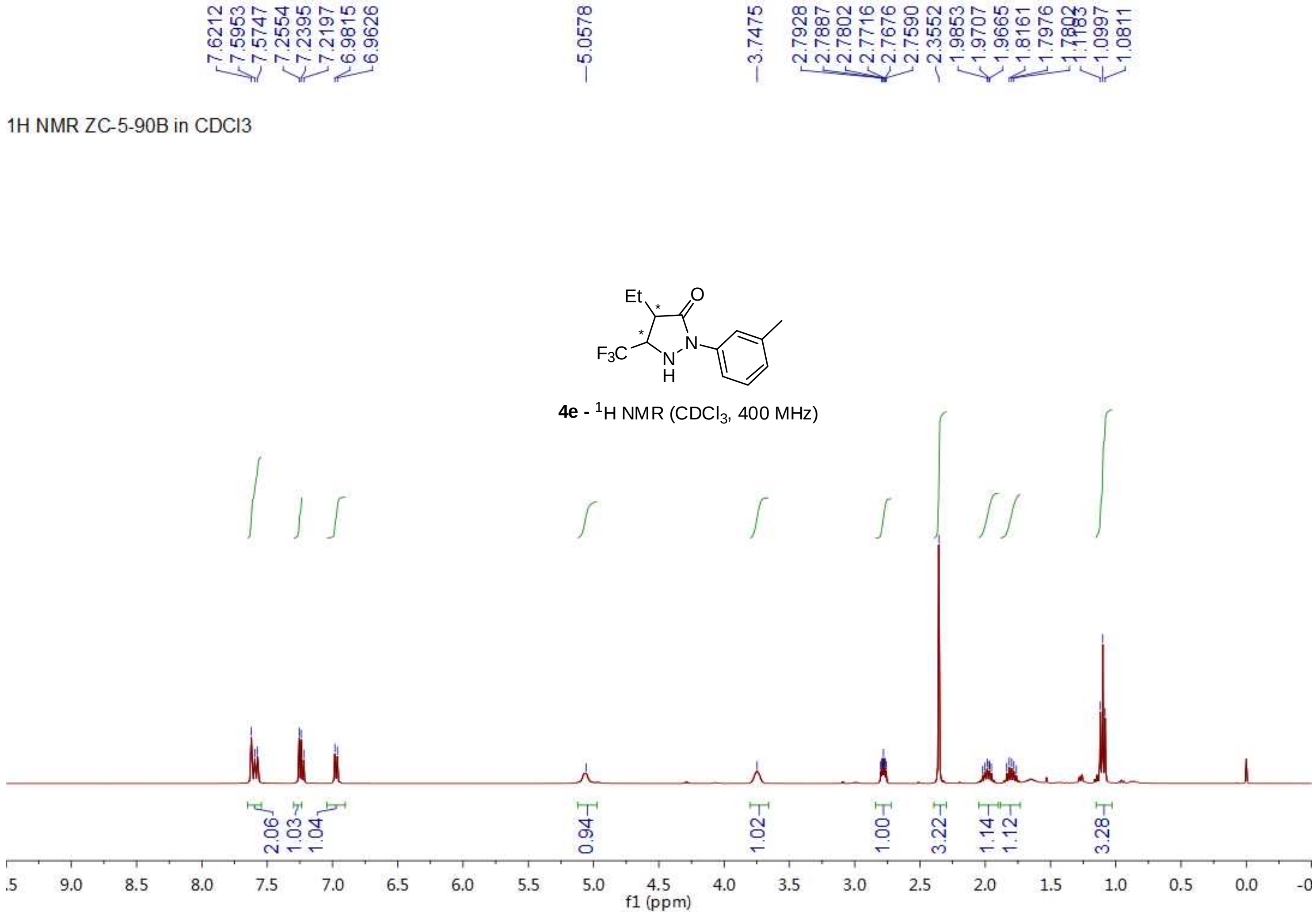

4e - ^{1}H NMR ($CDCl_3$, 400 MHz)

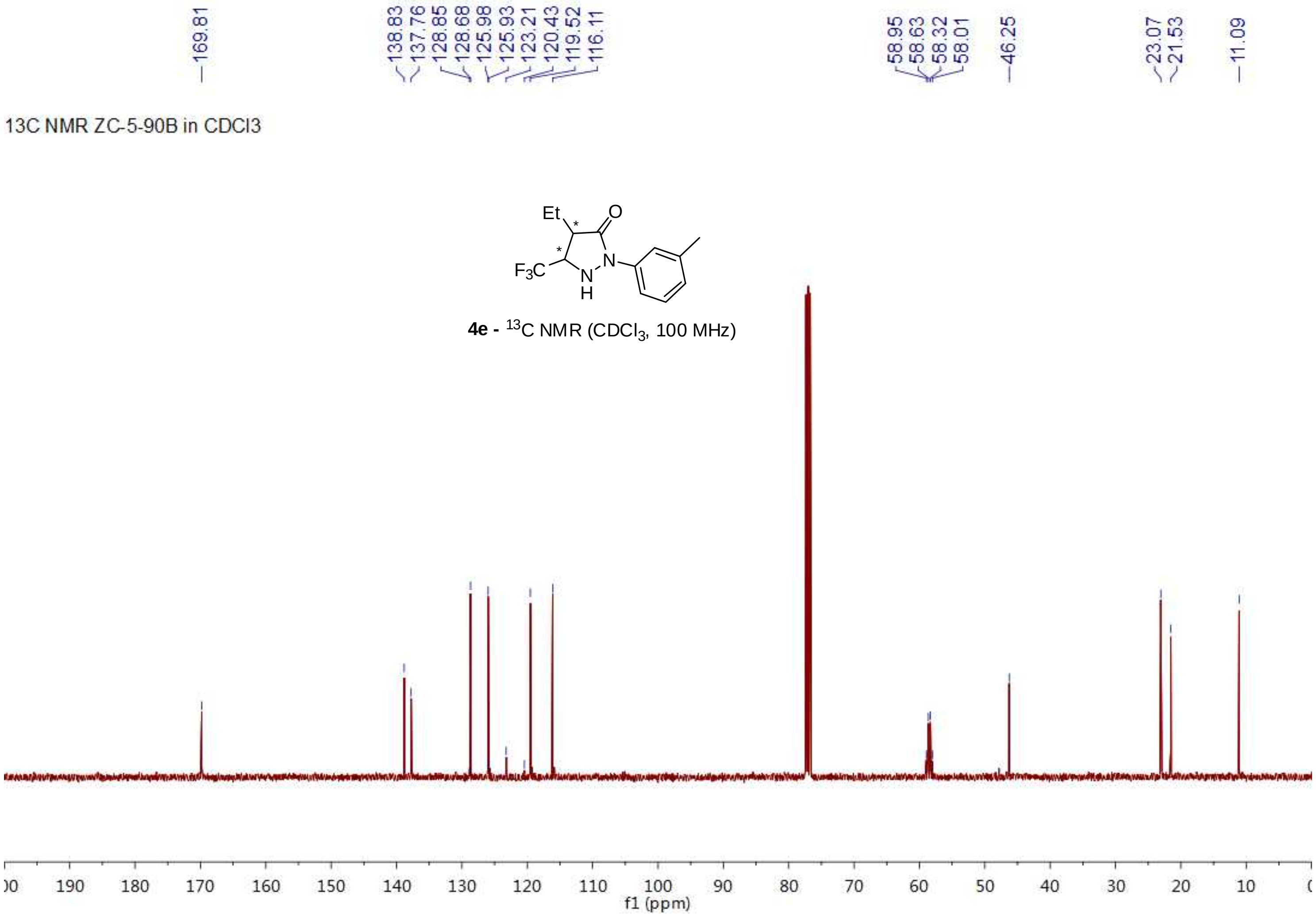
169.81
138.83
137.76
128.85
128.68
125.98
125.93
123.21
120.43
119.52
116.11
58.95
58.63
58.32
58.01
46.25
23.07
21.53
11.09
13C NMR ZC-5-90B in CDCl3
Et
O
F3C
N
N
H
4e - 13C NMR (CDCl3, 100 MHz)
190
180
170
160
150
140
130
120
110
100
90
80
70
60
50
40
30
20
10
f1 (ppm)

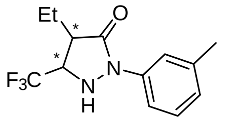

4e - ^{19}F NMR ($CDCl_3$, 377 MHz)

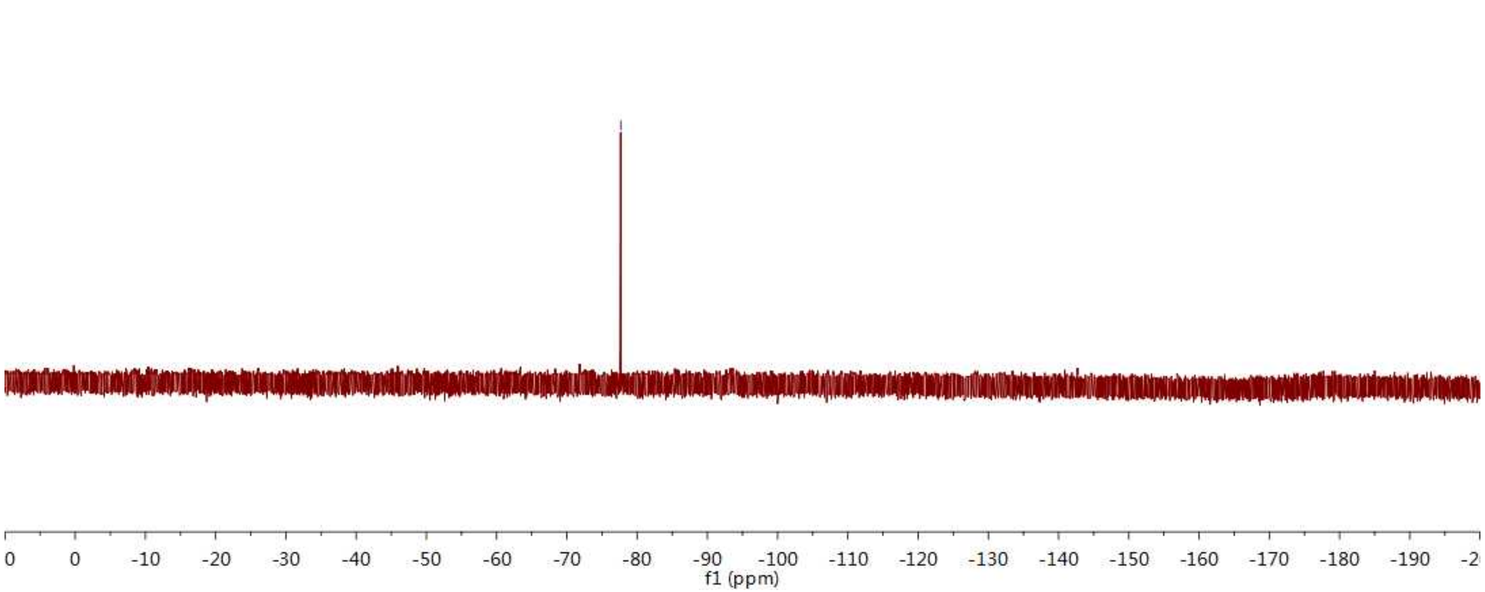

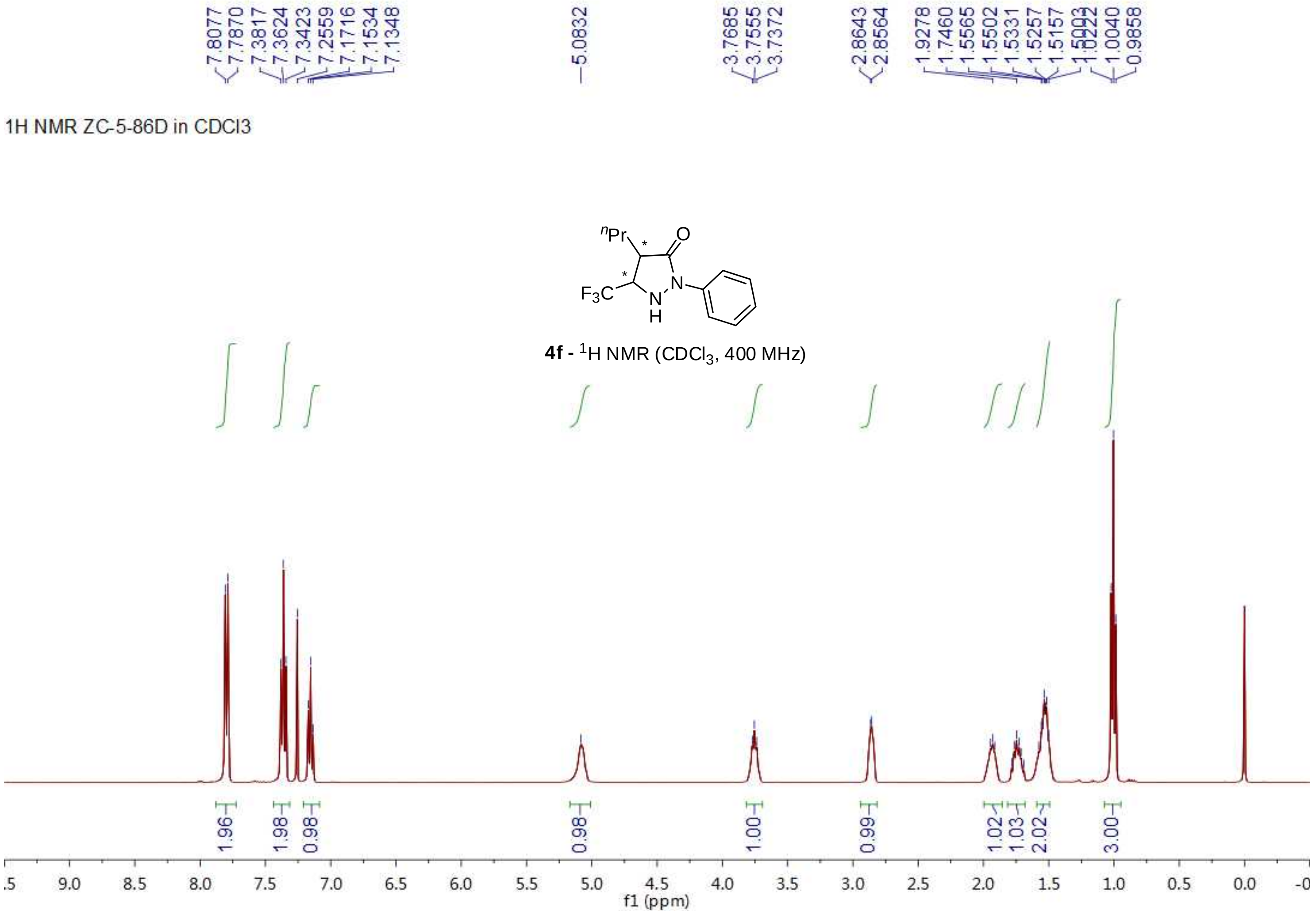

4f - ¹H NMR ($CDCl_3$, 400 MHz)

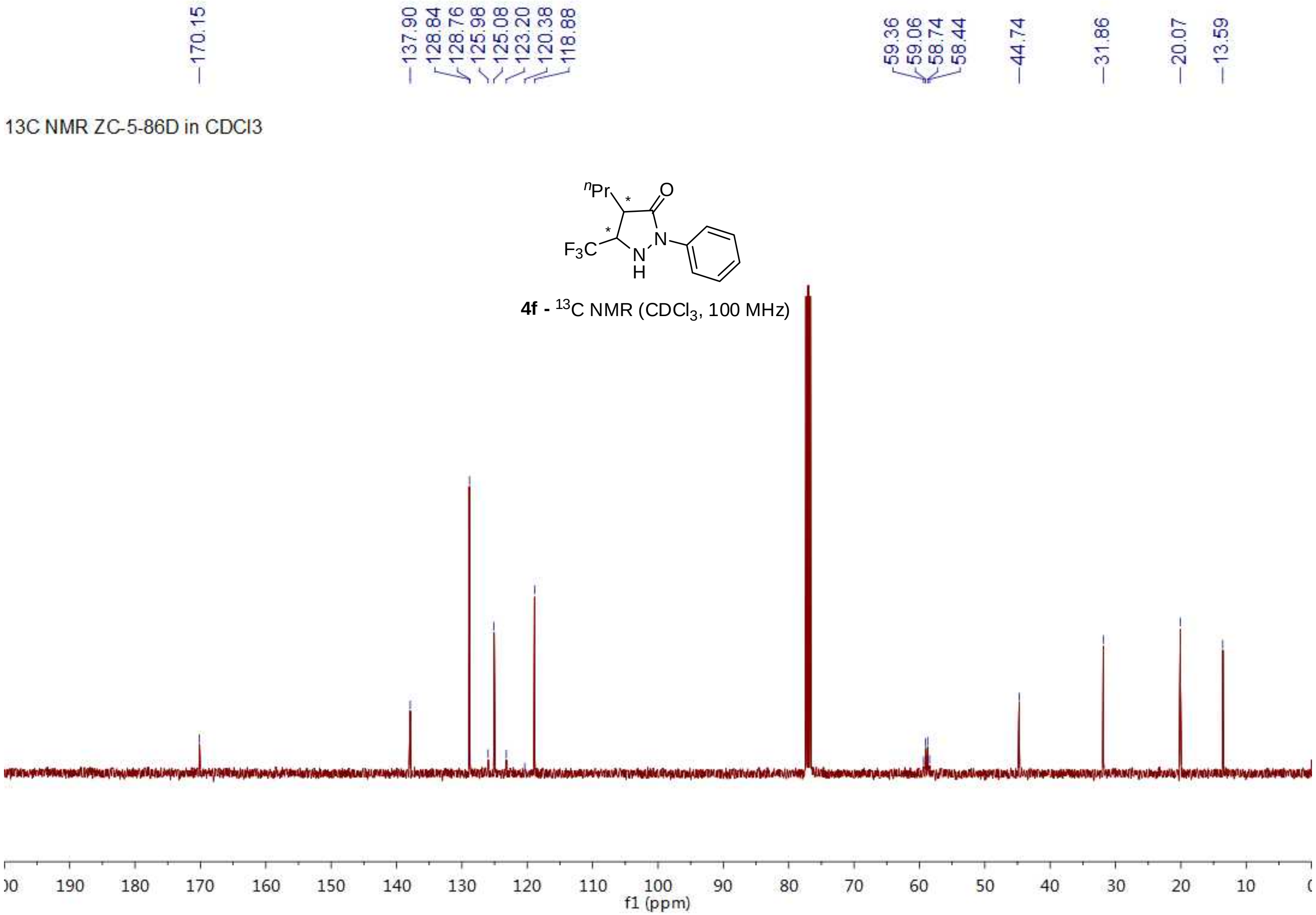

170.15
137.90
128.84
128.76
125.98
125.08
123.20
120.38
118.88
59.36
59.06
58.74
58.44
44.74
31.86
20.07
13.59
13C NMR ZC-5-86D in CDCl3
4f - 13C NMR (CDCl3, 100 MHz)
190
180
170
160
150
140
130
120
110
100
90
80
70
60
50
40
30
20
10
f1 (ppm)

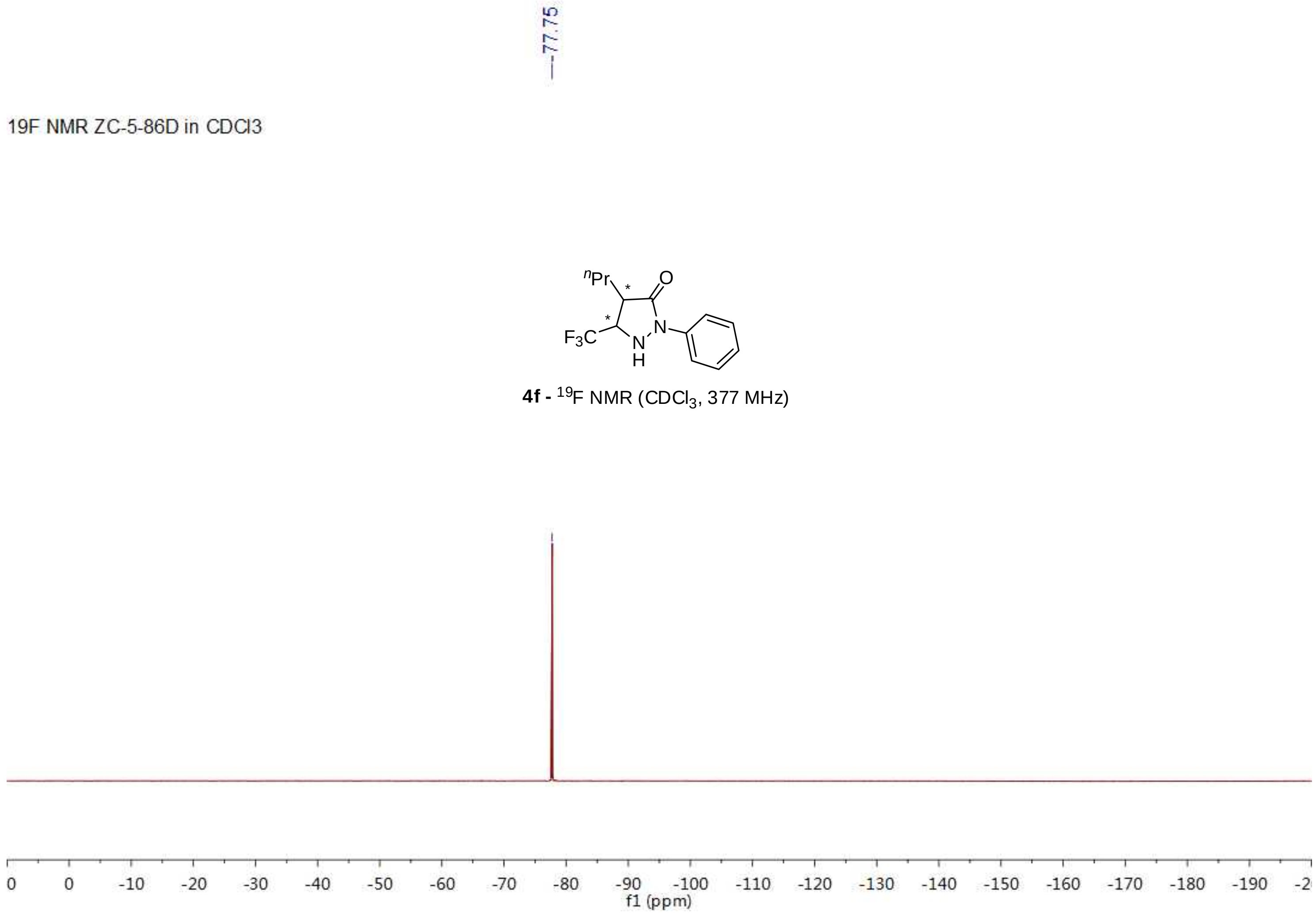

4f - ^{19}F NMR ($CDCl_3$, 377 MHz)

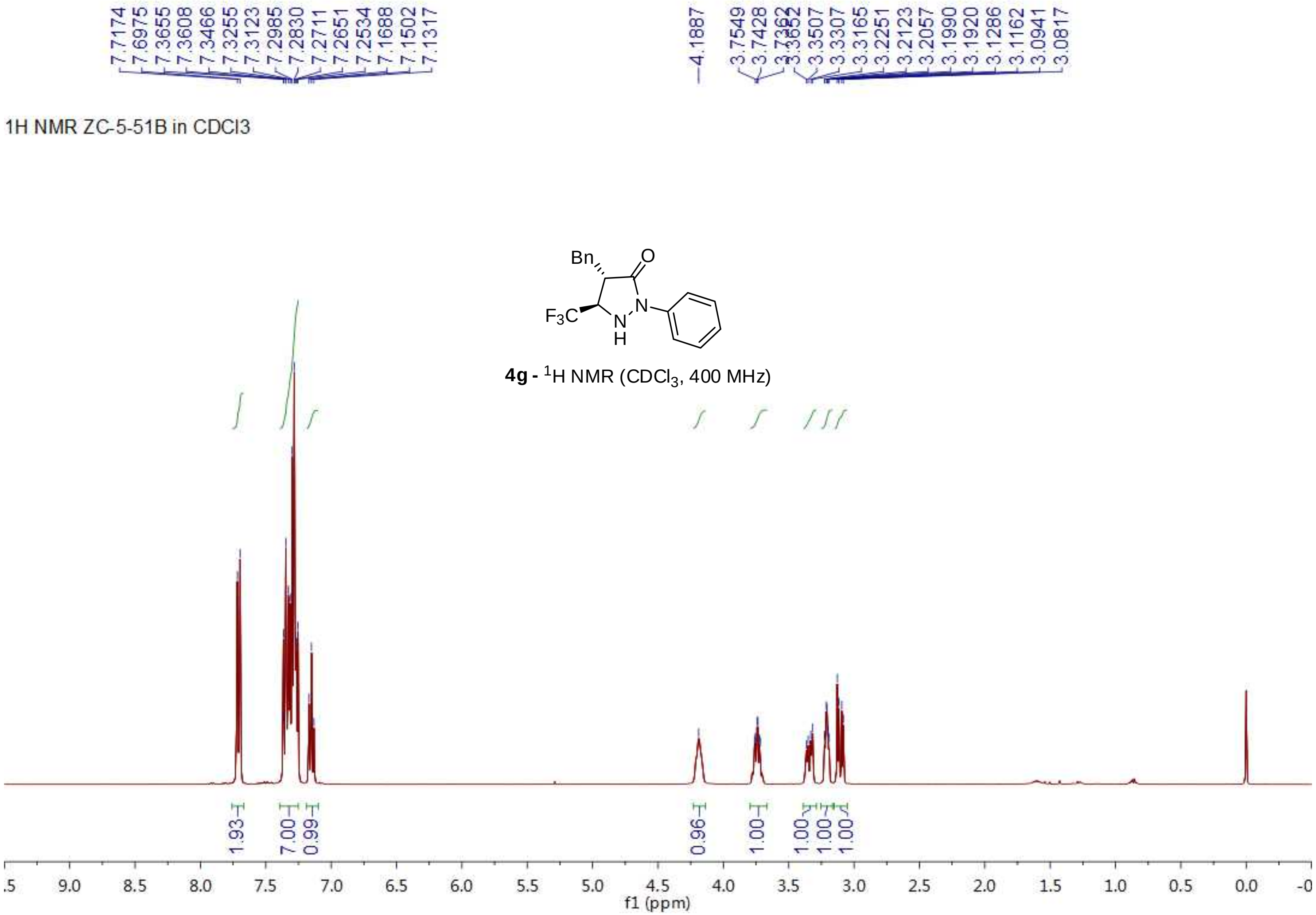

7.7174
7.6975
7.3655
7.3608
7.3466
7.3255
7.3123
7.2985
7.2830
7.2711
7.2651
7.2534
7.1688
7.1502
7.1317
4.1887
3.7549
3.7428
3.7362
3.3652
3.3507
3.3307
3.3165
3.2251
3.2123
3.2057
3.1990
3.1920
3.1286
3.1162
3.0941
3.0817
1H NMR ZC-5-51B in CDCl3
Bn
O
F3C
N
N
H
4g - 1H NMR (CDCl3, 400 MHz)
1.93
7.00
0.99
0.96
1.00
1.00
1.00
1.00
f1 (ppm)

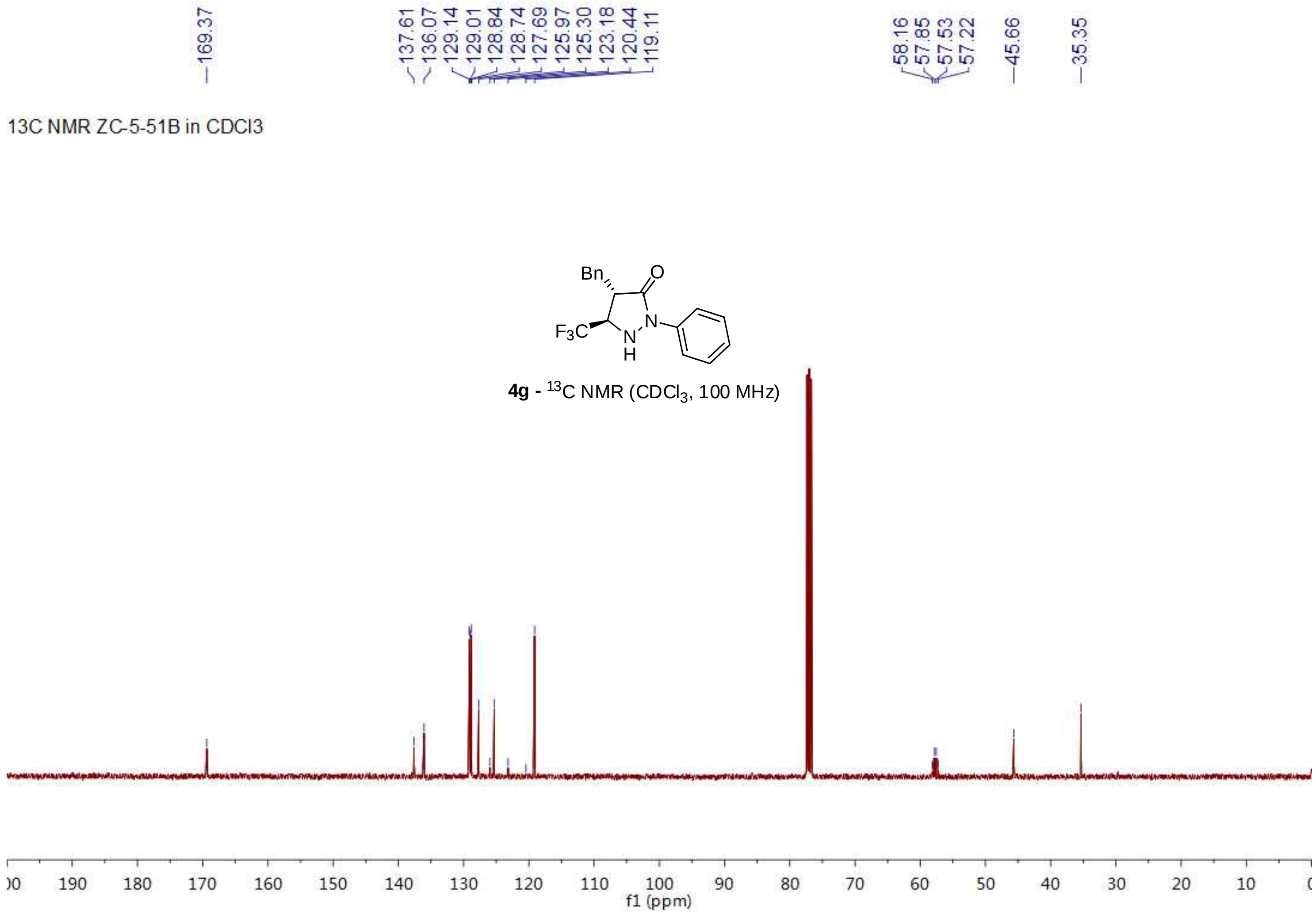

4g - ^{13}C NMR ($CDCl_3$, 100 MHz)

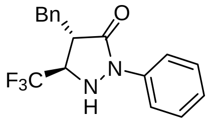

4g - ^{19}F NMR ($CDCl_3$, 377 MHz)

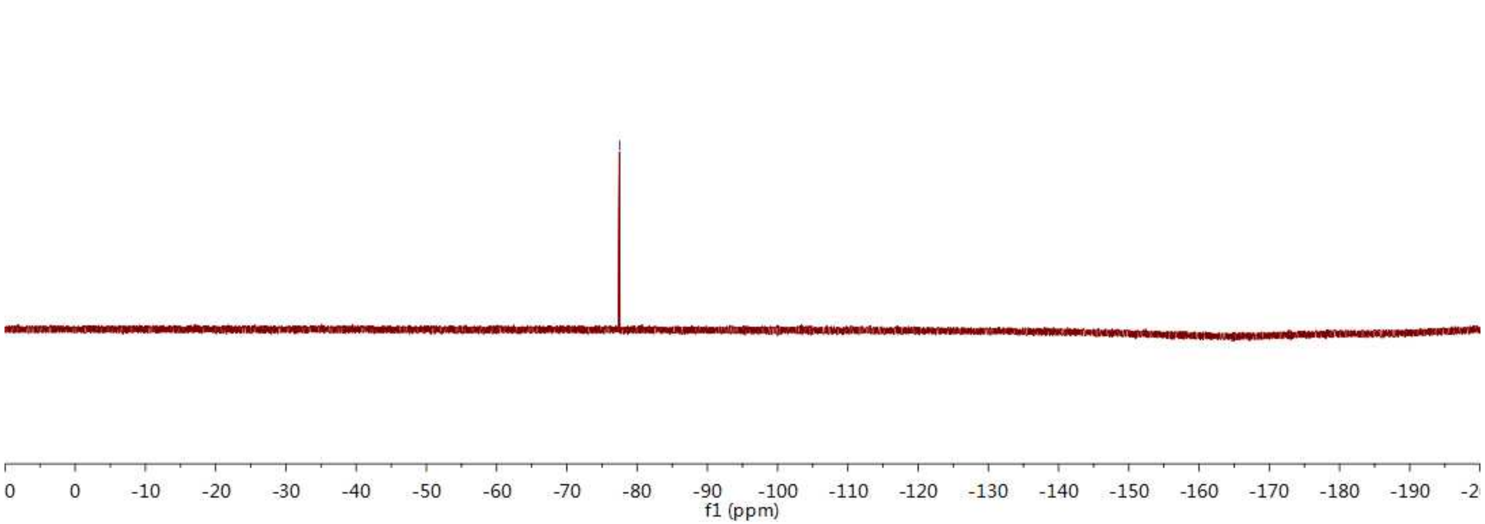

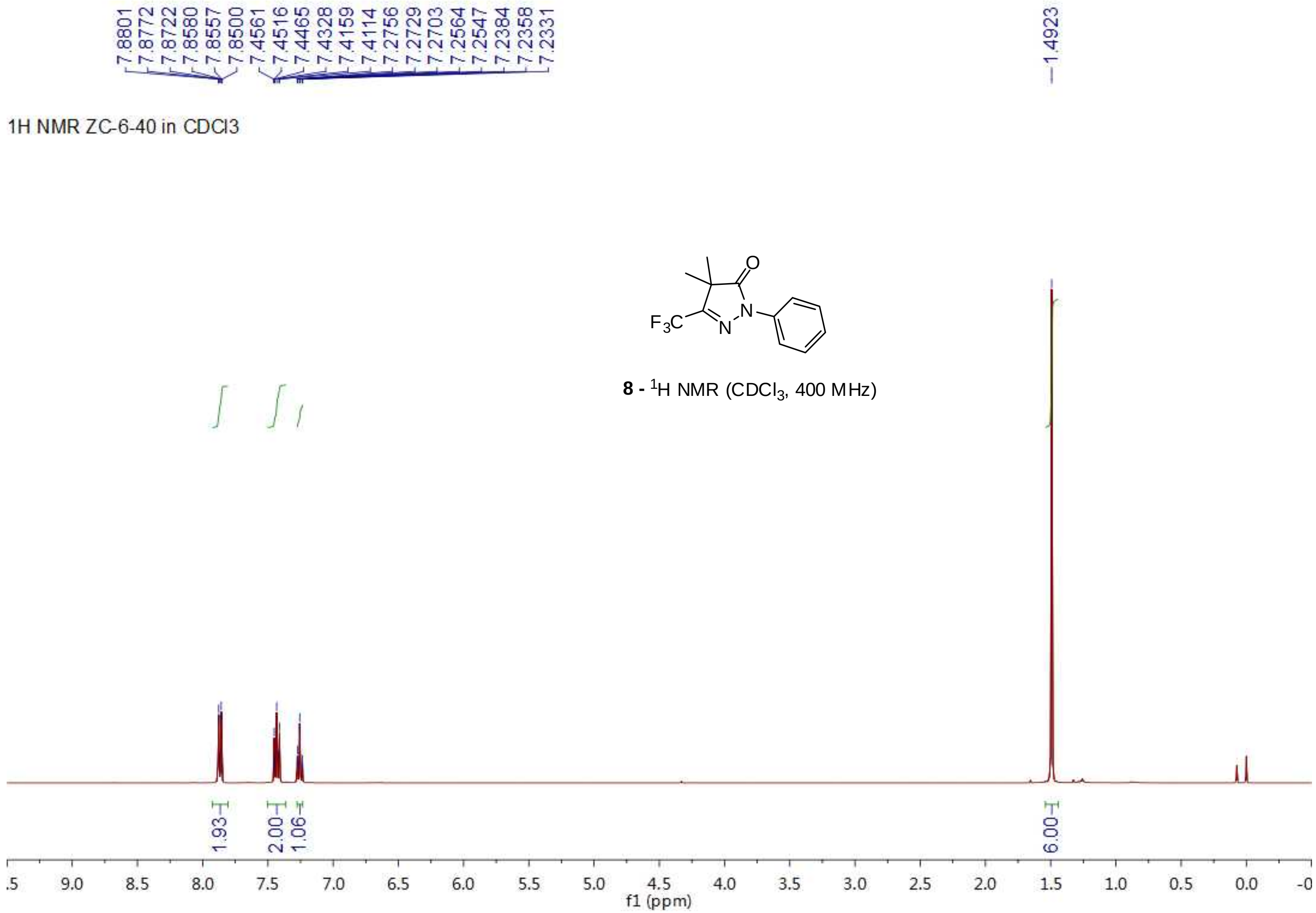
7.8801
7.8772
7.8722
7.8580
7.8557
7.8500
7.4561
7.4516
7.4465
7.4328
7.4159
7.4114
7.2756
7.2729
7.2703
7.2564
7.2547
7.2384
7.2358
7.2331
1.4923
1H NMR ZC-6-40 in CDCl3
O
N
N
F3C
8 - 1H NMR (CDCl3, 400 MHz)
1.93
2.00
1.06
6.00
.5
9.0
8.5
8.0
7.5
7.0
6.5
6.0
5.5
5.0
4.5
4.0
3.5
3.0
2.5
2.0
1.5
1.0
0.5
0.0
-0
f1 (ppm)

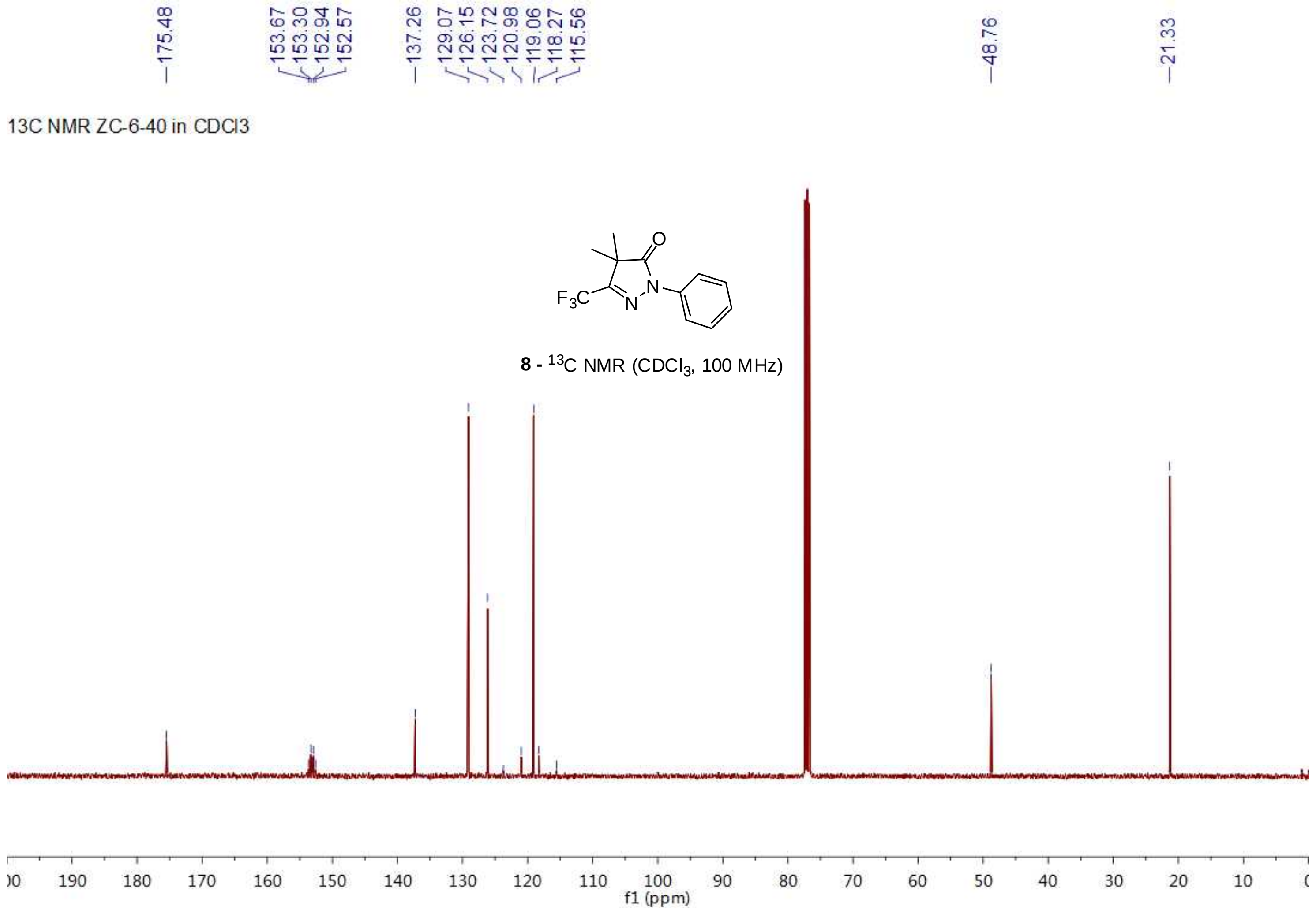
175.48
153.67
153.30
152.94
152.57
137.26
129.07
126.15
123.72
120.98
119.06
118.27
115.56
48.76
21.33
13C NMR ZC-6-40 in CDCl3
O
N
N
F3C
8 - 13C NMR (CDCl3, 100 MHz)
190
180
170
160
150
140
130
120
110
100
90
80
70
60
50
40
30
20
10
f1 (ppm)

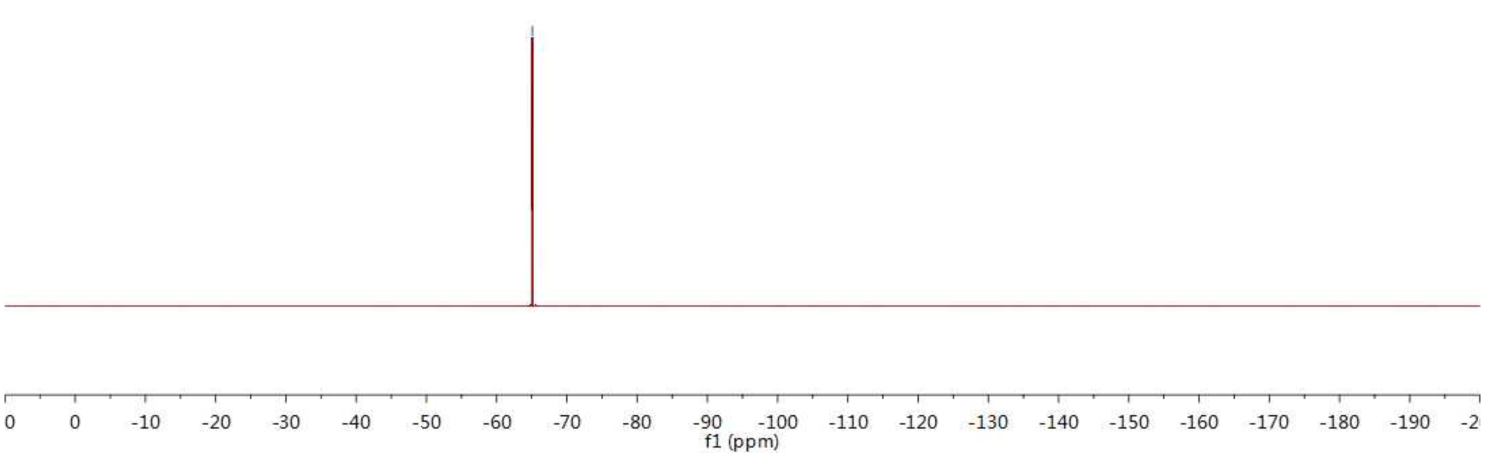

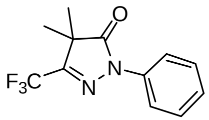

8 - ^{19}F NMR ($CDCl_3$, 377 MHz)

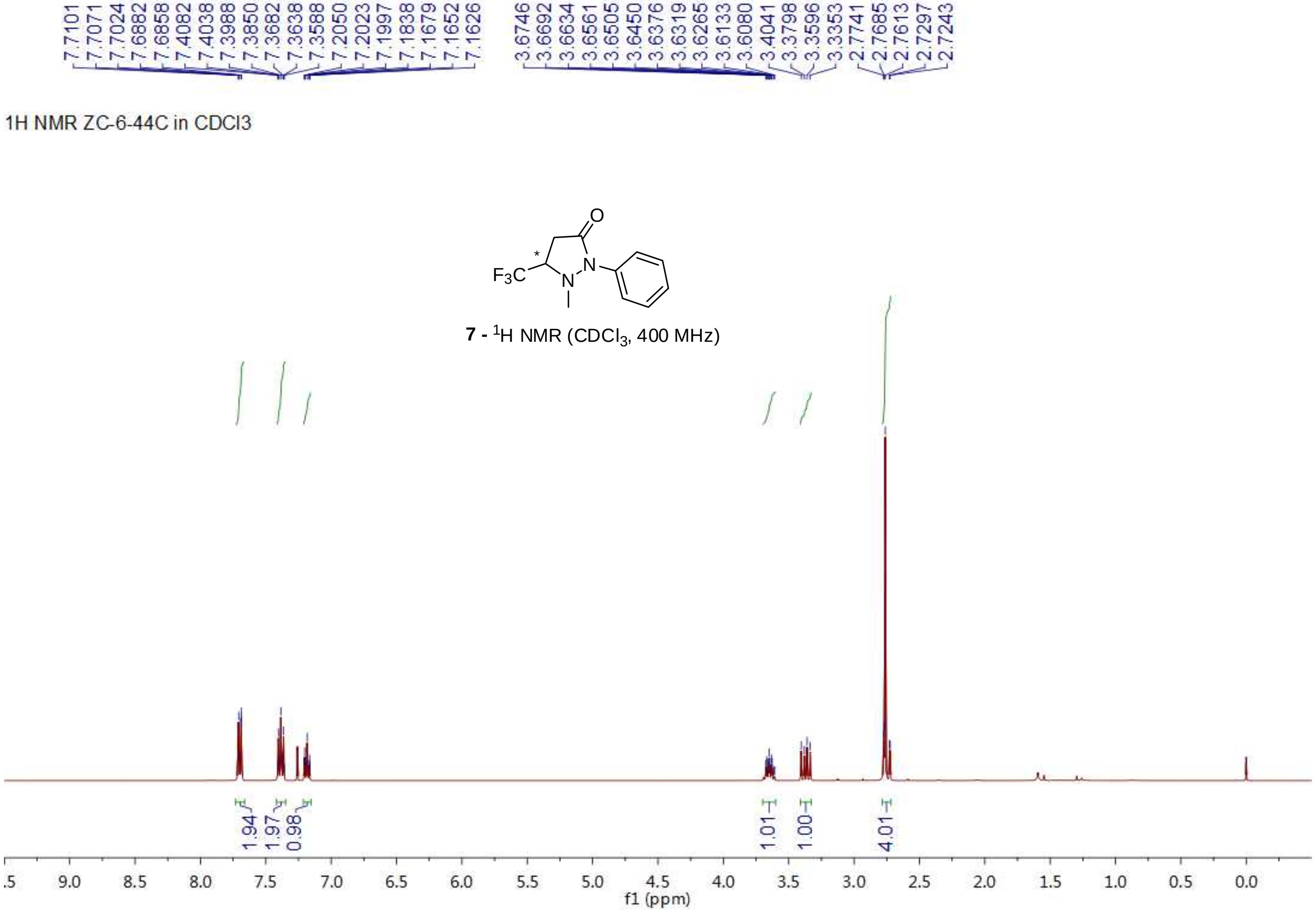

7 - ^{1}H NMR ($CDCl_3$, 400 MHz)

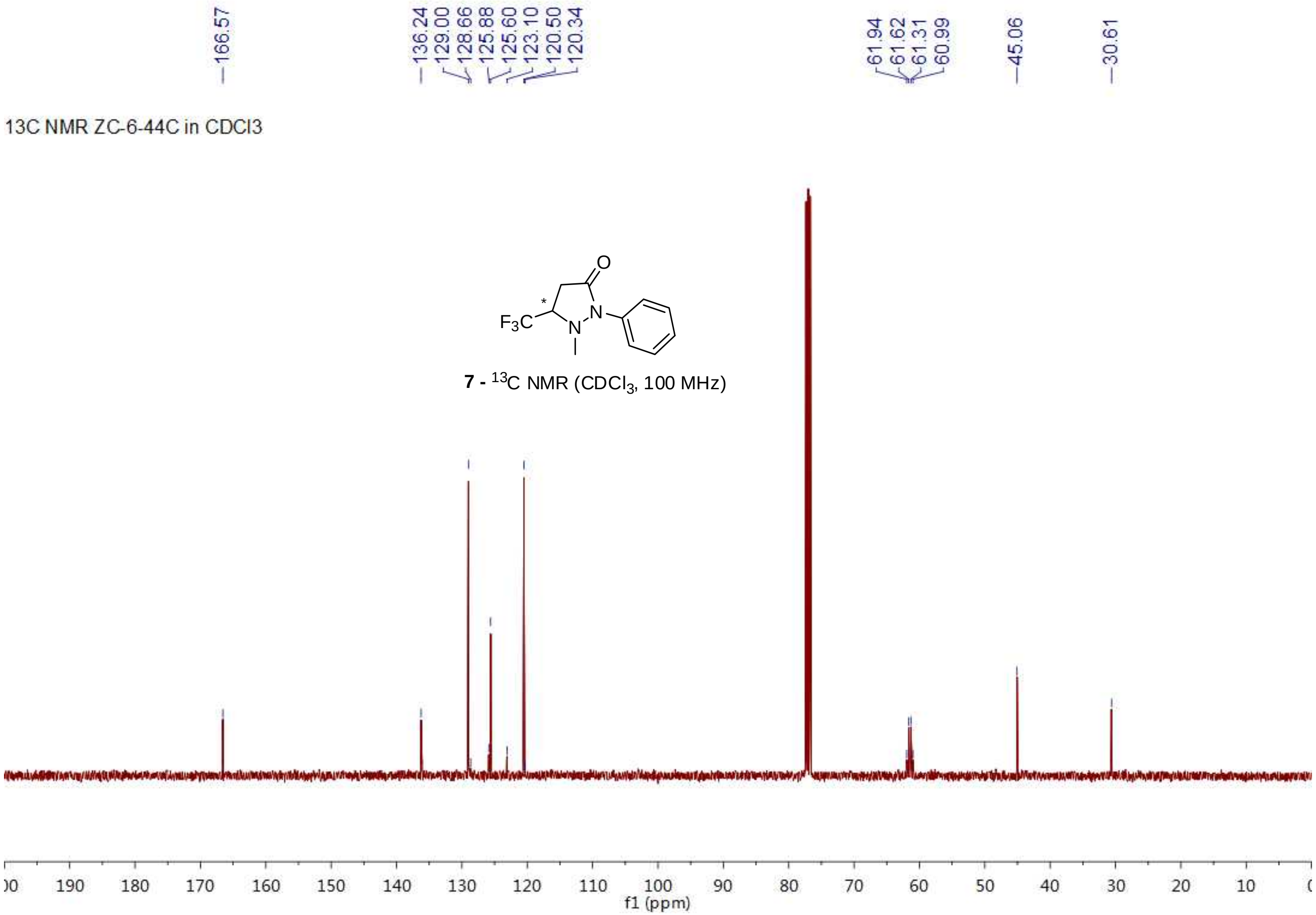

7 - ^{13}C NMR ($CDCl_3$, 100 MHz)

19F NMR ZC-6-44C in CDCl3

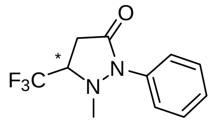

7 - ^{19}F NMR ($CDCl_3$, 377 MHz)

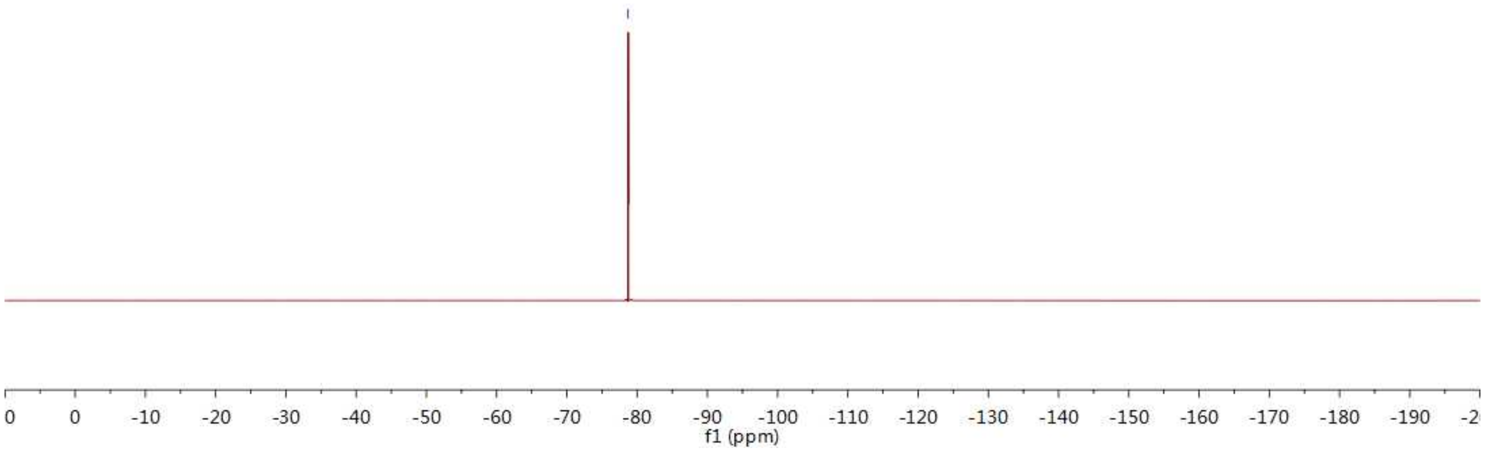

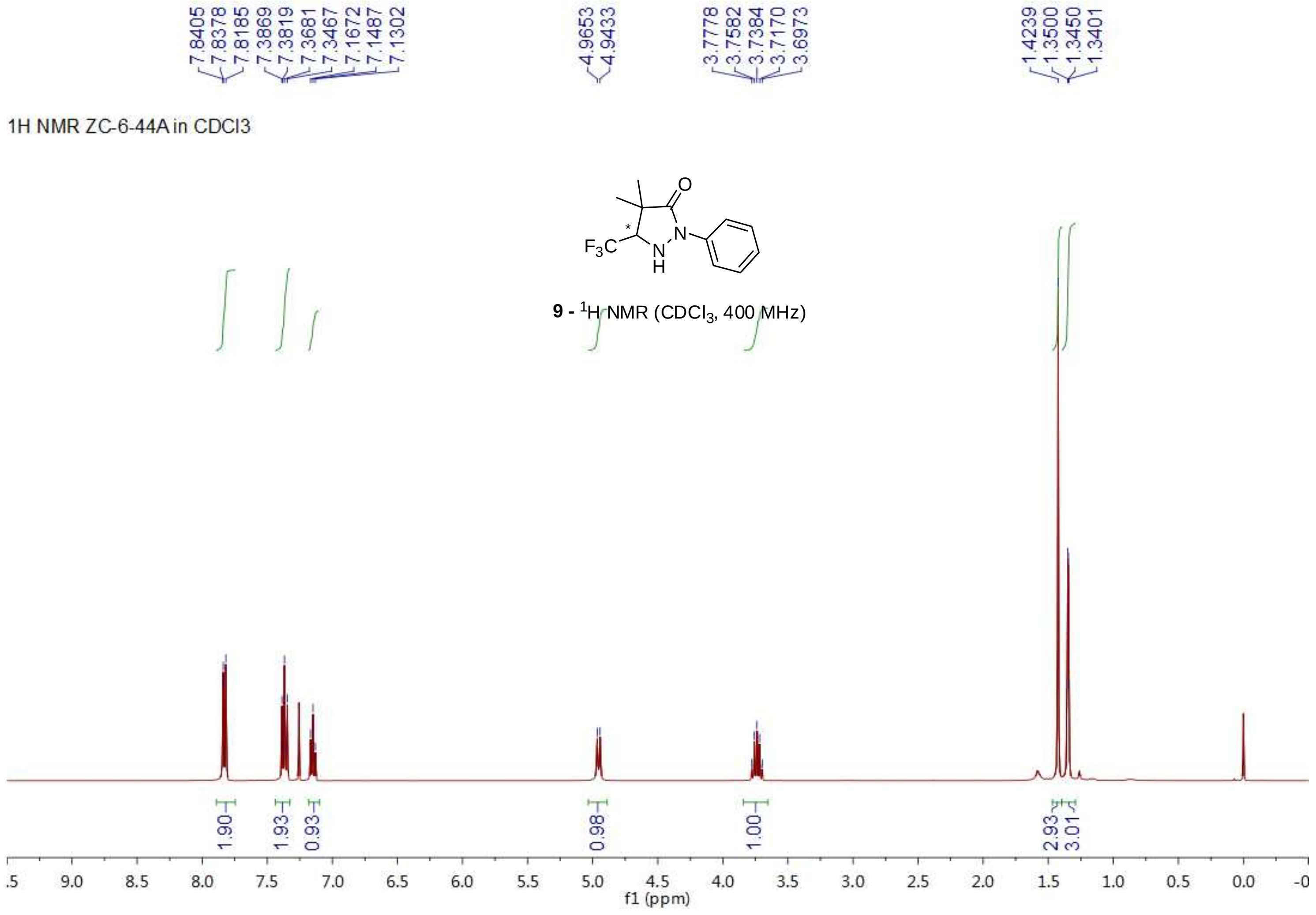

7.8405
7.8378
7.8185
7.3869
7.3819
7.3681
7.3467
7.1672
7.1487
7.1302
4.9653
4.9433
3.7778
3.7582
3.7384
3.7170
3.6973
1.4239
1.3500
1.3450
1.3401
1H NMR ZC-6-44A in CDCl3
O
N
N
H
F3C
*
9 - 1H NMR (CDCl3, 400 MHz)
1.90
1.93
0.93
0.98
1.00
2.93
3.01
.5
9.0
8.5
8.0
7.5
7.0
6.5
6.0
5.5
5.0
4.5
4.0
3.5
3.0
2.5
2.0
1.5
1.0
0.5
0.0
-0
f1 (ppm)

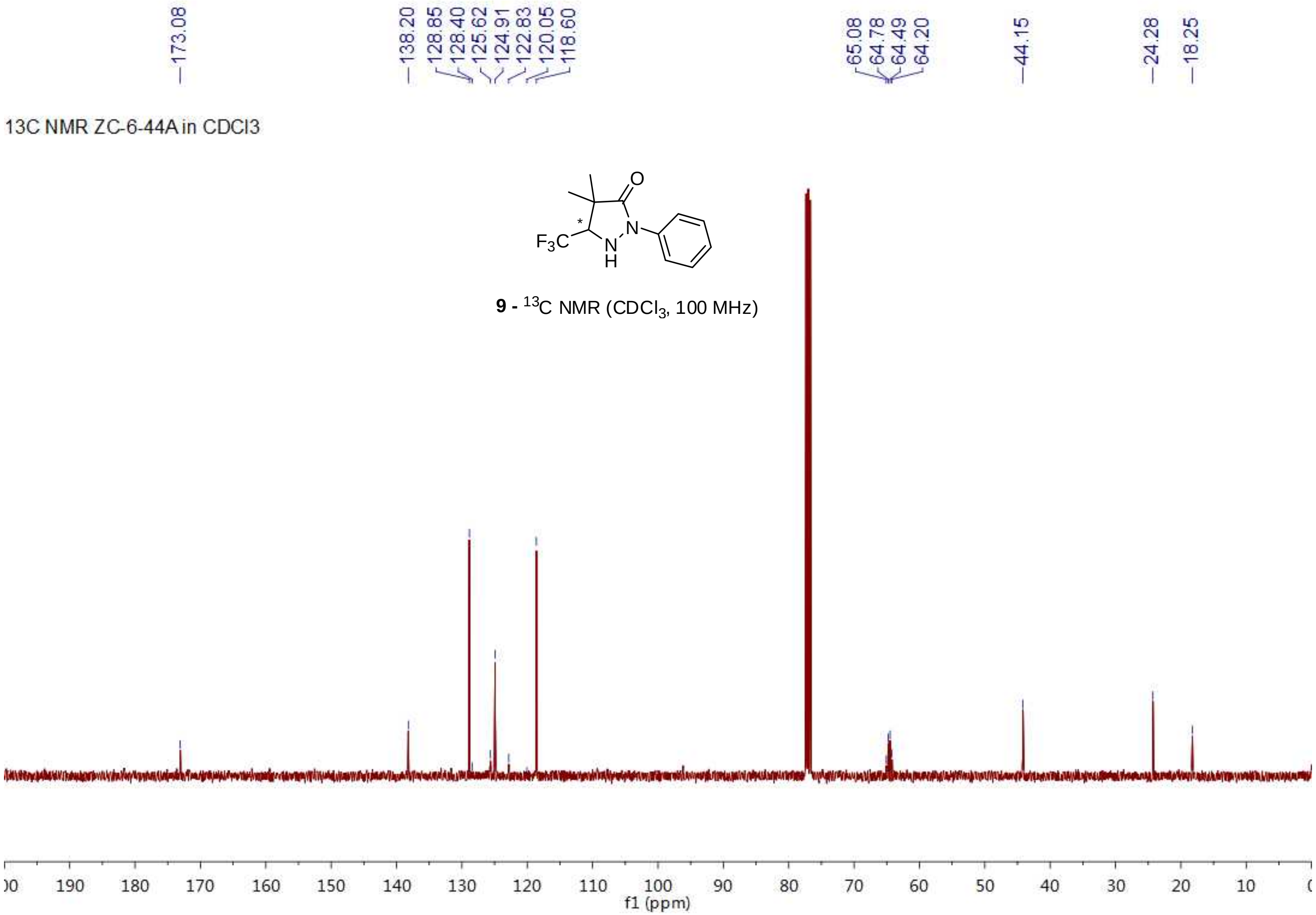

173.08
138.20
128.85
128.40
125.62
124.91
122.83
120.05
118.60
65.08
64.78
64.49
64.20
44.15
24.28
18.25
13C NMR ZC-6-44A in CDCl3
O
F3C
N
N
H
*
9 - 13C NMR (CDCl3, 100 MHz)
190
180
170
160
150
140
130
120
110
100
90
80
70
60
50
40
30
20
10
f1 (ppm)

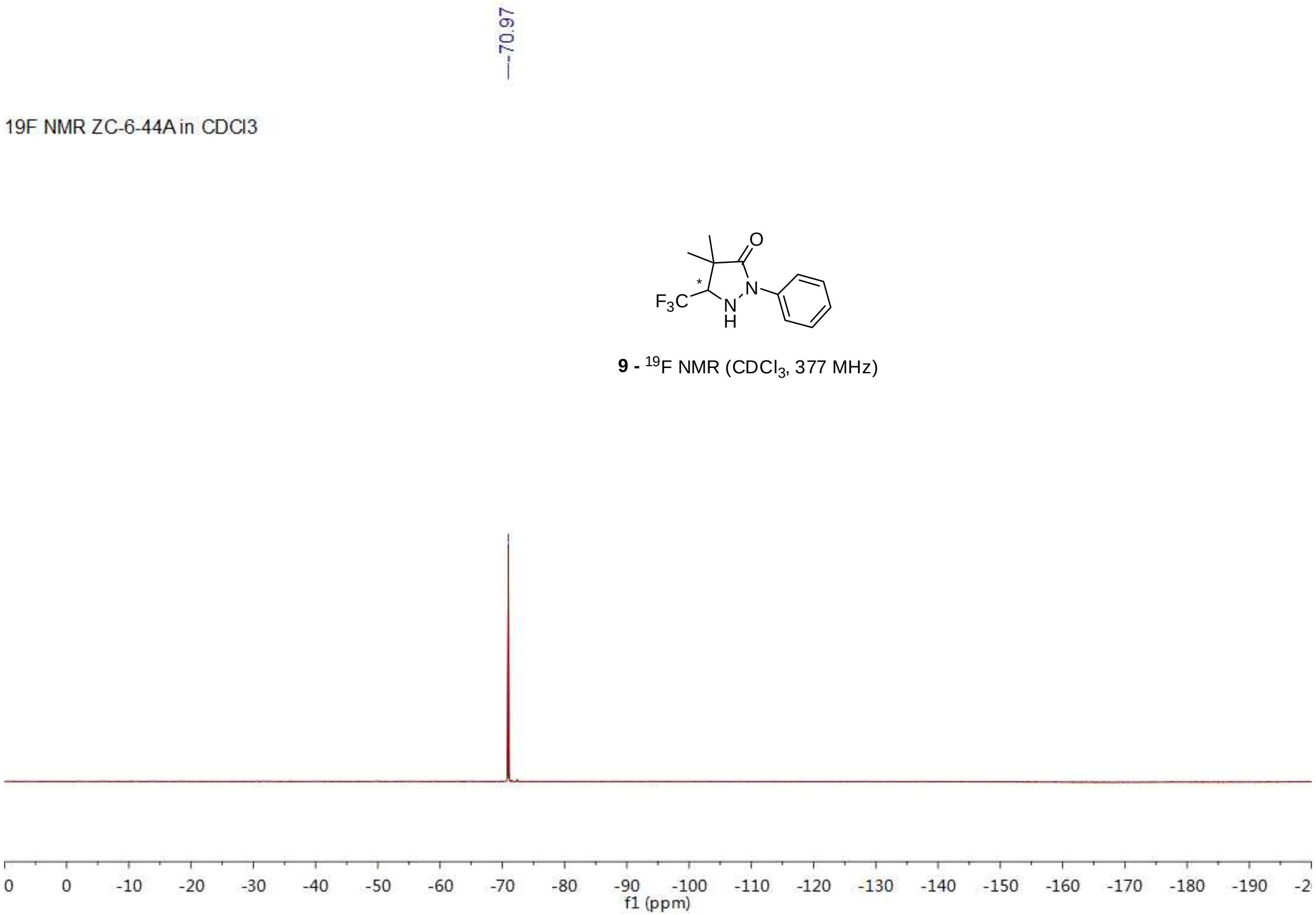

9 - ^{19}F NMR ($CDCl_3$, 377 MHz)

9. Copy of HPLC for Racemic and Chiral Products

Data File C:\CHEM32\1\DATA\ZHOU-14\YZN005068.D
Sample Name: ZC-M-20A+-

===

Acq. Operator : Z
Acq. Instrument : Instrument 1 Location : Vial 1
Injection Date : 5/16/2014 3:37:35 PM
Acq. Method : C:\CHEM32\1\METHODS\DEF LC.M
Last changed : 5/16/2014 2:57:52 PM by Z
(modified after loading)
Analysis Method : C:\CHEM32\1\METHODS\DEF LC.M
Last changed : 5/26/2014 2:25:21 PM by Z
(modified after loading)
Sample Info : AD-H, H/i-PrOH = 70/30, 0.7 mL/min, 30 oC, 254 nm

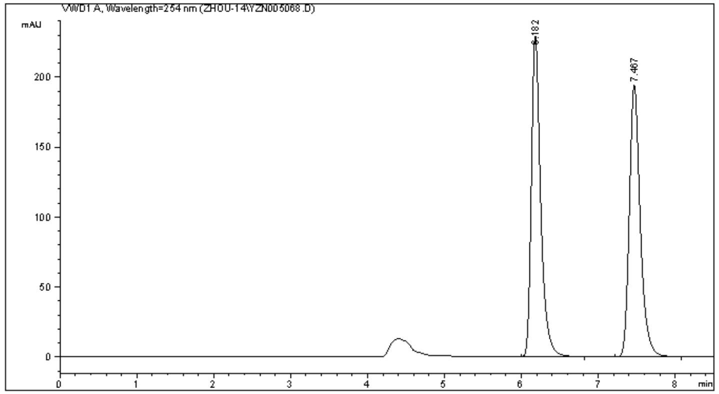

===
Area Percent Report
===

Sorted By : Signal
Multiplier: : 1.0000
Dilution: : 1.0000
Use Multiplier & Dilution Factor with ISTDs

Signal 1: VWD1 A, Wavelength=254 nm

Peak #	RetTime [min]	Type	Width [min]	Area mAU *s	Height [mAU]	Area %
1	6.182	BB	0.1276	1936.70898	229.33224	50.3419
2	7.467	BB	0.1496	1910.39893	194.30939	49.6581
Totals :				3847.10791	423.64163	

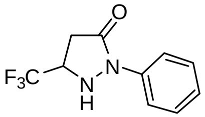

(+/-) - **2a**

===
*** End of Report ***

Instrument 1 5/26/2014 2:26:13 PM Z Page 1 of 1

Data File C:\CHEM32\1\DATA\ZHOU-14\YZN005339.D
Sample Name: ZC-5-59A

===

Acq. Operator : Z
Acq. Instrument : Instrument 1 Location : Vial 1
Injection Date : 6/20/2014 9:37:41 AM
Acq. Method : C:\CHEM32\1\METHODS\DEF LC.M
Last changed : 6/20/2014 8:58:39 AM by Z
(modified after loading)
Analysis Method : C:\CHEM32\1\METHODS\DEF LC.M
Last changed : 12/8/2014 8:57:12 AM by Z
(modified after loading)
Sample Info : AD-H, H/i-PrOH = 70/30, 0.7 mL/min, 30 oC, 254 nm

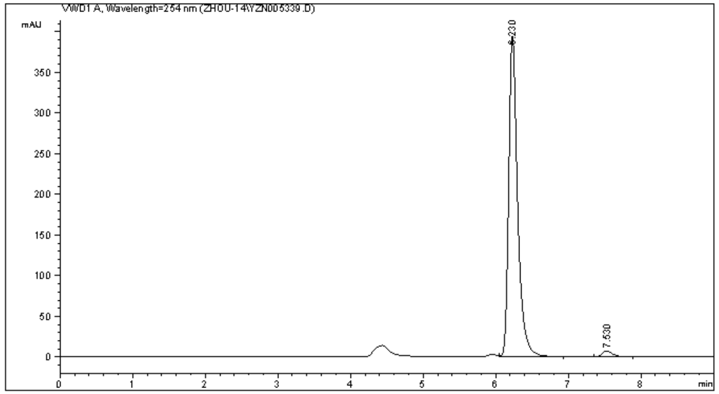

===
Area Percent Report
===

Sorted By : Signal
Multiplier: : 1.0000
Dilution: : 1.0000
Use Multiplier & Dilution Factor with ISTDs

Signal 1: VWD1 A, Wavelength=254 nm

Peak #	RetTime [min]	Type	Width [min]	Area mAU *s	Height [mAU]	Area %
1	6.230	VB	0.1287	3367.25513	394.48074	97.9239
2	7.530	BB	0.1472	71.39044	7.32410	2.0761
Totals :				3438.64557	401.80484	

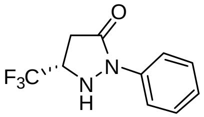

(+) - **2a**

===
*** End of Report ***

Instrument 1 12/8/2014 8:57:16 AM Z Page 1 of 1

Data File C:\CHEM32\1\DATA\ZHOU-14\YZN005667.D
Sample Name: ZC-5-83A+-

===

Acq. Operator : Z
Acq. Instrument : Instrument 1 Location : Vial 1
Injection Date : 7/24/2014 2:36:06 PM
Acq. Method : C:\CHEM32\1\METHODS\DEF LC.M
Last changed : 7/24/2014 2:34:41 PM by Z
(modified after loading)
Analysis Method : C:\CHEM32\1\METHODS\DEF LC.M
Last changed : 12/8/2014 9:00:48 AM by Z
(modified after loading)
Sample Info : AD-H , H/i-PrOH = 80/20, 0.70 mL/min, 30 oC, 254 nm

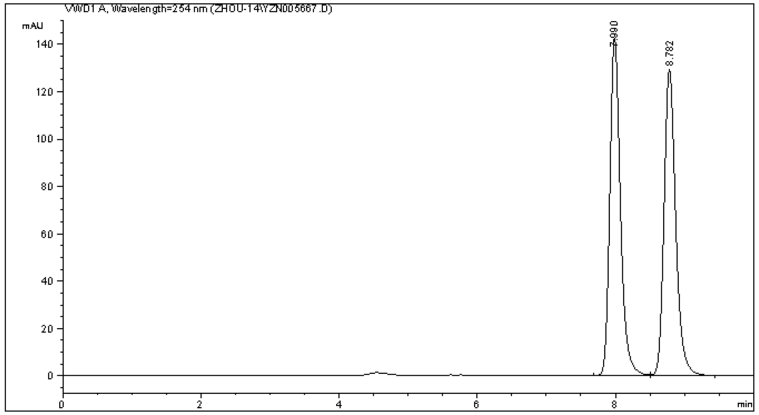

===
Area Percent Report
===

Sorted By : Signal
Multiplier: : 1.0000
Dilution: : 1.0000
Use Multiplier & Dilution Factor with ISTDs

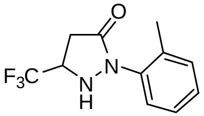

(+/-) - **2b**

Signal 1: VWD1 A, Wavelength=254 nm

Peak #	RetTime [min]	Type	Width [min]	Area mAU *s	Height [mAU]	Area %
1	7.990	BV	0.1639	1524.40454	142.75763	50.1431
2	8.782	VB	0.1789	1515.70349	129.41541	49.8569

Totals : 3040.10803 272.17303

===
*** End of Report ***

Instrument 1 12/8/2014 9:01:27 AM Z Page 1 of 1

Data File C:\CHEM32\1\DATA\ZHOU-14\YZN005666.D
Sample Name: ZC-5-83A

===

Acq. Operator : Z
Acq. Instrument : Instrument 1 Location : Vial 1
Injection Date : 7/24/2014 2:20:43 PM
Acq. Method : C:\CHEM32\1\METHODS\DEF LC.M
Last changed : 7/24/2014 2:18:12 PM by Z
(modified after loading)
Analysis Method : C:\CHEM32\1\METHODS\DEF LC.M
Last changed : 12/8/2014 9:00:48 AM by Z
(modified after loading)
Sample Info : AD-H , H/i-PrOH = 80/20, 0.70 mL/min, 30 oC, 254 nm

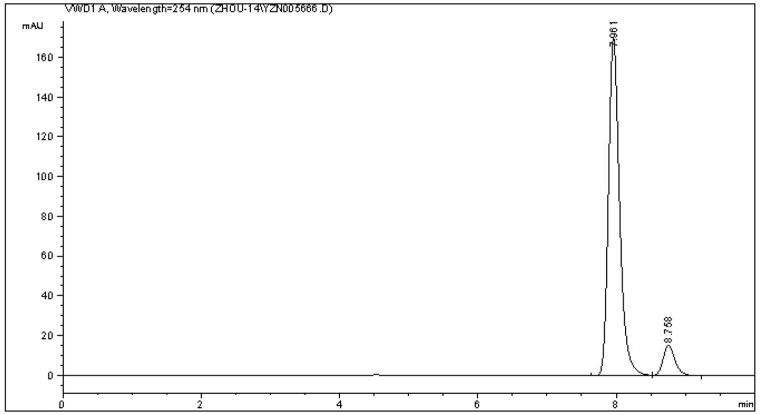

===
Area Percent Report
===

Sorted By : Signal
Multiplier: : 1.0000
Dilution: : 1.0000
Use Multiplier & Dilution Factor with ISTDs

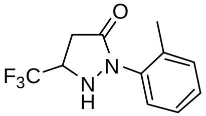

(+) - **2b**

Signal 1: VWD1 A, Wavelength=254 nm

Peak #	RetTime [min]	Type	Width [min]	Area mAU *s	Height [mAU]	Area %
1	7.961	BV	0.1701	1908.54773	170.18611	91.0756
2	8.758	VB	0.1866	187.01726	15.27134	8.9244

Totals : 2095.56499 185.45745

===
*** End of Report ***

Instrument 1 12/8/2014 9:00:53 AM Z Page 1 of 1

Data File C:\CHEM32\1\DATA\ZHOU-14\YZN005613.D
Sample Name: ZC-5-81B+-

===

Acq. Operator : Z
Acq. Instrument : Instrument 1   Location : Vial 1
Injection Date : 7/21/2014 4:25:12 PM
Acq. Method : C:\CHEM32\1\METHODS\DEF LC.M
Last changed : 7/21/2014 4:24:42 PM by Z
(modified after loading)
Analysis Method : C:\CHEM32\1\METHODS\DEF LC.M
Last changed : 12/8/2014 9:00:48 AM by Z
(modified after loading)
Sample Info : AD-H , H/i-PrOH = 70/30, 0.7 mL/min, 30 oC, 254 nm

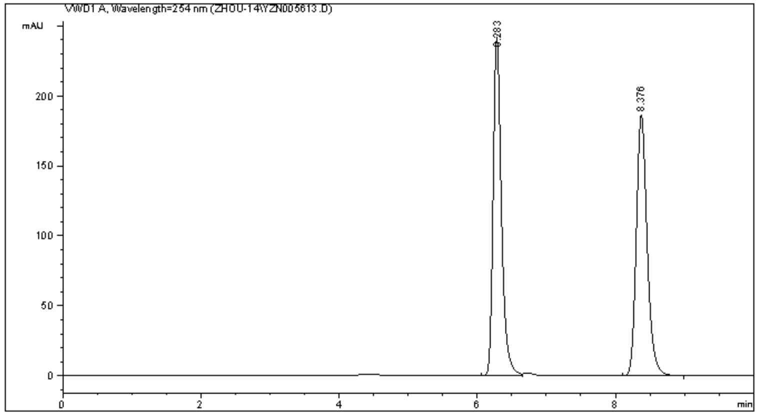

===

Area Percent Report

===

Sorted By : Signal
Multiplier: : 1.0000
Dilution: : 1.0000
Use Multiplier & Dilution Factor with ISTDs

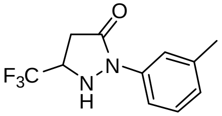

(+/-) - **2c**

Signal 1: VWD1 A, Wavelength=254 nm

Peak #	RetTime [min]	Type	Width [min]	Area mAU *s	Height [mAU]	Area %
1	6.283	VV	0.1266	2018.98083	241.69243	49.8280
2	8.376	BB	0.1665	2032.91565	186.51620	50.1720

Totals : 4051.89648 428.20863

===

*** End of Report ***

Instrument 1 12/8/2014 9:02:47 AM Z   Page 1 of 1

Data File C:\CHEM32\1\DATA\ZHOU-14\YZN005601.D
Sample Name: ZC-5-81B

===

Acq. Operator : Z
Acq. Instrument : Instrument 1   Location : Vial 1
Injection Date : 7/21/2014 11:03:23 AM
Acq. Method : C:\CHEM32\1\METHODS\DEF LC.M
Last changed : 7/21/2014 11:01:34 AM by Z
(modified after loading)
Analysis Method : C:\CHEM32\1\METHODS\DEF LC.M
Last changed : 12/8/2014 9:00:48 AM by Z
(modified after loading)
Sample Info : AD-H , H/i-PrOH = 70/30, 0.7 mL/min, 30 oC, 254 nm

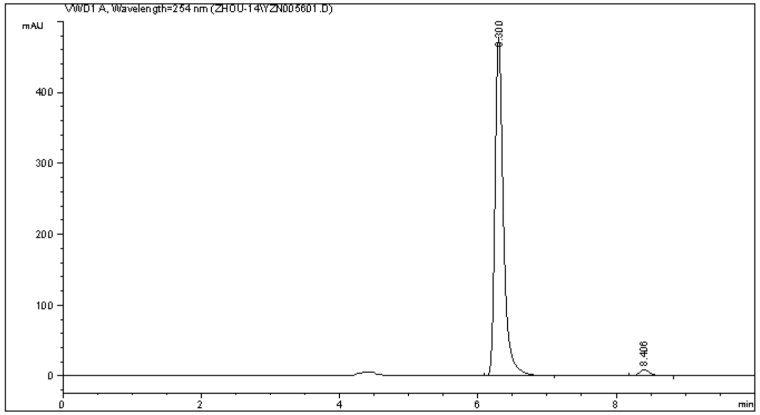

===

Area Percent Report

===

Sorted By : Signal
Multiplier: : 1.0000
Dilution: : 1.0000
Use Multiplier & Dilution Factor with ISTDs

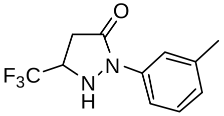

(+) - **2c**

Signal 1: VWD1 A, Wavelength=254 nm

Peak #	RetTime [min]	Type	Width [min]	Area mAU *s	Height [mAU]	Area %
1	6.300	BB	0.1267	4052.55566	477.21707	97.6812
2	8.406	BB	0.1666	96.20064	8.81815	2.3188

Totals : 4148.75630 486.03522

===

*** End of Report ***

Instrument 1 12/8/2014 9:02:14 AM Z   Page 1 of 1

Data File C:\CHEM32\1\DATA\ZHOU-14\YZN005615.D
Sample Name: ZC-5-81C+-

===

Acq. Operator : Z
Acq. Instrument : Instrument 1 Location : Vial 1
Injection Date : 7/21/2014 4:51:12 PM
Acq. Method : C:\CHEM32\1\METHODS\DEF LC.M
Last changed : 7/21/2014 4:50:42 PM by Z
(modified after loading)
Analysis Method : C:\CHEM32\1\METHODS\DEF LC.M
Last changed : 12/8/2014 9:00:48 AM by Z
(modified after loading)
Sample Info : AD-H , H/i-PrOH = 70/30, 0.7 mL/min, 30 oC, 254 nm

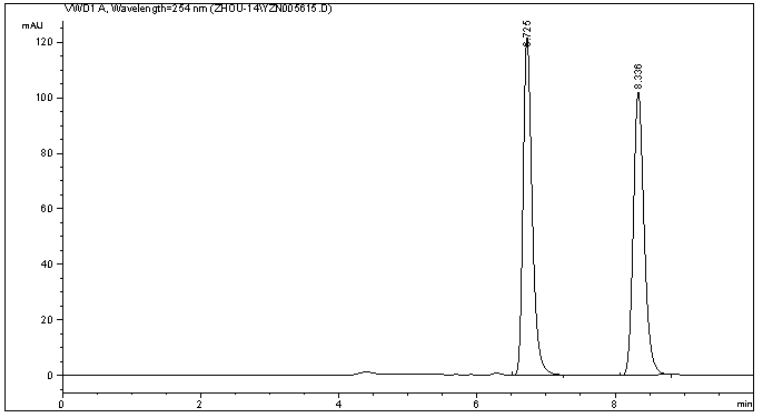

===
Area Percent Report
===

Sorted By : Signal
Multiplier: : 1.0000
Dilution: : 1.0000
Use Multiplier & Dilution Factor with ISTDs

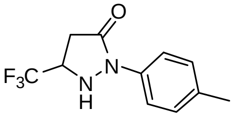

(+/-) - **2d**

Signal 1: VWD1 A, Wavelength=254 nm

Peak #	RetTime [min]	Type	Width [min]	Area mAU *s	Height [mAU]	Area %
1	6.725	VB	0.1389	1113.86377	121.64944	50.1462
2	8.336	BB	0.1676	1107.36755	101.84657	49.8538
Totals :				2221.23132	223.49602	

===
*** End of Report ***

Instrument 1 12/8/2014 9:03:37 AM Z Page 1 of 1

Data File C:\CHEM32\1\DATA\ZHOU-14\YZN005607.D
Sample Name: ZC-5-81C

===

Acq. Operator : Z
Acq. Instrument : Instrument 1 Location : Vial 1
Injection Date : 7/21/2014 2:46:54 PM
Acq. Method : C:\CHEM32\1\METHODS\DEF LC.M
Last changed : 7/21/2014 2:46:29 PM by Z
(modified after loading)
Analysis Method : C:\CHEM32\1\METHODS\DEF LC.M
Last changed : 12/8/2014 9:00:48 AM by Z
(modified after loading)
Sample Info : AD-H , H/i-PrOH = 70/30, 0.7 mL/min, 30 oC, 254 nm

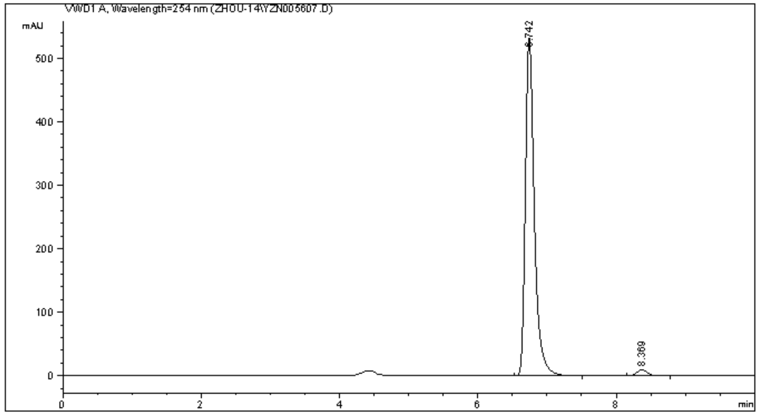

===
Area Percent Report
===

Sorted By : Signal
Multiplier: : 1.0000
Dilution: : 1.0000
Use Multiplier & Dilution Factor with ISTDs

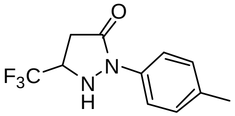

(+) - **2d**

Signal 1: VWD1 A, Wavelength=254 nm

Peak #	RetTime [min]	Type	Width [min]	Area mAU *s	Height [mAU]	Area %
1	6.742	VB	0.1359	4811.26807	532.89709	97.8408
2	8.369	BB	0.1648	106.17783	9.87357	2.1592
Totals :				4917.44589	542.77066	

===
*** End of Report ***

Instrument 1 12/8/2014 9:04:05 AM Z Page 1 of 1

```
Data File C:\CHEM32\1\DATA\ZHOU-14\YZN005610.D
Sample Name: ZC-5-81E+-

=====================================================================
Acq. Operator   : Z
Acq. Instrument : Instrument 1                    Location : Vial 1
Injection Date  : 7/21/2014 3:43:30 PM
Acq. Method     : C:\CHEM32\1\METHODS\DEF LC.M
Last changed    : 7/21/2014 3:27:19 PM by Z
                  (modified after loading)
Analysis Method : C:\CHEM32\1\METHODS\DEF LC.M
Last changed    : 12/8/2014 9:00:48 AM by Z
                  (modified after loading)
Sample Info     : AD-H , H/i-PrOH = 70/30, 0.7 mL/min, 30 oC, 254 nm
```

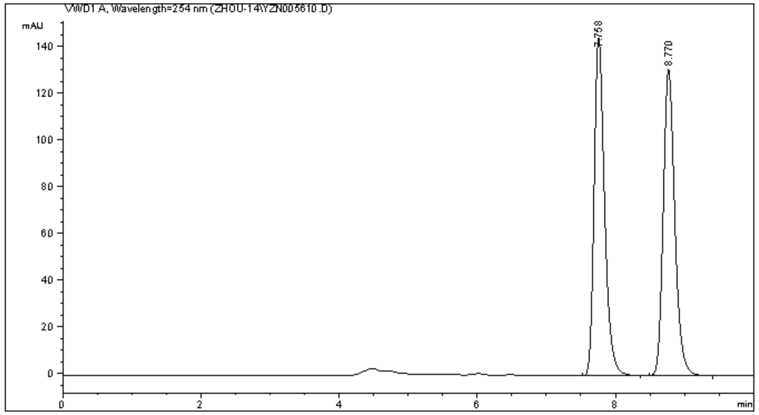


```
=====================================================================
                         Area Percent Report
=====================================================================

Sorted By             :      Signal
Multiplier:                  :      1.0000
Dilution:                    :      1.0000
Use Multiplier & Dilution Factor with ISTDs

Signal 1: VWD1 A, Wavelength=254 nm
```

Peak #	RetTime [min]	Type	Width [min]	Area mAU *s	Height [mAU]	Area %
1	7.758	BB	0.1603	1518.44153	144.70284	49.9998
2	8.770	BB	0.1773	1518.45313	131.11829	50.0002
Totals :				3036.89465	275.82112	

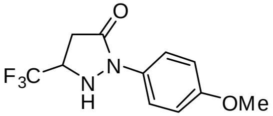

(+/-) - **2e**

```
=====================================================================
                        *** End of Report ***
```



```
Data File C:\CHEM32\1\DATA\ZHOU-14\YZN005611.D
Sample Name: ZC-5-81E

=====================================================================
Acq. Operator   : Z
Acq. Instrument : Instrument 1                    Location : Vial 1
Injection Date  : 7/21/2014 3:56:37 PM
Acq. Method     : C:\CHEM32\1\METHODS\DEF LC.M
Last changed    : 7/21/2014 3:56:12 PM by Z
                  (modified after loading)
Analysis Method : C:\CHEM32\1\METHODS\DEF LC.M
Last changed    : 12/8/2014 9:00:48 AM by Z
                  (modified after loading)
Sample Info     : AD-H , H/i-PrOH = 70/30, 0.7 mL/min, 30 oC, 254 nm
```

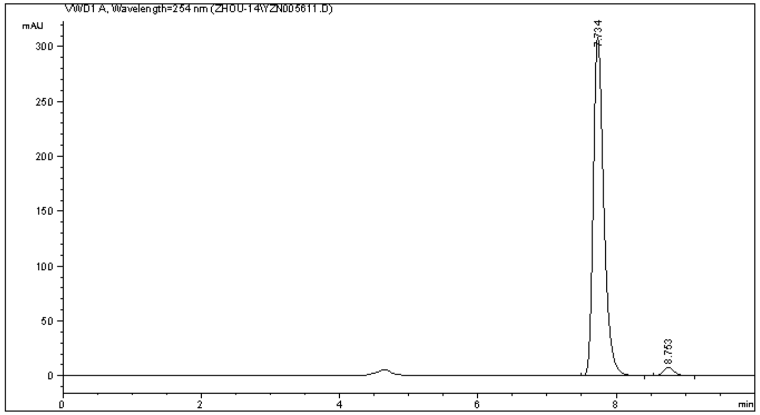


```
=====================================================================
                         Area Percent Report
=====================================================================

Sorted By             :      Signal
Multiplier:                  :      1.0000
Dilution:                    :      1.0000
Use Multiplier & Dilution Factor with ISTDs

Signal 1: VWD1 A, Wavelength=254 nm
```

Peak #	RetTime [min]	Type	Width [min]	Area mAU *s	Height [mAU]	Area %
1	7.734	BB	0.1609	3253.38062	308.39325	97.3675
2	8.753	BB	0.1762	87.96004	7.66204	2.6325
Totals :				3341.34066	316.05529	

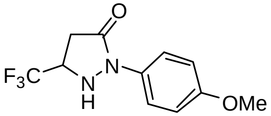

(+) - **2e**

```
=====================================================================
                        *** End of Report ***
```

Data File C:\CHEM32\1\DATA\ZHOU-14\YZN005608.D
Sample Name: ZC-5-81D+-

===

Acq. Operator : Z
Acq. Instrument : Instrument 1 Location : Vial 1
Injection Date : 7/21/2014 2:59:21 PM
Acq. Method : C:\CHEM32\1\METHODS\DEF LC.M
Last changed : 7/21/2014 2:57:35 PM by Z
(modified after loading)
Analysis Method : C:\CHEM32\1\METHODS\DEF LC.M
Last changed : 12/8/2014 9:00:48 AM by Z
(modified after loading)
Sample Info : AD-H , H/i-PrOH = 70/30, 0.7 mL/min, 30 oC, 254 nm

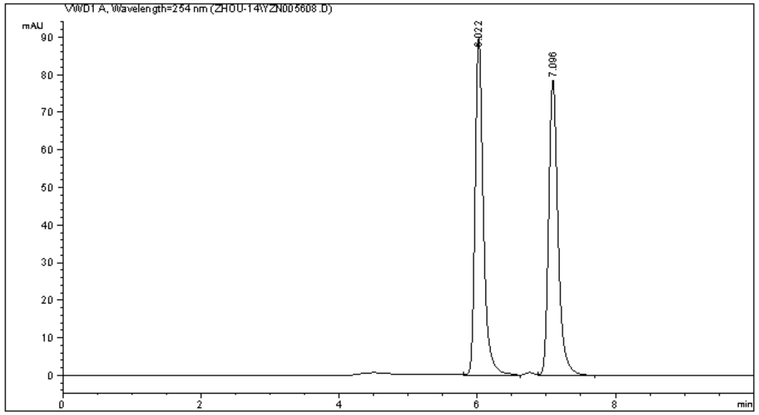

===

Area Percent Report

===

Sorted By : Signal
Multiplier: : 1.0000
Dilution: : 1.0000
Use Multiplier & Dilution Factor with ISTDs

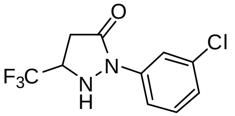

(+/-) - **2f**

Signal 1: VWD1 A, Wavelength=254 nm

Peak #	RetTime [min]	Type	Width [min]	Area mAU *s	Height [mAU]	Area %
1	6.022	VB	0.1267	753.17029	90.01208	50.3189
2	7.096	VB	0.1438	743.62482	78.66964	49.6811

Totals : 1496.79510 168.68172

===

*** End of Report ***

Instrument 1 12/8/2014 9:06:46 AM Z Page 1 of 1

Data File C:\CHEM32\1\DATA\ZHOU-14\YZN005609.D
Sample Name: ZC-5-81D

===

Acq. Operator : Z
Acq. Instrument : Instrument 1 Location : Vial 1
Injection Date : 7/21/2014 3:15:15 PM
Acq. Method : C:\CHEM32\1\METHODS\DEF LC.M
Last changed : 7/21/2014 3:13:39 PM by Z
(modified after loading)
Analysis Method : C:\CHEM32\1\METHODS\DEF LC.M
Last changed : 12/8/2014 9:00:48 AM by Z
(modified after loading)
Sample Info : AD-H , H/i-PrOH = 70/30, 0.7 mL/min, 30 oC, 254 nm

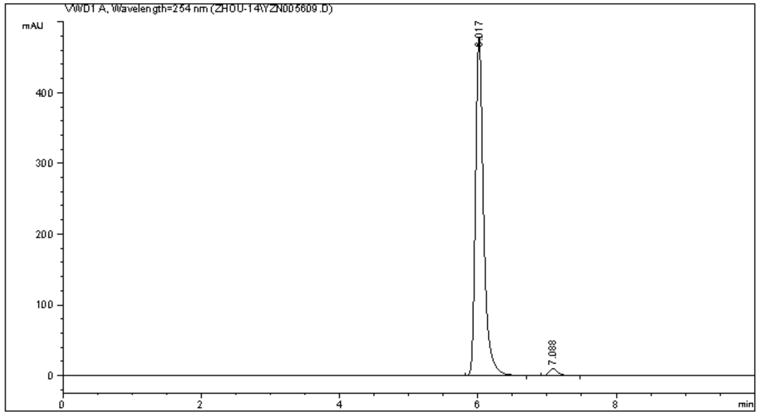

===

Area Percent Report

===

Sorted By : Signal
Multiplier: : 1.0000
Dilution: : 1.0000
Use Multiplier & Dilution Factor with ISTDs

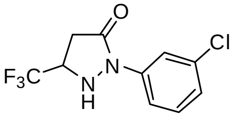

(+) - **2f**

Signal 1: VWD1 A, Wavelength=254 nm

Peak #	RetTime [min]	Type	Width [min]	Area mAU *s	Height [mAU]	Area %
1	6.017	BB	0.1230	3910.24976	478.38824	97.6016
2	7.088	BB	0.1410	96.08696	10.42237	2.3984

Totals : 4006.33672 488.81061

===

*** End of Report ***

Instrument 1 12/8/2014 9:06:16 AM Z Page 1 of 1

Data File C:\CHEM32\1\DATA\ZHOU-14\YZN005675.D
Sample Name: ZC-5-83D+-

===

Acq. Operator : Z
Acq. Instrument : Instrument 1 Location : Vial 1
Injection Date : 7/24/2014 4:27:01 PM
Acq. Method : C:\CHEM32\1\METHODS\DEF LC.M
Last changed : 7/24/2014 4:26:00 PM by Z
(modified after loading)
Analysis Method : C:\CHEM32\1\METHODS\DEF LC.M
Last changed : 12/8/2014 9:08:01 AM by Z
(modified after loading)
Sample Info : AD-H , H/i-PrOH = 85/15, 0.70 mL/min, 30 oC, 254 nm

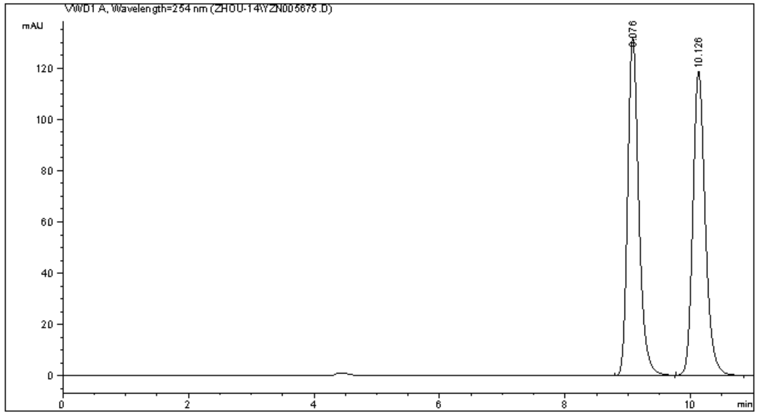

===
Area Percent Report
===

Sorted By : Signal
Multiplier: : 1.0000
Dilution: : 1.0000
Use Multiplier & Dilution Factor with ISTDs

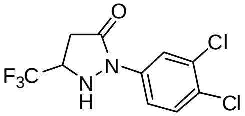

(+/-) - **2g**

Signal 1: VWD1 A, Wavelength=254 nm

Peak #	RetTime [min]	Type	Width [min]	Area mAU *s	Height [mAU]	Area %
1	9.076	BB	0.1892	1627.82117	131.78853	50.0202
2	10.126	BB	0.2096	1626.50952	118.56556	49.9798

Totals : 3254.33069 250.35409

===
*** End of Report ***

Instrument 1 12/8/2014 9:08:27 AM Z Page 1 of 1

Data File C:\CHEM32\1\DATA\ZHOU-14\YZN005674.D
Sample Name: ZC-5-83D

===

Acq. Operator : Z
Acq. Instrument : Instrument 1 Location : Vial 1
Injection Date : 7/24/2014 4:14:39 PM
Acq. Method : C:\CHEM32\1\METHODS\DEF LC.M
Last changed : 7/24/2014 4:13:02 PM by Z
(modified after loading)
Analysis Method : C:\CHEM32\1\METHODS\DEF LC.M
Last changed : 12/8/2014 9:08:01 AM by Z
(modified after loading)
Sample Info : AD-H , H/i-PrOH = 85/15, 0.70 mL/min, 30 oC, 254 nm

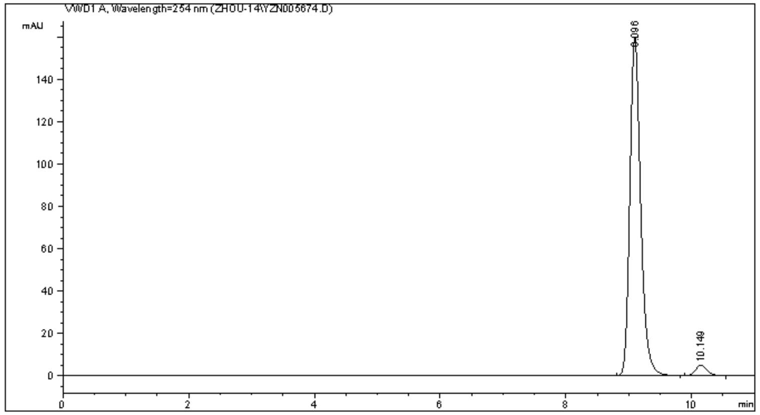

===
Area Percent Report
===

Sorted By : Signal
Multiplier: : 1.0000
Dilution: : 1.0000
Use Multiplier & Dilution Factor with ISTDs

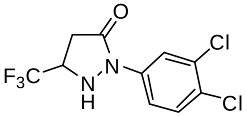

(+) - **2g**

Signal 1: VWD1 A, Wavelength=254 nm

Peak #	RetTime [min]	Type	Width [min]	Area mAU *s	Height [mAU]	Area %
1	9.096	BB	0.1820	1912.09912	159.59459	96.6600
2	10.149	BB	0.2020	66.07129	5.00566	3.3400

Totals : 1978.17041 164.60025

===
*** End of Report ***

Instrument 1 12/8/2014 9:08:04 AM Z Page 1 of 1

Data File G:\HPLC FOR 1\83C\YZ006381.D
Sample Name: ZC-5-83C+-

===

Acq. Operator : ZHOU
Acq. Instrument : Instrument 1 Location : Vial 1
Injection Date : 7/25/2014 7:27:38 AM
Acq. Method : C:\HPCHEM\1\METHODS\DEF LC1.M
Last changed : 7/25/2014 7:22:20 AM by ZHOU
(modified after loading)
Analysis Method : C:\CHEM32\1\METHODS\DEF LC.M
Last changed : 12/8/2014 9:17:59 AM by Z
(modified after loading)
Sample Info : OJ, H/i-PrOH = 70/30, 0.7 mL/min, 30 oC, 254 nm

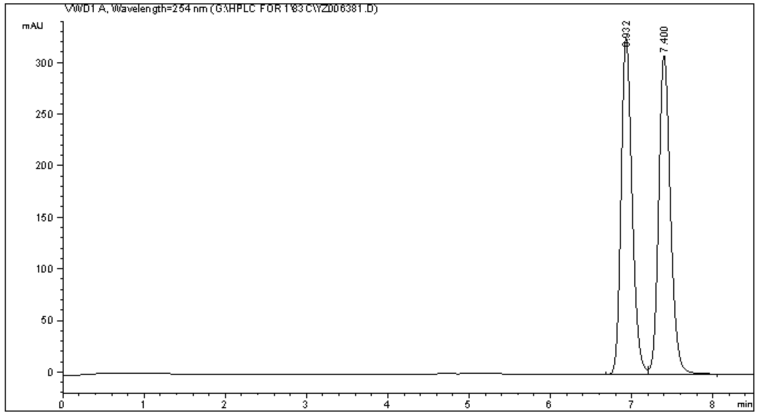

===

Area Percent Report

===

Sorted By : Signal
Multiplier: : 1.0000
Dilution: : 1.0000
Use Multiplier & Dilution Factor with ISTDs

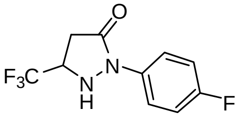

(+/-) - **2h**

Signal 1: VWD1 A, Wavelength=254 nm

Peak #	RetTime [min]	Type	Width [min]	Area mAU *s	Height [mAU]	Area %
1	6.932	VV	0.1443	3059.86548	326.39697	49.7621
2	7.400	VB	0.1530	3089.12012	309.18073	50.2379

Totals : 6148.98560 635.57770

===

*** End of Report ***

Instrument 1 12/8/2014 9:18:15 AM Z Page 1 of 1

Data File G:\HPLC FOR 1\83C\YZ006382.D
Sample Name: ZC-5-83C

===

Acq. Operator : ZHOU
Acq. Instrument : Instrument 1 Location : Vial 1
Injection Date : 7/25/2014 7:37:01 AM
Acq. Method : C:\HPCHEM\1\METHODS\DEF LC1.M
Last changed : 7/25/2014 7:36:48 AM by ZHOU
(modified after loading)
Analysis Method : C:\CHEM32\1\METHODS\DEF LC.M
Last changed : 12/8/2014 9:17:59 AM by Z
(modified after loading)
Sample Info : OJ, H/i-PrOH = 70/30, 0.7 mL/min, 30 oC, 254 nm

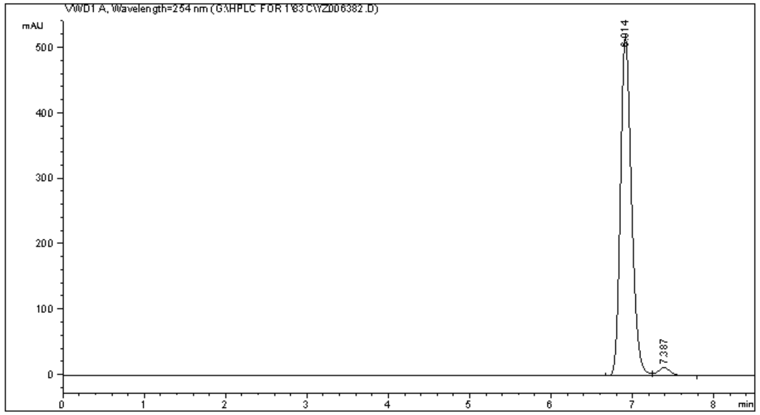

===

Area Percent Report

===

Sorted By : Signal
Multiplier: : 1.0000
Dilution: : 1.0000
Use Multiplier & Dilution Factor with ISTDs

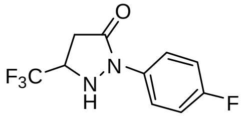

(+) - **2h**

Signal 1: VWD1 A, Wavelength=254 nm

Peak #	RetTime [min]	Type	Width [min]	Area mAU *s	Height [mAU]	Area %
1	6.914	BV	0.1454	4895.64893	517.16840	97.3574
2	7.387	VB	0.1619	132.88655	12.34933	2.6426

Totals : 5028.53548 529.51773

===

*** End of Report ***

Instrument 1 12/8/2014 9:18:04 AM Z Page 1 of 1

Data File C:\CHEM32\1\DATA\ZHOU-14\YZN005956.D
Sample Name: ZC-5-93A+-

===

Acq. Operator : Z
Acq. Instrument : Instrument 1 Location : Vial 1
Injection Date : 9/1/2014 7:51:04 PM
Acq. Method : C:\CHEM32\1\METHODS\DEF LC.M
Last changed : 9/1/2014 7:44:40 PM by Z
(modified after loading)
Analysis Method : C:\CHEM32\1\METHODS\DEF LC.M
Last changed : 12/8/2014 9:10:17 AM by Z
(modified after loading)
Sample Info : AD-H, H/i-PrOH = 70/30, 0.7 mL/min, 30 oC, 254nm

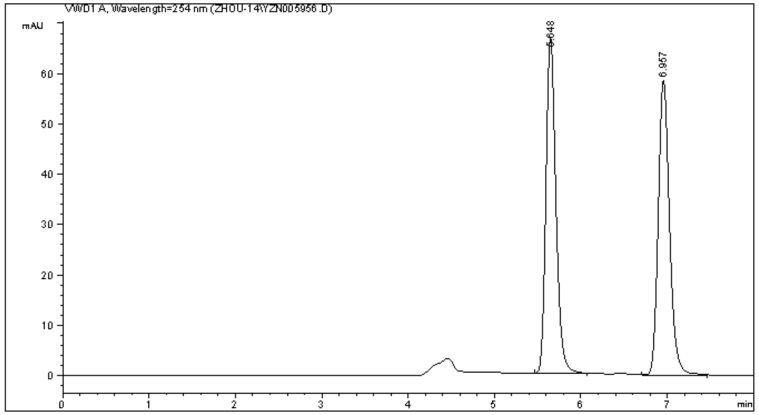

===
Area Percent Report
===

Sorted By : Signal
Multiplier: : 1.0000
Dilution: : 1.0000
Use Multiplier & Dilution Factor with ISTDs

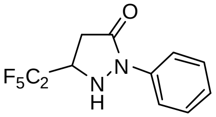

(+/-) - **2i**

Signal 1: VWD1 A, Wavelength=254 nm

Peak #	RetTime [min]	Type	Width [min]	Area mAU *s	Height [mAU]	Area %
1	5.648	BB	0.1229	535.93317	66.69895	50.2407
2	6.957	VB	0.1393	530.79865	58.55206	49.7593
Totals :				1066.73181	125.25101	

===
*** End of Report ***

Instrument 1 12/8/2014 9:10:22 AM Z Page 1 of 1

Data File C:\CHEM32\1\DATA\ZHOU-14\YZN005782.D
Sample Name: ZC-5-88A

===

Acq. Operator : Z
Acq. Instrument : Instrument 1 Location : Vial 1
Injection Date : 7/31/2014 10:07:18 AM
Acq. Method : C:\CHEM32\1\METHODS\DEF LC.M
Last changed : 7/31/2014 10:06:36 AM by Z
(modified after loading)
Analysis Method : C:\CHEM32\1\METHODS\DEF LC.M
Last changed : 12/8/2014 9:10:17 AM by Z
(modified after loading)
Sample Info : AD-H , H/i-PrOH = 70/30, 0.7 mL/min, 30 oC, 254 nm

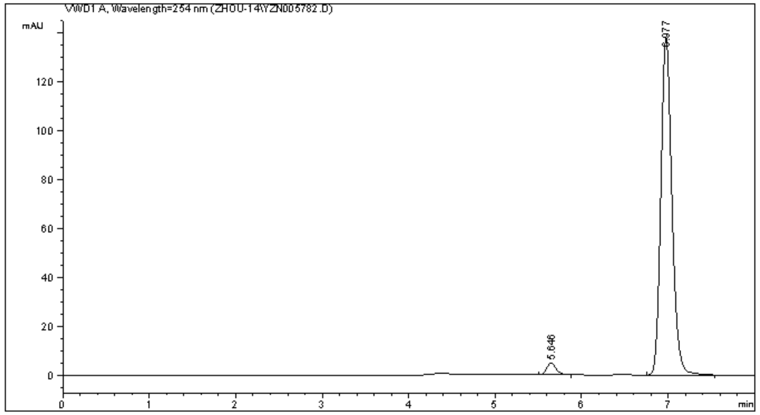

===
Area Percent Report
===

Sorted By : Signal
Multiplier: : 1.0000
Dilution: : 1.0000
Use Multiplier & Dilution Factor with ISTDs

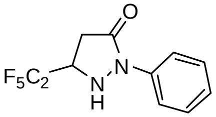

(-) - **2i**

Signal 1: VWD1 A, Wavelength=254 nm

Peak #	RetTime [min]	Type	Width [min]	Area mAU *s	Height [mAU]	Area %
1	5.646	BB	0.1148	36.22553	4.84819	2.8665
2	6.977	BB	0.1358	1227.51172	137.97807	97.1335
Totals :				1263.73725	142.82626	

===
*** End of Report ***

Instrument 1 12/8/2014 9:10:49 AM Z Page 1 of 1

```
Data File C:\CHEM32\1\DATA\ZHOU-14\YZN005910.D
Sample Name: ZC-5-93B+-

    =====================================================================
    Acq. Operator   : Z
    Acq. Instrument : Instrument 1                   Location : Vial 1
    Injection Date  : 8/26/2014 2:48:41 PM
    Acq. Method     : C:\CHEM32\1\METHODS\DEF LC.M
    Last changed    : 8/26/2014 2:47:22 PM by Z
                      (modified after loading)
    Analysis Method : C:\CHEM32\1\METHODS\DEF LC.M
    Last changed    : 12/8/2014 9:12:05 AM by Z
                      (modified after loading)
    Sample Info     : OG-H, H/i-PrOH = 90/10, 0.7 mL/min, 30 oC, 254nm
```

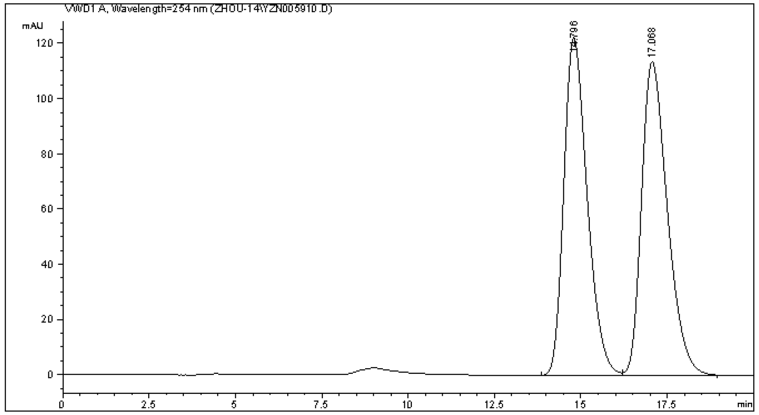


```
    =====================================================================
                             Area Percent Report
    =====================================================================

    Sorted By             :      Signal
    Multiplier:                  :      1.0000
    Dilution:                    :      1.0000
    Use Multiplier & Dilution Factor with ISTDs


    Signal 1: VWD1 A, Wavelength=254 nm

    Peak RetTime Type  Width     Area      Height     Area
      #  [min]         [min]  mAU   *s   [mAU  ]       %
    ----|-------|----|-------|----------|----------|--------|
       1  14.796 BB    0.7202 5749.26123  122.31118  49.3583
       2  17.068 BB    0.7968 5898.76172  113.55722  50.6417

    Totals :                  1.16480e4   235.86840

    =====================================================================
                              *** End of Report ***
```

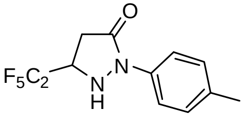

(+/-) - **2j**

```
Instrument 1 12/8/2014 9:12:37 AM Z                                  Page 1 of 1
```

```
Data File C:\CHEM32\1\DATA\ZHOU-14\YZN005911.D
Sample Name: ZC-5-93B

    =====================================================================
    Acq. Operator   : Z
    Acq. Instrument : Instrument 1                   Location : Vial 1
    Injection Date  : 8/26/2014 3:09:53 PM
    Acq. Method     : C:\CHEM32\1\METHODS\DEF LC.M
    Last changed    : 8/26/2014 3:09:15 PM by Z
                      (modified after loading)
    Analysis Method : C:\CHEM32\1\METHODS\DEF LC.M
    Last changed    : 12/8/2014 9:12:05 AM by Z
                      (modified after loading)
    Sample Info     : OG-H, H/i-PrOH = 90/10, 0.7 mL/min, 30 oC, 254nm
```

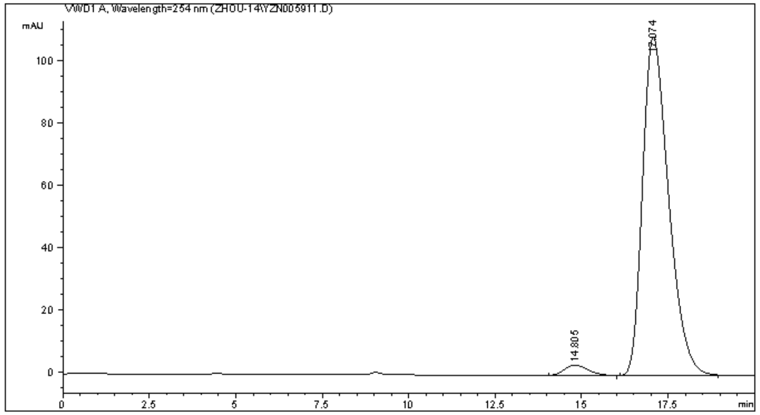


```
    =====================================================================
                             Area Percent Report
    =====================================================================

    Sorted By             :      Signal
    Multiplier:                  :      1.0000
    Dilution:                    :      1.0000
    Use Multiplier & Dilution Factor with ISTDs


    Signal 1: VWD1 A, Wavelength=254 nm

    Peak RetTime Type  Width     Area      Height     Area
      #  [min]         [min]  mAU   *s   [mAU  ]       %
    ----|-------|----|-------|----------|----------|--------|
       1  14.805 BB    0.6674  156.58360    3.36045   2.6821
       2  17.074 BB    0.8038 5681.59717  108.37350  97.3179

    Totals :                  5838.18077  111.73394

    =====================================================================
                              *** End of Report ***
```

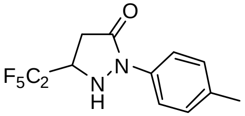

(-) - **2j**

```
Instrument 1 12/8/2014 9:12:10 AM Z                                  Page 1 of 1
```

Data File C:\CHEM32\1\DATA\ZHOU-14\YZN005772.D
Sample Name: ZC-5-86F+-

Acq. Operator : Z
Acq. Instrument : Instrument 1 Location : Vial 1
Injection Date : 7/30/2014 10:01:31 AM
Acq. Method : C:\CHEM32\1\METHODS\DEF LC.M
Last changed : 7/30/2014 9:59:11 AM by Z
(modified after loading)
Analysis Method : C:\CHEM32\1\METHODS\DEF LC.M
Last changed : 12/4/2014 9:39:58 PM by Z
(modified after loading)
Sample Info : AD-H , H/i-PrOH = 70/30 0.7 mL/min, 30 oC, 254 nm

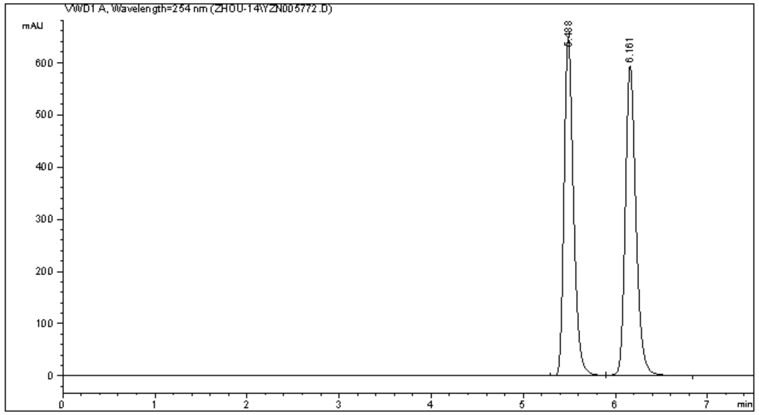

Area Percent Report

Sorted By : Signal
Multiplier: : 1.0000
Dilution: : 1.0000
Use Multiplier & Dilution Factor with ISTDs

Signal 1: VWD1 A, Wavelength=254 nm

Peak #	RetTime [min]	Type	Width [min]	Area mAU *s	Height [mAU]	Area %
1	5.488	BV	0.1095	4623.56934	647.60217	49.2684
2	6.161	VB	0.1228	4760.87842	593.07776	50.7316

Totals : 9384.44775 1240.67993

*** End of Report ***

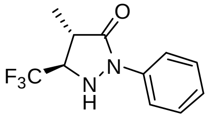

(+/-) - **4a**

Instrument 1 12/4/2014 9:41:51 PM Z Page 1 of 1

Data File C:\CHEM32\1\DATA\ZHOU-14\YZN005773.D
Sample Name: ZC-5-86F

Acq. Operator : Z
Acq. Instrument : Instrument 1 Location : Vial 1
Injection Date : 7/30/2014 10:16:46 AM
Acq. Method : C:\CHEM32\1\METHODS\DEF LC.M
Last changed : 7/30/2014 10:15:44 AM by Z
(modified after loading)
Analysis Method : C:\CHEM32\1\METHODS\DEF LC.M
Last changed : 12/4/2014 9:39:58 PM by Z
(modified after loading)
Sample Info : AD-H , H/i-PrOH = 70/30 0.7 mL/min, 30 oC, 254 nm

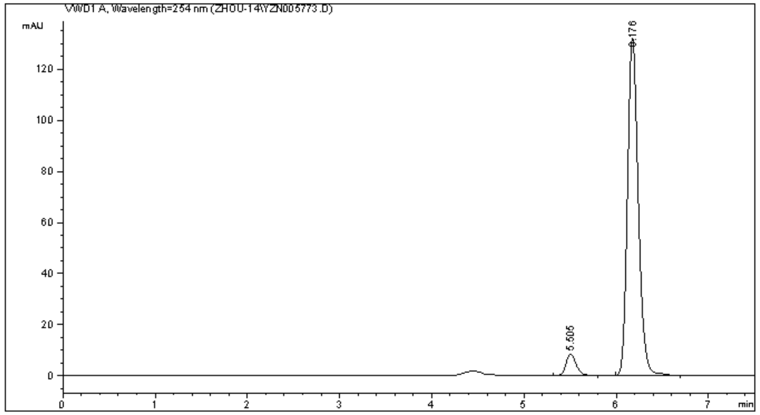

Area Percent Report

Sorted By : Signal
Multiplier: : 1.0000
Dilution: : 1.0000
Use Multiplier & Dilution Factor with ISTDs

Signal 1: VWD1 A, Wavelength=254 nm

Peak #	RetTime [min]	Type	Width [min]	Area mAU *s	Height [mAU]	Area %
1	5.505	BB	0.1176	63.91172	8.43166	5.6200
2	6.176	BB	0.1238	1073.29895	132.26836	94.3800

Totals : 1137.21067 140.70002

*** End of Report ***

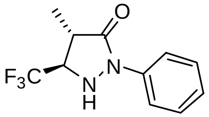

(-) - **4a**

Instrument 1 12/4/2014 9:42:04 PM Z Page 1 of 1

Data File C:\CHEM32\1\DATA\ZHOU-14\YZN005713.D
Sample Name: ZC-5-86A+-

===

Acq. Operator : Z
Acq. Instrument : Instrument 1 Location : Vial 1
Injection Date : 7/26/2014 10:30:10 AM
Acq. Method : C:\CHEM32\1\METHODS\DEF LC.M
Last changed : 7/26/2014 10:10:25 AM by Z
(modified after loading)
Analysis Method : C:\CHEM32\1\METHODS\DEF LC.M
Last changed : 12/4/2014 9:34:10 PM by Z
(modified after loading)
Sample Info : AD-H , H/i-PrOH = 70/30, 0.7 mL/min, 30 oC, 254 nm

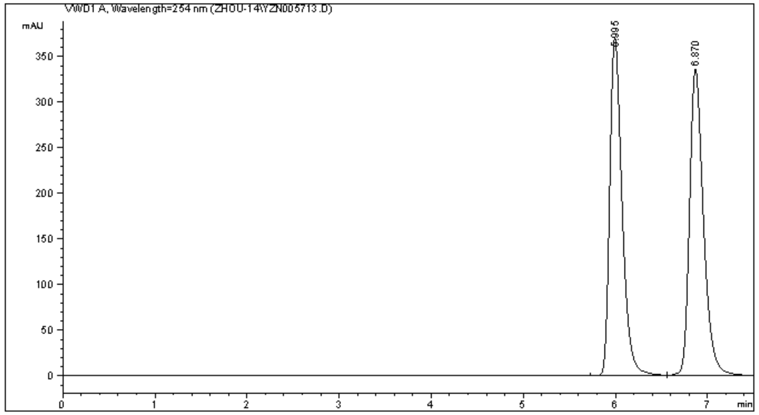

===
Area Percent Report
===

Sorted By : Signal
Multiplier: : 1.0000
Dilution: : 1.0000
Use Multiplier & Dilution Factor with ISTDs

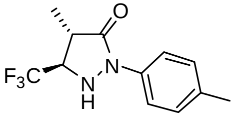

(+/-) **-4b**

Signal 1: VWD1 A, Wavelength=254 nm

Peak #	RetTime [min]	Type	Width [min]	Area mAU *s	Height [mAU]	Area %
1	5.995	VV	0.1421	3450.47607	370.58310	49.4396
2	6.870	VB	0.1604	3528.69922	335.83157	50.5604
Totals :				6979.17529	706.41467	

===
*** End of Report ***

Instrument 1 12/4/2014 9:34:15 PM Z Page 1 of 1

Data File C:\CHEM32\1\DATA\ZHOU-14\YZN005714.D
Sample Name: ZC-5-86A

===

Acq. Operator : Z
Acq. Instrument : Instrument 1 Location : Vial 1
Injection Date : 7/26/2014 10:42:42 AM
Acq. Method : C:\CHEM32\1\METHODS\DEF LC.M
Last changed : 7/26/2014 10:40:52 AM by Z
(modified after loading)
Analysis Method : C:\CHEM32\1\METHODS\DEF LC.M
Last changed : 12/4/2014 9:34:10 PM by Z
(modified after loading)
Sample Info : AD-H , H/i-PrOH = 70/30, 0.7 mL/min, 30 oC, 254 nm

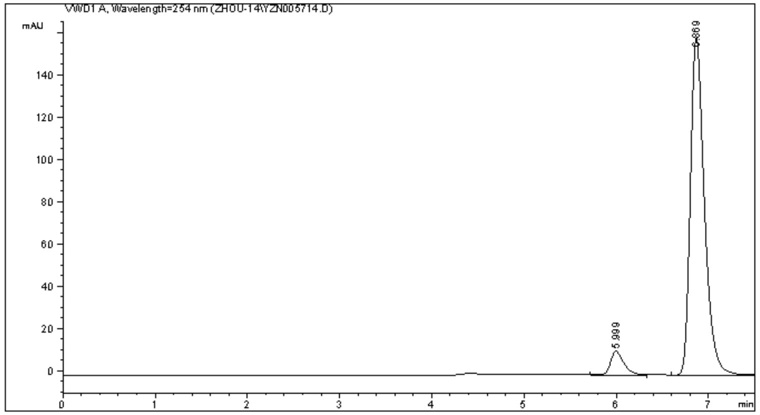

===
Area Percent Report
===

Sorted By : Signal
Multiplier: : 1.0000
Dilution: : 1.0000
Use Multiplier & Dilution Factor with ISTDs

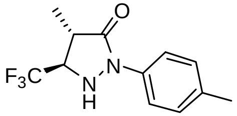

(-) **-4b**

Signal 1: VWD1 A, Wavelength=254 nm

Peak #	RetTime [min]	Type	Width [min]	Area mAU *s	Height [mAU]	Area %
1	5.999	VV	0.1505	112.12787	11.18441	6.2387
2	6.869	VB	0.1610	1685.17603	159.63228	93.7613
Totals :				1797.30389	170.81669	

===
*** End of Report ***

Instrument 1 12/4/2014 9:35:49 PM Z Page 1 of 1

```
Data File C:\CHEM32\1\DATA\ZHOU-14\YZN005775.D
Sample Name: ZC-5-86E+-

    =====================================================================
    Acq. Operator   : Z
    Acq. Instrument : Instrument 1                    Location : Vial 1
    Injection Date  : 7/30/2014 11:12:11 AM
    Acq. Method     : C:\CHEM32\1\METHODS\DEF LC.M
    Last changed    : 7/30/2014 10:47:53 AM by Z
                      (modified after loading)
    Analysis Method : C:\CHEM32\1\METHODS\DEF LC.M
    Last changed    : 12/4/2014 9:39:58 PM by Z
                      (modified after loading)
    Sample Info     : AD-H , H/i-PrOH = 70/30 0.7 mL/min, 30 oC, 254 nm
```

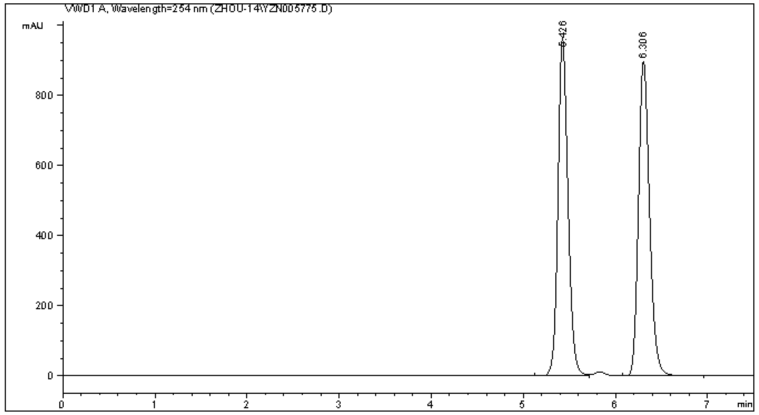


```
    =====================================================================
                             Area Percent Report
    =====================================================================

    Sorted By             :      Signal
    Multiplier:                  :      1.0000
    Dilution:                    :      1.0000
    Use Multiplier & Dilution Factor with ISTDs

    Signal 1: VWD1 A, Wavelength=254 nm

    Peak RetTime Type  Width     Area      Height     Area
      #   [min]        [min]  mAU   *s   [mAU   ]      %
    ----|-------|----|-------|----------|----------|--------|
       1   5.426 VV    0.1156 7279.71191  965.33405  49.1759
       2   6.306 VB    0.1297 7523.69189  898.46832  50.8241

    Totals :                  1.48034e4  1863.80237

    =====================================================================
                             *** End of Report ***
```

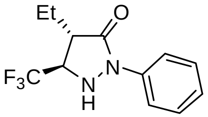

(+/-) -**4c**

Instrument 1 12/4/2014 9:40:03 PM Z

Page 1 of 1

```
Data File C:\CHEM32\1\DATA\ZHOU-14\YZN005808.D
Sample Name: ZC-5-87E

    =====================================================================
    Acq. Operator   : Z
    Acq. Instrument : Instrument 1                    Location : Vial 1
    Injection Date  : 8/14/2014 1:45:06 PM
    Acq. Method     : C:\CHEM32\1\METHODS\DEF LC.M
    Last changed    : 8/14/2014 1:26:59 PM by Z
                      (modified after loading)
    Analysis Method : C:\CHEM32\1\METHODS\DEF LC.M
    Last changed    : 12/4/2014 9:39:58 PM by Z
                      (modified after loading)
    Sample Info     : AD, H/i-PrOH = 70/30, 0.7 mL/min, 30 oC, 254 nm
```

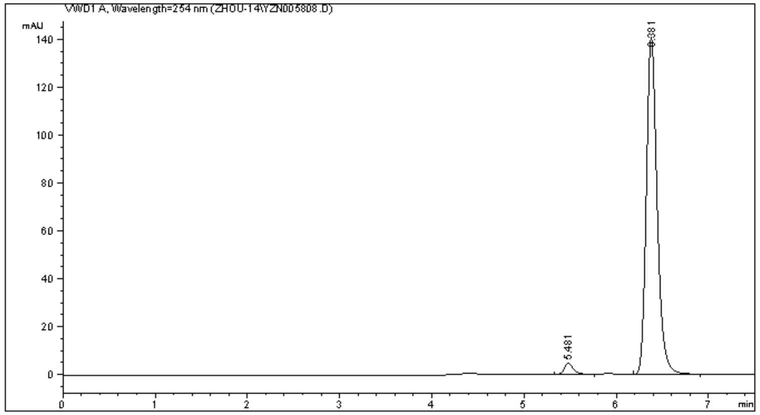


```
    =====================================================================
                             Area Percent Report
    =====================================================================

    Sorted By             :      Signal
    Multiplier:                  :      1.0000
    Dilution:                    :      1.0000
    Use Multiplier & Dilution Factor with ISTDs

    Signal 1: VWD1 A, Wavelength=254 nm

    Peak RetTime Type  Width     Area      Height     Area
      #   [min]        [min]  mAU   *s   [mAU   ]      %
    ----|-------|----|-------|----------|----------|--------|
       1   5.481 BB    0.1068   33.74635    4.71308   2.8550
       2   6.381 BB    0.1243 1148.25696  140.71727  97.1450

    Totals :                  1182.00331  145.43035

    =====================================================================
                             *** End of Report ***
```

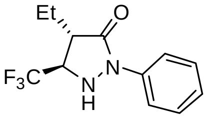

(-) -**4c**

Instrument 1 12/4/2014 9:40:50 PM Z

Page 1 of 1

Data File C:\CHEM32\1\DATA\ZHOU-14\YZN005841.D
Sample Name: ZC-5-90A+-

===

Acq. Operator : Z
Acq. Instrument : Instrument 1 Location : Vial 1
Injection Date : 8/15/2014 9:08:59 PM
Acq. Method : C:\CHEM32\1\METHODS\DEF LC.M
Last changed : 8/15/2014 9:06:53 PM by Z
(modified after loading)
Analysis Method : C:\CHEM32\1\METHODS\DEF LC.M
Last changed : 12/20/2014 10:57:17 AM by Z
(modified after loading)
Sample Info : OG-H, H/i-PrOH = 95/5, 0.7 mL/min, 30 oC, 254 nm

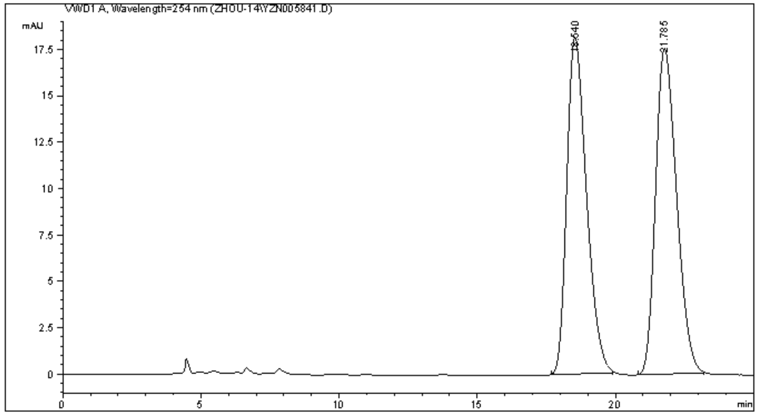

===
Area Percent Report
===

Sorted By : Signal
Multiplier: : 1.0000
Dilution: : 1.0000
Use Multiplier & Dilution Factor with ISTDs

Signal 1: VWD1 A, Wavelength=254 nm

Peak #	RetTime [min]	Type	Width [min]	Area mAU *s	Height [mAU]	Area %
1	18.540	BB	0.7551	897.38940	18.13141	49.1149
2	21.785	BB	0.8105	929.73413	17.54202	50.8851
Totals :				1827.12354	35.67343	

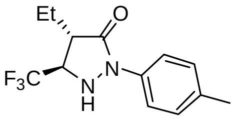

(+/-) -**4d**

===
*** End of Report ***

Instrument 1 12/20/2014 10:57:19 AM Z Page 1 of 1

Data File C:\CHEM32\1\DATA\ZHOU-14\YZN005842.D
Sample Name: ZC-5-90A

===

Acq. Operator : Z
Acq. Instrument : Instrument 1 Location : Vial 1
Injection Date : 8/15/2014 9:49:35 PM
Acq. Method : C:\CHEM32\1\METHODS\DEF LC.M
Last changed : 8/15/2014 9:36:53 PM by Z
(modified after loading)
Analysis Method : C:\CHEM32\1\METHODS\DEF LC.M
Last changed : 12/20/2014 10:58:16 AM by Z
(modified after loading)
Sample Info : OG-H, H/i-PrOH = 95/5, 0.7 mL/min, 30 oC, 254 nm

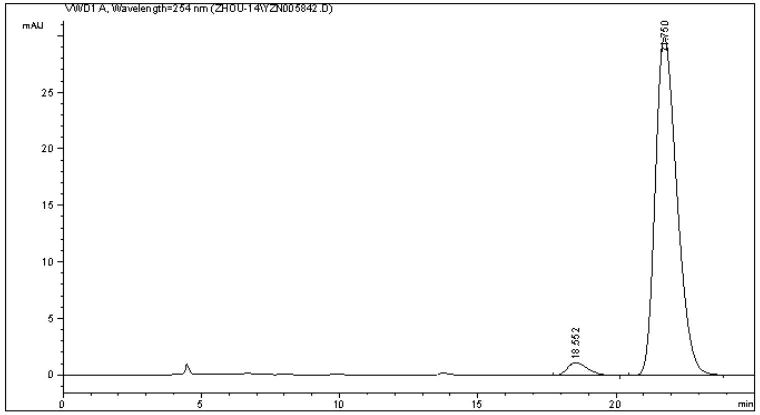

===
Area Percent Report
===

Sorted By : Signal
Multiplier: : 1.0000
Dilution: : 1.0000
Use Multiplier & Dilution Factor with ISTDs

Signal 1: VWD1 A, Wavelength=254 nm

Peak #	RetTime [min]	Type	Width [min]	Area mAU *s	Height [mAU]	Area %
1	18.552	BB	0.7550	60.09979	1.16728	3.5548
2	21.750	BB	0.8428	1630.55042	29.90775	96.4452
Totals :				1690.65020	31.07504	

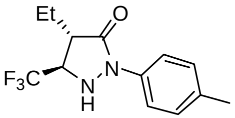

(+) -**4d**

===
*** End of Report ***

Instrument 1 12/20/2014 10:58:37 AM Z Page 1 of 1

Data File C:\CHEM32\1\DATA\ZHOU-14\YZN006631.D
Sample Name: ZC-5-90B+-

===

Acq. Operator : Z
Acq. Instrument : Instrument 1 Location : Vial 1
Injection Date : 12/13/2014 2:15:40 PM
Acq. Method : C:\CHEM32\1\METHODS\DEF LC.M
Last changed : 12/13/2014 1:45:53 PM by Z
(modified after loading)
Analysis Method : C:\CHEM32\1\METHODS\DEF LC.M
Last changed : 12/20/2014 10:55:06 AM by Z
(modified after loading)
Sample Info : AD-H, H/i-PrOH = 70/30, 0.7 mL/min, 30 oC, 254 nm

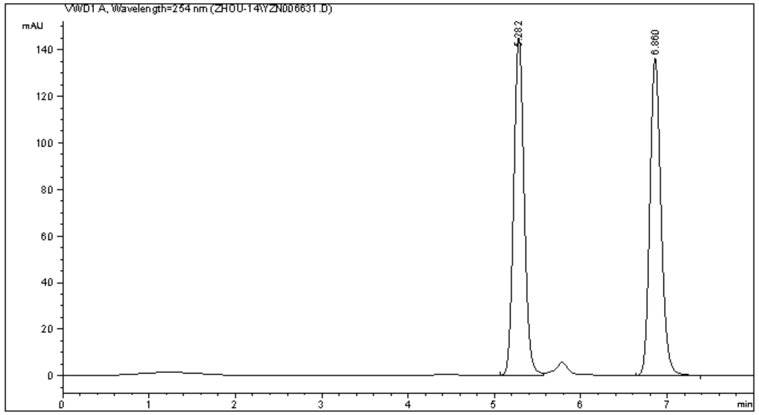

===
Area Percent Report
===

Sorted By : Signal
Multiplier: : 1.0000
Dilution: : 1.0000
Use Multiplier & Dilution Factor with ISTDs

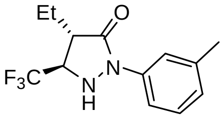

(+/-) -**4e**

Signal 1: VWD1 A, Wavelength=254 nm

Peak #	RetTime [min]	Type	Width [min]	Area mAU *s	Height [mAU]	Area %
1	5.282	BV	0.1296	1212.78735	144.99921	49.8640
2	6.860	BB	0.1380	1219.40527	136.18169	50.1360

Totals : 2432.19263 281.18089

===
*** End of Report ***

Instrument 1 12/20/2014 10:55:11 AM Z   Page 1 of 1

Data File C:\CHEM32\1\DATA\ZHOU-14\YZN005827.D
Sample Name: ZC-5-90B

===

Acq. Operator : Z
Acq. Instrument : Instrument 1 Location : Vial 1
Injection Date : 8/14/2014 9:16:36 PM
Acq. Method : C:\CHEM32\1\METHODS\DEF LC.M
Last changed : 8/14/2014 9:12:39 PM by Z
(modified after loading)
Analysis Method : C:\CHEM32\1\METHODS\DEF LC.M
Last changed : 12/4/2014 9:44:22 PM by Z
(modified after loading)
Sample Info : AD, H/i-PrOH = 70/30, 0.7 mL/min, 30 oC, 254 nm

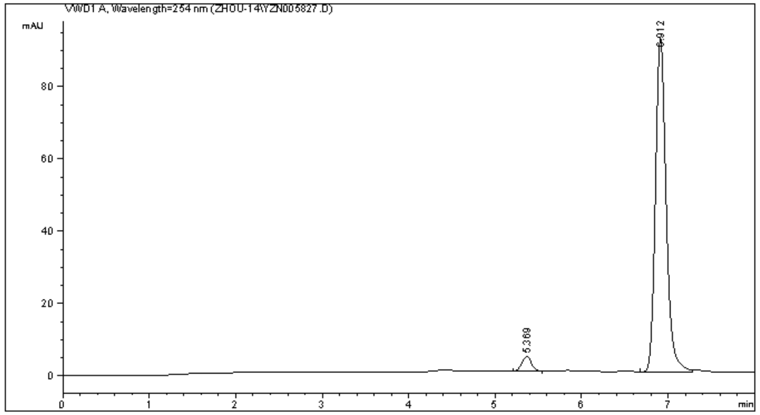

===
Area Percent Report
===

Sorted By : Signal
Multiplier: : 1.0000
Dilution: : 1.0000
Use Multiplier & Dilution Factor with ISTDs

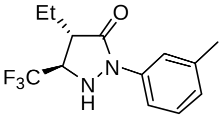

(+) -**4e**

Signal 1: VWD1 A, Wavelength=254 nm

Peak #	RetTime [min]	Type	Width [min]	Area mAU *s	Height [mAU]	Area %
1	5.369	BV	0.1261	32.52824	4.09973	3.9345
2	6.912	VV	0.1308	794.20612	92.47683	96.0655

Totals : 826.73436 96.57656

===
*** End of Report ***

Instrument 1 12/4/2014 9:46:57 PM Z   Page 1 of 1

Data File C:\CHEM32\1\DATA\ZHOU-14\YZN005769.D
Sample Name: ZC-5-86D+-

===

Acq. Operator : Z
Acq. Instrument : Instrument 1 Location : Vial 1
Injection Date : 7/30/2014 9:12:32 AM
Acq. Method : C:\CHEM32\1\METHODS\DEF LC.M
Last changed : 7/30/2014 9:11:39 AM by Z
(modified after loading)
Analysis Method : C:\CHEM32\1\METHODS\DEF LC.M
Last changed : 12/4/2014 9:37:43 PM by Z
(modified after loading)
Sample Info : AD-H , H/i-PrOH = 70/30 0.7 mL/min, 30 oC, 254 nm

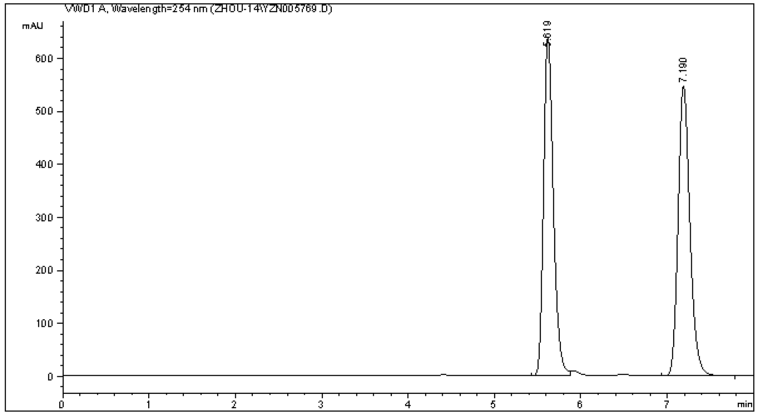

===
Area Percent Report
===

Sorted By : Signal
Multiplier: : 1.0000
Dilution: : 1.0000
Use Multiplier & Dilution Factor with ISTDs

Signal 1: VWD1 A, Wavelength=254 nm

Peak #	RetTime [min]	Type	Width [min]	Area mAU *s	Height [mAU]	Area %
1	5.619	VV	0.1213	5038.87988	637.91187	49.2415
2	7.190	BB	0.1457	5194.11768	547.28534	50.7585

Totals : 1.02330e4 1185.19720

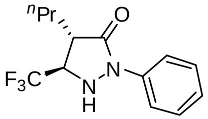

(+/-) -**4f**

===
*** End of Report ***

Instrument 1 12/4/2014 9:37:51 PM Z Page 1 of 1

Data File C:\CHEM32\1\DATA\ZHOU-14\YZN005768.D
Sample Name: ZC-5-86D

===

Acq. Operator : Z
Acq. Instrument : Instrument 1 Location : Vial 1
Injection Date : 7/30/2014 8:48:37 AM
Acq. Method : C:\CHEM32\1\METHODS\DEF LC.M
Last changed : 7/30/2014 8:16:45 AM by Z
(modified after loading)
Analysis Method : C:\CHEM32\1\METHODS\DEF LC.M
Last changed : 12/4/2014 9:37:43 PM by Z
(modified after loading)
Sample Info : AD-H , H/i-PrOH = 70/30 0.7 mL/min, 30 oC, 254 nm

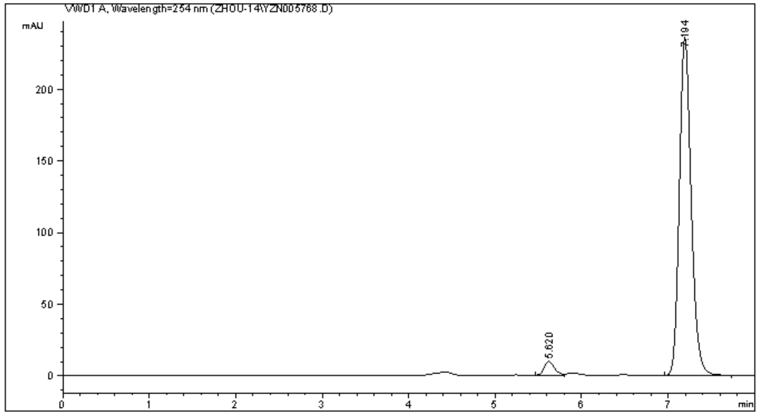

===
Area Percent Report
===

Sorted By : Signal
Multiplier: : 1.0000
Dilution: : 1.0000
Use Multiplier & Dilution Factor with ISTDs

Signal 1: VWD1 A, Wavelength=254 nm

Peak #	RetTime [min]	Type	Width [min]	Area mAU *s	Height [mAU]	Area %
1	5.620	BV	0.1283	79.75753	9.52649	3.4421
2	7.194	BB	0.1457	2237.34302	235.64395	96.5579

Totals : 2317.10055 245.17044

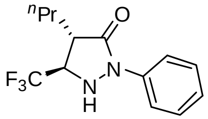

(-) -**4f**

===
*** End of Report ***

Instrument 1 12/4/2014 9:38:28 PM Z Page 1 of 1

Data File C:\CHEM32\1\DATA\ZHOU-14\YZN005133.D
Sample Name: ZC-5-42

===

Acq. Operator : Z
Acq. Instrument : Instrument 1 Location : Vial 1
Injection Date : 5/27/2014 2:58:09 PM
Acq. Method : C:\CHEM32\1\METHODS\DEF LC.M
Last changed : 5/27/2014 2:35:10 PM by Z
(modified after loading)
Analysis Method : C:\CHEM32\1\METHODS\DEF LC.M
Last changed : 12/5/2014 10:35:55 AM by Z
(modified after loading)
Sample Info : AD-H, H/i-PrOH = 70/30, 0.7 mL/min, 30 oC, 254 nm

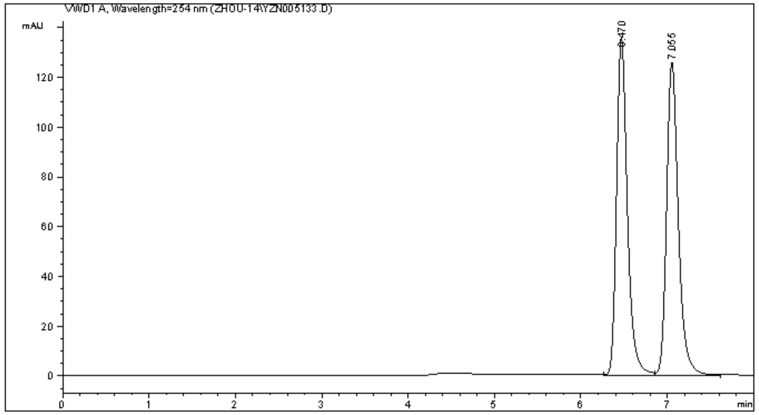

===
Area Percent Report
===

Sorted By : Signal
Multiplier: : 1.0000
Dilution: : 1.0000
Use Multiplier & Dilution Factor with ISTDs

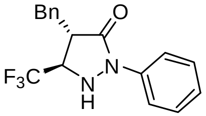

(+/-) **-4g**

Signal 1: VWD1 A, Wavelength=254 nm

Peak #	RetTime [min]	Type	Width [min]	Area mAU *s	Height [mAU]	Area %
1	6.470	VV	0.1320	1179.32263	135.60698	49.8211
2	7.055	VB	0.1436	1187.79102	125.86638	50.1789

Totals : 2367.11365 261.47336

===
*** End of Report ***

Instrument 1 12/5/2014 10:36:32 AM Z Page 1 of 1

Data File C:\CHEM32\1\DATA\ZHOU-14\YZN005774.D
Sample Name: ZC-5-85

===

Acq. Operator : Z
Acq. Instrument : Instrument 1 Location : Vial 1
Injection Date : 7/30/2014 10:32:04 AM
Acq. Method : C:\CHEM32\1\METHODS\DEF LC.M
Last changed : 7/30/2014 10:30:12 AM by Z
(modified after loading)
Analysis Method : C:\CHEM32\1\METHODS\DEF LC.M
Last changed : 12/5/2014 10:35:55 AM by Z
(modified after loading)
Sample Info : AD-H , H/i-PrOH = 70/30 0.7 mL/min, 30 oC, 254 nm

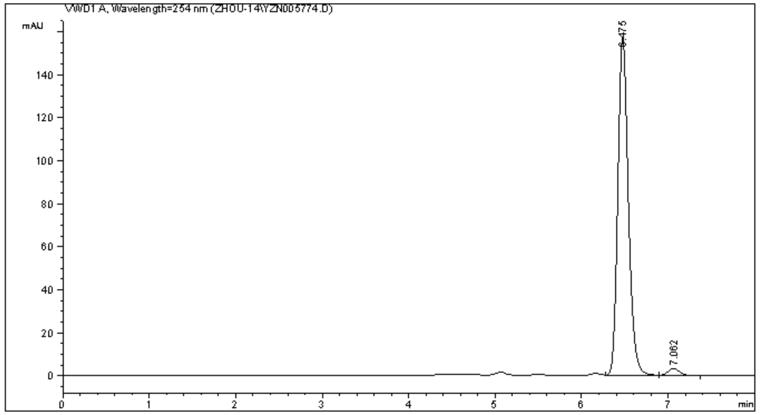

===
Area Percent Report
===

Sorted By : Signal
Multiplier: : 1.0000
Dilution: : 1.0000
Use Multiplier & Dilution Factor with ISTDs

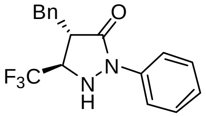

(+) **-4g**

Signal 1: VWD1 A, Wavelength=254 nm

Peak #	RetTime [min]	Type	Width [min]	Area mAU *s	Height [mAU]	Area %
1	6.475	VV	0.1295	1330.98718	157.02785	97.7393
2	7.062	VB	0.1438	30.78589	3.25714	2.2607

Totals : 1361.77308 160.28499

===
*** End of Report ***

Instrument 1 12/5/2014 10:35:59 AM Z Page 1 of 1

Data File G:\吡唑\机理实验产物图\44C\YZ006700.D
Sample Name: ZC-6-44C+-

===

Acq. Operator : ZHOU
Acq. Instrument : Instrument 1 Location : Vial 1
Injection Date : 11/18/2014 12:24:36 PM
Acq. Method : C:\HPCHEM\1\METHODS\DEF LC1.M
Last changed : 11/18/2014 12:21:56 PM by ZHOU
(modified after loading)
Analysis Method : C:\CHEM32\1\METHODS\DEF LC.M
Last changed : 12/20/2014 11:20:25 AM by Z
(modified after loading)
Sample Info : OD-H, H/i-PrOH = 90/10, 0.8 mL/min, 30 oC, 254 nm

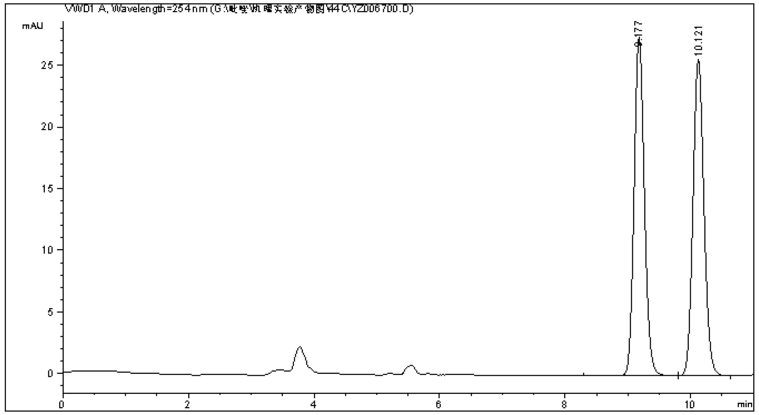

===
Area Percent Report
===

Sorted By : Signal
Multiplier: : 1.0000
Dilution: : 1.0000
Use Multiplier & Dilution Factor with ISTDs

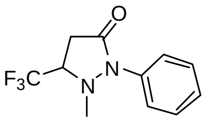

(+/-) - **7**

Signal 1: VWD1 A, Wavelength=254 nm

Peak #	RetTime [min]	Type	Width [min]	Area mAU *s	Height [mAU]	Area %
1	9.177	VV	0.1770	313.46347	27.42233	50.1650
2	10.121	VV	0.1884	311.40149	25.62359	49.8350

Totals : 624.86496 53.04592

===
*** End of Report ***

Instrument 1 12/20/2014 11:20:27 AM Z Page 1 of 1

Data File G:\吡唑\机理实验产物图\44C\YZ006701.D
Sample Name: ZC-6-44C

===

Acq. Operator : ZHOU
Acq. Instrument : Instrument 1 Location : Vial 1
Injection Date : 11/18/2014 12:37:24 PM
Acq. Method : C:\HPCHEM\1\METHODS\DEF LC1.M
Last changed : 11/18/2014 12:36:11 PM by ZHOU
(modified after loading)
Analysis Method : C:\CHEM32\1\METHODS\DEF LC.M
Last changed : 12/20/2014 11:19:38 AM by Z
(modified after loading)
Sample Info : OD-H, H/i-PrOH = 90/10, 0.8 mL/min, 30 oC, 254 nm

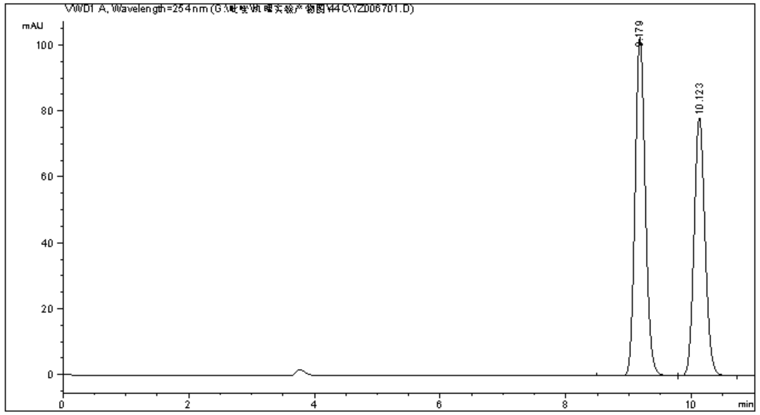

===
Area Percent Report
===

Sorted By : Signal
Multiplier: : 1.0000
Dilution: : 1.0000
Use Multiplier & Dilution Factor with ISTDs

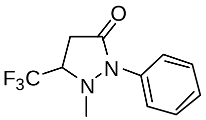

(+) - **7**

Signal 1: VWD1 A, Wavelength=254 nm

Peak #	RetTime [min]	Type	Width [min]	Area mAU *s	Height [mAU]	Area %
1	9.179	VV	0.1756	1159.77759	102.57599	54.9986
2	10.123	VV	0.1881	948.96246	78.25777	45.0014

Totals : 2108.74005 180.83376

===
*** End of Report ***

Instrument 1 12/20/2014 11:19:40 AM Z Page 1 of 1

Data File G:\吡唑\机理实验产物图\44A\YZ006699.D
Sample Name: ZC-6-44A+-

```
=====================================================================
Acq. Operator   : ZHOU
Acq. Instrument : Instrument 1                    Location : Vial 1
Injection Date  : 11/18/2014 12:06:12 PM
Acq. Method     : C:\HPCHEM\1\METHODS\DEF LC1.M
Last changed    : 11/18/2014 11:57:14 AM by ZHOU
                  (modified after loading)
Analysis Method : C:\CHEM32\1\METHODS\DEF LC.M
Last changed    : 12/20/2014 11:18:08 AM by Z
                  (modified after loading)
Sample Info     : OD-H, H/i-PrOH = 90/10, 0.8 mL/min, 30 oC, 254 nm
```

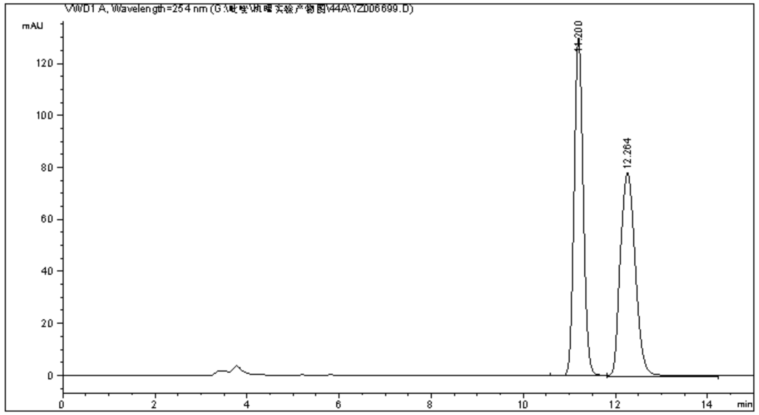


```
=====================================================================
                         Area Percent Report
=====================================================================

Sorted By             :      Signal
Multiplier:                   :      1.0000
Dilution:                     :      1.0000
Use Multiplier & Dilution Factor with ISTDs


Signal 1: VWD1 A, Wavelength=254 nm

Peak RetTime Type  Width     Area      Height     Area
  #   [min]        [min]  mAU   *s    [mAU   ]      %
----|-------|----|-------|----------|----------|--------|
   1  11.200 VV     0.2124 1784.32935  130.15268  50.3100
   2  12.264 VB     0.3680 1762.34241   78.13858  49.6900

Totals :                   3546.67175  208.29126

=====================================================================
                          *** End of Report ***
```

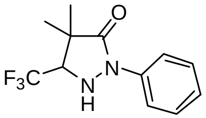

(+/-) -**9**

Instrument 1 12/20/2014 11:18:17 AM Z   Page 1 of 1

Data File G:\吡唑\机理实验产物图\44A\YZ006698.D
Sample Name: ZC-6-44A

```
=====================================================================
Acq. Operator   : ZHOU
Acq. Instrument : Instrument 1                    Location : Vial 1
Injection Date  : 11/18/2014 11:40:02 AM
Acq. Method     : C:\HPCHEM\1\METHODS\DEF LC1.M
Last changed    : 11/18/2014 11:21:46 AM by ZHOU
                  (modified after loading)
Analysis Method : C:\CHEM32\1\METHODS\DEF LC.M
Last changed    : 12/20/2014 11:18:08 AM by Z
                  (modified after loading)
Sample Info     : OD-H, H/i-PrOH = 90/10, 0.8 mL/min, 30 oC, 254 nm
```

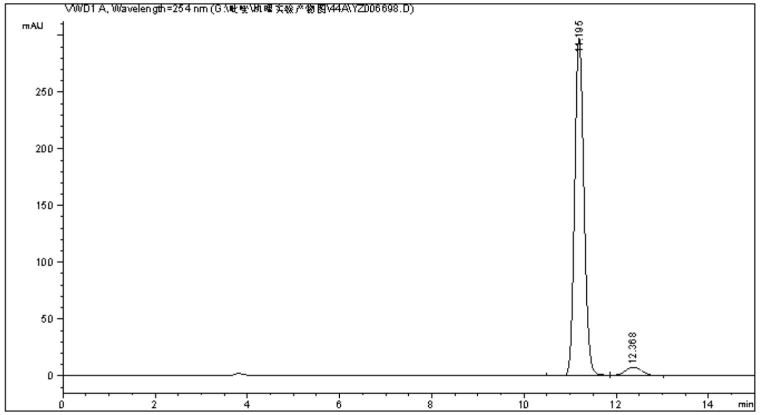


```
=====================================================================
                         Area Percent Report
=====================================================================

Sorted By             :      Signal
Multiplier:                   :      1.0000
Dilution:                     :      1.0000
Use Multiplier & Dilution Factor with ISTDs


Signal 1: VWD1 A, Wavelength=254 nm

Peak RetTime Type  Width     Area      Height     Area
  #   [min]        [min]  mAU   *s    [mAU   ]      %
----|-------|----|-------|----------|----------|--------|
   1  11.195 BV     0.2188 4178.64844  298.17264  95.2759
   2  12.368 VV     0.4133  207.19157    7.69088   4.7241

Totals :                   4385.84001  305.86351

=====================================================================
                          *** End of Report ***
```

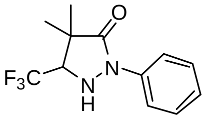

(+) - **9**

Instrument 1 12/20/2014 11:18:34 AM Z   Page 1 of 1
